# Modeling, Synthesis, and Biological Evaluation of Potential Retinoid-X-Receptor (RXR) Selective Agonists: Analogs of 4-[1-(3,5,5,8,8-Pentamethyl-5,6,7,8-tetrahyro-2-naphthyl)ethynyl]benzoic Acid (Bexarotene) and 6-(Ethyl(4-isobutoxy-3-isopropylphenyl)amino)nicotinic Acid (NEt-4IB)

**DOI:** 10.3390/ijms222212371

**Published:** 2021-11-16

**Authors:** Peter W. Jurutka, Orsola di Martino, Sabeeha Reshi, Sanchita Mallick, Zhela L. Sabir, Lech J. P. Staniszewski, Ankedo Warda, Emma L. Maiorella, Ani Minasian, Jesse Davidson, Samir J. Ibrahim, San Raban, Dena Haddad, Madleen Khamisi, Stephanie L. Suban, Bradley J. Dawson, Riley Candia, Joseph W. Ziller, Ming-Yue Lee, Chang Liu, Wei Liu, Pamela A. Marshall, John S. Welch, Carl E. Wagner

**Affiliations:** 1 School of Mathematical and Natural Sciences, Arizona State University, Glendale, AZ 85306, USA; Peter.Jurutka@asu.edu (P.W.J.); sreshi@asu.edu (S.R.); Sanchita.mallick@gmail.com (S.M.); zlsabir@asu.edu (Z.L.S.); lech@email.arizona.edu (L.J.P.S.); anwarda@email.arizona.edu (A.W.); emaiorella@cox.net (E.L.M.); anicminasian@gmail.com (A.M.); jrdavid3@asu.edu (J.D.); sjibrahim7@gmail.com (S.J.I.); sanyousif96@gmail.com (S.R.); denahaddad227@gmail.com (D.H.); madleenkhamisi@gmail.com (M.K.); ssuban@cox.net (S.L.S.); bradleyjdawson@gmail.com (B.J.D.); rcandia@asu.edu (R.C.); pamela.marshall@asu.edu (P.A.M.); 2 Basic Medical Sciences, University of Arizona College of Medicine, Phoenix, AZ 85004, USA; 3 Department of Internal Medicine, Washington University, St. Louis, MO 63110, USA; orsoladimartino@wustl.edu (O.d.M.); jwelch@wustl.edu (J.S.W.); 4 Department of Chemistry, University of California, Irvine, CA 92697, USA; jziller@uci.edu; 5 School of Molecular Sciences, Arizona State University, Tempe, AZ 85201, USA; mlee171@asu.edu (M.-Y.L.); cliu207@asu.edu (C.L.); w.liu@asu.edu (W.L.)

**Keywords:** retinoid-X-receptor, retinoid, rexinoid, leukemia, small molecule therapeutic, structure–activity relationship

## Abstract

Five novel analogs of 6-(ethyl)(4-isobutoxy-3-isopropylphenyl)amino)nicotinic acid—or NEt-4IB—in addition to seven novel analogs of 4-[1-(3,5,5,8,8-pentamethyl-5,6,7,8-tetrahydro-2-naphthyl)ethynyl]benzoic acid (bexarotene) were prepared and evaluated for selective retinoid-X-receptor (RXR) agonism alongside bexarotene (**1**), a FDA-approved drug for cutaneous T-cell lymphoma (CTCL). Bexarotene treatment elicits side-effects by provoking or disrupting other RXR-dependent pathways. Analogs were assessed by the modeling of binding to RXR and then evaluated in a human cell-based RXR-RXR mammalian-2-hybrid (M2H) system as well as a RXRE-controlled transcriptional system. The analogs were also tested in KMT2A-MLLT3 leukemia cells and the EC_50_ and IC_50_ values were determined for these compounds. Moreover, the analogs were assessed for activation of LXR in an LXRE system as drivers of ApoE expression and subsequent use as potential therapeutics in neurodegenerative disorders, and the results revealed that these compounds exerted a range of differential LXR-RXR activation and selectivity. Furthermore, several of the novel analogs in this study exhibited reduced RARE cross-signaling, implying RXR selectivity. These results demonstrate that modification of partial agonists such as NEt-4IB and potent rexinoids such as bexarotene can lead to compounds with improved RXR selectivity, decreased cross-signaling of other RXR-dependent nuclear receptors, increased LXRE-heterodimer selectivity, and enhanced anti-proliferative potential in leukemia cell lines compared to therapeutics such as **1**.

## 1. Introduction

The human retinoid X receptors consist of three identified isoforms (α, β, γ) [1,2] with one or more of the isoforms exhibiting expression in every human tissue type where the receptor regulates—sometimes in partnership with other nuclear receptors—gene transcription, often stimulated by receptor-specific molecular signaling. The RXRs display a remarkable versatility unknown among other nuclear receptors (NRs) making up a transcriptional modulator superfamily because they join with many of the NRs to create heterodimers that actively modulate the pathways central to cell differentiation, metabolism, proliferation, and migration. Some of the critical receptor pathways where RXR participates as an essential component to realize functional responses include the liver X receptor (LXR), the thyroid hormone receptor (TR), the peroxisome proliferator-activated receptor (PPAR), the vitamin D receptor (VDR), and the retinoic acid receptor (RAR), to name a few. All NRs control gene expression, primarily by regulating transcription and usually in response to the presence of an associated receptor ligand and their obligate partnering receptor. Receptor ligands, often endogenous molecules, bind to the receptor’s ligand-binding domain (LBD), which, in turn, compels the receptor to adopt a conformation that can then dimerize with an additional receptor, recruit co-factors, and ultimately bind with high affinity to a specific hormone responsive element (HRE) that the receptor regulates on DNA. Increasingly, HREs are being identified considerably up- or downstream from their moderated genes; however, a large number of HREs have also been identified close to or within the promoter region of the regulated genes. The HREs exhibited sequence specificity, consisting of two repeat hexads of half sites punctuated by a specified quantity of spacers separating those direct, inverted, or everted repeats [3]. VDRs, TRs, and RAR HREs include half-sites separated by 3-, 4-, and 5-nucleotide spacers, respectively [4,5].

Initially, TRs, VDRs, and RARs were presumed to bind to their HREs as homodimers [6], though they were later discovered to associate with RXR as a prerequisite to binding and activating their HREs [7]. Zhang and colleagues first reported that 9-*cis*-retinoic acid (9-*cis*-RA)—a naturally occurring isomer of all-*trans*-retinoic acid (ATRA)—is an RXR-specific ligand that functions as an agonist where its binding to RXR compels the formation of RXR homodimers and subsequent association with RXR responsive elements (RXREs) [8]. When RXR associates with other NRs as a heterodimer, the heterodimer does not need to possess a RXR-specific ligand in the LBD for RXR. For example, the RXR-VDR heterodimer has been reported to function without a ligand bound to RXR [9]. Alternatively, some RXR heterodimers exhibit enhanced activity with RXR-specific ligands (rexinoids) bound to RXRs’ LBD, as in the case of the RXR-LXR heterodimer.[10] Considering this degree of versatility—the necessity for RXR to partner with several NRs with or without ligands for those NRs to function—RXR has reasonably been termed the master receptor [11].

Numerous RXR-studies, comprising multitudes of rexinoids with different partnering NRs, have distilled two primary RXR heterodimer classifications—the so-called permissive and non-permissive RXR heterodimers [12]. Only the heteropartner’s agonists can activate purely nonpermissive RXR heterodimers, whereas either the heteropartner’s agonists or rexinoids can activate permissive RXR heterodimers. The RXR-RAR, RXR-TR, and RXR-VDR heterodimers have all been characterized as non-permissive. In most, but not all conditions, the RXR partnering receptor for the VDR and TR heterodimers was “silent.” The RXR-RAR heterodimer, on the other hand, showed enhanced activation by both certain rexinoids and RAR-specific agonists. Specific RXR agonists, in fact, have shown activation of RXR-RAR in the absence of RAR-specific agonists [13]. The primary classification of RXR-RAR as nonpermissive has evolved in light of these observations to have a more accurate “conditionally nonpermissive” designation. The RXR-LXR, RXR-PPAR, and RXR-FXR heterodimers, alternatively, are all known to be fully permissive.

Both permissive and nonpermissive RXR heterodimers often give rise to pleiotropic effects from exposure to potent rexinoids—the former by stimulating RXR heterodimer pathways and the later by titrating a finite pool of RXR away from participating in the proper formation and functioning of those nonpermissive RXR heterodimers. This potential for pleiotropy has frustrated the clinical development of rexinoids for therapeutic applications. Rexinoids such as 9-*cis*-RA can arrest the functioning of the RXR-VDR [14,15,16] and RXR-TR [17] heterodimers. Similarly, molecular signals such as 1,25-dihydroxyvitamin D_3_ (1,25D) or T3 promote RXR-VDR or RXR-TR formation, depleting RXR availability, and thereby inhibiting alternative RXR-dependent mechanisms of action. This so-called cross-receptor squelching is exemplified by the loss of VDR function via T_3_-TR-RXR-modulated inhibition [18,19] and similarly by the loss of TR translational activity concomitant with 1,25D-VDR-RXR-activation [17,20]; however, the inhibition by crosstalk in the former examples likely concerns more than just the depletion of RXR. Nevertheless, the two overarching characteristics concerning the development of rexinoid therapeutics that exert fewer side effects and greater benefits comprise selectivity and potency [21]. Thus, an approach to modify a parent RXR-agonist’s structure may impact both potency and RXR-heterodimer selectivity, leading to improved pleotropic profiles by generating specific NR modulators (SNuRMs) [22].

**Figure 1:**
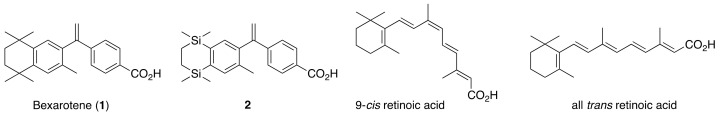
Structures of bexarotene (**1**), disilabexarotene (**2**), 9-*cis* retinoic acid, and all trans retinoic acid.

Several rexinoid SNuRMs are being investigated as drug targets, particularly in the case of cancer where selective RXR over RAR activation results in chemotherapeutic effects in many human cancers and avoids potential RAR toxicities [23]. Following several studies [24,25] that modeled and tested compounds evolved from 9-*cis* retinoic acid, an isomer of all trans retinoic acid (ATRA), 4-[1-(3,5,5,8,8-pentamethyltetralin-2-yl)ethynyl]benzoic acid (**1**) [26] has emerged as a lead RXR-selective, potent synthetic agonist, and although several other candidates have displayed equally potent if not superior profiles, Ligand Pharmaceuticals Inc. was granted FDA approval of **1**, known more widely as bexarotene, to treat cutaneous T-cell lymphoma (CTCL). Several studies have reported structural analogs of bexarotene such as disilabexarotene (**2**) [27], for example, which have been shown to exhibit similar activation of RXR (Figure 1).

While bexarotene (**1**) was first approved for the treatment of CTCL, it has also been tested in breast cancer [28], colon cancer [29], and lung cancer models [30]. In fact, a proof-of-concept (POC) clinical trial reported therapeutic benefits for use of **1** in non-small cell lung cancer [31,32], and bexarotene can be prescribed off-label for this disease. A mounting number of studies have linked cell-proliferation suppression and combination-chemotherapeutic apoptosis synergy with RXR-controlled pathways. Bexarotene (**1**) and numerous other synthetic rexinoids have also demonstrated positive impacts in non-insulin-dependent diabetes mellitus (NIDDM) mouse models, arising from metabolism regulation by RXR:PPAR [33]. While bexarotene (**1**) is predominantly RXR-selective and avoids significant RAR-activation, patients treated with **1** often experience hypothyroidism [34], hyperlipidemia, and occasionally cutaneous toxicity. Bexarotene (**1**), similar to 9-*cis*-RA, incites these side effects by disrupting nonpermissive heterodimers—hypothyroidism by RXR-TR [35] disruption—or stimulating the permissive heterodimers—hyperlipidemia via RXR-LXR activation [36,37] and cutaneous toxicity [38] from RAR activity at high dose concentrations. A number of groups are actively designing rexinoids with greater potency and specificity toward RXR-homodimer formation, in order to mitigate impacts on at least the permissive RXR heterodimer pathways. Adding to the urgency of developing novel rexinoids possessing attenuated side effect profiles, compound **1** has shown some promise in neurodegenerative disease models such as Parkinson’s disease [39] and Alzheimer’s disease (AD) [40]. Moreover, several novel rexinoids were recently reported to be equally or more effective at modulating gene expression on LXREs and NBREs and are thus superior at inducing ApoE and tyrosine hydroxylase, two genes whose enhanced expression is thought to mitigate the pathophysiology associated with Parkinson’s and Alzheimer’s diseases [41]. Significantly, a POC trial of **1** in AD patients exhibiting moderate symptoms demonstrated a statistically significant clearance of soluble amyloid beta in non-apoE 4 genotypes [42]. Furthermore, bexarotene (**1**) exhibited one of the best profiles—similar to that of remdesivir—in preventing SARS-CoV-2 infection in vitro in a recently reported robust screening assay of a 1528 FDA-approved drug library that identified four drugs that were active against the virus [43]. Bexarotene has also been shown to reduce inflammation [44] as well as decrease CL22 production by M2-polarized tumor-associated macrophages [45], which then modulates the tumor microenvironment. Furthermore, bexarotene is also being explored for a novel treatment of Cushing’s disease [46] and glioma [47].

**Figure 2:**
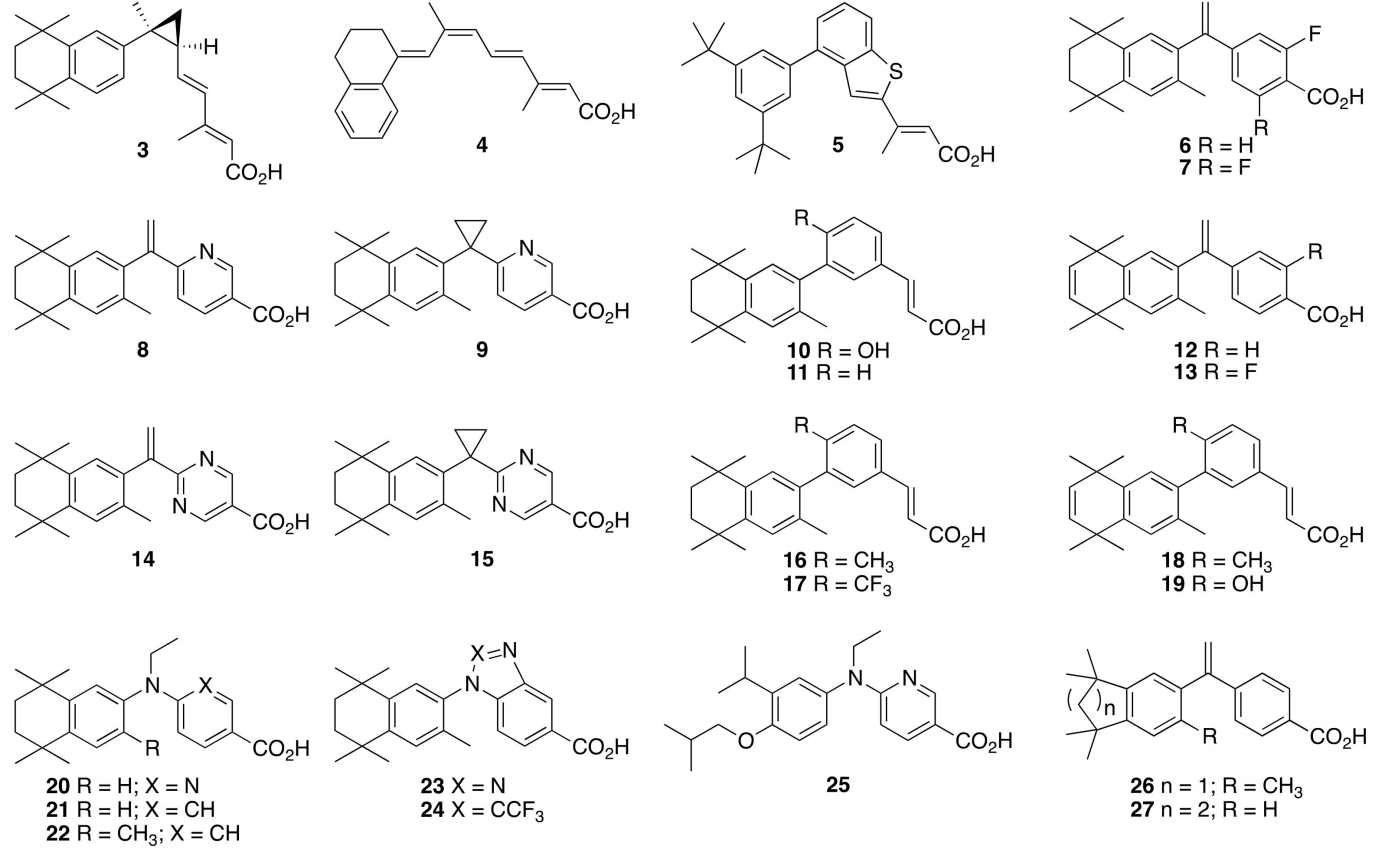
Structures of reported rexinoids **3**–**27**.

Employing modeling and structural features of reported rexinoids as starting points, many groups have successfully developed novel rexinoids with unique profiles. One such rexinoid that has been examined as a potential therapeutic for several human cancer and neurodegenerative diseases is IRX-4204 (**3**) [48], which was shown to activate RXR most potently compared to its other stereoisomer. Another well-studied rexinoid known as 9cUAB30 (**4**) [49] is currently in clinical trials for early stage mammary cancer [50,51,52], and several methylated variants of **4** [53,54] have helped demonstrate why **4** does not incite hyperlipidemia through RXR-LXR agonism compared to other moderately potent rexinoids. Boehm and colleagues have described unbranched trienoic acids [55] as well as analogous compounds containing a single [56] or multiple-fused [57] aryl rings—compound **5** [57] exemplifying the latter. Our group reported a mono-fluoro-bexarotene (**6**) [58] and a difluoro-bexarotene (**7**) [59] that displayed increased RXR activity relative to bexarotene. Compounds **8** [60] and LGD100268 (**9**) [60] both exhibited increased RXR activity in a CV-1 cell line versus **1**. The acrylic acids **10** (CD3254) [61] and **11** (CD2915) [62] possess similar potency for RXR agonism as **1**. Compound **12** [63,64] possesses a single unsaturation in the aliphatic ring system as its only structural difference from **1**. We used compounds **8**–**12** as starting points to prepare analogous rexinoids **13**–**19** [21] with unique gene expression and side-effect profiles in vivo [65]. Indeed, pyrimidine bexarotene (**14**) and pyrimidine LGD100268 (**15**) both showed improved therapeutic profiles over **1** in a mouse model of lung cancer [30]. Kakuta’s group reported the highly potent rexinoid **20** (NEt-TMN) [66,67,68,69,70], where NEt-TMN analogs **21** [71,72,73] and **22** [71,72] have also shown high potency in addition to several other NEt-TMN analogs that our team has described [74]. Kakuta’s group also reported the partial RXR agonist **23** [68] and a partial RXR agonist analog **24** [70] in pursuit of novel treatments for type II diabetes. A new partial RXR agonist **25** (NEt-4IB) [75] reported by Kakuta’s group has also shown promise in mouse models of diabetes and pulmonary emphysema [76], and we were interested in testing compound **25** and a few analogs of **25** for their activity and anti-proliferative properties in vitro, since we expect a reduced side-effect profile via RXR-dependent cross-signaling for these types of compounds. Finally, the indanyl-compound **26** [77] was reported in a patent for novel RXR agonists by Boehm, Heyman, and Lin, and compound **27** [26] was originally reported alongside **1** and showed similar activity (Figure 2).

**Figure 3:**
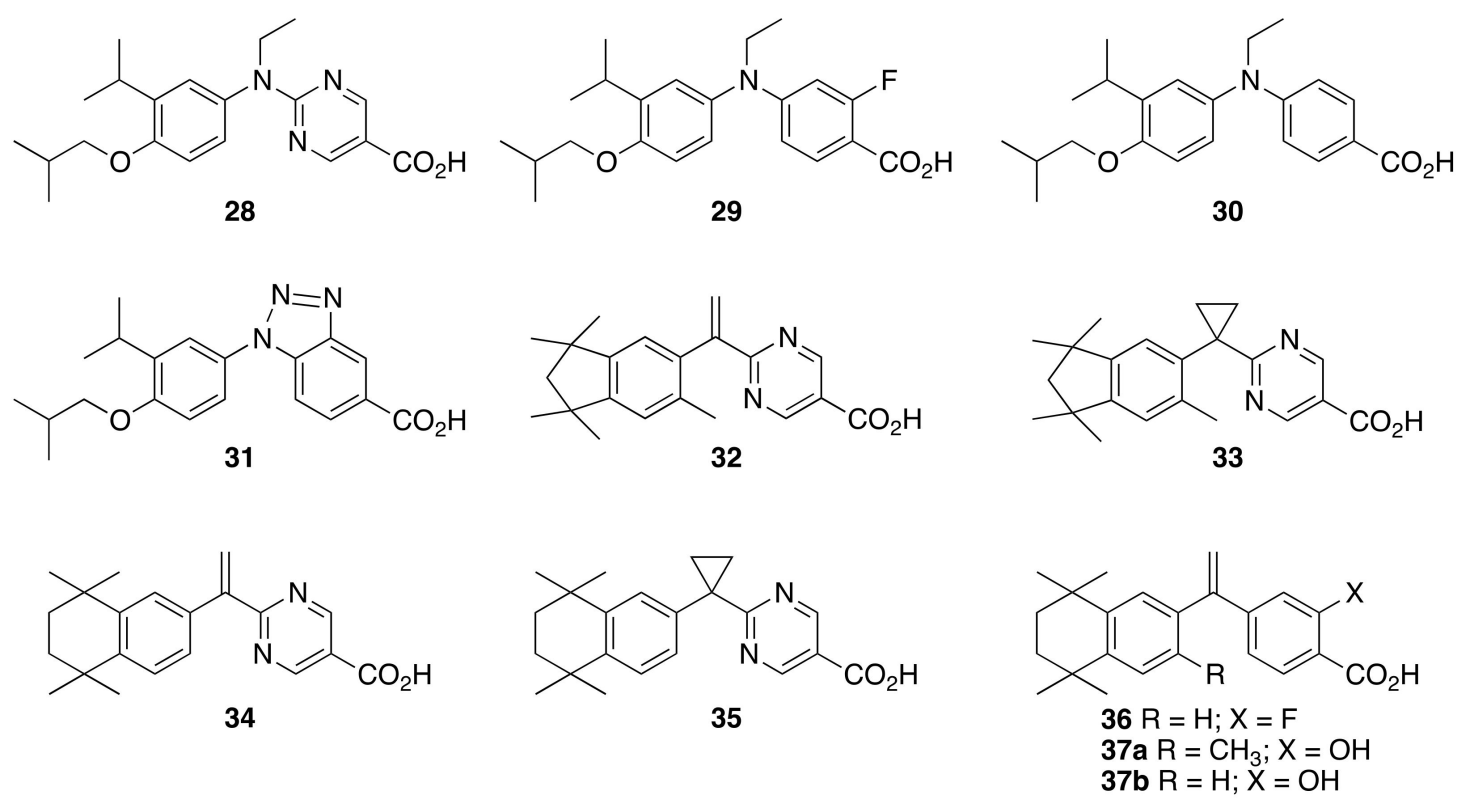
Structures of novel target rexinoids **28**–**36**, **37a**, and **37b**.

The current work concerns the synthesis of four novel analogs of NEt-4IB, compounds **28**–**31**, and seven novel analogs of bexarotene, compounds **32**–**37a** and **37b**, for preliminary biological evaluation in KMT2A-MLLT3 leukemia cells as well as a number of receptor-based assays in human cell lines to probe off-target activity (Figure 3).

## 2. Results: Molecular Modeling

**Figure 4:**
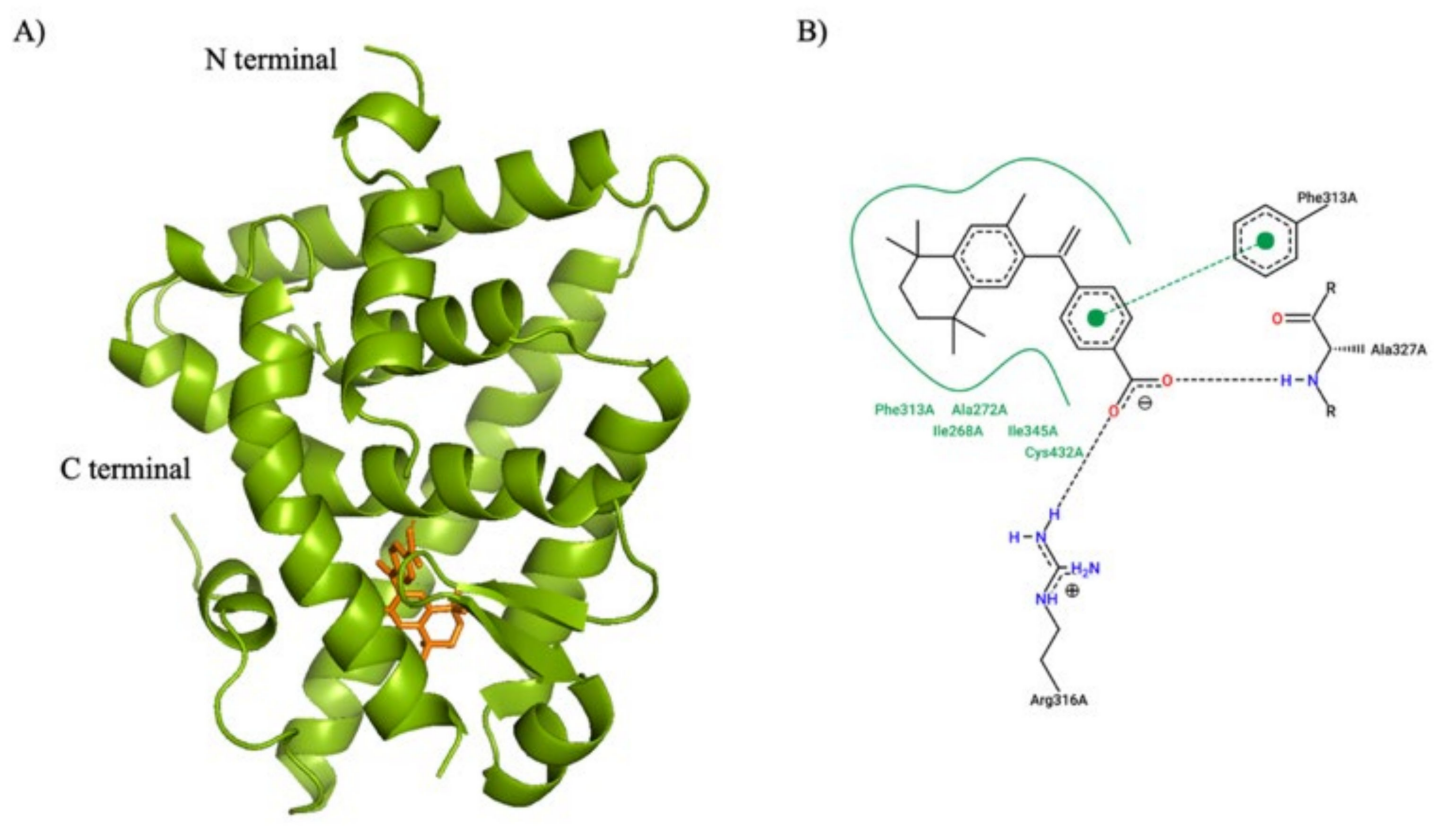
Illustration of AutoDock Vina simulation for RXR binding with bexarotene. (**A**) Cartoon representation of the human RXR alpha ligand binding domain (PDB:1FBY) in green and the compound bexarotene in orange. N and C terminals are labeled. (**B**) 2-dimentional depiction of the interactions between protein residue sidechains with bexarotene using PoseView (BioSolvIT). Hydrogen bonds are presented as dashed lines between interaction partners, and hydrophobic interactions are depicted as smooth contour lines.

AutoDock Vina was used to predict noncovent binding of human-RXR with different compounds. The output of AutoDock Vina is the prediction of bound conformations and a score represents binding affinity. The predicted binding affinity of human-RXR for each ligand is output as an energy unit in kcal/mol (Table 1), followed by visual inspection of the bound ligand–protein complexes in PyMOL (version 2.3, Schrödinger, LLC) (Figure 4a). To further analyze and illustrate the interactions between protein residue sidechains with the ligands, PoseView (BioSolvIT [78,79], Sankt Augustin, Germany) was used to generate the two-dimensional renderings depicting RXR protein sidechain interactions with each ligand (Figure 4b). In these two-dimensional depictions, hydrogen bonds are presented as dashed lines between interaction partners, and hydrophobic interactions are depicted as smooth contour lines. In the predicted conformation, the binding pocket of bexarotene involved five key residues: Ile268, Ala272, Phe313, Ile345, and Cys432. This detailed structural information from docking provides insights to design or modify compounds with higher affinity and selectivity in the future.

The AutoDock Vina score showed that the standard compound bexarotene (**1**), with a score of −12.7 kcal/mol, was the most potent among all compounds. Compounds **26**, **27**, **33**, **35**, **36**, **37a**, and **37b** had comparable scores of −12.3 kcal/mol, −12.0 kcal/mol, −11.6 kcal/mol, −11.5 kcal/mol, −12.4 kcal/mol, −12.1 kcal/mol, and −11.9 kcal/mol, respectively (Table 1). The lower AutoDock Vina scores for **33**, **35**, **36**, **37a**, and **37b** provided the motivation to synthesize these compounds for biological evaluation. Based on prior experience with modeling for these compounds, we were eager to synthesize all RXR compounds with a docking score within the range of 10% to that of bexarotene, since these compounds possessed the potential to be better candidates that exhibited comparable EC_50_ and IC_50_ profiles for further study.

## 3. Results: Chemistry

**Scheme 1:**
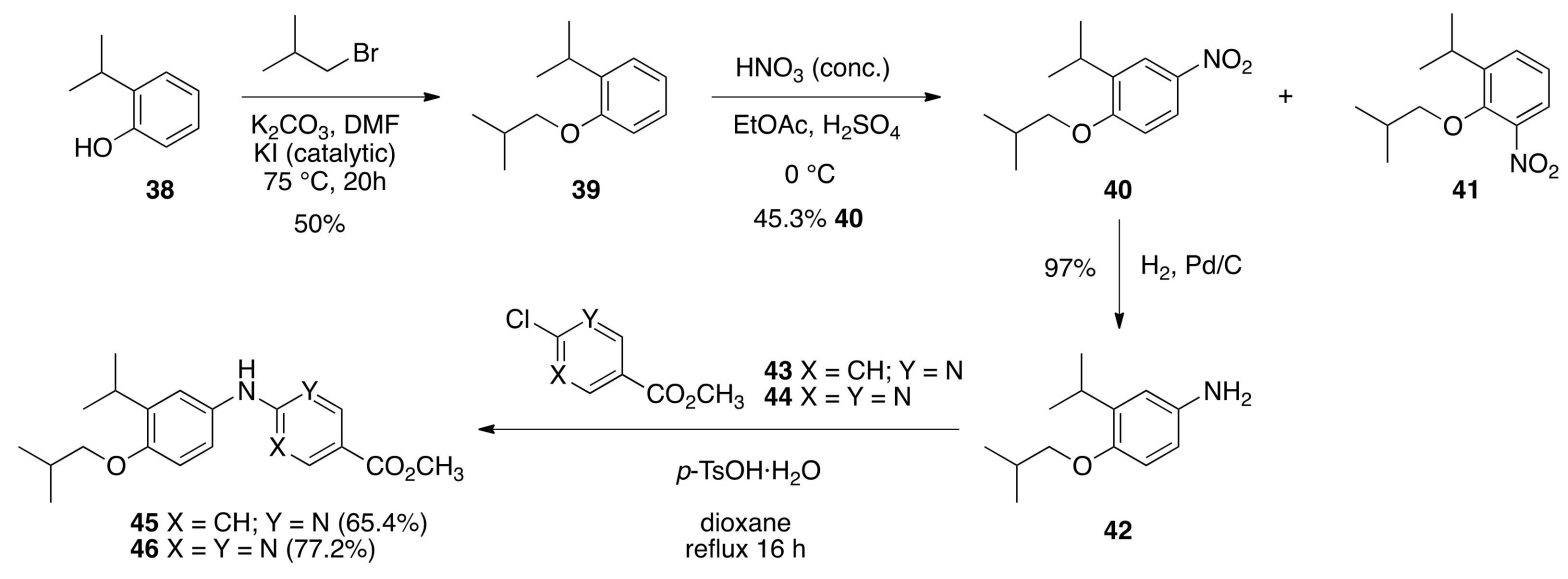
Synthesis of **45** and **46** from **38**.

The NEt-4IB analogs **28**–**31** were synthesized largely following the protocols described by Kakuta and co-workers. While the published synthetic route to **NEt-4IB** begins with the nitration of 2-isopropyl phenol in the presence of zinc(II) chloride under ultrasonication conditions, the route that was undertaken in the current study begins with the alkylation of 2-isopropyl phenol (**38**) to give 1-isobutoxy-2-isopropylbenzene (**39**) in 50% yield followed by nitration with concentrated (>90%) nitric acid and sulfuric acid in ethyl acetate at 0 °C to give a 3:1 mixture of mono-nitrated products **40** and **41**. The 1-isobutoxy-2-isopropyl-4-nitrobenzene (**40**) was separated by column chromatography and isolated in a 45.3% yield. The nitro-group of compound **40** was reduced with Pd/C to give 4-isobutoxy-3-isopropylaniline (**42**) in 97% yield. Following the method of Kakuta and co-workers for the synthesis of **NEt-4IB**, aniline **42** was combined with methyl 6-chloronicotinate (**43**) and para-toluene sulfonic acid in dioxane and the reaction was refluxed for 16 h to give methyl 6-((4-isobutoxy-3-isopropylphenyl)amino)nicotinate (**45**) in 65.4% yield. In a similar manner, aniline **42** was combined with methyl 2-chloropyrimidine-5-carboxylate (**44**) and para-toluene sulfonic acid in dioxane and the reaction was refluxed for 16 h to give methyl 2-((4-isobutoxy-3-isopropylphenyl)amino)pyrimidine-5-carboxylate (**46**) in a 77.2% yield (Scheme 1).

**Scheme 2:**
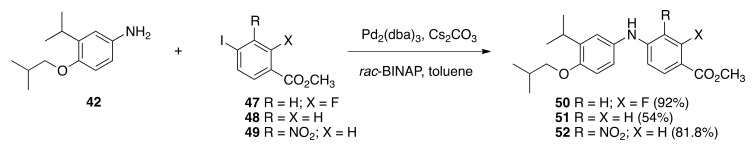
Synthesis of **50**, **51**, and **52** from **42**.

Using a different nitrogen-aryl bond forming reaction, aniline **42** was next coupled to methyl 2-fluoro-4-iodobenzoate (**47**) with tris(dibenzylideneacetone)-dipalladium in the presence of rac-BINAP and cesium carbonate to give methyl 2-fluoro-4-((4-isobutoxy-3-isopropylphenyl)amino)benzoate (**50**) in a 92% yield. Using a similar method, aniline **42** was coupled to methyl 4-iodobenzoate (**48**) with tris(dibenzylideneacetone)-dipalladium in the presence of rac-BINAP and cesium carbonate to give methyl 4-((4-isobutoxy-3-isopropylphenyl)amino)benzoate (**51**) in a 54% yield. Again, aniline **42** was coupled to methyl 4-iodo-3-nitrobenzoate (**49**) with tris(dibenzylideneacetone)-dipalladium in the presence of rac-BINAP and cesium carbonate to give methyl 4-((4-isobutoxy-3-isopropylphenyl)amino)-3-nitrobenzoate (**52**) in an 81.8% yield (Scheme 2).

**Scheme 3:**
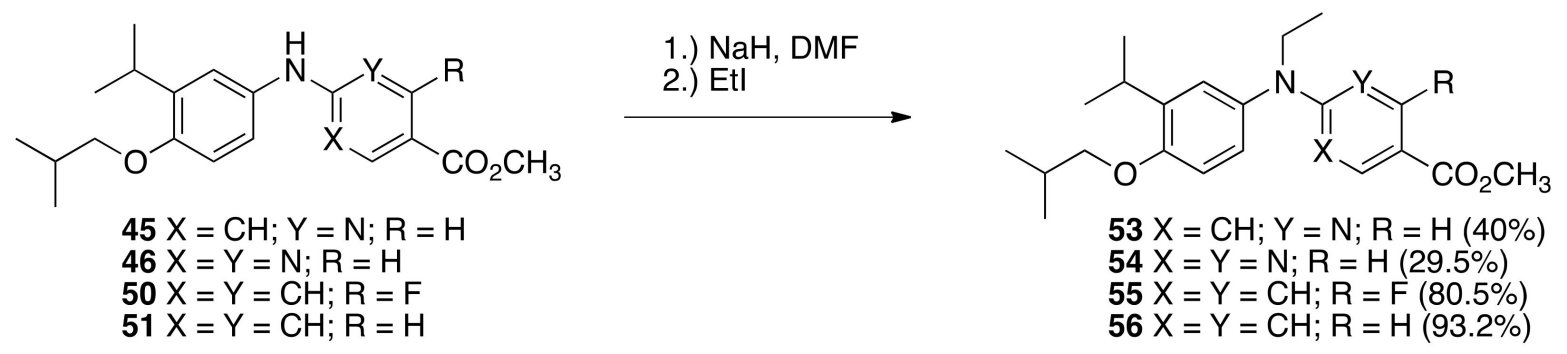
Synthesis of **53**, **54**, **55**, and **56** from **45**, **46**, **50**, and **51**, respectively.

Next, diarylamines **45**, **46**, **50**, and **51** were alkylated by treatment with sodium hydride in DMF followed by the addition of ethyl iodide to give ethyl-diarylamine methyl esters **53** (40%), **54** (29.5%), **55** (80.5%), and **56** (93.2%) (Scheme 3).

**Scheme 4:**
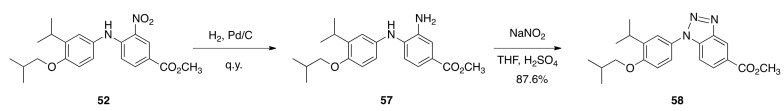
Synthesis of **58** from **52**.

Compound **52**, however, was reduced with Pd/C to give **57** in quantitative yield, and **57** was treated with sodium nitrite and sulfuric acid in THF to give methyl 1-(4-isobutoxy-3-isopropylphenyl)-1H-benzo[d][1-3]triazole-5-carboxylate (**58**) in an 87.6% yield (Scheme 4).

**Scheme 5:**
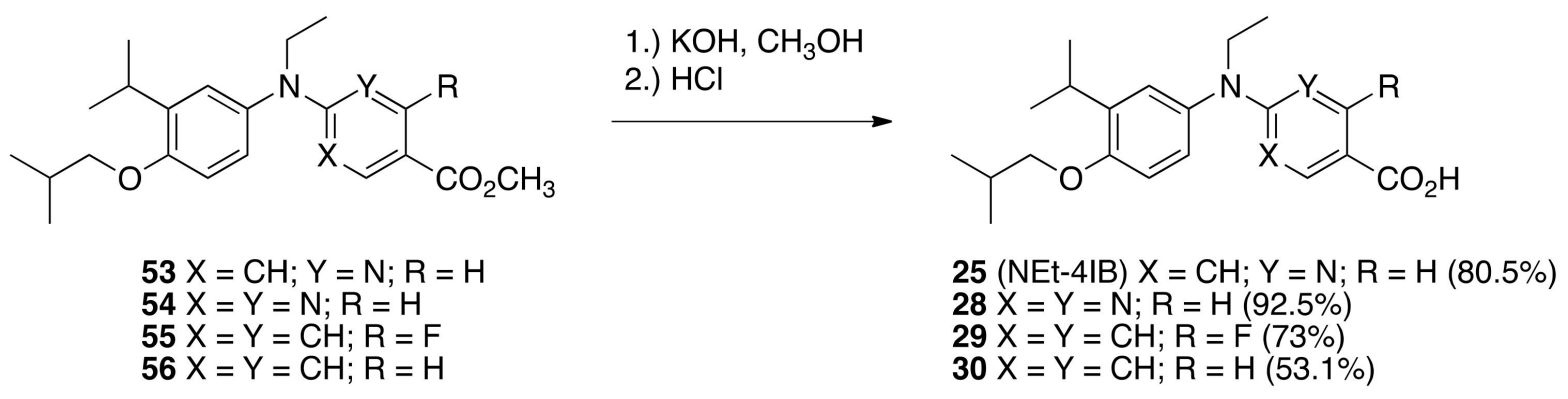
Synthesis of **25**, **28**, **29**, and **30** from **53**, **54**, **55**, and **56**, respectively.

Methyl esters **53**, **54**, **55**, and **56** were saponified in methanolic potassium hydroxide followed by acidification with hydrochloric acid to give **25** (NEt-4IB) (80.5%), **28** (92.5%), **29** (73%), and **30** (53.1%) after purification by column chromatography (Scheme 5).

**Scheme 6:**
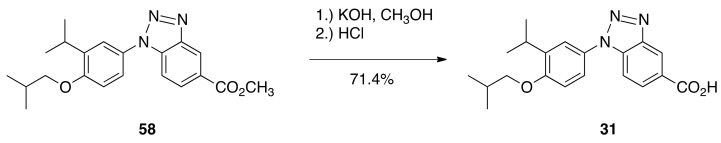
Synthesis of **31** from **58**.

Methyl ester **58** was also saponified in methanolic potassium hydroxide followed by acidification with hydrochloric acid to give 1-(4-isobutoxy-3-isopropylphenyl)-1H-benzo[d][1-3]triazole-5-carboxylic acid (**31**) in 71.4% (Scheme 6).

**Figure 5:**
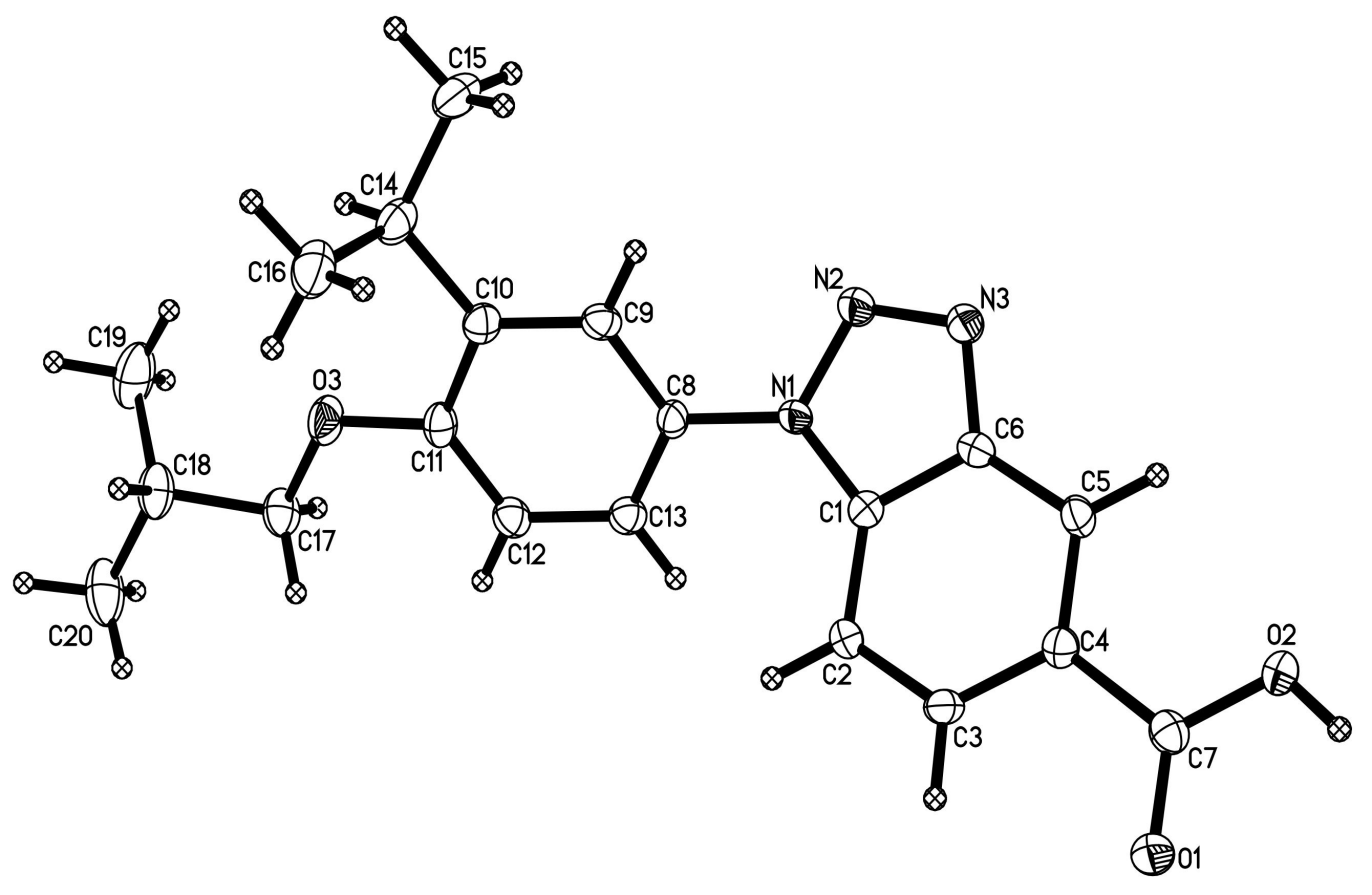
X-ray crystal structure of **31**.

Acid **31** formed transparent, single crystals suitable for X-ray diffraction, and a study confirmed the structure of **31** (Figure 5). The structural determination of **31** helped confirm the correct relative positioning of the iso-butoxy and isopropyl groups with respect to the linking nitrogen atom on the aromatic ring of **31** and our other analogs of NEt-4IB.

**Scheme 7:**
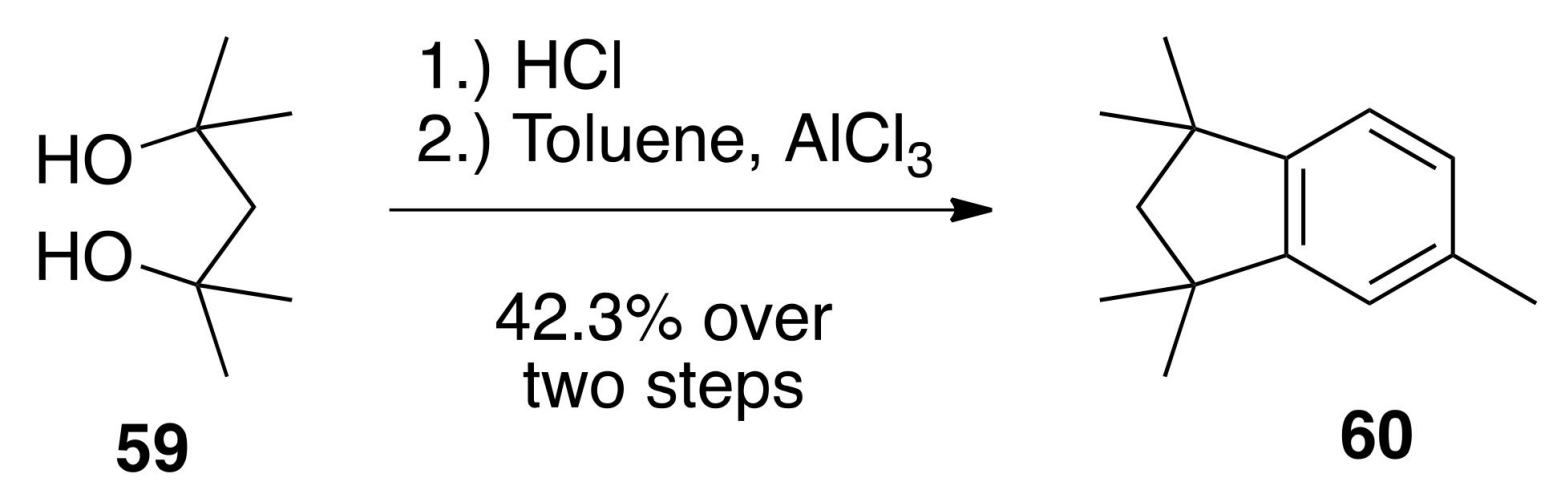
Synthesis of **60** from **59**.

The synthesis of target novel rexinoids **26**, **32**, and **33** begins with the conversion of 2,4-dimethyl-2,4-pentanediol (**59**) to 1,1,3,3,5-pentamethyl-2,3-dihydro-1H-indene (**60**). This was facilitated through the treatment of commercially available **59** with concentrated hydrochloric acid, providing a 46% crude yield of 2,4,-dichloro-2,4-dimethylpentane, which was then subjected to catalytic AlCl_3_ and toluene to produce the indane **60** in a 42% yield over two steps (Scheme 7).

**Scheme 8:**
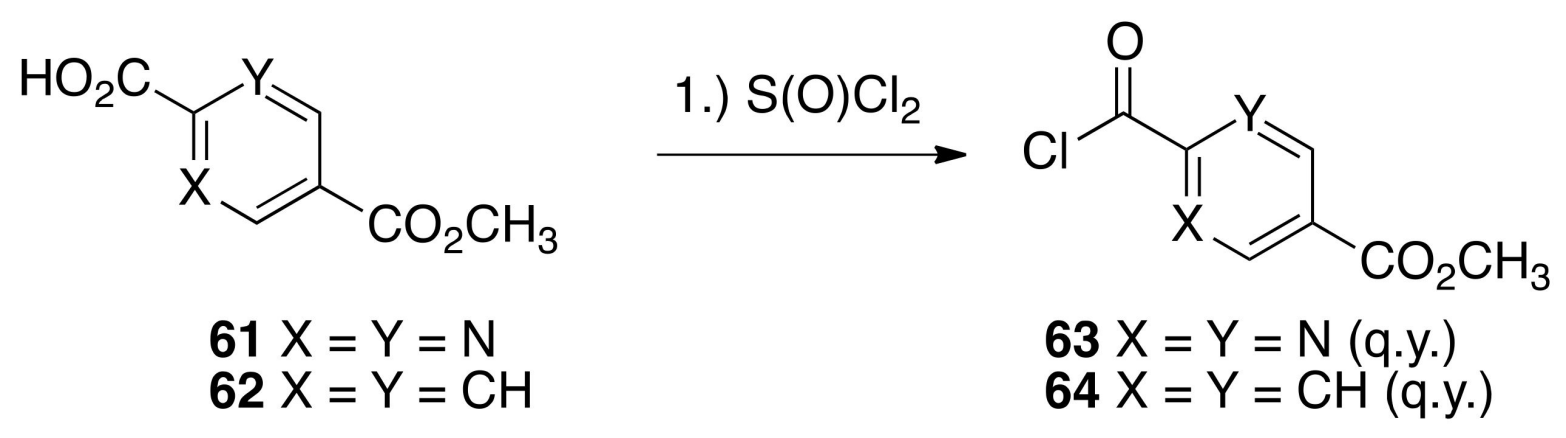
Synthesis of **63** and **64** from **61** and **62**, respectively.

Known compound **61 [21]** and commercially available compound **62** were converted by reflux in thionyl chloride to acid chlorides **63** and **64** in quantitative crude yield after removal of excess thionyl chloride (Scheme 8).

**Scheme 9:**
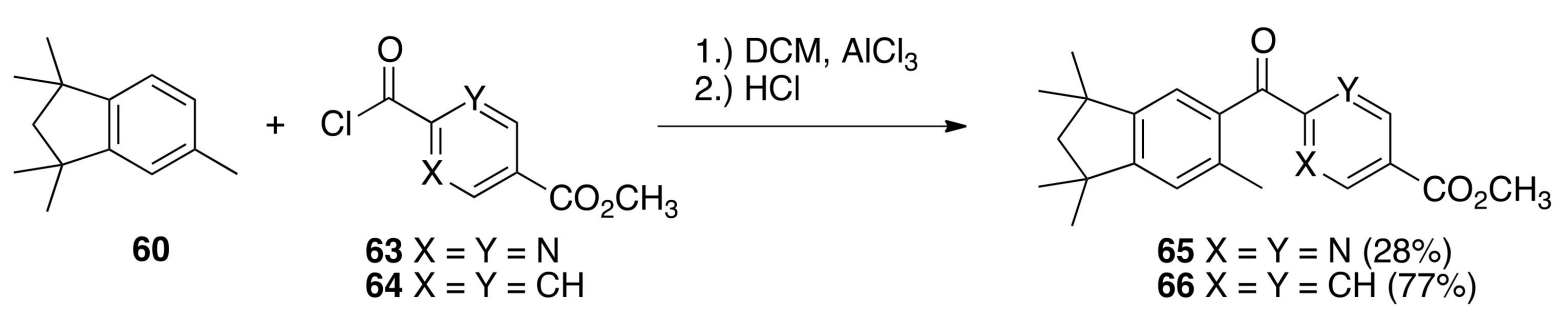
Synthesis of **65** and **66** from **63** and **64**, respectively.

Acid chlorides **63** and **64** gave carbonyl compounds **65** and **66** by Friedel–Crafts acylation with indane **60** (Scheme 9).

**Scheme 10:**
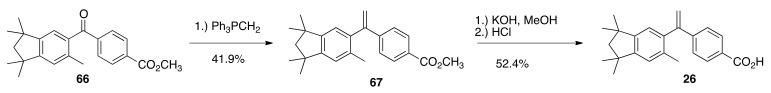
Synthesis of **26** from **66**.

Ketone **66** was treated with a solution of triphenylphosphonium methylide to give alkene **67**, in a 41.9% yield, and methyl ester **67** was saponified with potassium hydroxide in boiling methanol and precipitated with hydrochloric acid to give rexinoid **26** in a 52.4% yield after purification (Scheme 10).

**Scheme 11:**
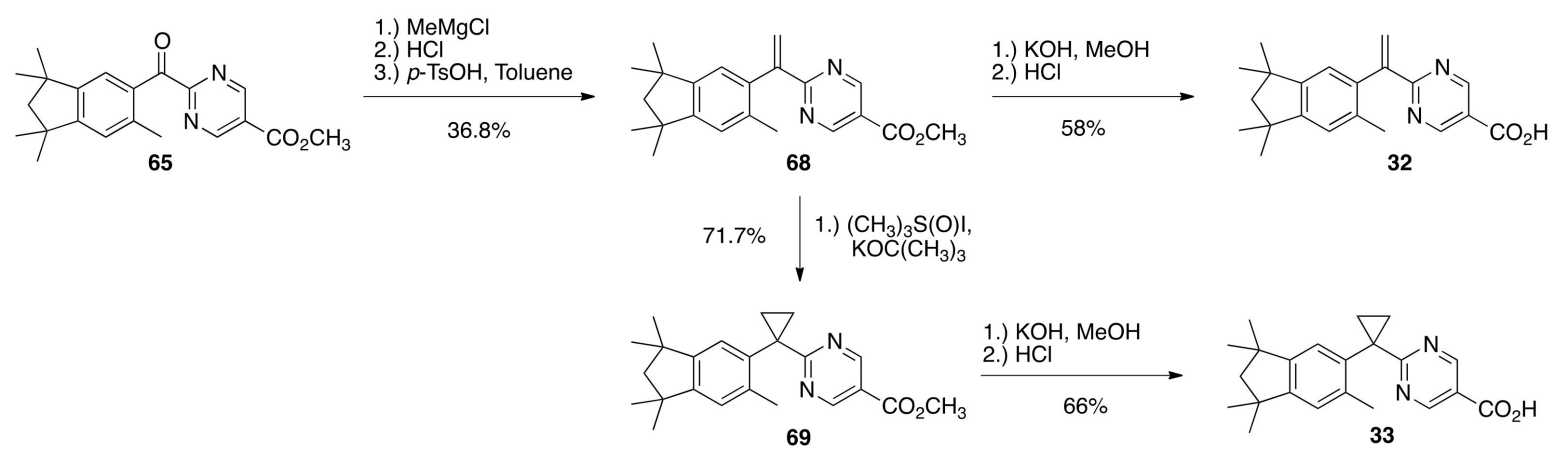
Synthesis of **32** and **33** from **65**.

Ketone **65** was treated with methyl magnesium chloride and quenched with hydrochloric acid, followed by reflux using para-toluene sulfonic acid monohydrate and toluene in a three-step synthesis of alkene **68**, giving an overall yield of 36.8%, and alkene **68** was saponified to give rexinoid **32** in a 58% yield. Alkene **68** was also subjected to cyclopropanation by treatment with a solution of dimethylsulfoxonium methylide to give methyl ester **69** in 71.7% yield, which was subsequently saponified to provide rexinoid **33** in 66% yield (Scheme 11).

**Figure 6:**
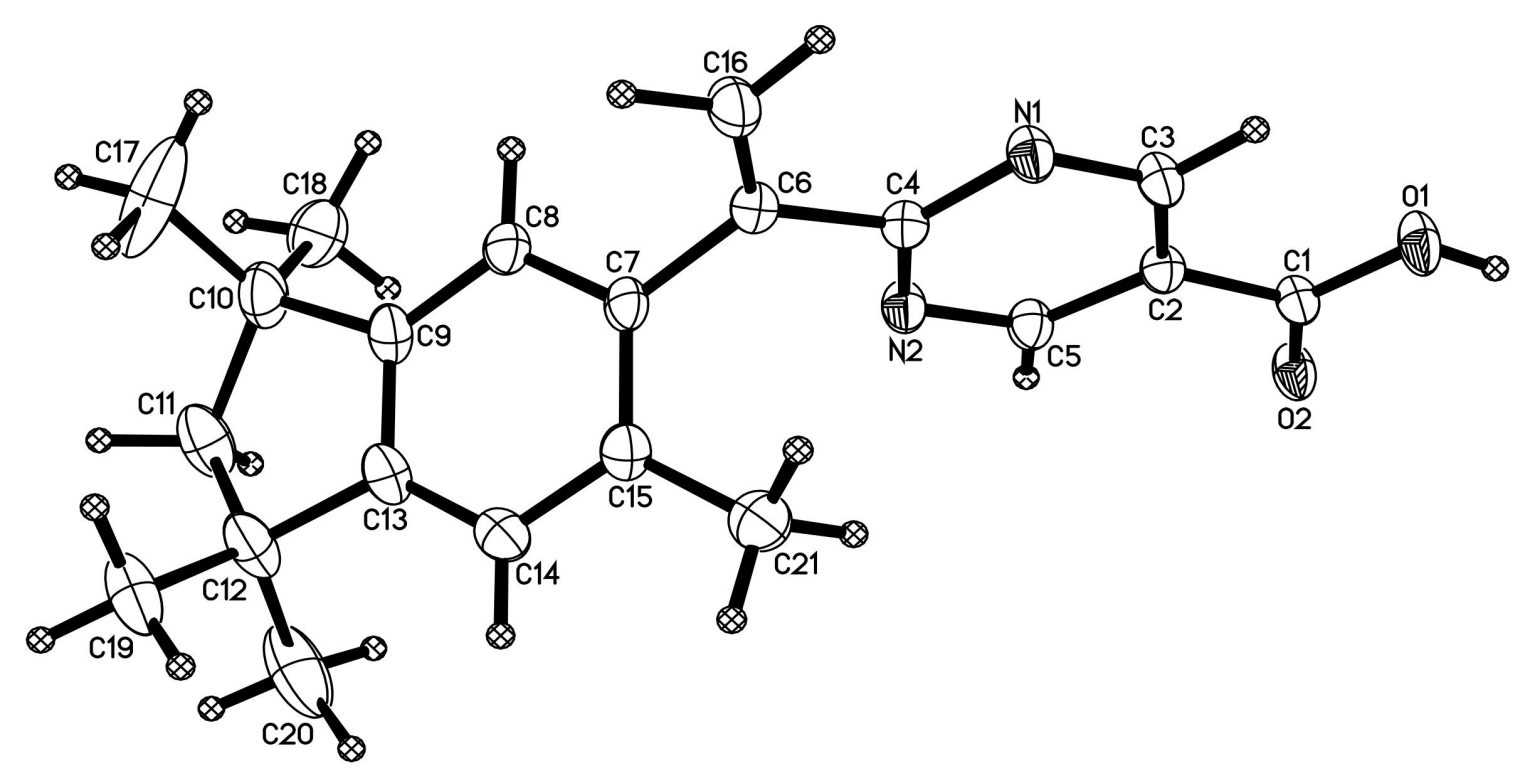
X-ray crystal structure of **32**.

Acid **32** formed transparent, single crystals suitable for X-ray diffraction, and a study confirmed the structure of **32** (Figure 6). The structural determination of **32** helps confirm the correct structure assignment of the indanyl-ring system of **32** and the other analogs in the current work (**26** and **33**) possessing the same ring system.

**Scheme 12:**
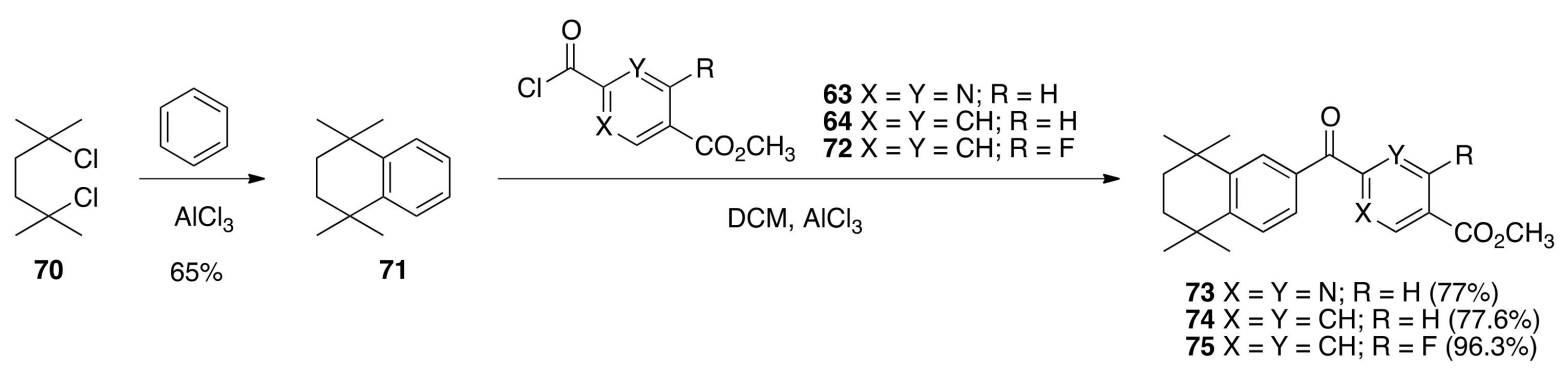
Synthesis of **73**, **74**, and **75** from **63**, **64**, and **72** respectively.

The synthesis of **27** and **34**–**36** begins with the Friedel–Crafts alkylation of benzene by 2,5-dichloro-2,5-dimethylhexane (**70**) catalyzed by aluminum chloride to give 1,1,4,4-tetramethyl-1,2,3,4-tetrahydronaphthalene (**71**) in a 65% yield by distillation, according to the method of Bruson and Kroger [80]. The Friedel–Crafts acylation of **71** was then performed with acid chlorides **63**, **64**, and **72** to give ketones **73**, **74**, and **75**, respectively, in good yields (77–96%) (Scheme 12).

**Scheme 13:**
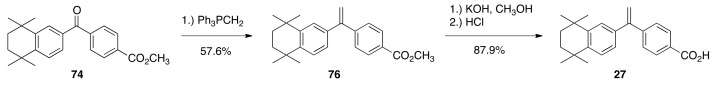
Synthesis of **27** from **74**.

To produce rexinoid **27**, the method of Boehm and co-workers was followed. Ketone **74** was treated with a solution of triphenylphosphonium methylide to give methyl ester **76** (57.6%), and then **76** was saponified to give rexinoid **27** (87.9%) (Scheme 13).

**Scheme 14:**
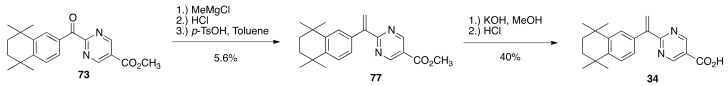
Synthesis of **34** from **73**.

Ketone **73** was treated with methylmagnesium chloride, followed by aqueous acidic workup, extraction and then reflux in toluene with p-TsOH monohydrate to give alkene **77** in a 5.6% yield—alternative Wittig reaction conditions improved yields only modestly—and **77** was then saponified to give rexinoid **34** in an 87% yield (Scheme 14).

**Scheme 15:**
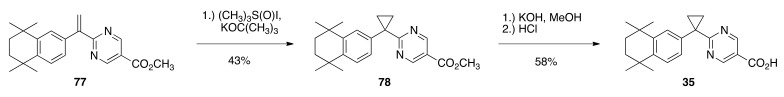
Synthesis of **35** from **77**.

Alkene **77** was also subjected to cyclopropanation by treatment with a solution of dimethylsulfoxonium methylide to give methyl ester **78** in a 43% yield, which was subsequently saponified to provide rexinoid **35** in a 58% yield (Scheme 15).

**Scheme 16:**
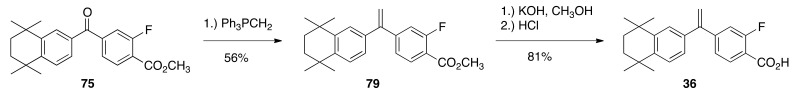
Synthesis of **36** from **75**.

Ketone **75** was treated with a solution of triphenylphosphonium methylide in THF and converted to alkene **79** (56%), which was then saponified to give rexinoid **36** in an 81% yield (Scheme 16).

**Scheme 17:**
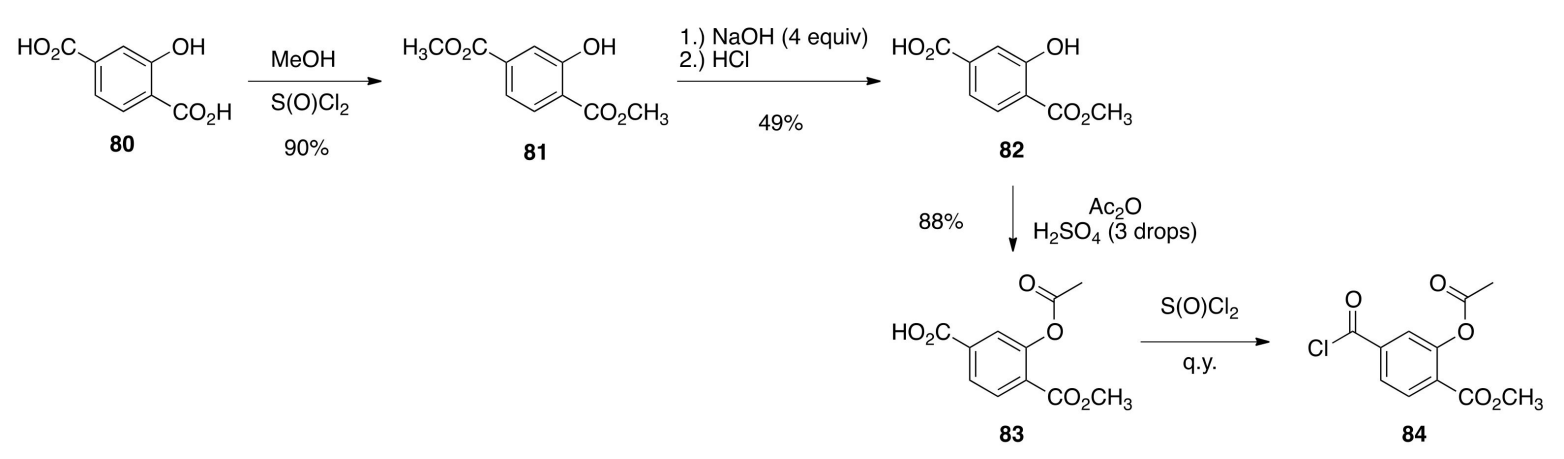
Synthesis of **84** from **80**.

Finally, for the synthesis of hydroxylated analogs of bexarotene (**37a** and **37b**), the commercially available 2-hydroxyterephthalic acid (**80**) was esterified by reflux in acidic methanol to give dimethyl 2-hyroxyterephthalate (**81**) in 90%, and using the method of Ningren and co-workers [81], **81** was selectively saponified to **82** in a 49% yield. The hydroxyl group of **82** was then acetylated to give **83** in an 88% yield, and **83** was converted to acid chloride **84** quantitatively by treatment with thionyl chloride (Scheme 17).

**Scheme 18:**
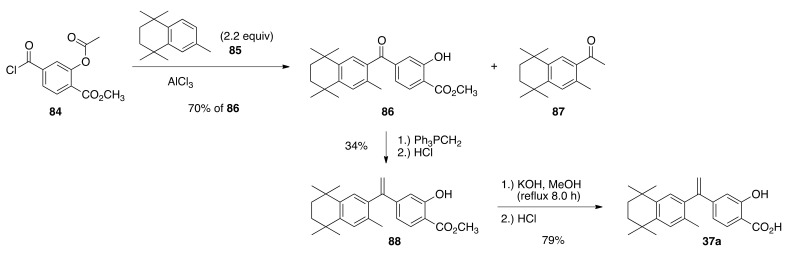
Synthesis of **37a** from **84**.

Since the acetyl protecting group was discovered to be labile under typical Friedel–Crafts acylation conditions, 2.2 equivalents of known compound **85** [58] were combined with **84** and aluminum chloride to give a 70% yield of ketone **86** after aqueous acidic workup and an un-recovered mass of **87** as the by-product of the labile acetyl protecting group. Finally, ketone **86** was treated with an excess of triphenylphosphonium methylide solution to give alkene **88** in a 34% yield after acidic workup, and **88** was saponified to **37** in a 79% yield (Scheme 18).

**Scheme 19:**
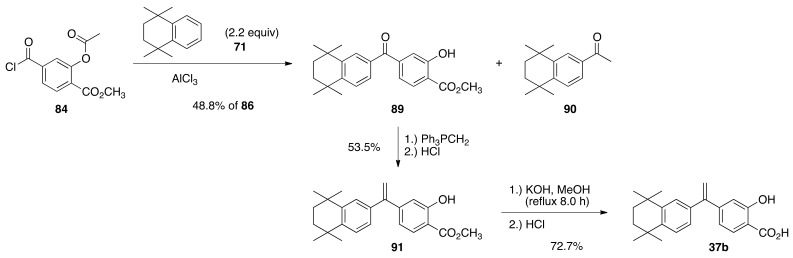
Synthesis of **37b** from **84**.

In a similar manner, acid chloride **84** was combined with **71** (2.2 equivalents) in dichloromethane with aluminum chloride to give ketone **89** in a 48.8% yield and an unrecovered mass of **90** after aqueous acidic workup. Ketone **89** was treated with a solution of triphenylphophine methylide followed by aqueous acidic workup to give **91** in a 53.5% yield, and compound **91** was saponified to give compound **37b** in a 72.7% yield after purification by silica gel column chromatography (Scheme 19).

## 4. Results and Discussion: Biological Assays

**Figure 7:**
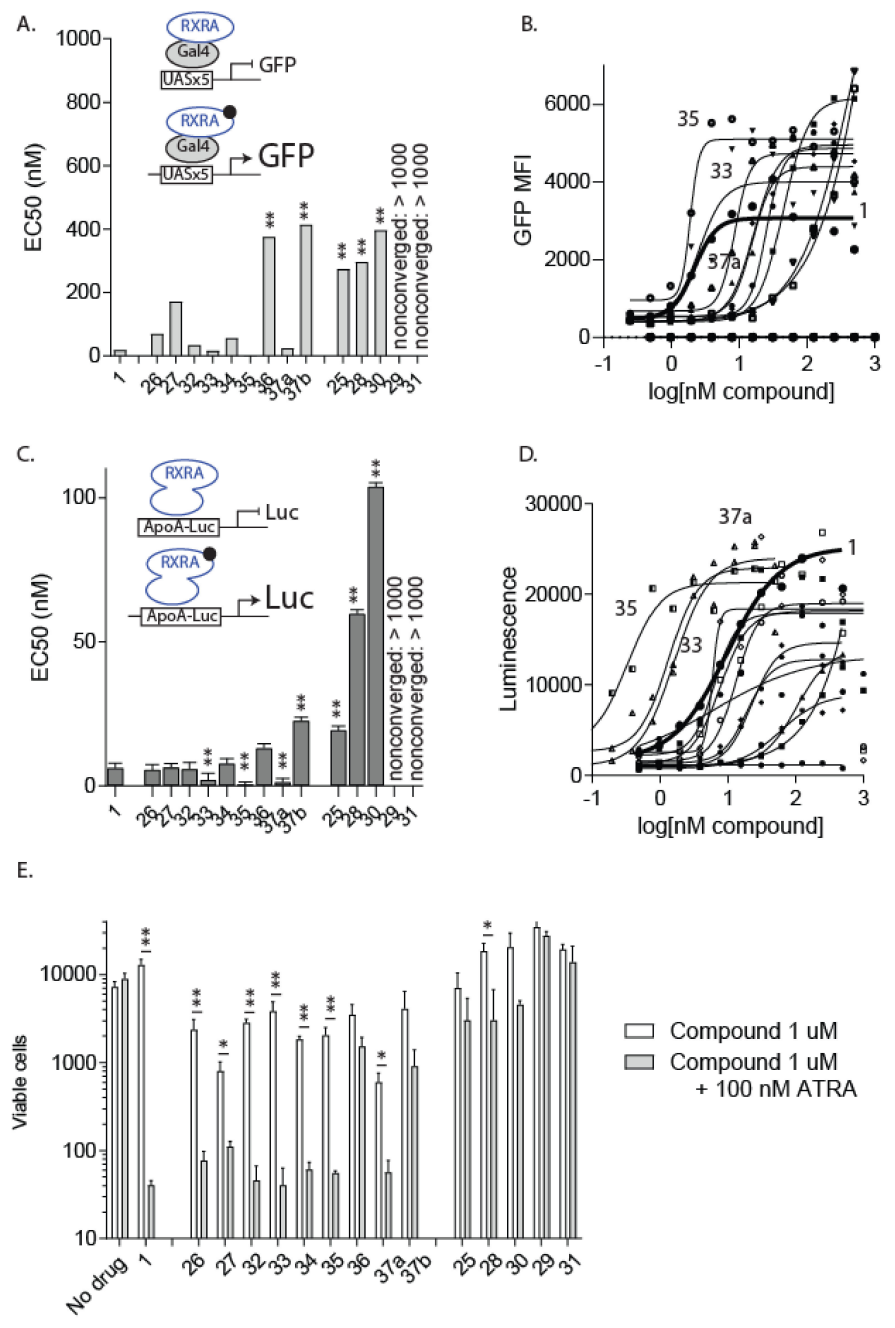
Effect of pharmacological targeting of RXRA on KMT2A-MLLT3 proliferation in vitro. (**A**) EC_50_ (nM) values for each compound were calculated based on the ratio of GFP+ mCherry+ cells to total mCherry+ cells in UAS-GFP × KMT2A-MLLT3 cells transduced with Gal4-RXRA retrovirus and treated as indicated. ** *p* < 0.01 compared with 1 (bexarotene) result. (**B**) GFP activation used to calculate EC_50_ in (**A**), each dose evaluated in duplicate. (**C**) EC_50_ (nM) values for each compound were calculated based on the luciferase relative intensity of 293T cells transduced with pBABE-RXRA and ApoA1-Luc and treated as indicated. * *p* < 0.05, ** *p* < 0.01 compared with 1 (bexarotene) result. (**D**) Luciferase luminescence results used to calculate EC_50_ in (**C**), each dose evaluated in duplicate. (**E**) UAS-GFP x KMT2A-MLLT3 cells were treated as indicated, replated after 48 h, and total viable cells in 50 µL assessed in duplicate after 96 total hours of treatment. ** *p* < 0.01 comparing results with and without ATRA (all *trans* retinoic acid) treatment. Pairwise T-test.

Bexarotene (**1**) and analogs **25**—**36**, **37a**, and **37b** were assessed in KMT2A-MLLT3 cells to obtain EC_50_ values for RXRα activation in both a GFP and Luc-assay, and then also in a 96 h cell viability assay both with and without 100 nM ATRA (Figure 7), the results of which are summarized in Table 1.

These compounds were also assessed for mutagenicity and toxicity in Saccharomyces cerevisiae and the toxicity results are summarized in Table 1. No compound was mutagenic in this assay.

The EC_50_ determination showed that the standard bexarotene (**1**) with an EC_50_ value of 18 nM, was one of the most potent rexinoids for the assay, where only compounds **33** and **35** possessed lower EC_50_ value concentrations of 17 nM and 1.3 nM, respectively, and the hydroxy-bexarotene analog **37a** possessed a comparable EC_50_ value of 24.2 nM (Table 1). Not surprisingly, only **33**, **35**, and **37a** exhibited comparable IC_50_ values to bexarotene (**1**) in the 96 h cell viability assay where compounds were tested in the presence of 100 nM ATRA (Table 1). Of these compounds, only **37a** demonstrated cytotoxicity in the Saccharomyces cerevisiae assay of 1 µg/µL (Table 1). In terms of SAR, the most active compounds, **33** and **35**, possessed a cyclopropyl-linking ring and the pyrimidyl-carboxylic acid system. It is curious that changing the pentamethyl-napthalenyl ring system of bexarotene to either the pentamethyl-indanyl or tetramethyl-napthalenyl system of **33** and **35**, in combination with substituting the vinyl-linking group with a cyclopropyl ring system and the benzene ring with a pyrimidine ring system, did not reduce activity for the receptor or antiproliferative effects in cell culture. Changing the pentamethyl-napthalenyl to a tetramethyl-napthalenyl group without changing other groups appears to lower the activity at the receptor—**27** versus **1**, **37b** versus **37a**, **36** versus **6**, and **34** versus **14**. The NEt-4IB (**25**) and related analogs **28**–**31** possessed higher EC_50_ values than bexarotene, ranging from 274 nM to >1000 nM, and all of the IC_50_ values for the 96 h cell viability assays of **25** and **28**–**31** in the presence of 100 nM ATRA were >1000 nM. Finally, there appeared to be a fairly good correlation between the EC_50_ values and relative IC_50_ values in the 96 h cell viability assay of the analog plus 100 nM ATRA for analogs **26**, **27**, **34**, **36**, and **37b**.

**Figure 8:**
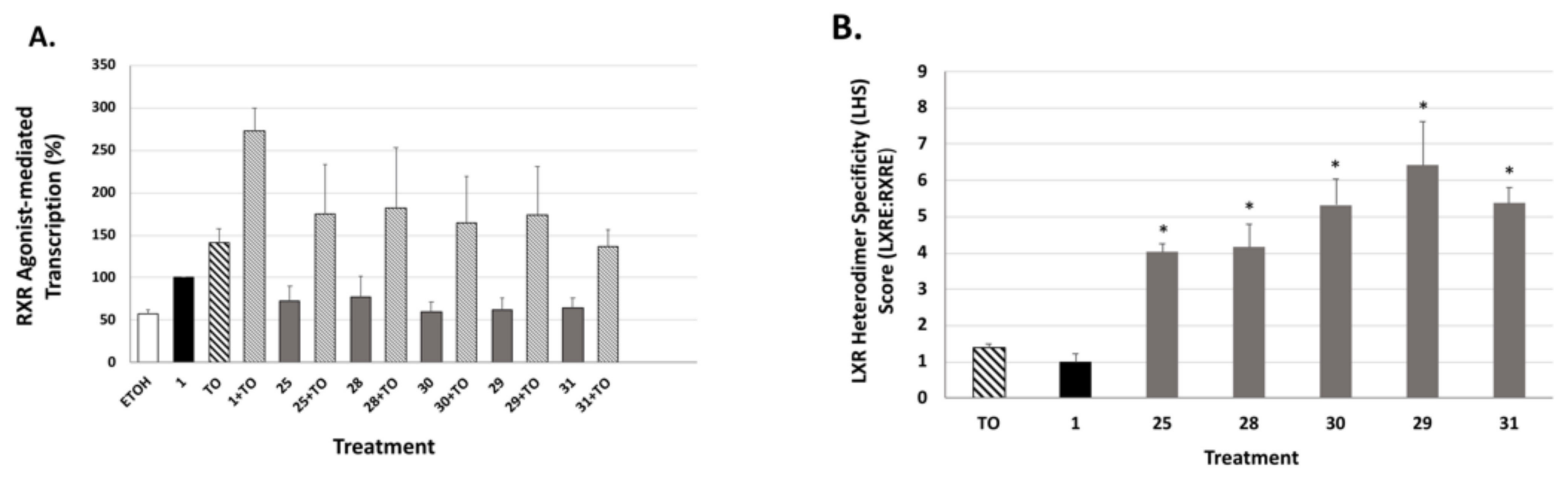
Evaluation of RXR agonists to potentiate LXRE-mediated transactivation in the absence and presence of LXR ligand T0901317. (**A**) HEK-293 human embryonic cells were transfected with pCMX-hLXRα, an expression vector for human LXRα, an LXRE-luciferase reporter gene with three tandem copies of the LXRE from the human ApoE gene, and a renilla control plasmid. Cells were transfected for 24 h utilizing a liposome-mediated transfection protocol and then treated with the ethanol vehicle, or 100 nM of the indicated compound alone or in combination with 100 nM TO901317 (TO). LXRE-directed activity was compared to compound 1 (bexarotene), set to 100%. (**B**) The “Heterodimer Specificity Score” (LHS) was determined by calculating the LXRE:RXRE activity ratio of each analog, with compound 1 set to 1.0. Values are means ± SD with indicated analogs exhibiting greater LHS vs. compound 1 (* *p* < 0.05).

**Figure 9:**
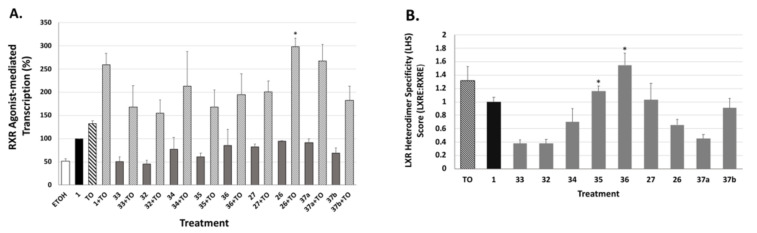
Evaluation of additional RXR agonists to potentiate LXRE-mediated transactivation in the absence and presence of LXR ligand T0901317. (**A**) Human HEK-293 cells were transfected and treated as described in Figure 8. LXRE-directed activity was compared to compound 1 (bexarotene), set to 100%. Values are means ± SD with indicated analog+TO exhibiting greater activity vs. compound 1+TO (* *p*, 0.05). (**B**) The “Heterodimer Specificity Score” (LHS) was determined by the LXRE:RXRE ratio with compound 1 set to 1.0. Values are means ± SD with indicated analogs exhibiting greater LHS vs. compound 1 (* *p* < 0.05).

We next tested the analogs for their ability to bind and activate the liver-X-receptor (LXR) using a liver-X-receptor responsive element (LXRE)-based assay, and we compared the effect in the presence vs. absence of an activating LXR compound (TO901317). LXR has been demonstrated to regulate lipid metabolism and inflammatory responses in the central nervous system, and there is ample evidence that robust cholesterol and lipid metabolism in the brain (including enhanced ApoE expression) are critical to mitigating dementia. Biological evaluation of our novel RXR agonists for their ability to transactivate via an LXRE sequence that is found naturally in the promoter of LXR-RXR controlled genes including ApoE was carried out in human embryonic cells (HEK293) with bexarotene (**1**) as a comparison. The activation from this natural LXRE in our system was tested in the presence of either 100 nM RXR agonists alone or in combination with 100 nM of both the RXR agonist and LXR agonist T0901317 (TO). The use of the combination of LXR and RXR agonists was expected to display a more robust response in LXRE transactivation due to additive or synergistic effects of dual ligand activation of the RXR-LXR heterodimer. The results (Figure 8A and Figure 9A) revealed that in comparison to the parent bexarotene (**1**) compound alone, single dosing of the cells with any of the tested analogs displayed less LXR/LXRE activity. Specifically, the analogs possessed activities ranging from 46 to 94.5% of the bexarotene control (set to 100%; Table 1). Moreover, when a LXR synthetic ligand (TO) was used in combination with Bex or analogs, a similar profile was observed, with the exception of TO+**26**, which displayed a higher activity than TO+**1** (Figure 8A and Figure 9A).

While most of the analogs possessed slightly lower LXR activation when compared to bexarotene (**1**), it is important to consider this activity in the context of the RXR-RXR homodimer activity of each analog, and to thus “normalize” the LXR/LXRE heterodimer activation in order to yield a LXRE Heterodimer Specificity (LHS) score (Figure 8B and Figure 9B). The results of this LHS analysis (Table 1) revealed that many of our novel compounds (e.g., **25, 28, 29, 30, 31, 35**, and **36**) possessed greater LXR/LXRE activity via increased heterodimer specificity than the parent bexarotene (**1**).

**Figure 10:**
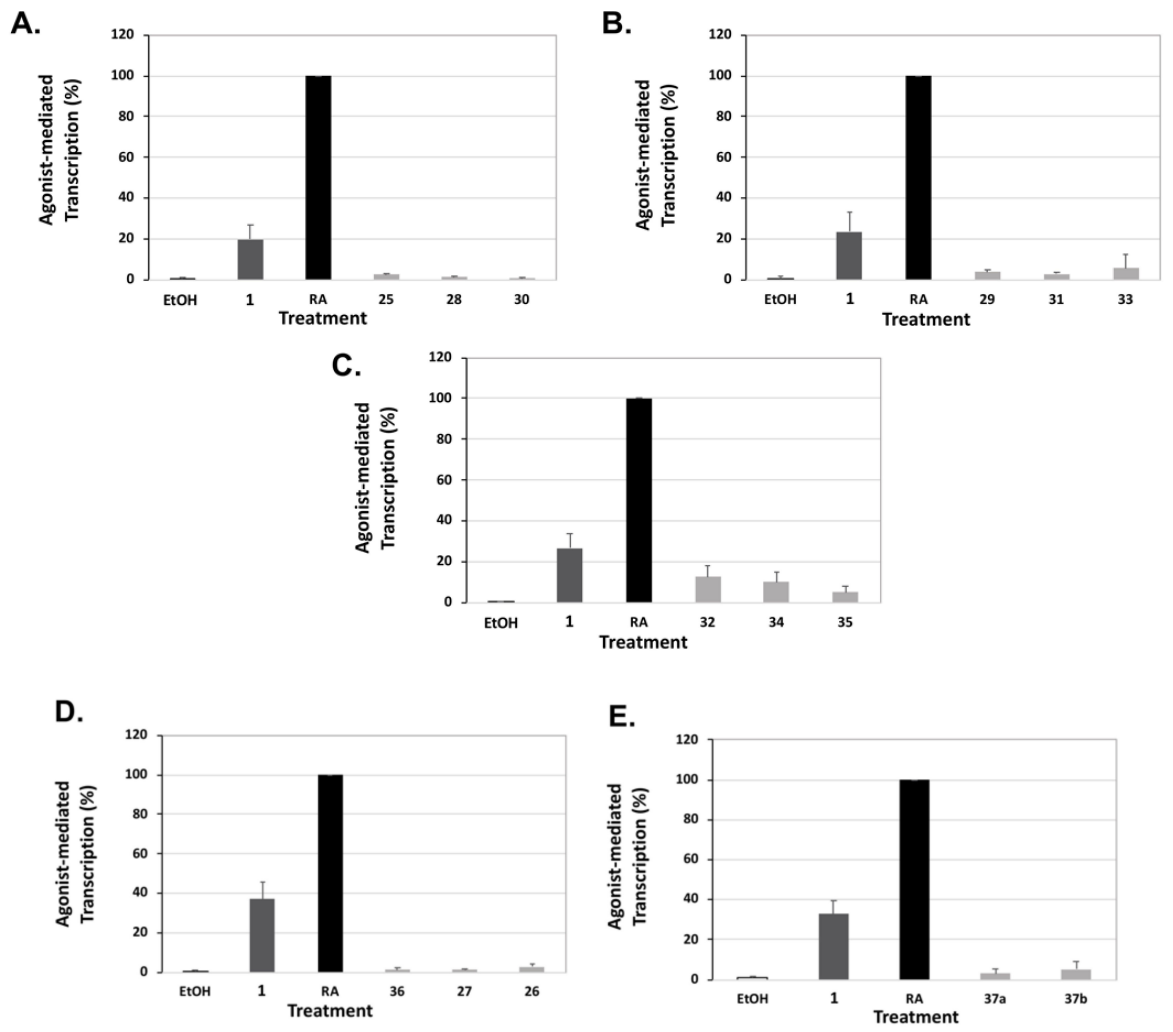
Assessment of RXR agonists via a RARE-luciferase reporter based assay in human cells. (**A**–**E**) Human embryonic cells (HEK293) were co-transfected with expression vectors for hRXRα, a RARE-luciferase reporter gene, and a renilla control plasmid for 24 h utilizing a liposome-mediated transfection protocol. Cells were treated with bexarotene, analog, or all-trans-retinoic acid (RA) at 10 nM for 24 h. The RARE activity for RA was set to 100%. Values are means ± SD with all analogs tested displaying lowered RARE activity vs. compound **1** (*p* < 0.05).

Finally, since compound **1** is known to possess “residual” RARE activity, we evaluated the ability of our compounds to induce transcription via the retinoic acid response element and retinoic acid receptor (RAR). Human embryonic cells (HEK293) were transfected with human RARα and dosed with 10 nM of either all-trans retinoic acid (RA), the natural ligand for RARα, compound **1**, or analogs. Employing this assay, compound **1** possessed and average 28.1% of the activity of the RA control (Figure 10). Compound **32** displayed the greatest RARE activation at 12.9% of RA, while compound **30** showed the lowest RARE activity at 1.1%, which is indistinguishable from the ethanol control (Table 1). Thus, all of our novel analogs displayed significantly less “cross-over” onto RAR-RARE signaling compared to bexarotene (**1**).

## 5. Conclusions

This work generated a number of NEt-4IB and bexarotene analogs to evaluate as RXR agonists and their ability to prohibit cell viability in a KMT2A-MLLT3 cell line in combination with 100 nM ATRA. In general, the EC_50_ values determined for the analogs correlated well with their IC_50_ values in the cell viability assay, and we identified two bexarotene analogs—**33** and **35**—more potent than bexarotene as well as one analog, **37a**, with comparable potency. Many of the analogs also revealed an enhanced ability to activate LXRE-mediated transcription, intimating their ability to significantly stimulate LXR/LXRE target genes such as ApoE, which have been implicated in protection against dementias. Furthermore, our novel panel of analogs possessed less “cross-over” activation than other retinoid pathways including RARE-directed transcription. Taken together, these results provide compelling motivation to continue to modify bexarotene and other reported RXR agonists to evaluate their potential anti-proliferative and other therapeutic activities.

## 6. Materials and Methods

### 6.1. Reagents and Constructs

Bexarotene was from LC Laboratories. ATRA was from Sigma Aldrich. The ApoA1-Luciferase and pBABE-RXRA plasmids were a gift from Vivek Arora, Washington University. MSCV–Gal4 DBD–RXRA LBD–IRES–mCherry (Gal4-RXRA) has been previously described [82].

### 6.2. Molecular Modeling

The three-dimensional structures of the compounds reported herein were generated using ChemDraw 3D (PerkinElmer Informatics), energy minimized, and exported in the Protein Data Bank (PDB) format. The human RXR alpha ligand binding domain structure model was obtained from the PDB (PDB code: 1FBY, [83]). The crystallized ligand, 9-cis retinoic acid, was removed from the protein model prior to docking simulations. Furthermore, 9-cis retinoic acid was also used as a positive control in the docking studies presented here. Both the protein and ligand models were prepared using MGLTools (version 1.5.7) [84] and screened virtually using AutoDock Vina [85]. The search space volume (4,032 Å^3^) was determined using MGLTools (center_x = 12.848, center_y = 29.174, center_z = 50.269, size_x = 16, size_y = 14, size_z = 18). The exhaustiveness was set to 8.

### 6.3. Cell Culture

UAS-GFP x KMT2A-MLLT3 cells were produced as described [82] and cultured in vitro using expansion medium (RPMI1640 medium, 15% FBS, Scf (50 ng/mL), IL3 (10 ng/mL), L-glutamine (2 mM), sodium pyruvate (1 mM), HEPES buffer (10 mM), penicillin/streptomycin (100 units/mL), and β-mercaptoethanol (50 µM)).

### 6.4. UAS/Gal4 Assay

UAS-GFP x KMT2A-MLLT3 cells were transduced with retroviruses MSCV-Gal4 (DNA binding domain, DBD)—RXRA (ligand binding domain, LBD)—IRES—mCherry. Cells were treated, and after 48 h, GFP measured by a ZE5 Flow Cytometer (Biorad).

### 6.5. Luciferase Detection

293T cells were transfected using Lipofectamine 2000 (Invitrogen). Six hours after transfection, the cells were collected and plated into a 48-well plate in 1% BSA media in triplicate and treated with compounds. After 40 h incubation, the cells were harvested and assayed for luciferase (Luc Assay System with Reporter Lysis Buffer, Promega) in a Beckman Coulter LD400 plate reader.

### 6.6. LXRE Assay

The LXRE-mediated assays were performed using human embryonic cells (HEK293) seeded at a density of 60,000 cells/well in a 24-well plate and maintained in DMEM (Hyclone) supplemented with 10% fetal bovine serum, 100 µg/mL streptomycin, 100 U/mL penicillin (Invitrogen, Carlsbad, CA, USA) at 37 degrees Celsius, 5% CO_2_ for 24 h. The cells were co-transfected with 250 ng of an LXRE-luciferase reporter gene, 50 ng of pSG5-human RXRα, and 20 ng of renilla control plasmid. The transfections were conducted using 1.25 µL of polyethylenimine (PEI) (Polysciences, Inc., Warrington, PA, USA) for 16–22 h. After transfection, the cells were treated with either ethanol vehicle control (0.1%), reference compound bexarotene (**1**) or analog, and/or T0901317 (an LXR ligand) at the indicated concentrations. After 24 h post treatment, the cells were lysed and the transcriptional activity mediated by the LXRE was measured using the Dual Luciferase Assay System (Promega, Madison, WI) in a Sirius FB12 luminometer (Berthold Detection Systems, Pforzheim, Germany) according to the manufacturer’s protocol. The data are a compilation of between three and six independent assays with each treatment group dosed in triplicate for each independent assay. The transcription efficiency on the LXRE was measured in comparison to the reference compound bexarotene (**1**) set to 100%. Bars on all graphs indicate standard deviation of the replicate experiments.

### 6.7. RARE Assay

Human embryonic kidney cells (HEK293) were plated at 60,000 cells per well in a 24-well plate and maintained as described above. After 24 h, the cells were transfected with 250 ng pTK-DR5(X2)-Luc, 25 ng pCMX-human RARα, and 20 ng renilla utilizing 1.25 µL polyethylenimine (PEI) per well for 24 h. The sequence of the double DR5 RARE is: 5′-AAAGGTCACCGAAAGGTCACCATCCCGGGAGGTCACCGAAAGGTCACC-3′ (DR5 responsive elements underlined). The cells were treated with ethanol vehicle (0.1%), all-trans-retinoic acid (RA, the ligand for RAR), or the indicated rexinoid at a final concentration of 10 nM. After 24 h of treatment, the retinoid activity was measured as described above (dual luciferase assay). The activity of compound **1** or analog divided by the activity of all-trans-RA (expressed as a percentage) represents the RARE activity. Three independent assays were conducted with triplicate samples for each treatment group. The value for RA was set to 100%.

### 6.8. Cell Viability and Growth Analysis

UAS-GFP x KMT2A-MLLT3 cells were plated at 10,000 cells per well in 96-well plates with indicated compounds in 200 µL. These cells doubled every 8–10 h. After 48 h, 10 µL were replated in new media with indicated compounds re-applied in 200 µL. After an additional 96 h, the number of viable cells in 50 µL was determined using a ZE5 flow cytometer (Bio-rad) using forward scatter/side scatter and PE exclusion to isolate viable cells.

### 6.9. Data Analysis

Statistical analysis was performed using Prism (Graphpad). T-test was performed, as appropriate. Error bars represent standard deviation. Data points without error bars have standard deviations below Graphpad’s limit to display. For Figure 8, Figure 9 and Figure 10, data are expressed as means ± SD. Statistical differences between two groups (generally the bexarotene control group versus bexarotene analog group) were determined by a two-sided Student’s *t*-test. A *p*-value of less than 0.05 was considered significant.

### 6.10. Mutagenicity and Toxicity Assay

All compounds were tested for toxicity and mutagenicity using a Saccharomyces cerevisiae based assay as described previously [58]. Toxicity was assessed in this assay (Table 1), comparing growth on plates to control treatments. Compounds were solubilized in DMSO at increasing concentrations and cells were incubated with the compounds for 3 h before plating on selective media or YPD to assess toxicity and mutagenicity. Cytotoxicity was assessed as described [86]. Growth of colonies on the full nutrient YPD plate for each treatment was compared to the DMSO only control. The concentration at which 50% cell death (as indicated by colony count compared to DMSO only control) +/−10% cell death is reported as the 50% killing rate. The highest concentration tested was 11 µg/µL.

### 6.11. HPLC

All tested compounds were assessed on a Waters Acquity UPLC with QDA and PDA detectors. Compounds were assayed in ESI-mode on an ACE Excel C18-PFP (1.7 µm, 50 mm × 2.1 mm) column using a 0.1% formic acid/water:acetonitrile gradient over 5 min. HPLC traces for compounds **25**–**36**, **37a**, and **37b** are available in the Supplementary Materials.

### 6.12. NMR and High Resolution Mass Spectrometry

A 400 MHz Bruker Avance III spectrometer was used to acquire ^1^H NMR and ^13^C NMR spectra. Chemical shifts (δ) are listed in ppm against residual non-deuterated solvent peaks in a given deuterated solvent (e.g., CHCl_3_ in CDCl_3_) as an internal reference. Coupling constants (J) are reported in Hz, and the abbreviations for splitting include: s, single; d, doublet; t, triplet; q, quartet; p, pentet; m, multiplet; br, broad. All ^13^C NMR spectra were acquired on a Bruker instrument at 100.6 MHz. Chemical shifts (δ) are listed in ppm against deuterated solvent carbon peaks as an internal reference. High resolution mass spectra were recorded using either a JEOL GCmate (2004), a JEOL LCmate (2002) high resolution mass spectrometer or an ABI Mariner (1999) ESI-TOF mass spectrometer. NMR spectra are available in the Supplementary Materials.

### 6.13. General Procedures

Removal of volatile solvents transpired under reduced pressure using a Büchi rotary evaporator and is referred to as removing solvents in vacuo. Thin layer chromatography was conducted on precoated (0.25 mm thickness) silica gel plates with 60F-254 indicator (Merck). Column chromatography was conducted using 230–400 mesh silica gel (E. Merck reagent silica gel 60). All tested compounds were analyzed for purity by NMR as well as HPLC analysis and were found to be >95% pure.

### 6.14. 1-isobutoxy-2-isopropylbenzene

**(39)** To a solution of 2-isopropylphenol (**38**) (12.5 mL, 92.9 mmols) and 1-bromo-2-methylpropane (20.5 mL, 189 mmols) in DMF (50 mL) was added finely ground potassium carbonate (13.9 g, 101 mmols) and potassium iodide (0.652 g, 3.9 mmols), and the reaction was stirred for 20 h at 70–75 °C. The reaction solution was then poured into water and extracted with ethyl acetate. The organic layers were washed with brine, dried over sodium sulfate, and concentrated in vacuo to provide a crude oil that was purified by column chromatography (1% ethyl acetate in hexanes) to give **39** as a colorless oil (8.7947 g, 50%): ^1^H NMR (400 MHz, CDCl_3_) δ 7.23 (d, J = 7.6, 1H), 7.16 (td, J = 8.0, 2.4, 1H), 6.93 (t, J = 7.6, 1H), 6.85 (d, J = 8.0, 1H), 3.76 (d, J = 6.4, 2H), 3.39 (hept, J = 6.8, 1H), 2.15 (nonet, J = 6.8, 1H), 1.27 (d, J = 6.8, 6H), 1.08 (d, J = 6.4, 6H); ^13^C NMR (100.6 MHz, CDCl_3_) δ156.3, 136.9, 126.4, 125.9, 120.2, 74.2, 28.5, 26.9, 22.6, 19.4; IR (neat) 2959, 1599, 1491, 1236 cm*^−^*^1^; GC-MS-CI (M + NH_4_)+ calcd for C_13_H_24_NO 210.1858, found 210.1850.

### 6.15. 1-isobutoxy-2-isopropyl-4-nitrobenzene (40)

To a solution of 1-isobutoxy-2-isopropylbenzene (**39**) (17.208 g, 89.486 mmols) in ethyl acetate (100 mL) at 0 °C was added concentrated (>90%) nitric acid (50.5 mL, 1.2 mols). The reaction was stirred at 0 °C for 40 min at which point it was carefully poured into water and extracted with ethyl acetate. The organic layer was washed with brine and dried over sodium sulfate to give a crude oil that consisted of **40** and **41** in a 3:1 ratio—TLC separates these isomers after four elutions in 1% ethyl acetate:hexanes (**41** R_f_ ~ 0.5 and **40** R_f_ ~ 0.45). This crude oil was purified by column chromatography (0.7% to 1% to 5% ethyl acetate in hexanes) to give **40** (9.6225 g, 45.3%) as a pale yellow oil: ^1^H NMR (400 MHz, CDCl_3_) δ 8.09 (d, J = 2.4, 1H), 8.06 (dd, J = 8.8, 2.8, 1H), 6.84 (d, J = 9.2, 1H), 3.83 (d, J = 6.4, 2H), 3.35 (hept, J = 6.8, 1H), 2.16 (nonet, J = 6.8, 1H), 1.25 (d, J = 6.8, 6H), 1.07 (d, J = 6.8, 6H); ^13^C NMR (100.6 MHz, CDCl_3_) δ161.5, 141.2, 138.0, 123.3, 121.9, 110.2, 28.3, 27.0, 22.1, 19.2; IR (neat) 2962, 1588, 1512, 1336, 1251 cm*^−^*^1^; ES-MS (M + Na)+ calcd for C_13_H_19_NO_3_Na 260.1263, found 260.1256.

### 6.16. Methyl 6-((4-isobutoxy-3-isopropylphenyl)amino)nicotinate (45)

A solution of 1-isobutoxy-2-isopropyl-4-nitrobenzene (**40**) (2.0064 g, 8.455 mmols) in ethyl acetate (183 mL) was passed through a 10% Pd/C cartridge at 1.0 mL/min in the ThalesNano H-cube*^®^* at 65 °C and 2–5 bar pressure. The resulting solution was concentrated in vacuo to give 4-isobutoxy-3-isopropylaniline (**42**) (1.7057 g, 97%) as a yellow oil that was used without further purification: ^1^H NMR (400 MHz, CDCl_3_) δ 6.66 (d, J = 8.4, 1H), 6.63 (d, J = 2.8, 1H), 6.50 (dd, J = 8.4, 2.8, 1H), 3.64 (d, J = 6.4, 2H), 3.63 (br s, 1H), 3.31 (hept, J = 6.8, 1H), 2.09 (nonet, J = 6.8, 1H), 1.20 (d, J = 6.8, 6H), 1.03 (d, J = 6.8, 6H); ^13^C NMR (100.6 MHz, CDCl_3_) δ 149.8, 138.9, 138.2, 114.3, 113.1, 112.6, 75.2, 28.5, 26.8, 22.6, 19.4. To a solution of **42** (1.783 g, 8.60 mmols) and methyl 6-chloronicotinate (1.6567 g, 9.655 mmols) in dioxane (15.0 mL) was added para-toluenesulfonic acid monohydrate (1.7977 g, 9.45 mmols) and the reaction was refluxed overnight in an oil bath at 111 °C. The reaction was cooled to room temperature, and then the mixture was poured into water, extracted with ethyl acetate, and the organic layers were washed with brine, dried over sodium sulfate and concentrated to give a crude oil that was purified by column chromatography (150 mL SiO_2_, 6% ethyl acetate:hexanes) to give **45** (1.9259 g, 65.4%) as a white crystalline solid, m.p. 123–124 °C: ^1^H NMR (400 MHz, CDCl_3_) δ 8.76 (dd, J = 2.4, 0.8, 1H), 8.00 (dd, J = 8.8, 2.4, 1H), 7.77 (br s, 1H), 7.11 (s, 1H), 7.09 (dd, J = 7.6, 2.8, 1H), 6.82 (dd, J = 7.6, 0.8, 1H), 6.65 (dd, J = 8.8, 0.8, 1H), 3.86 (s, 3H), 3.74 (d, J = 6.0, 2H), 3.36 (hept, J = 6.8, 1H), 2.13 (nonet, J = 6.8, 1H), 1.22 (d, J = 6.8, 6H), 1.06 (d, J = 6.8, 6H); ^13^C NMR (100.6 MHz, CDCl_3_) δ 166.1, 160.1, 154.1, 151.1, 138.9, 138.4, 131.0, 122.2, 121.9, 115.9, 111.6, 105.5, 51.6, 28.4, 26.9, 22.5, 19.3; IR (neat) 3235, 2953, 1721, 1612, 1598, 1496, 1277, 1115 cm*^−^*^1^; ES-MS (M + H)+ calcd for C_20_H_27_N_2_O_3_ 343.2022, found 343.2024.

### 6.17. Methyl 2-((4-isobutoxy-3-isopropylphenyl)amino)pyrimidine-5-carboxylate(46)

To a solution of **42** (1.7057 g, 8.23 mmols) and methyl 2-chloropyrimidine-5-carboxylate (1.5852 g, 9.1859 mmols) in dioxane (15.0 mL) was added para-toluenesulfonic acid monohydrate (1.7197 g, 9.04 mmols) and the reaction was refluxed overnight in an oil bath at 111 °C. The reaction was cooled to room temperature, and then the mixture was poured into water, extracted with ethyl acetate, and the organic layers were washed with brine, dried over sodium sulfate, and concentrated to give a crude oil that was purified by column chromatography (150 mL SiO_2_, 10% ethyl acetate:hexanes) to give **46** (2.1821 g, 71.3%) as a white crystalline solid, m.p. 122–124.2 °C: ^1^H NMR (400 MHz, CDCl_3_) δ 8.92 (s, 2H), 8.36 (br s, 1H), 7.44 (dd, J = 8.8, 2.4, 1H), 7.27 (d, J = 2.4, 1H), 6.83 (d, J = 8.8, 1H), 3.89 (s, 3H), 3.74 (d, J = 6.0, 2H), 3.37 (hept, J = 6.8, 1H), 2.12 (nonet, J = 6.8, 1H), 1.24 (d, J = 6.8, 6H), 1.06 (d, J = 6.4, 6H); ^13^C NMR (100.6 MHz, CDCl_3_) δ 164.7, 161.7, 160.0, 153.5, 137.7, 130.4, 120.1, 120.1, 114.4, 111.3, 74.5, 51.8, 28.4, 26.9, 22.5, 19.3; IR (neat) 3261, 2956, 1721, 1597, 803 cm*^−^*^1^; ES-MS (M + Na)+ calcd for C_29_H_25_N_3_O_3_Na 366.1794, found 366.1801.

### 6.18. Methyl 2-fluoro-4-((4-isobutoxy-3-isopropylphenyl)amino)benzoate (50)

To a solution of **42** (1.3452 g, 6.49 mmol), methyl 2-fluoro-4-iodobenzoate **47** [74] (1.9807 g, 7.07 mmols), Cs_2_CO_3_ (5.5562 g, 17.08 mmol), and rac-BINAP (0.3386 g, 0.55 mmol) in toluene (8.6 mL) in a 100 mL round-bottomed flask was added Pd_2_(dba)_3_ (0.319 g, 1.82 mmol). The solution was sparged with nitrogen for 5 min, then a reflux condenser was fitted to the flask, the atmosphere was evacuated and back-filled with nitrogen (three times), and then the reaction was heated to reflux with stirring in an oil bath (125–120 °C) for 22 h. After cooling the reaction to room temperature, excess cesium carbonate and other solid particulates were filtered and washed with ethyl acetate, and the organic filtrate was concentrated in vacuo to obtain a crude product that was purified by column chromatography (150 mL SiO_2_, 6% ethyl acetate:hexanes to 12% ethyl acetate: hexanes) to give **50** (2.147 g, 88.9%) as a crystalline solid, m.p. 93.4–100.9 °C: ^1^H NMR (400 MHz, CDCl_3_) δ 7.84–7.75 (m, 1H), 7.01 (br s, 1H), 6.97–6.72 (m, 1H), 6.80 (d, J = 8.4, 1H), 6.54 (d, J = 8.4, 1H), 6.47 (d, J = 13.6, 1H), 3.86 (s, 3H), 3.75 (d, J = 6.0, 2H), 3.35 (hept, J = 6.8, 1H), 2.13 (nonet, J = 6.8, 1H), 1.21 (d, J = 6.8, 6H), 1.06 (d, J = 6.8, 6H); ^13^C NMR (100.6 MHz, CDCl_3_) δ 165.3, 164.9, 164.9, 164.5, 164.4, 164.2, 161.6, 155.2, 153.9, 152.0, 151.9, 139.4, 138.5, 136.9, 133.6, 133.0, 131.9, 128.9, 128.3, 125.7, 125.5, 122.1, 121.7, 116.9, 112.1, 112.0, 111.9, 111.7, 109.7, 109.4, 109.3, 107.4, 107.3, 100.6, 100.3, 74.6, 51.6, 28.4, 26.9, 22.5, 22.4, 19.3; IR (neat) 3343, 2960, 1686, 1618, 1603, 1498, 1439 cm*^−^*^1^; ES-MS (M + Na)+ calcd for C_21_H_26_NFO_3_Na 382.1794, found 382.1795.

### 6.19. Methyl 4-((4-isobutoxy-3-isopropylphenyl)amino)benzoate (51)

To a solution of **42** (1.1807 g, 5.695 mmol), methyl 4-iodobenzoate **48** (1.6531 g, 6.308 mmols), Cs_2_CO_3_ (4.9777 g, 15.28 mmol), and rac-BINAP (0.3097 g, 0.50 mmol) in toluene (7.4 mL) in a 100 mL round-bottomed flask was added Pd_2_(dba)_3_ (0.2868 g, 1.64 mmol). The solution was sparged with nitrogen for 5 min, then a reflux condenser was fitted to the flask, the atmosphere was evacuated and back-filled with nitrogen (three times), and the reaction was heated to reflux with stirring in an oil bath (125–120 °C) for 22 h. After cooling the reaction to room temperature, excess cesium carbonate and other solid particulates were filtered and washed with ethyl acetate, and the organic filtrate was concentrated in vacuo to give a crude product that was purified by column chromatography (150 mL SiO_2_, 6% ethyl acetate:hexanes to 12% ethyl acetate: hexanes) to give **51** (1.0494 g, 54%) as a crystalline solid, m.p. 101.1–103.8 °C: ^1^H NMR (400 MHz, CDCl_3_) δ 8.13 (d, J = 8.4, 1H), 7.88 (br s, 1H), 7.69 (d, J = 8.4, 1H), 7.06 (d, J = 8.8, 1H), 6.80 (d, J = 8.4, 4H), 3.86 (s, 3H), 3.74 (d, J = 6.4, 2H), 3.35 (hept, J = 7.2, 1H), 2.13 (nonet, J = 6.8, 1H), 1.21 (d, J = 7.2, 6H), 1.06 (d, J = 6.8, 6H); ^13^C NMR (100.6 MHz, CDCl_3_) δ 167.0, 151.2, 131.5, 131.5, 131.4, 131.4, 130.8, 130.2, 129.7, 127.2, 121.4, 113.1, 74.6, 51.5, 28.5, 26.9, 22.5, 19.3; IR (neat) 3379, 2958, 1684, 1612, 1591, 1283, 1177 cm*^−^*^1^; ES-MS (M + Na)+ calcd for C_21_H_27_NO_3_Na 364.1889, found 364.1896.

### 6.20. Methyl 4-((4-isobutoxy-3-isopropylphenyl)amino)-3-nitrobenzoate (52)

To a solution of **42** (1.68 g, 8.10 mmol), methyl 4-iodo-3-nitrobenzoate **49** [68] (2.63 g, 8.57 mmols), Cs_2_CO_3_ (5.44 g, 16.7 mmol), and rac-BINAP (0.4286 g, 0.7472 mmol) in toluene (10.8 mL) in a 100 mL round-bottomed flask was added Pd_2_(dba)_3_ (0.404 g, 2.31 mmol). The solution was sparged with nitrogen for 5 min, then a reflux condenser was fitted to the flask, the atmosphere was evacuated and back-filled with nitrogen (three times), and the reaction was heated to reflux with stirring in an oil bath (125–120 °C) for 22 h. After cooling the reaction to room temperature, excess cesium carbonate and other solid particulates were filtered and washed with ethyl acetate, and the organic filtrate was concentrated in vacuo to give a crude product that was purified by column chromatography (150 mL SiO_2_, 4% ethyl acetate:hexanes to 12% ethyl acetate: hexanes) to give **52** (2.5619 g, 81.8%) as a crystalline solid, m.p. 78.1–82.3 °C: ^1^H NMR (400 MHz, CDCl_3_) δ 9.71 (br s, 1H), 8.90 (d, J = 2.0, 1H), 7.90 (ddd, J = 9.2, 2.0, 0.4, 1H), 7.07 (d, J = 2.8, 1H), 7.04 (dd, J = 8.8, 2.8, 4H), 7.00 (d, J = 8.8, 1H), 6.86 (d, J = 8.4, 1H), 3.89 (s, 3H), 3.77 (d, J = 6.4, 2H), 3.37 (hept, J = 6.8, 1H), 2.13 (nonet, J = 6.8, 1H), 1.22 (d, J = 6.8, 6H), 1.07 (d, J = 6.8, 6H); ^13^C NMR (100.6 MHz, CDCl_3_) δ 165.4, 155.2, 147.2, 138.9, 135.7, 131.5, 129.5, 129.2, 128.9, 128.3, 124.0, 123.9, 118.2, 115.5, 111.7, 74.5, 52.0, 28.4, 26.9, 22.4, 19.3; IR (neat) 3340, 2958, 1716, 1705, 1622, 1212, 757 cm*^−^*^1^; ES-MS (M + Na)+ calcd for C_21_H_26_N_2_O_5_Na 409.1740, found 409.1750.

### 6.21. Methyl 6-(ethyl(4-isobutoxy-3-isopropylphenyl)amino)nicotinate (53)

To a flame-dried, 100 mL round-bottomed flask equipped with a magnetic stir bar was added a 60% dispersion of sodium hydride in mineral oil (0.2351 g, 5.88 mmol). The dispersion of sodium hydride was washed with hexanes (3 mL, twice) and dried under vacuum and suspended in 3.0 mL of DMF under nitrogen. To this solution of sodium hydride in DMF was added a solution of **45** (0.8331 g, 2.433 mmol) in DMF (9.2 mL), and the reaction was stirred for 15 min, and then ethyl iodide (0.30 mL, 3.8 mmol) was added, and the reaction was stirred for 1 h. The reaction was poured into water and extracted with ethyl acetate. The combined organic layers were washed with brine, dried over sodium sulfate, filtered, and concentrated in vacuo to yield a crude product that was purified by column chromatography (150 mL SiO_2_, 6% ethyl acetate:hexanes) to give **53** (0.3629 g, 40%) as a colorless oil: ^1^H NMR (400 MHz, CDCl_3_) δ 8.83 (dd, J = 2.0, 0.4, 1H), 7.77 (dd, J = 8.8, 2.4, 1H), 7.01 (d, J = 2.4, 1H), 6.96 (dd, J = 8.4, 2.4, 1H), 6.86 (d, J = 8.4, 1H), 6.14 (dd, J = 8.8, 0.4, 1H), 4.00 (q, J = 7.2, 2H), 3.85 (s, 3H), 3.76 (d, J = 6.4, 2H), 3.36 (hept, J = 6.8, 1H), 2.14 (nonet, J = 6.8, 1H), 1.21 (t, J = 7.2, 3H), 1.20 (d, J = 6.8, 6H), 1.08 (d, J = 6.8, 6H); ^13^C NMR (100.6 MHz, CDCl_3_) δ 165.3, 162.8, 159.7, 155.1, 138.1, 135.3, 125.4, 125.3, 112.7, 111.2, 74.2, 51.6, 46.4, 28.4, 27.1, 22.4, 19.4, 12.7; IR (neat) 2959, 1711, 1596, 1495, 1263 cm*^−^*^1^; ES-MS (M)+ calcd for C_22_H_30_N_2_O_3_ 370.2256, found 370.2242.

### 6.22. Methyl 2-(ethyl(4-isobutoxy-3-isopropylphenyl)amino)pyrimidine-5-carboxylate (54)

To a flame-dried, 100 mL round-bottomed flask equipped with a magnetic stir bar was added a 60% dispersion of sodium hydride in mineral oil (0.2377 g, 5.95 mmol). The dispersion of sodium hydride was washed with hexanes (3 mL, twice) and dried under vacuum and suspended in 3.0 mL of DMF under nitrogen. To this solution of sodium hydride in DMF was added a solution of **46** (0.8442 g, 2.458 mmol) in DMF (9.2 mL), and the reaction was stirred for 15 min, and then ethyl iodide (0.30 mL, 3.8 mmol) was added, and the reaction was stirred for 1 h. The reaction was poured into water and extracted with ethyl acetate. The combined organic layers were washed with brine, dried over sodium sulfate, filtered, and concentrated in vacuo to yield a crude product that was purified by column chromatography (150 mL SiO_2_, 6% ethyl acetate:hexanes) to give **54** (0.2698 g, 29.5%) as a white crystalline solid, m.p. 122.8–125.8 °C: ^1^H NMR (400 MHz, CDCl_3_) δ 8.83 (br s, 2H), 7.03 (d, J = 2.8, 1H), 6.99 (dd, J = 8.4, 2.8, 1H), 6.85 (d, J = 8.4, 1H), 4.02 (q, J = 6.8, 2H), 3.86 (s, 3H), 3.75 (d, J = 6.4, 2H), 3.35 (hept, J = 6.8, 1H), 2.13 (nonet, J = 6.8, 1H), 1.24 (t, J = 6.8, 3H), 1.23 (d, J = 6.8, 6H), 1.06 (d, J = 6.8, 6H); ^13^C NMR (100.6 MHz, CDCl_3_) δ 165.3, 162.8, 159.7, 155.1, 138.1, 135.3, 125.4, 125.3, 112.7, 111.2, 74.2, 51.6, 46.4, 28.4, 27.1, 22.4, 19.4, 12.7; IR (neat) 2960, 1708, 1595, 1494, 1284, 805 cm*^−^*^1^; ES-MS (M + Na)+ calcd for C_21_H_29_N_3_O_3_Na 394.2107, found 394.2109.

### 6.23. Methyl 4-(ethyl(4-isobutoxy-3-isopropylphenyl)amino)-2-fluorobenzoate (55)

To a flame-dried, 100 mL round-bottomed flask equipped with a magnetic stir bar was added a 60% dispersion of sodium hydride in mineral oil (0.2461 g, 6.16 mmol). The dispersion of sodium hydride was washed with hexanes (3 mL, twice) and dried under vacuum and suspended in 3.0 mL of DMF under nitrogen. To this solution of sodium hydride in DMF was added a solution of **50** (0.8918 g, 2.301 mmol) in DMF (9.2 mL), and the reaction was stirred for 15 min, and then ethyl iodide (0.30 mL, 3.8 mmol) was added, and the reaction was stirred for 1 h. The reaction was poured into water and extracted with ethyl acetate. The combined organic layers were washed with brine, dried over sodium sulfate, filtered, and concentrated in vacuo to yield a crude product that was purified by column chromatography (150 mL SiO_2_, 6% ethyl acetate:hexanes) to give **55** (0.7741 g, 80.5%) as a white crystalline solid, m.p. 66–71 °C: ^1^H NMR (400 MHz, CDCl_3_) δ 7.70 (t, J = 8.8, 1H), 6.98 (d, J = 2.4, 1H), 6.93 (dd, J = 8.8, 2.4, 1H), 6.84 (d, J = 8.8, 1H), 6.32 (dd, J = 8.8, 2.4, 1H), 6.22 (dd, J = 15.2, 2.4, 1H), 3.85 (s, 3H), 3.76 (d, J = 6.0, 2H), 3.69 (q, J = 7.2, 2H), 3.35 (hept, J = 6.8, 1H), 2.14 (nonet, J = 6.8, 1H), 1.22 (t, J = 7.2, 3H), 1.22 (d, J = 6.8, 6H), 1.08 (d, J = 6.4, 6H); ^13^C NMR (100.6 MHz, CDCl_3_) δ 165.1, 165.1, 162.6, 155.0, 154.1, 154.0, 139.0, 137.2, 133.1, 133.0, 126.0, 125.9, 111.9, 108.1, 105.2, 105.1, 99.8, 99.6, 74.4, 51.5, 46.9, 28.5, 27.0, 22.5, 19.4, 12.2; IR (neat) 2957, 1707, 1692, 1620, 1495, 1299, 763 cm*^−^*^1^; ES-MS (M)+ calcd for C_23_H_30_FNO_3_ 387.2210, found 387.2200.

### 6.24. Methyl 4-(ethyl(4-isobutoxy-3-isopropylphenyl)amino)benzoate (56)

To a flame-dried, 100 mL round-bottomed flask equipped with a magnetic stir bar was added a 60% dispersion of sodium hydride in mineral oil (0.2417 g, 6.05 mmol). The dispersion of sodium hydride was washed with hexanes (3 mL, twice) and dried under vacuum and suspended in 3.0 mL of DMF under nitrogen. To this solution of sodium hydride in DMF was added a solution of **51** (0.8581 g, 2.513 mmol) in DMF (9.2 mL), and the reaction was stirred for 15 min, and then ethyl iodide (0.30 mL, 3.8 mmol) was added, and the reaction was stirred for 1 h. The reaction was poured into water and extracted with ethyl acetate. The combined organic layers were washed with brine, dried over sodium sulfate, filtered, and concentrated in vacuo to yield a crude product that was purified by column chromatography (150 mL SiO_2_, 6% ethyl acetate:hexanes) to give **56** (0.8655 g, 93.2%) as a white crystalline solid, m.p. 86.8–89.9 °C: ^1^H NMR (400 MHz, CDCl_3_) δ 7.80 (dd, J = 7.2, 2.4, 1H), 7.01 (d, J = 2.8, 1H), 6.95 (dd, J = 8.4, 2.4, 1H), 6.84 (d, J = 8.4, 1H), 6.58 (dd, J = 7.2, 2.0, 1H), 3.84 (s, 3H), 3.76 (d, J = 6.0, 2H), 3.71 (q, J = 7.2, 2H), 3.35 (hept, J = 7.2, 1H), 2.14 (nonet, J = 6.8, 1H), 1.24 (t, J = 6.8, 3H), 1.21 (d, J = 7.2, 6H), 1.08 (d, J = 6.8, 6H); ^13^C NMR (100.6 MHz, CDCl_3_) δ 167.3, 154.7, 152.2, 138.8, 137.9, 131.0, 126.0, 125.9, 117.3, 112.0, 111.8, 74.4, 51.4, 46.7, 28.5, 27.0, 22.5, 19.4, 12.3; IR (neat) 2958, 1698, 1609, 1598, 1495, 1269, 1179, 767 cm*^−^*^1^; ES-MS (M)+ calcd for C_23_H_31_NO_3_Na 392.2202, found 392.2196.

### 6.25. Methyl 3-amino-4-((4-isobutoxy-3-isopropylphenyl)amino)benzoate (57)

A solution of **52** (1.2000 g, 3.105 mmols) in ethyl acetate (63 mL) was passed through a 10% Pd/C cartridge at 1.0 mL/minute in the ThalesNano H-cube*^®^* at 65 °C and 2–5 bar pressure. The resulting solution was concentrated in vacuo to give **57** (quantitative yield) as a yellow oil that was used without further purification: ^1^H NMR (400 MHz, CDCl_3_) δ 7.47 (d, J = 2.0, 1H), 7.45 (dd, J = 5.6, 2.0, 1H), 6.95 (d, J = 8.8, 1H), 6.94 (d, J = 2.8, 1H), 6.83 (dd, J = 8.8, 2.8, 1H), 6.78 (d, J = 8.8, 1H), 5.52 (br s, 1H), 3.85 (s, 3H), 3.72 (d, J = 6.4), 3.50 (br s, 2H), 3.35 (hept, J = 6.8, 1H), 2.13 (nonet, J = 6.8, 1H), 1.20 (d, J = 6.8, 6H), 1.06 (d, J = 6.8, 6H); ^13^C NMR (100.6 MHz, CDCl_3_) δ 167.2, 152.4, 139.3, 138.3, 134.7, 134.2, 122.9, 121.6, 119.6, 118.8, 114.1, 111.9, 60.3, 51.6, 28.5, 26.9, 22.5, 21.0, 19.4, 14.1; IR (neat) 3379, 2959, 1697, 1496, 1296 cm*^−^*^1^; ES-MS (M + Na)+ calcd for C_21_H_28_N_2_O_3_Na 379.1998, found 379.1998.

### 6.26. Methyl 1-(4-isobutoxy-3-isopropylphenyl)-1H-benzo[d][1,2,3]triazole-5-carboxylate (58)

To a solution of **57** (1.0839 g, 3.04 mmols) in THF (14.0 mL) at 0 °C was added a 1:1 solution of concentrated sulfuric acid and water (14.0 mL) followed by a solution of NaNO_2_ (0.3292 g, 4.77 mmols) in water (14.0 mL), and the reaction was allowed to warm to room temperature and stirred for 1 h. The reaction was poured into ethyl acetate, extracted, and the organic layers were combined, washed with saturated sodium bicarbonate and brine, dried over sodium sulfate, filtered, and concentrated to give crude **58**, which was purified by column chromatography (150 mL SiO_2_, 4–6% ethyl acetate:hexanes) to give **58** (0.9791 g, 87.6%) as a crystalline solid, m.p. 86–89 °C: ^1^H NMR (400 MHz, CDCl_3_) δ 8.83 (d, J = 0.8, 1H), 8.19 (dd, J = 8.8, 1.6, 1H), 7.65 (dd, J = 8.8, 0.4, 1H), 7.53 (d, J = 2.8, 1H), 7.45 (dd, J = 8.8, 2.8, 1H), 6.99 (d, J = 8.8, 1H), 3.98 (s, 3H), 3.82 (d, J = 6.0, 2H), 3.44 (hept, J = 6.8, 1H), 2.18 (nonet, J = 6.4, 1H), 1.28 (d, J = 6.8, 6H), 1.09 (d, J = 6.4, 6H); ^13^C NMR (100.6 MHz, CDCl_3_) δ 166.4, 156.8, 145.9, 138.9, 134.8, 129.0, 128.6, 126.4, 122.9, 121.5, 121.4, 111.4, 110.2, 52.4, 28.4, 27.1, 22.3, 19.3; IR (neat) 2962, 1711, 1616, 1505, 1471, 1239, 750 cm*^−^*^1^; ES-MS (M + Na)+ calcd for C_21_H_25_N_3_O_3_Na 390.1794, found 390.1794.

### 6.27. 6-(ethyl(4-isobutoxy-3-isopropylphenyl)amino)nicotinic acid (25) (NEt-4IB)

To a solution of **53** (0.9265 g, 2.501 mmols) in methanol (9.0 mL) was added a solution of KOH (0.4545 g, 8.100 mmols) in water (0.56 mL) and the solution was heated to reflux with stirring for 1 h. The solution was then cooled to room temperature, quenched with 1 N HCl (80 mL), extracted with ethyl acetate, the organic layer was washed with brine, dried over sodium sulfate, filtered, and concentrated in vacuo to give a crude product that was purified by column chromatography (25 mL SiO_2_, 20–52% ethyl acetate:hexanes) to give **25** (0.7184 g, 80.5%) as a crystalline solid, m.p. 158.5–168.2 °C: ^1^H NMR (400 MHz, CDCl_3_) δ10.90 (br s, 1H), 8.92 (d, J = 2.4, 1H), 7.82 (dd, J = 9.2, 2.4, 1H), 7.03 (d, J = 2.4, 1H), 6.98 (dd, J = 8.4, 2.4, 1H), 6.87 (d, J = 8.4, 1H), 6.17 (d, J = 9.2, 1H), 4.03 (q, J = 7.2, 2H), 3.77 (d, J = 6.4, 2H), 3.36 (hept, J = 6.8, 1H), 2.15 (nonet, J = 6.4, 1H), 1.24 (t, J = 7.2, 3H), 1.22 (d, J = 7.2, 6H), 1.08 (d, J = 6.4, 6H); ^13^C NMR (100.6 MHz, CDCl_3_) δ 171.4, 160.9, 155.4, 151.6, 139.1, 137.9, 135.6, 126.0, 125.9, 113.3, 111.9, 107.5, 74.4, 45.6, 28.4, 27.0, 22.5, 19.3, 12.9; IR (neat) 2959, 1661, 1592, 1495, 1271, 785 cm*^−^*^1^; ES-MS (M–H)- calcd for C_21_H_27_N_2_O_3_ 355.2022, found 355.2022.

### 6.28. 2-(ethyl(4-isobutoxy-3-isopropylphenyl)amino)pyrimidine-5-carboxylic acid (28)

To a solution of **54** (0.7580 g, 2.04 mmols) in methanol (7.3 mL) was added a solution of KOH (0.3846 g, 6.854 mmols) in water (0.46 mL) and the solution was heated to reflux with stirring for 1 h. The solution was then cooled to room temperature, quenched with 1 N HCl (80 mL), and the resulting precipitate was filtered to give a crude product that was purified by column chromatography (25 mL SiO_2_, 20–60% ethyl acetate:hexanes) to give **28** (0.6753 g, 92.6%) as a crystalline solid, m.p. 201.5-202.8 °C: ^1^H NMR (400 MHz, CDCl_3_) δ10.65 (br s, 1H), 8.88 (br s, 2H), 7.03 (d, J = 2.4, 1H), 7.00 (dd, J = 8.4, 2.4, 1H), 6.86 (d, J = 8.4, 1H), 4.05 (q, J = 7.2, 2H), 3.74 (d, J = 6.0, 2H), 3.35 (hept, J = 6.8, 1H), 2.13 (nonet, J = 6.4, 1H), 1.27 (t, J = 7.2, 3H), 1.25 (d, J = 6.8, 6H), 1.08 (d, J = 6.8, 6H); ^13^C NMR (100.6 MHz, CDCl_3_) δ 169.8, 162.7, 160.4, 155.3, 138.3, 135.0, 125.5, 125.3, 112.0, 111.3, 74.2, 46.6, 28.4, 27.1, 22.4, 19.4, 12.7; IR (neat) 2961, 1663, 1589, 1518, 1495, 1271, 807 cm*^−^*^1^; ES-MS (M–H)- calcd for C_20_H_26_N_3_O_3_ 356.1974, found 356.1965.

### 6.29. 4-(ethyl(4-isobutoxy-3-isopropylphenyl)amino)-2-fluorobenzoic acid (29)

To a solution of **55** (0.6837 g, 1.764 mmols) in methanol (6.0 mL) was added a solution of KOH (0.3493 g, 6.225 mmols) in water (0.41 mL) and the solution was heated to reflux with stirring for 1 h. The solution was then cooled to room temperature, quenched with 1 N HCl (80 mL), and the resulting precipitate was filtered to give a crude product that was purified by column chromatography (25 mL SiO_2_, 10–60% ethyl acetate:hexanes) to give **29** (0.4821 g, 73%) as a crystalline solid, m.p. 184–186 °C: ^1^H NMR (400 MHz, CDCl_3_) δ10.01 (br s, 1H), 7.77 (t, J = 9.2, 1H), 6.99 (d, J = 2.4, 1H), 6.94 (dd, J = 8.4, 2.4, 1H), 6.85 (d, J = 8.8, 1H), 6.34 (d, J = 2.4, 1H), 6.32 (d, J = 2.4, 1H), 3.76 (d, J = 6.4, 2H), 3.70 (q, J = 7.2, 2H), 3.74 (d, J = 7.2, 2H), 3.35 (hept, J = 6.8, 1H), 2.15 (nonet, J = 6.8, 1H), 1.24 (t, J = 7.2, 3H), 1.22 (d, J = 6.8, 6H), 1.08 (d, J = 6.8, 6H); ^13^C NMR (100.6 MHz, CDCl_3_) δ 169.7, 165.8, 163.3, 155.2, 154.8, 154.7, 139.1, 137.0, 133.7, 126.0, 125.9, 111.9, 108.1, 104.1, 104.0, 99.8, 99.5, 74.4, 47.0, 28.4, 27.0, 22.5, 19.3, 12.2; IR (neat) 2958, 1667, 1613, 1600, 1496, 1299, 1273, 1244, 836 cm*^−^*^1^; ES-MS (M–H)- calcd for C_22_H_27_FNO_3_ 372.1975, found 372.1982.

### 6.30. 4-(ethyl(4-isobutoxy-3-isopropylphenyl)amino)benzoic acid (30)

To a solution of **56** (0.7035 g, 1.904 mmols) in methanol (6.8 mL) was added a solution of KOH (0.3626 g, 6.462 mmols) in water (0.43 mL) and the solution was heated to reflux with stirring for 1 h. The solution was then cooled to room temperature, quenched with 1 N HCl (80 mL), and the resulting precipitate was filtered to give a crude product that was purified by column chromatography (25 mL SiO_2_, 10–60% ethyl acetate:hexanes) to give **30** (0.3594 g, 53.1%) as a crystalline solid, m.p. 179.4–181.0 °C: ^1^H NMR (400 MHz, CDCl_3_) δ10.89 (br s, 1H), 7.88 (d, J = 8.8, 2H), 7.03 (d, J = 2.8, 1H), 6.97 (dd, J = 8.8, 2.8, 1H), 6.86 (d, J = 8.4, 1H), 6.60 (d, J = 9.2, 2H), 3.77 (d, J = 6.4, 2H), 3.75 (q, J = 7.2, 2H), 3.36 (hept, J = 6.8, 1H), 2.15 (nonet, J = 6.8, 1H), 1.25 (t, J = 7.2, 3H), 1.23 (d, J = 7.2, 6H), 1.09 (d, J = 6.8, 6H); ^13^C NMR (100.6 MHz, CDCl_3_) δ 172.4, 154.8, 152.9, 138.9, 137.7, 131.8, 126.0, 116.3, 112.0, 111.8, 74.4, 46.8, 28.5, 27.0, 22.5, 19.4, 12.3; IR (neat) 2955, 1664, 1593, 1268, 1273, 1181, 773 cm*^−^*^1^; ES-MS (M–H)- calcd for C_22_H_28_NO_3_ 354.2069, found 354.2077.

### 6.31. 1-(4-isobutoxy-3-isopropylphenyl)-1H-benzo[d][1,2,3]triazole-5-carboxylic acid (31)

To a solution of **58** (0.7544 g, 2.053 mmols) in methanol (5.2 mL) was added a solution of KOH (0.3795 g, 6.764 mmols) in water (0.45 mL) and the solution was heated to reflux with stirring for 1 h. The solution was then cooled to room temperature, quenched with 1 N HCl (80 mL), and the resulting precipitate was filtered to give a crude product that was purified by column chromatography (150 mL SiO_2_, 20–60% ethyl acetate:hexanes) to give **31** (0.5182 g, 71.4%) as a crystalline solid, m.p. 177.3–178.7 °C: ^1^H NMR (400 MHz, CDCl_3_) δ10.91 (br s, 1H), 8.98 (t, J = 0.8, 1H), 8.27 (dd, J = 8.8, 1.6, 1H), 7.71 (dd, J = 8.8, 0.4, 1H), 7.55 (d, J = 2.4, 1H), 7.47 (dd, J = 8.8, 2.4, 1H), 7.01 (d, J = 8.8, 1H), 3.84 (d, J = 6.4, 2H), 3.45 (hept, J = 6.8, 1H), 2.19 (nonet, J = 6.8, 1H), 1.30 (d, J = 6.8, 6H), 1.10 (d, J = 6.8, 6H); ^13^C NMR (100.6 MHz, CDCl_3_) δ 171.3, 156.9, 145.8, 139.0, 135.3, 129.0, 128.9, 125.7, 124.0, 121.6, 121.5, 111.5, 110.4, 74.7, 28.4, 27.1, 22.4, 19.3; IR (neat) 2955, 1675, 1613, 1495, 1302, 1242, 1028, 814 cm*^−^*^1^; ES-MS (M–H)- calcd for C_20_H_22_N_3_O_3_ 352.1661, found 352.1657.

### 6.32. 1,1,3,3,5-. pentamethyl-2,3-dihydro-1H-indene (60)

To 2,4-dimethylpentane-2,4-diol (**59**) (5.00 g, 37.8 mmols) in a 100 mL round bottom flask was added concentrated hydrochloric acid (50.0 mL). The reaction was slightly diluted with water and extracted with hexanes. The hexanes was concentrated and the crude product was run through a column of silica gel (25 mL) in hexanes. The fractions containing the product were combined and concentrated to give crude 2,4-dichloro-2,4-dimethylpentane as a colorless oil (2.94 g, 46%) that was used without further purification. The crude 2,4-dichloro-2,4-dimethylpentane (2.94 g, 17.4 mmols) was dissolved in dichloromethane (10.0 mL) in a 100 mL round bottom flask and toluene (18.2 mL) was added. To this solution was slowly added aluminum chloride (1.80 g). The reaction was stirred at reflux in an oil bath for 15 min, then cooled to room temperature and poured into ice. The organics were extracted with ethyl acetate, and the organic layers were dried over sodium sulfate, filtered, and concentrated to give a crude oil that was purified by column chromatography (silica gel; hexanes) to give **60** (3.024 g, 92%) as a colorless oil: ^1^H NMR (400 MHz, CDCl_3_) δ 7.03–7.04 (m, 2H), 6.95 (s, 1H), 2.37 (s, 3H), 1.93 (s, 2H), 1.32 (s, 6H), 1.31 (s, 6H).

### 6.33. Methyl 2-(1,1,3,3,6-pentamethyl-2,3-dihydro-1H-indene-5-carbonyl)pyrimidine-5-carboxylate (65)

To a solution of **60** (3.05 g, 16.0 mmols) and methyl 2-(chlorocarbonyl)pyrimidine-5-carboxylate **63** (3.19 g, 15.9 mmols) in dichloromethane (35 mL) in a 100 mL round bottom flask was slowly added aluminum chloride (5.6 g) and the resulting mixture was stirred in an oil bath at reflux for 15 min. The reaction solution was cooled to room temperature and quenched by pouring onto 100 mL of an ice water solution. The solution was extracted with ethyl acetate, and the combined organic layers were dried over sodium sulfate, filtered, and concentrated to give a crude product that was purified by column chromatography (silica gel; 1:9 ethyl acetate:hexanes to 1:4 ethyl acetate:hexane) to give pure **65** (1.5869 g, 28%) as an orange, crystalline solid (98.1–103.2 °C): ^1^H NMR (400 MHz, CDCl_3_) δ 9.42 (s, 2H), 7.17 (s, 1H), 7.03 (s, 1H), 4.02 (s, 3H), 2.44 (s, 3H), 1.92 (s, 2H), 1.31 (s, 6H), 1.23 (s, 6H); ^13^C NMR (100.6 MHz, CDCl_3_) δ193.2, 166.0, 163.5, 158.5, 156.6, 148.6, 138.9, 133.5, 126.1, 126.0, 124.1, 56.5, 52.9, 42.8, 42.2, 31.3, 31.1, 21.3. ES-MS (M + Na)+ calcd for C_21_H_24_N_2_O_3_Na 375.1685, found 375.1668.

### 6.34. Methyl 4-(1,1,3,3,6-pentamethyl-2,3-dihydro-1 H-indene-5-carbonyl)benzoate (66)

To a solution of **60** (4.8058 g, 25.5 mmols) and methyl 4-(chlorocarbonyl)benzoate **64** (3.214 g, 16.18 mmols) in dichloromethane (35 mL) in a 100 mL round bottom flask was slowly added aluminum chloride (5.54 g) and the resulting mixture was stirred in an oil bath at reflux for 15 min. The reaction solution was cooled to room temperature and quenched by pouring onto 100 mL of an ice water solution. The solution was extracted with ethyl acetate, and the combined organic layers were dried over sodium sulfate, filtered, and concentrated to give a crude product that was purified by column chromatography (150 mL silica gel; 2.5% ethyl acetate:hexanes) to give pure **66** (4.4007 g, 77.6%) as a white, crystalline solid (120.2–122.2 °C): ^1^H NMR (400 MHz, CDCl_3_) δ 8.11 (dd, J = 6.8, 2.0, 2H), 7.86 (dd, J = 6.8, 2.0, 2H), 7.05 (s, 1H), 7.03 (s, 1H), 3.95 (s, 3H), 2.35 (s, 3H), 1.94 (s, 2H), 1.34 (s, 6H), 1.26 (s, 6H); ^13^C NMR (100.6 MHz, CDCl_3_) δ198.1, 166.3, 154.5, 148.4, 141.8, 136.3, 136.2, 133.4, 129.9, 129.5, 125.3, 123.7, 56.5, 52.4, 42.6, 42.2, 31.3, 31.2, 20.3. ES-MS (M + H)+ calcd for C_23_H_27_O_3_ 351.1960, found 351.1959.

### 6.35. Methyl 4-(1-(1,1,3,3,6-pentamethyl-2,3-dihydro-1 H-inden-5-yl)vinyl)benzoate (67)

To a solution of diisopropylamine (0.66 mL, 4.71 mmols) in THF (2 mL) in a 100 mL round bottom flask was added a 1.6 M solution of n-butyl lithium in hexanes (2.7 mL, 4.32 mmols) at room temperature with stirring. After 15 min of stirring, methyltriphenylphosphonium bromide (1.15 g, 3.22 mmol) was added and the heterogeneous solution was stirred for 20 min after which time the solution became homogeneous and bright canary yellow. This yellow ylide solution was added to a solution of **66** (0.7867 g, 2.24 mmol) in THF (4 mL), and the resulting reaction solution was stirred for 1 h and then poured into water (50 mL) and extracted with ethyl acetate. The combined organic extracts were washed with water, then brine, dried over sodium sulfate, filtered, and concentrated to give a crude oil that was purified by column chromatography (150 mL silica gel; 2.5% ethyl acetate:hexanes to 5% ethyl acetate:hexanes) to give pure **67** (0.3272 g, 41.9%) as a white, crystalline solid (120.2–122.2 °C): ^1^H NMR (400 MHz, CDCl_3_) δ 7.96 (d, J = 8.0, 2H), 7.35 (d, J = 8.8, 2H), 6.95 (s, 1H), 6.91 (s, 1H), 5.83 (d, J = 1.6, 1H), 5.33 (d, J = 1.2, 1H), 3.91 (s, 3H), 1.99 (s, 3H), 1.94 (s, 2H), 1.34 (s, 6H), 1.31 (s, 6H); ^13^C NMR (100.6 MHz, CDCl_3_) δ166.9, 150.9, 149.4, 148.9, 145.5, 139.3, 134.2, 129.6, 128.9, 126.5, 123.9, 116.8, 56.8, 52.0, 42.4, 42.3, 31.6, 31.5, 20.2. GC-MS CI (M)+ calcd for C_24_H_28_O_2_ 348.2089, found 348.2082.

### 6.36. Methyl 4-(1-(1,1,3,3,6-pentamethyl-2,3-dihydro-1 H-inden-5-yl)vinyl)benzoate (26)

To a 100 mL round bottom flask charged with **67** (0.4262 g, 1.223 mmols) and a small magnetic Teflon stir bar was added methanol (5.0 mL), followed by a solution of potassium hydroxide (0.2242 g, 3.996 mmols) in water (0.30 mL). A reflux condenser was fitted to the flask, and the solution was refluxed in an oil bath at 85 °C for 1.3 h. After cooling to room temperature, 1 N HCl (84 mL) was poured into the reaction, and the crude precipitate was filtered and dried to give crude **26** (0.3713 g, 90.7%) which was dissolved in a minimum of warm ethyl acetate and purified by column chromatography (150 mL silica, 50% ethyl actate:hexanes) to give pure **26** (0.2174, 52.4%) as a white, crystalline solid (197.0–198.8 °C): ^1^H NMR (400 MHz, CDCl_3_) δ 8.04 (dd, J = 8.8, 2.0, 2H), 7.38 (d, J = 8.8, 2H), 6.95 (s, 1H), 6.92 (s, 1H), 5.86 (d, J = 1.6, 1H), 5.36 (d, J = 0.8, 1H), 2.00 (s, 3H), 1.94 (s, 2H), 1.34 (s, 6H), 1.32 (s, 6H); ^13^C NMR (100.6 MHz, CDCl_3_) δ171.8, 150.9, 149.2, 148.9, 146.4, 139.2, 134.2, 130.3, 128.0, 126.6, 124.0, 117.2, 56.8, 42.4, 42.3, 31.6, 31.5, 20.2. ES-MS- (M-H)- calcd for C_23_H_25_O_2_ 333.1855, found 333.1867.

### 6.37. Methyl 2-(1-(1,1,3,3,6-pentamethyl-2,3-dihydro-1H-inden-5-yl)vinyl)pyrimidine-5-carboxylate (68)

A solution of **65** (2.18 g, 6.18 mmols) in toluene (22.0 mL) in a 100 mL round bottom flask was cooled to −10 °C under nitrogen with stirring and a 3.0 M solution methyl magnesium chloride (2.72 mL, 8.16 mmols) was added dropwise. After 15 min of stirring, the reaction solution was warmed to room temperature and stirred for an additional 35 min. The reaction mixture was then quenched by the slow addition of 1.0 N hydrochloric acid (14.0 mL, 14.0 mmols). The mixture was extracted with ethyl acetate, and the organic layers were washed with water and saturated sodium chloride, then dried over sodium sulfate, filtered, and concentrated in a 300 mL round bottom flask to give a crude alcohol product that was used without further purification. The alcohol product was dissolved in toluene (98.0 mL) and p-TsOH·H_2_O (1.197 g) was added, and the reaction flask was fitted with a Dean Stark trap and a water condenser. The vessel was evacuated and back-filled with nitrogen three times, and then heated to reflux in an oil bath at 130 °C and stirred for 3 h, during which time water collected in the Dean Stark trap. The reaction was cooled to room temperature, poured into water, and extracted with ethyl acetate. The combined organic layers were dried over sodium sulfate, filtered, and concentrated to give a crude product that was purified by column chromatography (silica gel; 2.5% ethyl acetate: hexanes to 5% ethyl acetate:hexanes) to give pure **68** (0.7969 g, 36.8%) as a white solid (182.9–185.5 °C): ^1^H NMR (400 MHz, CDCl_3_) δ 9.25 (s, 2H), 6.98 (s, 1H), 6.94 (s, 1H), 6.84 (d, J = 2.0, 1H), 5.81 (d, J = 2.0, 1H), 3.96 (s, 3H), 2.02 (s, 3H), 1.92 (s, 2H), 1.33 (s, 6H), 1.31 (s, 6H); ^13^C NMR (100.6 MHz, CDCl_3_) δ168.7, 164.3, 158.2, 150.9, 148.8, 148.7, 137.6, 134.2, 126.6, 123.9, 123.8, 121.1, 56.9, 52.5, 42.4, 42.3, 31.6, 31.5, 20.3; IR (neat) 2953, 1722.25 cm*^−^*^1^. ES-MS (M + H)+ calcd for C_22_H_27_N_2_O_2_ 351.2072, found 351.2068.

### 6.38. 2-(1-(1,1,3,3,6-pentamethyl-2,3-dihydro-1H-inden-5-yl)vinyl)pyrimidine-5-carboxylic Acid (32)

To a solution of **68** (0.6637 g, 1.8938 mmols) in methanol (12.0 mL) in a 100 mL round bottom flask was added a solution of potassium hydroxide (0.3032 g, 5.40 mmols) in water (0.45 mL). The resulting reaction solution was refluxed with stirring for 1 h in an oil bath at 85 °C. After cooling the reaction solution to room temperature, 1 N HCl (90 mL) was added. The resulting precipitate was filtered and washed with cold water and dried to give crude **32** (0.6143 g, 96.4%). The crude **32** was dissolved in hot ethyl acetate (16.0 mL), hexanes (51 mL) were added, and the homogenous solution was concentrated, filtered, and washed with hexanes to give pure **32** (0.3695 g, 58%) as a white solid (182.7–188.2 °C): ^1^H NMR (400 MHz, CDCl_3_) δ 9.31 (s, 2H), 6.98 (s, 1H), 6.95 (s, 1H), 6.87 (d, J = 2.0, 1H), 5.87 (d, J = 1.6, 1H), 2.03 (s, 3H), 1.91 (s, 2H), 1.30 (s, 12H); ^13^C NMR (100.6 MHz, CDCl_3_) δ 169.0, 168.0, 158.8, 151.1, 148.9, 148.3, 137.2, 134.2, 127.5, 124.0, 123.9, 120.7, 56.8, 42.4, 42.3, 31.5, 31.4, 20.3; IR (neat) 2954, 1715 cm*^−^*^1^. ES-MS (M + H)+ calcd for C_21_H_25_N_2_O_2_ 337.1916, found 337.1913.

### 6.39. Methyl 2-(1-(1,1,3,3,6-pentamethyl-2,3-dihydro-1H-inden-5-yl)cyclopropyl)pyrimidine-5-carboxylate (69)

To a suspension of trimethylsulfoxonium iodide (0.760 g, 3.45 mmols) in DMSO (2.5 mL) in a 50 mL 2-neck round bottom flask was added a 20 wt% solution of potassium tert-butoxide in THF (1.94 mL, 3.45 mmols) with stirring at 35 °C. The reaction mixture was stirred for 5 min and then a solution of **68** (0.8061 g, 2.30 mmols) in THF (9.9 mL) was added. The reaction was stirred for 1 h at 35 °C, then allowed to cool to room temperature, at which point 1 N hydrochloric acid (10.0 mL) was added. The resulting solution was extracted with ethyl acetate, the combined organic layers were washed with saturated sodium chloride, dried over sodium sulfate, filtered, and concentrated to give a crude off-white solid that was purified by column chromatography (silica gel; 2.5% ethyl acetate:hexanes to 10% ethyl acetate: hexanes) to give **69** (0.6009 g, 71.7%) as a white solid (236.4–242.4 °C): ^1^H NMR (400 MHz, CDCl_3_) δ 9.08 (s, 2H), 7.06 (s, 1H), 6.92 (s, 1H), 3.92 (s, 3H), 2.16 (s, 3H), 1.91 (s, 2H), 1.90 (t, J = 2.8, 2H), 1.49 (t, J = 3.2, 2H), 1.32 (s, 6H), 1.29 (s, 6H); ^13^C NMR (100.6 MHz, CDCl_3_) δ 176.8, 164.6, 157.8, 149.8, 148.5, 138.2, 137.3, 124.5, 123.8, 119.9, 56.9, 52.3, 42.3, 42.2, 32.0, 31.6, 31.5, 23.8, 21.8, 19.8; IR (neat) 2963, 1680 cm*^−^*^1^. ES-MS (M + H)+ calcd for C_23_H_29_N_2_O_2_ 365.2229, found 365.2213.

### 6.40. 2-(1-(1,1,3,3,6-pentamethyl-2,3-dihydro-1H-inden-5-yl)cyclopropyl)pyrimidine-5-carboxylic Acid (33)

To a solution of **69** (0.5324 g, 1.4607 mmols) in methanol (9.4 mL) in a 100 mL round bottom flask was added a solution of potassium hydroxide (0.2492 g, 4.44 mmols) in water (0.34 mL). The resulting reaction solution was refluxed with stirring for 1 h in an oil bath at 85 °C. After cooling the reaction solution to room temperature, 1 N HCl (90 mL) was added. The resulting precipitate was filtered and washed with cold water and dried to give crude **33** (0.4932 g, 96.3%). The crude **33** was dissolved in hot ethyl acetate (28.0 mL), hexanes (20 mL) were added, and the homogenous solution was concentrated, filtered, and washed with hexanes to give pure **33** (0.3402 g, 66%) as a white solid (261.6–267.3 °C): ^1^H NMR (400 MHz, CDCl_3_) δ 9.12 (s, 2H), 7.05 (s, 1H), 6.92 (s, 1H), 2.17 (s, 3H), 1.93 (m, 2H), 1.89 (s, 2H), 1.53 (m, 2H), 1.29 (s, 6H), 1.28 (s, 6H); ^13^C NMR (100.6 MHz, CDCl_3_) δ 177.1, 167.3, 159.0, 158.5, 158.4, 150.4, 148.9, 137.3, 124.7, 124.1, 119.7, 56.8, 42.4, 42.3, 32.0, 31.6, 31.5, 31.4, 23.9, 22.8, 19.8; IR (neat) 2954, 1721 cm*^−^*^1^. ES-MS (M + H)+ calcd for C_22_H_27_N_2_O_2_ 351.2072, found 351.2067.

### 6.41. 1,1,4,4-. tetramethyl-1,2,3,4-tetrahydronaphthalene (71)

The procedure of Bruson and Kroeger was followed. To a solution of 2,5-dichloro-2,5-dimethylhexane (**70**) (11.36 g, 62.04 mmols) in benzene (280 mL) was added aluminum chloride (1.5 g) in a 500 mL round bottom flask equipped with a stir bar and water condenser and the reaction was heated to 75–82 °C for 24 h with stirring under nitrogen. After cooling to room temperature, the reaction solution was poured into 1 N HCl (450 mL) and extracted with benzene. The combined organic layers were washed with water, saturated sodium bicarbonate, water, and finally saturated sodium chloride. The combined organic layers were dried over sodium sulfate, concentrated to a crude oil that was then vacuum distilled with a short-path distillation head at an oil bath temperature of 95–100 °C, and a head temperature of 78 °C for the major fraction, at 0.2–0.3 mm Hg to give pure **71** (7.5693g, 65%) as a colorless oil: ^1^H NMR (400 MHz, CDCl_3_) δ 7.35 (m, 2H), 7.16 (m, 2H), 1.72 (s, 4H), 1.32 (s, 12H); ^13^C NMR (100.6 MHz, CDCl_3_) δ 144.7, 126.4, 125.5, 35.0, 34.1, 31.8; IR (neat) 3021, 1715 cm*^−^*^1^.

### 6.42. Methyl 2-(5,5,8,8-tetramethyl-5,6,7,8-tetrahydronaphthalene-2-carbonyl)pyrimidine-5-carboxylate (73)

To a solution of **71** (5.3945 g, 28.646 mmols) and **63** (5.37 g, 26.772 mmols) in dichloromethane (60 mL) was slowly added aluminum chloride (8.8 g) and the resulting mixture was stirred at reflux in an oil bath at 55 °C for 15 min. The solution was then cooled to room temperature and poured into 200 mL of an ice water solution. The resulting mixture was extracted with ethyl acetate. The combined organic layers were washed with saturated sodium chloride, dried over sodium sulfate, filtered, and concentrated to give a crude product that was purified by column chromatography (silica gel, 15% ethyl acetate:hexanes to 20% ethyl acetate: hexanes) to give pure **73** (5.9627 g, 77%) as a bright canary yellow crystalline solid (79.1–83.4 °C): ^1^H NMR (400 MHz, CDCl_3_) δ 9.43 (s, 2H), 8.02 (d, J = 1.6, 1H), 7.63 (dd, J = 7.6, 2.0, 1H), 7.39 (d, J = 8.0, 1H), 4.03 (s, 3H), 1.70 (s, 4H), 1.29 (s, 12H); ^13^C NMR (100.6 MHz, CDCl_3_) δ 190.3, 165.3, 163.5, 158.3, 152.1, 145.5, 131.9, 129.3, 128.0, 126.7, 124.1, 52.9, 34.8, 34.7, 34.6, 34.4, 31.7, 31.5; IR (neat) 2954, 1721 cm*^−^*^1^. ES-MS (M + H)+ calcd for C_21_H_25_N_2_O_3_ 353.1865, found 353.1864.

### 6.43. Methyl 4-(5,5,8,8-tetramethyl-5,6,7,8-tetrahydronaphthalene-2-carbonyl)benzoate (74)

To a 250 mL flask, 4-(methoxycarbonyl)benzoic acid (3.535 g, 19.62 mmols) was treated with thionyl chloride (30.0 mL), resulting in a quantitative crude yield of methyl 4-(chlorocarbonyl)-benzoate (**64**) after the removal of thionyl chloride. Acid chloride **64** and compound **71** (4.0404 g, 21.45) were dissolved in 30 mL of DCM and aluminum chloride (4.18 g, 31.34 mmols) was slowly added followed by heating in an oil bath at 55 °C for 15 min. The reaction was quenched in 100 mL of ice water, the product was extracted with ethyl acetate, dried over sodium sulfate, and purified by column chromatography (300 mL silica gel, 2.5% ethyl acetate:hexanes to 20% ethyl acetate: hexanes) to give pure **74** (5.3353 g, 77.6%) as an off-white crystalline solid, mp 136.9–139.8 °C: 1 H NMR (400 MHz, CDCl3) δ 8.16 (dd, J = 6.8, 2.0, 2H), 7.83 (dd, J = 6.8, 2.0, 2H), 7.78 (d, J = 2.0, 1H), 7.53 (dd, J = 8.4, 2.0, 1H), 7.40 (d, J = 8.4, 1H), 3.96 (s, 3H), 1.72 (s, 4H), 1.31 (s, 6H), 1.29 (s, 6H); 13C NMR (100.6 MHz, CDCl3) δ 195.9, 166.4, 150.7, 145.2, 141.8, 134.1, 132.8, 129.6, 129.3, 128.8, 127.3, 126.7, 52.4, 34.8, 34.7, 34.7, 34.4, 31.7, 31.6. ES-MS (M + H)+ calcd for C_23_H_27_O_3_ 351.1960, found 351.1978.

### 6.44. Methyl 2-fluoro-4-(5,5,8,8-tetramethyl-5,6,7,8-tetrahydronaphthalene-2-carbonyl)benzoate (75)

To a 250 mL flask, 2-fluoro-terephthalic acid 4-methyl ester (2.4067 g, 12.146 mmol) was treated with thionyl chloride (20.0 mL), resulting in a quantitative crude yield of methyl 4-(chlorocarbonyl)-2-fluorobenzoate (**72**). Acid chloride **72** and compound **71** (2.68 g, 14.243 mmol) were dissolved in 30 mL DCM and aluminum chloride (4.24 g) was slowly added, followed by heating in an oil bath at 55 °C for 15 min. The reaction was quenched in 100 mL of ice water, the product was extracted with ethyl acetate, dried over sodium sulfate, and purified by column chromatography (2.5% ethyl acetate/hexane; 7.5% ethyl acetate/hexane; 20% ethyl acetate/hexane) to give pure ketone **75** (4.3126 g, 96.3%) as white solid, mp 94.4–96.8 °C: 1 H NMR (400 MHz, CDCl3) δ 8.03 (t, J = 7.6, 1H), 7.77 (d, J = 2.0, 1H), 7.58 (dd, J = 8.0, 1.6, 1H), 7.53 (dd, J = 11.2, 1.6, 1H), 7.50 (d, J = 2.0, 1H), 7.41 (d, J = 8.0, 1H), 3.97 (s, 3H), 1.72 (s, 4H), 1.29 (s, 6H), 1.24 (s, 6H); 13C NMR (100.6 MHz, CDCl3) δ 194.3, 194.2, 164.3, 164.3, 162.7, 160.1, 151.1, 145.5, 143.6, 143.6, 133.5, 132.0, 128.8, 127.2, 126.8, 124.9, 124.9, 121.4, 121.3, 118.2, 118.0, 52.6, 34.8, 34.7, 34.6, 34.4, 31.7, 31.5. ES-MS (M + Na)+ calcd for C_23_H_25_FO_3_Na 391.1685, found 391.1671.

### 6.45. Methyl 4-(1-(5,5,8,8-tetramethyl-5,6,7,8-tetrahydronaphthalen-2-yl)vinyl)benzoate (76)

To a 250 mL flask, ketone **74** (0.750 g, 2.14 mmol) was combined with dry THF (7.0 mL) at room temperature and a triphenylphosphonium methylide solution—made from the addition of methyltriphenylphosphonium bromide (1.15 g, 3.22 mmol) to a solution of n-butyl lithium (2.7 mL, 1.6 M in hexanes, 4.32 mmol) and diisopropyl amine (0.66 mL, 4.67 mmol) in THF (5 mL)—was added with stirring. The reaction was stirred for thirty minutes, poured into water, and extracted with ethyl acetate. The organic extracts were washed with water, dried over sodium sulfate, and purified by column chromatography (150 mL SiO_2_) with 2.5% ethyl acetate/hexanes; 5% ethyl acetate/hexane; 20% ethyl acetate/hexane to give pure **76** (0.4293 g, 57.6%) as a colorless, waxy solid, mp 112.1−113.9 °C: 1 H NMR (400 MHz, CDCl3) δ 8.02 (dd, J = 8.4, 2.0, 2H), 7.44 (dd, J = 8.4, 2.0, 2H), 7.27 (d, J = 8.4, 1H), 7.24 (d, J = 2.0, 1H), 7.08 (dd, J = 8.0, 2.0, 1H), 5.54 (d, J = 1.2, 1H), 5.48 (d, J = 1.2, 1H), 3.93 (s, 3H), 1.70 (s, 4H), 1.30 (s, 6H), 1.24 (s, 6H); 13C NMR (100.6 MHz, CDCl3) δ 166.9, 149.3, 146.3, 144.8, 144.6, 137.5, 129.3, 129.1, 128.2, 126.4, 126.3, 125.3, 114.9, 52.0, 35.0, 34.9, 34.2, 34.1, 31.7. ES-MS (M + H)+ calcd for C_24_H_29_O_2_ 349.2168, found 349.2168.

### 6.46. 4-(1-(5,5,8,8-tetramethyl-5,6,7,8-tetrahydronaphthalen-2-yl)vinyl)benzoic acid (27)

Compound **27** was synthesized according to the method of Boehm and co-workers. Compound **76** (1.5597 g, 4.476 mmols) was suspended in methanol (28.0 mL) and a solution of potassium hydroxide (0.7420 g, 13.22 mmols) in water (1.08 mL) was added. The reaction was refluxed in an oil bath at 85 °C. After 70 min of reflux, the solution was cooled to room temperature and the crude product was precipitated with 1 N hydrochloric acid (200 mL) and filtered to give (1.4655 g, 97.9%) of crude **27**. It was then purified by column chromatography (150 mL SiO_2_, with 2.5% ethyl acetate/hexanes; 5% ethyl acetate/hexane; 20% ethyl acetate/hexane) to give pure **27** (1.3152 g, 87.9%) as tint yellow solid, mp 211.3−215.8 °C: 1 H NMR (400 MHz, CDCl3) δ 10.33 (br s, 1H), 8.10 (dd, J = 6.8, 2.0, 2H), 7.48 (dd, J = 6.8, 2.0, 2H), 7.29 (d, J = 8.4, 1H), 7.25 (d, J = 2.0, 1H), 7.08 (dd, J = 8.4, 2.0, 1H), 5.57 (d, J = 0.8, 1H), 5.51 (d, J = 1.2, 1H), 1.70 (s, 4H), 1.31 (s, 6H), 1.25 (s, 6H); 13C NMR (100.6 MHz, CDCl3) δ 172.1, 149.3, 147.2, 144.9, 144.7, 137.4, 130.0, 128.4, 128.3, 126.4, 126.3, 125.3, 115.2, 35.1, 35.0, 34.2, 34.1, 31.8. ES-MS- (M-H)- calcd for C_23_H_25_O_2_ 333.1855, found 333.1872.

### 6.47. Methyl 2-(1-(5,5,8,8-tetrahydronaphthalen-2-yl)vinyl)pyrimidine-5-carboxylate (77)

A solution of **73** (5.2603 g, 14.926 mmols) in toluene (53.0 mL) in a 250 mL round bottom flask was cooled to −10 °C under nitrogen with stirring and a 3.0 M solution methyl magnesium chloride (6.56 mL, 19.68 mmols) was added dropwise. After 15 min of stirring, the reaction solution was warmed to room temperature and stirred for an additional 35 min. The reaction mixture was then quenched by the slow addition of 1.0 N hydrochloric acid (35.0 mL, 35.0 mmols). The mixture was extracted with ethyl acetate, and the organic layers were washed with water and saturated sodium chloride, then dried over sodium sulfate, filtered, and concentrated in a 300 mL round bottom flask to give a crude alcohol product that was used without further purification. The alcohol product was dissolved in toluene (110.0 mL) and p-TsOH·H_2_O (5.7782 g, 33.56 mmol) was added, and the reaction flask was fitted with a Dean Stark trap and a water condenser. The vessel was evacuated and back-filled with nitrogen three times, and then heated to reflux in an oil bath at 130 °C and stirred for 3 h, during which time water collected in the Dean Stark trap. The reaction was cooled to room temperature, poured into water, and extracted with ethyl acetate. The combined organic layers were dried over sodium sulfate, filtered, and concentrated to give a crude product that was purified by column chromatography (silica gel; 2.5% ethyl acetate:hexanes to 5% ethyl acetate:hexanes) to give pure **77** (0.2936 g, 5.6%) as a white solid (171.3–174.1 °C): ^1^H NMR (400 MHz, CDCl_3_) δ 9.28 (s, 2H), 7.35 (d, J = 2.0, 1H), 7.31 (d, J = 8.0, 1H), 7.17 (dd, J = 8.4, 2.0, 1H), 6.58 (d, J = 1.6, 1H), 5.93 (d, J = 1.6, 1H), 3.98 (s, 3H), 1.70 (s, 4H), 1.30 (s, 6H), 1.28 (s, 6H); ^13^C NMR (100.6 MHz, CDCl_3_) δ 168.9, 164.3, 158.0, 147.7, 144.7, 144.4, 135.5, 126.8, 126.2, 125.8, 124.4, 121.5, 52.5, 36.6, 35.1, 35.0, 34.2, 34.1, 31.8, 31.7, 24.6; IR (neat) 2952, 1721.95 cm*^−^*^1^. ES-MS (M + Na)+ calcd for C_22_H_26_N_2_O_2_Na 373.1892, found 373.1902.

### 6.48. 2-(1-(5,5,8,8-tetramethyl-5,6,7,8-tetrahydronaphthalen-2-yl)vinyl)pyrimidine-5-carboxylic acid (34)

To a solution of **77** (0.6585 g, 1.879 mmols) in methanol (12.0 mL) in a 100 mL round bottom flask was added a solution of potassium hydroxide (0.3046 g, 5.43 mmols) in water (0.45 mL). The resulting reaction solution was refluxed with stirring for 1 h in an oil bath at 85 °C. After cooling the reaction solution to room temperature, 1 N HCl (90 mL) was added. The resulting precipitate was filtered and washed with cold water and dried to give crude **34** (0.5526 g, 87%). The crude **34** was dissolved in hot ethyl acetate (17.0 mL), hexanes (50 mL) were added, and the homogenous solution was concentrated, filtered, and washed with hexanes to give pure **34** (0.2572 g, 40%) as a white solid (224.0–227.8 °C): ^1^H NMR (400 MHz, CDCl_3_) δ 9.35 (s, 2H), 7.35 (d, J = 1.6, 1H), 7.32 (d, J = 8.0, 1H), 7.17 (dd, J = 8.4, 2.0, 1H), 6.62 (d, J = 1.2, 1H), 5.98 (d, J = 1.6, 1H), 1.69 (s, 4H), 1.29 (s, 6H), 1.28 (s, 6H); ^13^C NMR (100.6 MHz, CDCl_3_) δ 169.4, 158.7, 147.5, 144.9, 144.5, 135.3, 126.8, 126.3, 125.8, 125.2, 120.7, 35.1, 35.0, 34.2, 34.1, 31.8, 31.7; IR (neat) 3009, 1715.95 cm*^−^*^1^. ES-MS (M + Na)+ calcd for C_21_H_24_N_2_O_2_Na 359.1736, found 359.1720.

### 6.49. Methyl 2-(1-(5,5,8,8-tetramethyl-5,6,7,8-tetrahydronaphthalen-2-yl)cyclopropyl)pyrimidine-5-carboxylate (78)

To a suspension of trimethylsulfoxonium iodide (0.3204 g, 1.45 mmols) in DMSO (1.05 mL) in a 50 mL 2-neck round bottom flask was added a 20 wt% solution of potassium tert-butoxide in THF (0.84 mL, 1.49 mmols) with stirring at 35 °C. The reaction mixture was stirred for 5 min and then a solution of **78** (0.3408 g, 0.97 mmols) in THF (4.8 mL) was added. The reaction was stirred for 1 h at 35 °C, then allowed to cool to room temperature, at which point 1 N hydrochloric acid (5.0 mL) was added. The resulting solution was extracted with ethyl acetate, the combined organic layers were washed with saturated sodium chloride, dried over sodium sulfate, filtered, and concentrated to give a crude off-white solid that was purified by column chromatography (silica gel; 2.5% ethyl acetate:hexanes to 10% ethyl acetate:hexanes) to give **78** (0.1533 g, 43%) as a white solid (168.7–171.8 °C): ^1^H NMR (400 MHz, CDCl_3_) δ 9.08 (s, 2H), 7.30 (d, J = 1.6, 1H), 7.27 (d, J = 8.0, 1H), 7.15 (dd, J = 8.0, 2.0, 1H), 3.92 (s, 3H), 1.79 (m, 2H), 1.68 (s, 4H), 1.50 (m, 2H), 1.29 (s, 6H), 1.26 (s, 6H); ^13^C NMR (100.6 MHz, CDCl_3_) δ 177.0, 164.6, 157.7, 144.5, 143.1, 138.2, 129.5, 128.8, 127.6, 126.2, 120.4, 120.0, 115.2, 52.4, 36.5, 35.1, 35.0, 34.2, 34.0, 33.0, 31.8, 24.6, 20.2; IR (neat) 2954, 1721 cm*^−^*^1^. ES-MS (M + Na)+ calcd for C_23_H_28_N_2_O_2_Na 387.2048, found 387.2048.

### 6.50. 2-(1-(5,5,8,8-tetramethyl-5,6,7,8-tetrahydronaphthalen-2-yl)cyclopropyl)pyrimidine-5-carboxylic acid (35)

To a solution of **78** (0.1316 g, 0.361 mmols) in methanol (2.5 mL) in a 100 mL round bottom flask was added a solution of potassium hydroxide (0.0803 g, 1.43 mmols) in water (0.18 mL). The resulting reaction solution was refluxed with stirring for 1 h in an oil bath at 85 °C. After cooling the reaction solution to room temperature, 1 N HCl (70 mL) was added. The resulting precipitate was filtered and washed with cold water and dried to give crude **78** (0.1041 g, 82%). The crude **78** was dissolved in hot ethyl acetate (5.0 mL), and the homogenous solution was concentrated, filtered, and washed with hexanes to give pure **78** (0.0734 g, 58%) as a white solid (251.5–254.6 °C): ^1^H NMR (400 MHz, CDCl_3_) δ 9.13 (s, 2H), 7.30 (d, J = 1.6, 1H), 7.29 (d, J = 8.4, 1H), 7.15 (dd, J = 8.0, 2.0, 1H), 1.81 (m, 2H), 1.68 (s, 4H), 1.53 (m, 2H), 1.28 (s, 6H), 1.26 (s, 6H); ^13^C NMR (100.6 MHz, CDCl_3_) δ 177.6, 167.7, 158.8, 158.3, 144.7, 143.4, 137.6, 128.9, 127.8, 126.4, 119.4, 35.1, 35.0, 34.2, 34.0, 33.2, 31.9, 31.8, 20.9, 20.9; IR (neat) 2957, 1679.64 cm*^−^*^1^. ES-MS (M + Na)+ calcd for C_22_H_26_N_2_O_2_Na 373.1892, found 373.1898.

### 6.51. Methyl 2-fluoro-4-(1-(5,5,8,8-tetramethyl-5,6,7,8-tetrahydronaphthalen-2-yl)vinyl)benzoate (79)

To a 250 mL flask, ketone product **75** (0.7971 g, 2.16 mmol) was combined with dry THF (4.0 mL) at room temperature and a triphenylphosphonium methylide solution—made from the addition of methyltriphenylphosphonium bromide (1.15 g, 3.22 mmol) to a solution of n-butyl lithium (2.7 mL, 1.6 M in hexanes, 4.32 mmol) and diisopropyl amine (66 mL, 4.67 mmol) in THF (2 mL)—was added with stirring. The reaction was stirred for thirty minutes, poured into water, and extracted with ethyl acetate. The organic extracts were washed with water, dried over sodium sulfate, and purified by column chromatography (150 mL SiO_2_) with 2.5% ethyl acetate/hexanes to 5% ethyl acetate/hexane giving pure compound **79** (0.4454 g, 56%) as yellow solid, mp 80.8–96.5 °C: ^1^H NMR (400 MHz, CDCl3) δ 7.90 (t, J = 7.6, 2H), 7.28 (d, J = 8.0, 1H), 7.22 (dd, J = 8.0, 1.6, 1H), 7.21 (dd, J = 2.0, 1H), 7.14 (dd, J = 12.4, 1.2, 1H), 7.05 (dd, J = 8.4, 2.0, 1H), 5.54 (d, J = 0.8, 1H), 5.50 (d, J = 0.8, 1H), 1.69 (s, 4H), 1.30 (s, 6H), 1.24 (s, 6H); ^13^C NMR (100.6 MHz, CDCl3) δ 164.8, 164.7, 163.0, 160.4, 148.5, 148.4, 148.3, 148.2, 145.0, 144.8, 136.9, 131.8, 126.5, 126.2, 125.3, 123.7, 123.7, 117.3, 117.2, 116.7, 116.4, 115.7, 52.2, 35.0, 34.9, 34.2, 34.1, 31.7. ES-MS (M + Na)+ calcd for C_24_H_27_FO_2_Na 389.1893, found 389.1892.

### 6.52. 2-fluoro-4-(1-(5,5,8,8-tetramethyl-5,6,7,8-tetrahydronaphthalen-2-yl)vinyl)benzoic Acid (36)

Compound **36** was synthesized according to the method of Boehm and co-workers. Compound **79** (2.0486 g, 5.59 mmols) and methanol (36.0 mL) were added to a solution of potassium hydroxide (0.9348 g, 16.66 mmols) in water (1.34 mL). The reaction was refluxed, and after 70 min of reflux, the solution was cooled to room temperature and the crude product was precipitated with 1 N hydrochloric acid (560 mL) to give crude product (1.7523 g) **36**. It was then purified by column chromatography (150 mL SiO_2_) in 50% ethyl acetate/hexane to give pure **36** (1.5974 g, 81.0%) as white crystalline solid, mp 165.6–183.0 °C: 1 H NMR (400 MHz, CDCl3) δ 8.01 (t, J = 8.0, 2H), 7.29 (d, J = 8.4, 1H), 7.27 (dd, J = 8.4, 1.6, 1H), 7.23 (d, J = 2.0, 1H), 7.18 (dd, J = 12.0, 1.2, 1H), 7.06 (dd, J = 8.4, 2.0, 1H), 5.58 (d, J = 0.4, 1H), 5.54 (d, J = 0.4, 1H), 1.70 (s, 4H), 1.31 (s, 6H), 1.26 (s, 6H); 13C NMR (100.6 MHz, CDCl3) δ 169.5, 169.4, 163.8, 161.2, 149.7, 149.6, 148.2, 148.2, 145.1, 144.9, 136.8, 132.4, 126.5, 126.3, 125.3, 123.9, 123.8, 116.8, 116.6, 116.3, 116.2, 116.1, 35.0, 34.9, 34.2, 34.1, 31.8, 31.7. ES-MS (M + Na)+ calcd for C_23_H_25_FO_2_Na 375.1736, found 375.1753.

### 6.53. Dimethyl-2-hydroxyterepthalate (81)

To a solution of hydroxyl-terepthalic acid (**80**) (9.93 g, 54.5 mmols) in methanol (189 mL) in a 500 mL round bottom flask equipped with a magnetic stir bar and cooled to 0 °C in an ice bath was added thionyl chloride (14.5 mL, 200 mmols) dropwise with stirring. After addition, the flask was equipped with a reflux condenser, placed under a nitrogen atmosphere, and warmed to reflux in an oil bath set at 85 °C and boiled for 2.5 h. The solution was allowed to cool to room temperature, and most of the methanol was removed in vacuo. The crude product was dissolved in ethyl acetate, and the solvent was washed with water followed by brine and then dried over sodium sulfate. The solvent was filtered and the ethyl acetate was removed in vacuo to provide a crude product that was dissolved in warm ethyl acetate (20 mL) and purified by column chromatography (250 mL SiO_2_) with 10% ethyl acetate/hexanes to give **81** (10.24 g, 90%) as white solid, m.p. 92.2–94.8 °C: ^1^H NMR (400 MHz, CDCl3) δ 10.75 (s, 1H), 7.89 (d, J = 8.4, 1H), 7.62 (d, J = 1.6, 1H), 7.51 (dd, J = 8.4, 1.6, 1H), 3.97 (s, 3H), 3.92 (s, 3H); ^13^C NMR (100.6 MHz, CDCl3) δ 169.9, 165.9, 161.2, 136.3, 130.0, 119.6, 118.8, 115.6, 52.6, 52.4. ES-MS (M + Na)+ calcd for C_10_H_10_O_5_Na 233.0426, found 233.0416.

### 6.54. 3-Hydroxy-4-(methoxycarbonyl)benzoic Acid (82)

The method of Zhang and co-workers was followed. Sodium hydroxide (0.7966 g, 19.9 mmols) was dissolved in water (32 mL), the solution was cooled to 0 °C, and a finely ground powder of dimethyl-2-hydroxyterepthalate (**81**) (1.0119 g, 4.81 mmols) was added to the solution. The solution was stirred for 1.5 h at 0 °C, and then a solution of 1 N hydrochloric acid was added (12 mL, 12 mmol)m which brought the solution to pH = 9.0, and the insoluble precipitate was filtered off. To the filtrate, an additional amount of 1 N hydrochloric acid (9.5 mL, 9.5 mmol) was added that brought the pH = 1.0 and the resulting precipitate was filtered and washed with cold water to give a crude product (0.67 g) that was purified by column chromatography (150 mL SiO_2_) with 20% ethyl acetate/hexanes to 70% ethyl acetate/hexanes to give **82** (0.4606 g, 49%) as white solid, m.p. 213.7–216.2 °C: ^1^H NMR (400 MHz, CDCl3) δ 10.79 (s, 1H), 7.94 (d, J = 8.0, 1H), 7.71 (d, J = 1.6, 1H), 7.58 (dd, J = 8.0, 1.6, 1H), 3.99 (s, 3H). ES-MS- (M-H)- calcd for C_9_H_7_O_5_ 195.0293, found 195.0285.

### 6.55. 3-Acetoxy-4-(methoxycarbonyl)benzoic Acid (83)

To a solution of 3-hydroxy-4-(methoxycarbonyl)benzoic acid (**82**) (0.5328 g, 2.716 mmols) in acetic anhydride (20.0 mL) in a 100 mL round bottom flask equipped with a stir bar was added concentrated sulfuric acid (3 drops) and the reaction was stirred in an oil bath at 45 °C for 40 min. The acetic anhydride was removed in vacuo and the crude oil was purified by column chromatography (150 mL SiO_2_) with 10% ethyl acetate/hexanes to pure ethyl acetate to give **83** (0.5715 g, 88%) as white solid, m.p. 182.6–185.1 °C: ^1^H NMR (400 MHz, CDCl3) δ 8.10 (d, J = 8.4, 1H), 8.03 (dd, J = 8.4, 1.6, 1H), 7.84 (d, J = 1.6, 1H), 3.91 (s, 3H), 2.38 (s, 3H); ^13^C NMR (100.6 MHz, CDCl3) δ 169.8, 169.5, 164.1, 150.5, 134.0, 131.9, 127.8, 127.4, 125.6, 52.6, 20.9. ES-MS (M + Na)+ calcd for C_11_H_10_O_6_Na 261.0375, found 261.0366.

### 6.56. Methyl 2-hydroxy-4-(3,5,5,8,8-pentamethyl-5,6,7,8-tetrahydronaphthalene-2-carbonyl)benzoate (86)

To a 100 mL round bottom flask charged with 3-acetoxy-4-(methoxycarbonyl)benzoic acid (**83**) (3.236 g, 13.59 mmols) was added thionyl chloride (22 mL, 300 mmols) and a few drops of DMF. A water condenser was added to the flask, and the solution was refluxed in an oil bath for 1.2 h to give methyl 2-acetoxy-4-(chlorocarbonyl)benzoate (**84**) in quantitative yield after the excess thionyl chloride was removed in vacuo. To the 100 mL round bottom flask containing **84** was added 1,1,4,4,6-pentamethyl-1,2,3,4-tetrahydronaphthalene (**85**) (6.0508 g, 29.9 mmols) and DCM (30 mL). To the resulting homogeneous solution was slowly added aluminum chloride (3.0 g) at room temperature, with the observed evolution of gas, and the reaction was refluxed for 15 min at 55 °C in an oil bath. The reaction solution was cooled to 0 °C in an ice bath and poured onto 100 mL of an ice water solution. The layers were separated, and the aqueous layer was extracted with ethyl acetate. The combined organic layers were washed with water and then brine, dried over sodium sulfate, filtered, and concentrated in vacuo to give a crude product that was purified by column chromatography (250 mL SiO_2_) with 1.5% to 5% ethyl acetate/hexanes to give **86** (3.64 g, 70%) as a white solid, m.p. 104.2–106.3 °C: ^1^H NMR (400 MHz, CDCl3) δ 10.78 (s, 1H), 7.93 (d, J = 8.0, 1H), 7.33 (d, J = 1.2, 1H), 7.31 (dd, J = 8.0, 1.6, 1H), 7.27 (s, 1H), 7.18 (s, 1H), 3.99 (s, 3H), 2.32 (s, 3H), 1.68 (s, 4H), 1.30 (s, 6H), 1.20 (s, 6H); ^13^C NMR (100.6 MHz, CDCl3) δ 197.5, 170.0, 161.3, 148.3, 144.5, 141.8, 134.5, 134.4, 129.9, 129.3, 128.3, 119.8, 119.4, 115.2, 52.6, 34.9, 34.8, 34.3, 33.8, 31.7, 31.6, 20.0. ES-MS (M + Na)+ calcd for C_24_H_28_O_4_Na 403.1885, found 403.1888.

### 6.57. Methyl 2-hydroxy-4-(1-(3,5,5,8,8-pentamethyl-5,6,7,8-tetrahydronaphthalen-2-yl)vinyl)benzoate (88)

To a 100 mL round bottom flask containing a solution of diisopropylamine (5.67 mL, 40.5 mmols) in THF (16.8 mL) was added a 1.6 M solution of n-butyl lithium in hexanes (22.65 mL, 36.24 mmols) at room temperature, and the reaction was stirred for 15 min followed by the addition of methyl triphenylphosphonium bromide (9.7201 g, 27.21 mmols). After stirring this reaction for 1 h, the resulting solution was added to a 100 mL round bottom flask contain a solution of methyl 2-hydroxy-4-(3,5,5,8,8-pentamethyl-5,6,7,8-tetrahydronaphthalene-2-carbonyl)benzoate (**86**) (3.8134 g, 10.02 mmols) in THF (8.86 mL) and the resulting reaction solution was stirred for 1 h, poured into 1 N hydrochloric acid (150 mL, 150 mmols), and extracted with ethyl acetate. The combined organic layers were washed with water and brine, dried over sodium sulfate, filtered, and concentrated in vacuo to give a crude product that was purified by column chromatography (150 mL SiO_2_) with 1.5% to 5% ethyl acetate/hexanes to give a mixture of spots containing **88** and this mixture was again purified by column chromatography (250 mL SiO_2_) with 1% to 2% ethyl acetate/hexanes to give pure **88** (1.2997 g, 34%) as white solid, m.p. 103.6–106.6 °C: ^1^H NMR (400 MHz, CDCl3) δ 10.74 (s, 1H), 7.76 (d, J = 8.4, 1H), 7.11 (s, 1H), 7.06 (s, 1H), 6.88 (dd, J = 8.4, 1.6, 1H), 6.84 (d, J = 2.0, 1H), 5.81 (d, J = 1.2, 1H), 5.33 (d, J = 1.2, 1H), 3.94 (s, 3H), 1.96 (s, 3H), 1.69 (s, 4H), 1.30 (s, 6H), 1.27 (s, 6H). ES-MS (M + Na)+ calcd for C_25_H_30_O_3_Na 401.2093, found 401.2110.

### 6.58. 2-Hydroxy-4-(1-(3,5,5,8,8-pentamethyl-5,6,7,8-tetrahydronaphthalen-2-yl)vinyl)benzoic acid (37a)

To a 100 mL round bottom flask containing a suspension of methyl 2-hydroxy-4-(1-(3,5,5,8,8-pentamethyl-5,6,7,8-tetrahydronaphthalen-2-yl)vinyl)benzoate (**88**) (0.3027 g, 0.800 mmols) in methanol (4.0 mL) was added a solution of potassium hydroxide (0.1634 g, 2.9 mmols) in water (0.20 mL), and the flask was fitted with a condenser and refluxed in an oil bath set to 85 °C for 1.2 h. The solution was cooled to room temperature and acidified with 1 N hydrochloric acid (90 mL, 90 mmols), and the resulting precipitate was filtered and dried to give crude **37** (0.2380 g, 81.6%) as a white solid. This crude material was purified by column chromatography (25 mL SiO_2_) with 40% ethyl acetate/hexanes to pure ethyl acetate to 8% methanol/ethyl acetate to give pure **37a** (0.2316 g, 79%) as white solid, m.p. 220.4–224.9 °C: ^1^H NMR (400 MHz, CDCl3) δ 10.35 (br s, 1H), 7.85 (d, J = 8.4, 1H), 7.11 (s, 1H), 7.07 (s, 1H), 6.92 (dd, J = 8.4, 1.6, 1H), 6.86 (d, J = 1.6, 1H), 5.84 (d, J = 0.8, 1H), 5.36 (d, J = 1.2, 1H), 1.96 (s, 3H), 1.69 (s, 4H), 1.30 (s, 6H), 1.27 (s, 6H); ^13^C NMR (100.6 MHz, CDCl3) δ 174.5, 162.1, 149.8, 148.8, 144.4, 142.3, 137.5, 132.6, 130.7, 128.0, 128.0, 118.0, 117.9, 115.7, 109.9, 35.2, 35.1, 33.9, 33.8, 31.9, 31.8, 19.8. ES-MS (M + H)+ calcd for C_24_H_29_O_3_ 365.2117, found 365.2122.

### 6.59. Methyl 2-hydroxy-4-(5,5,8,8-tetramethyl-5,6,7,8-tetrahydronaphthalene-2-carbonyl)benzoate (89)

To a 100 mL round bottom flask charged with 3-acetoxy-4-(methoxycarbonyl)benzoic acid (**83**) (4.7646 g, 20.00 mmols) was added thionyl chloride (32 mL, 440 mmols) and a few drops of DMF. A water condenser was added to the flask, and the solution was refluxed in an oil bath for 1.2 h to give methyl 2-acetoxy-4-(chlorocarbonyl)benzoate (**84**) in quantitative yield after the excess thionyl chloride was removed in vacuo. To the 100 mL round bottom flask containing **84** was added 1,1,4,4-tetramethyl-1,2,3,4-tetrahydronaphthalene (**71**) (7.4885 g, 39.8 mmols) and DCM (45 mL). To the resulting homogeneous solution was slowly added aluminum chloride (6.7750 g) at room temperature, with the observed evolution of gas, and the reaction was refluxed for 15 min at 55 °C in an oil bath. The reaction solution was cooled to 0 °C in an ice bath and poured onto 100 mL of an ice water solution. The layers were separated, and the aqueous layer was extracted with ethyl acetate. The combined organic layers were washed with water and then brine, dried over sodium sulfate, filtered, and concentrated in vacuo to give a crude product that was purified by column chromatography (250 mL SiO_2_) with 1.5% to 5% ethyl acetate/hexanes to give **89** (3.5757 g, 48.8%) as white solid, m.p. 76.7–81.3 °C: ^1^H NMR (400 MHz, CDCl3) δ 10.82 (s, 1H), 7.95 (d, J = 8.4, 1H), 7.81 (d, J = 1.6, 1H), 7.53 (dd, J = 8.4, 1.6, 1H), 7.39 (d, J = 8.0, 1H), 7.32 (d, J = 1.2, 1H), 7.25 (dd, J = 8.0, 1.6, 1H), 3.95 (s, 3H), 1.71 (s, 4H), 1.31 (s, 6H), 1.29 (s, 6H); ^13^C NMR (100.6 MHz, CDCl3) δ 195.4, 170.0, 161.1, 150.8, 145.2, 144.5, 133.8, 129.8, 128.8, 127.4, 126.6, 119.8, 118.8, 114.6, 52.6, 34.8, 34.7, 34.6, 34.3, 31.7, 31.5; ES-MS (M + Na)+ calcd for C_23_H_26_O_4_Na 389.1729, found 389.1728.

### 6.60. Methyl 2-hydroxy-4-(1-(5,5,8,8-tetramethyl-5,6,7,8-tetrahydronaphthalen-2-yl)vinyl)benzoate (91)

To a 100 mL round bottom flask containing a solution of diisopropylamine (5.07 mL, 36.2 mmols) in THF (15.0 mL) was added a 1.6 M solution of n-butyl lithium in hexanes (20.25 mL, 32.40 mmols) at room temperature, and the reaction was stirred for 15 min followed by the addition of methyl triphenylphosphonium bromide (8.700 g, 24.35 mmols). After stirring this reaction for 1 h, the resulting solution was added to a 100 mL round bottom flask containing a solution of methyl 2-hydroxy-4-(5,5,8,8-tetramethyl-5,6,7,8-tetrahydronaphthalene-2-carbonyl)benzoate (**89**) (3.3653 g, 9.18 mmols) in THF (7.92 mL) and the resulting reaction solution was stirred for 1 h, poured into 1 N hydrochloric acid (180 mL, 180 mmols), and extracted with ethyl acetate. The combined organic layers were washed with water and brine, dried over sodium sulfate, filtered, and concentrated in vacuo to give a crude product that was purified by column chromatography (250 mL SiO_2_) with 1% to 2% ethyl acetate/hexanes to give pure **91** (1.7909 g, 53.5%) as a white solid, m.p. 104.7–112.3 °C: ^1^H NMR (400 MHz, CDCl3) δ 10.75 (s, 1H), 7.78 (d, J = 8.0, 1H), 7.26 (d, J = 8.0, 1H), 7.25 (dd, J = 7.2, 2.0), 7.05 (dd, J = 8.0, 2.0, 1H); 7.00 (d, J = 1.6, 1H), 6.88 (dd, J = 8.4, 1.6, 1H), 5.51 (d, J = 0.8, 1H), 5.49 (d, J = 1.2, 1H), 3.95 (s, 3H), 1.69 (s, 4H), 1.29 (s, 6H), 1.25 (s, 6H); ^13^C NMR (100.6 MHz, CDCl3) δ 170.4, 161.3, 149.2, 149.1, 144.7, 144.6, 137.3, 129.4, 126.3, 125.4, 119.4, 117.1, 115.3, 111.3, 52.2, 35.0, 34.9, 34.2, 34.1, 31.7; ES-MS (M + Na)+ calcd for C_24_H_28_O_3_Na 387.1936, found 387.1939.

### 6.61. 2-Hydroxy-4-(1-(5,5,8,8-tetramethyl-5,6,7,8-tetrahydronaphthalen-2-yl)vinyl)benzoic Acid (37b)

To a 100 mL round bottom flask containing a suspension of methyl 2-hydroxy-4-(1-(5,5,8,8-tetramethyl-5,6,7,8-tetrahydronaphthalen-2-yl)vinyl)benzoate (**91**) (1.6706 g, 4.58 mmols) in methanol (5.0 mL) was added a solution of potassium hydroxide (0.9389 g, 16.7 mmols) in water (1.16 mL), and the flask was fitted with a condenser and refluxed in an oil bath set to 85 °C for 1.5 h. The solution was cooled to room temperature and acidified with 1 N hydrochloric acid (85 mL, 85 mmols), and the resulting precipitate was filtered and dried to give crude **37b** (1.5797 g, 98.3%) as a white solid. This crude material was purified by column chromatography (150 mL SiO_2_) with 40% ethyl acetate/hexanes to pure ethyl acetate to give pure **37b** (1.1678 g, 72.7%) as a white solid, m.p. 183.9–191.5 °C: ^1^H NMR (400 MHz, CDCl3) δ 10.37 (br s, 1H), 7.88 (d, J = 8.4, 1H), 7.27 (d, J = 8.4, 1H), 7.25 (d, J = 2.4, 1H), 7.06 (dd, J = 8.4, 2.0, 1H), 7.03 (d, J = 1.2, 1H), 6.95 (dd, J = 8.4, 1.2, 1H), 5.54 (d, J = 5.6, 1H), 1.70 (s, 4H), 1.30 (s, 6H), 1.26 (s, 6H); ^13^C NMR (100.6 MHz, CDCl3) δ 174.8, 162.0, 150.6, 149.0, 144.8, 144.7, 137.1, 130.5, 126.4, 126.3, 125.4, 119.8, 117.3, 115.7, 110.2, 35.0, 34.9, 34.2, 34.1, 31.8; ES-MS (M + H)+ calcd for C_23_H_27_O_3_ 351.1960, found 351.1964.

## Figures and Tables

**Table 1:** Auto-Dock Vina scores, EC_50_ values, IC_50_ values, LXRE activities, LHS scores, RARE activities, and cytotoxicities for Bex and compounds 25–36, 37a, and 37b.

Compound	Auto-Dock Vina Scores (kcal/mol)	EC_50_ (nM) +/−(SD)	96 h IC_50_ (nM) + 100 nM ATRA +/−SD	LXRE Activity (% of Bex)	LHS Score (vs. Bex)	RARE Activity (%RA at 10 nM)	Cyto-Toxicity (50% Cell Death)
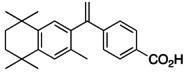 **1**	−12.7	18 (1)	8 (1)	100	1.0	28.1	0.5 µg/µL
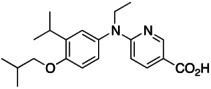 **25**	−9.3	274 (1)	>1000	72.4	4.0	2.9	none
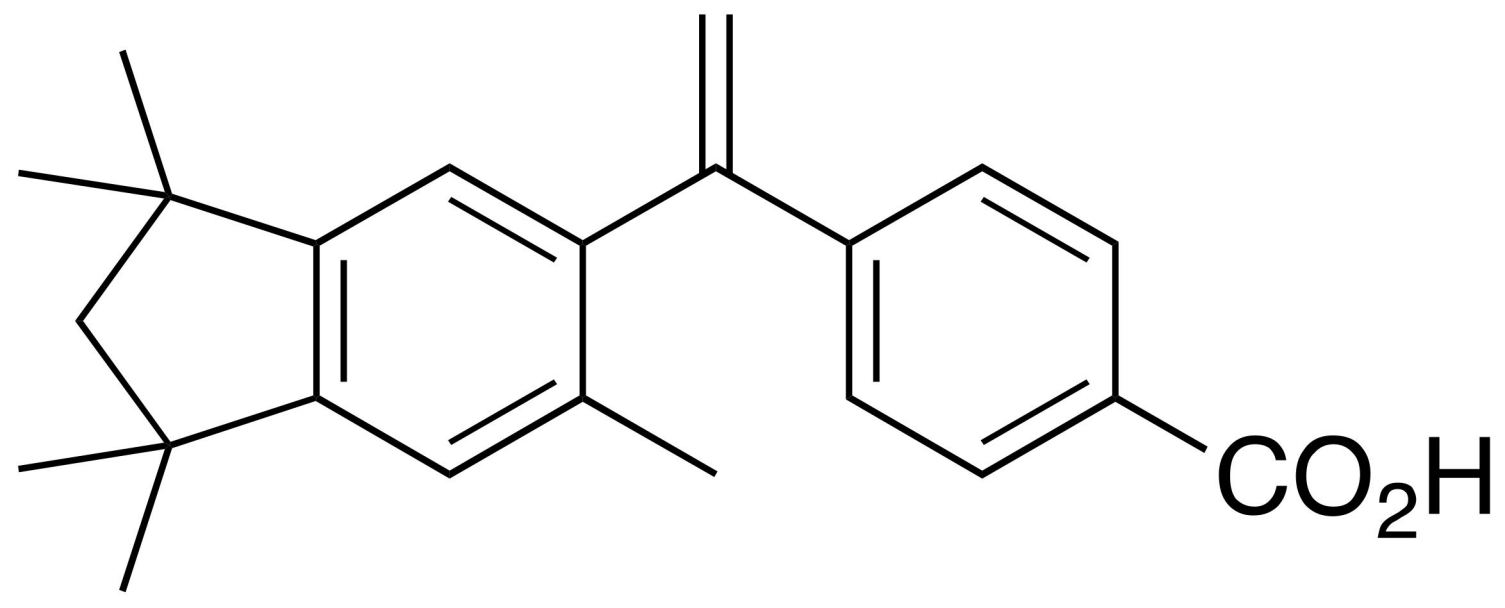 **26**	−12.3	70.1 (0.2)	85 (1)	94.5	0.66	3.0	none
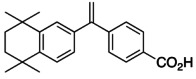 **27**	−12.0	171.2 (0.1)	114 (1)	82.4	1.0	1.5	0.5 µg/µL
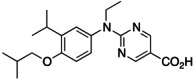 **28**	−8.6	297 (1)	>1000	77.7	4.2	1.6	1 µg/µL
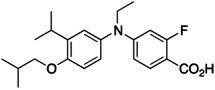 **29**	−10.0	>1000	>1000	61.7	6.4	4.0	1 µg/µL
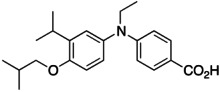 **30**	−9.3	397 (1)	>1000	60.2	5.3	1.1	0.08 µg/µL
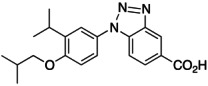 **31**	−9.3	>1000	>1000	64.5	5.4	3.1	none
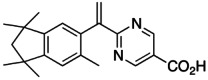 **32**	−7.9	34.4 (0.2)	54 (1)	46.0	0.38	12.9	none
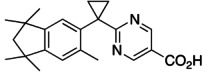 **33**	−11.6	17 (1)	2 (1)	50.9	0.38	5.9	none
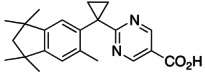 **34**	−8.6	56.2 (0.1)	43 (1)	77.6	0.70	10.3	none
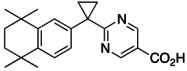 **35**	−11.5	1.3 (0.5)	2 (1)	60.6	1.2	5.1	none
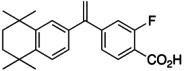 **36**	−12.4	375.2 (0.1)	376 (2)	85.2	1.6	1.4	0.08 µg/µL
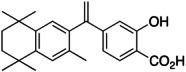 **37a**	−12.1	24.2 (0.2)	6 (1)	91.1	0.45	2.9	1 µg/µL
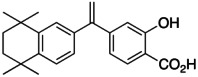 **37b**	−11.9	413.1 (0.1)	500 (10)	69.1	0.91	5.2	0.5 µg/µL

## Data Availability

CCDC numbers 2110149 and 2110150 contain the supplementary crystallographic data for this paper. These data can be obtained free of charge from The Cambridge Crystallographic Data Center via www.ccdc.cam.ac.uk/data_request/cif (accessed on 27 September 2021).

## Supplementary Materials

The following are available online at https://www.mdpi.com/article/10.3390/ijms222212371/s1.

## Abbreviations

RXR	Retinoid-X-receptor
RAR	Retinoic-acid-receptor
CTCL	Cutaneous T-cell lymphoma
RXRE	Retinoid-X-receptor element
HRE	Hormone responsive element
TR	Thyroid hormone receptor
VDR	Vitamin D receptor
SNuRMs	Specific nuclear receptor modulators
NR	Nuclear receptor
LXR	Liver-X-receptor
PPAR	Peroxisome proliferator activating receptor
LBD	Ligand binding domain
LHS	LXR heterodimer specificity
NaBu	Sodium butyrate
POC	Proof of concept
SREBP	Sterol regulatory element binding protein
LR	Lipid risk assessment index
