# Supplementary material for: Modeling, Synthesis, and Biological Evaluation of Potential Retinoid-X-Receptor (RXR) Selective Agonists: Analogs of 4-[1-(3,5,5,8,8-Pentamethyl-5,6,7,8-tetrahyro-2-naphthyl)ethynyl]benzoic Acid (Bexarotene) and 6-(Ethyl(4-isobutoxy-3-isopropylphenyl)amino)nicotinic Acid (NEt-4IB)
Source: Int J Mol Sci. 2021 Nov 16;22(22):12371. doi: 10.3390/ijms222212371 (PMC8624485; doi:10.3390/ijms222212371)
Supplement: Supplementary file 1 [file ijms-22-12371-s001.zip › ijms-1417084-supplementary.pdf]

Supporting Information for **Modeling, Synthesis and Biological Evaluation of Potential Retinoid-X-Receptor (RXR) Selective Agonists: Analogs of 4-[1-(3,5,5,8,8-Pentamethyl-5,6,7,8-tetrahydro-2-naphthyl)ethynyl]benzoic Acid (Bexarotene) and 6-(Ethyl(4-isobutoxy-3-isopropylphenyl)amino)nicotinic Acid (NEt-4IB)**:

^{1}H- and ^{13}C-NMR for all reported compounds

Representative HPLC Method for compound **33**

HPLC Traces for compounds **25-36**, **37a** and **37b**

CWV-195

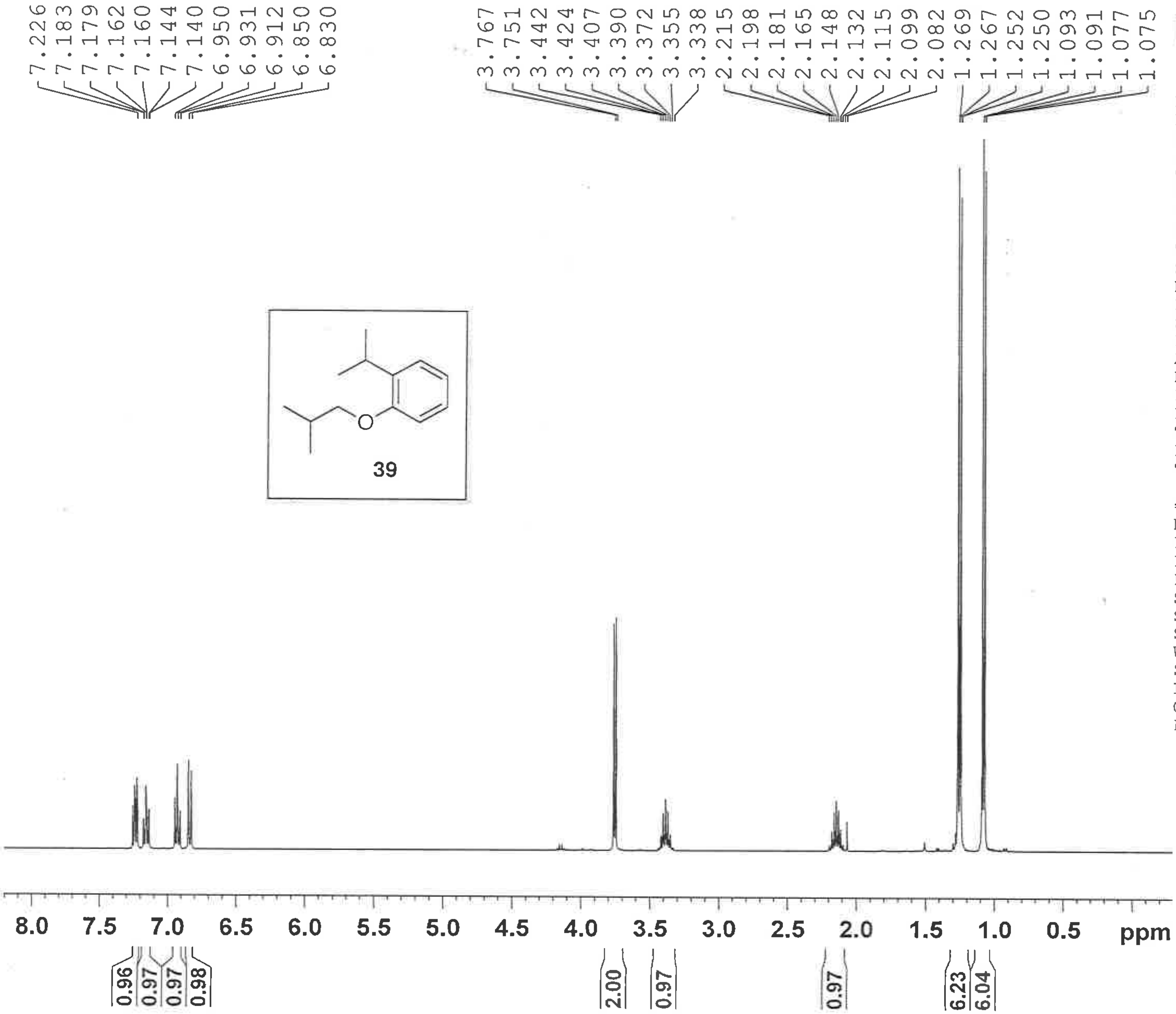

39

BRUKER

NAME	CWV-195
EXPNO	1
PROCNO	1
Date_	20141227
Time	15.46
INSTRUM	spect
PROBHD	5 mm PABBO BB-
PULPROG	zg30
TD	65536
SOLVENT	CDCl3
NS	16
DS	2
SWH	8223.685 Hz
FIDRES	0.125483 Hz
AQ	3.9846387 sec
RG	4
DW	60.800 usec
DE	6.50 usec
TE	298.2 K
D1	1.00000000 sec
TD0	1

======== CHANNEL f1 ========

NUC1	1H
P1	14.00 usec
PL1	0.50 dB
PL1W	12.76071072 W
SFO1	400.1324710 MHz
SI	32768
SF	400.1300096 MHz
WDW	EM
SSB	0
LB	0.30 Hz
GB	0
PC	1.00

CWV-195

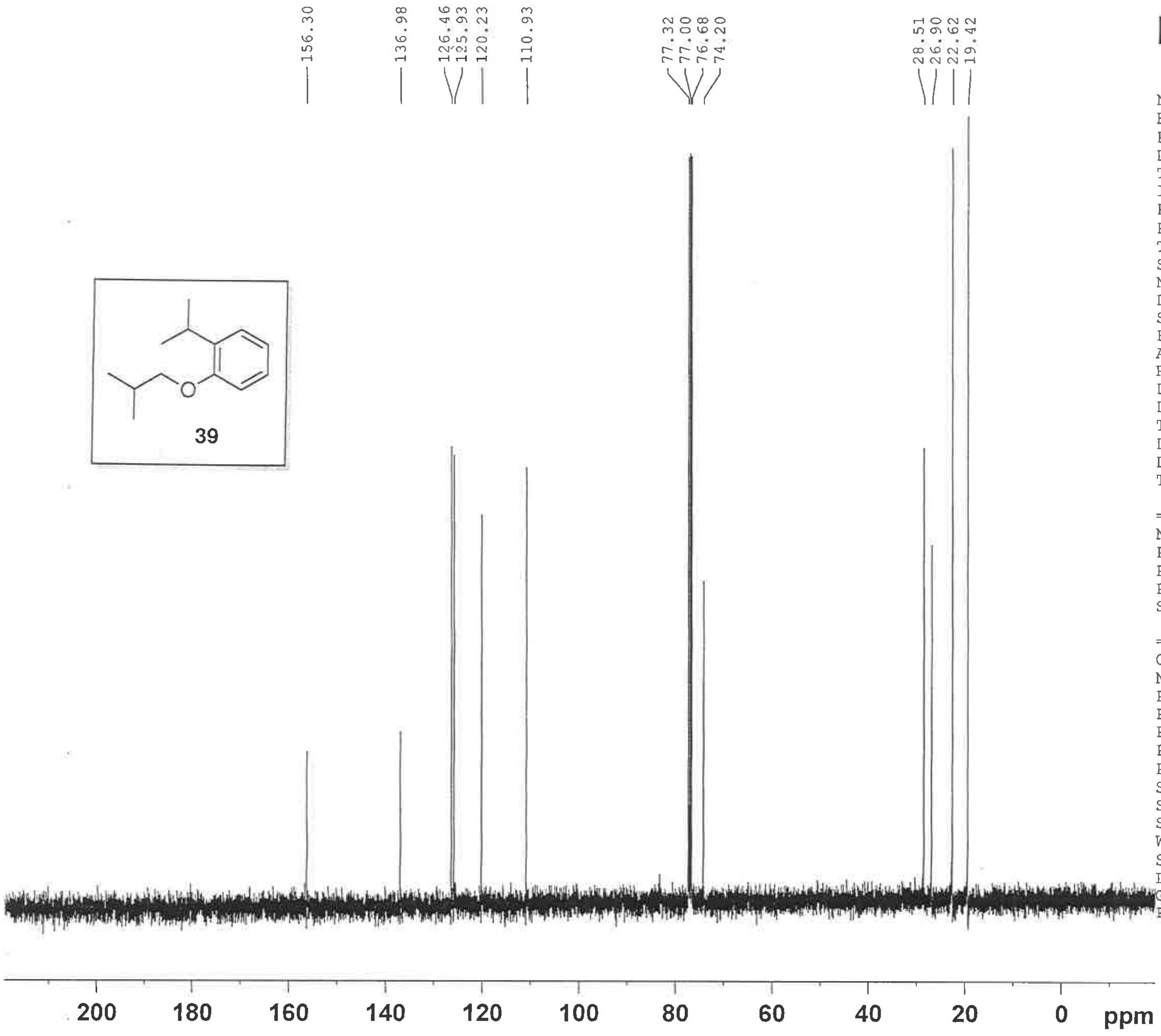

BRUKER

NAME	CWV-195
EXPNO	2
PROCNO	1
Date_	20141227
Time	15.53
INSTRUM	spect
PROBHD	5 mm PABBO BB-
PULPROG	zgig30
TD	65536
SOLVENT	CDCl3
NS	51
DS	4
SWH	24038.461 Hz
FIDRES	0.366798 Hz
AQ	1.3631988 sec
RG	2050
DW	20.800 usec
DE	6.50 usec
TE	298.2 K
D1	2.00000000 sec
D11	0.03000000 sec
TD0	1

======== CHANNEL f1 ========

NUC1	13C
P1	8.50 usec
PL1	-2.10 dB
PL1W	60.29227829 W
SFO1	100.6228298 MHz

======== CHANNEL f2 ========

CPDPRG2	waltz16
NUC2	1H
PCPD2	90.00 usec
PL2	-1.80 dB
PL12	17.28 dB
PL2W	21.67079544 W
PL12W	0.26783961 W
SFO2	400.1316005 MHz
SI	32768
SF	100.6127722 MHz
WDW	EM
SSB	0
LB	1.00 Hz
GB	0
PC	1.40

CWV-197 bottom spot

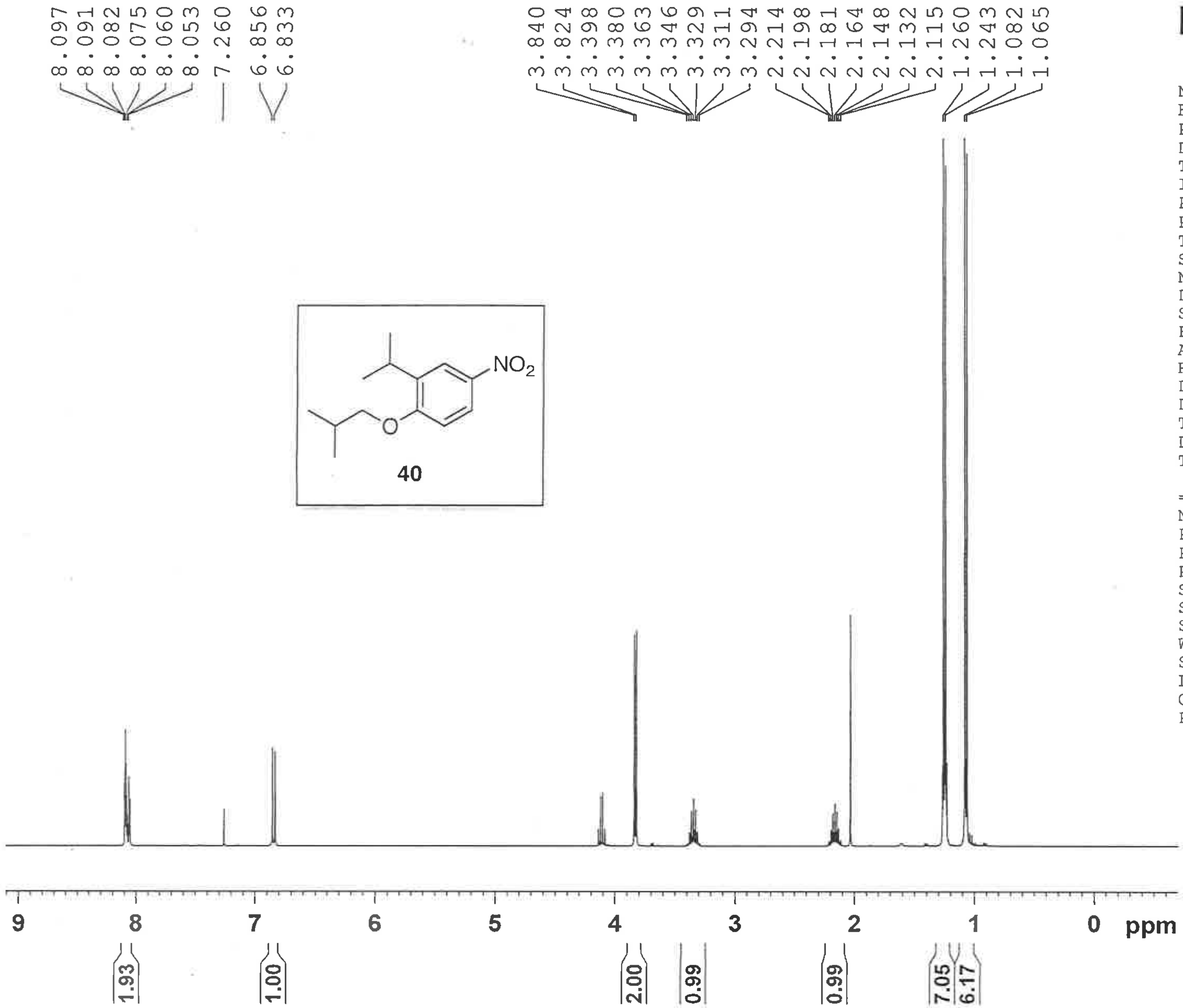

40

BRUKER

NAME	CWV-197
EXPNO	7
PROCNO	1
Date_	20150108
Time	9.47
INSTRUM	spect
PROBHD	5 mm PABBO BB-
PULPROG	zg30
TD	65536
SOLVENT	CDCl3
NS	16
DS	2
SWH	8223.685 Hz
FIDRES	0.125483 Hz
AQ	3.9846387 sec
RG	4
DW	60.800 usec
DE	6.50 usec
TE	298.2 K
D1	1.00000000 sec
TD0	1

======== CHANNEL f1 ========

NUC1	1H
P1	14.00 usec
PL1	0.50 dB
PL1W	12.76071072 W
SFO1	400.1324710 MHz
SI	32768
SF	400.1300099 MHz
WDW	EM
SSB	0
LB	0.30 Hz
GB	0
PC	1.00

CWV-197 bottom spot

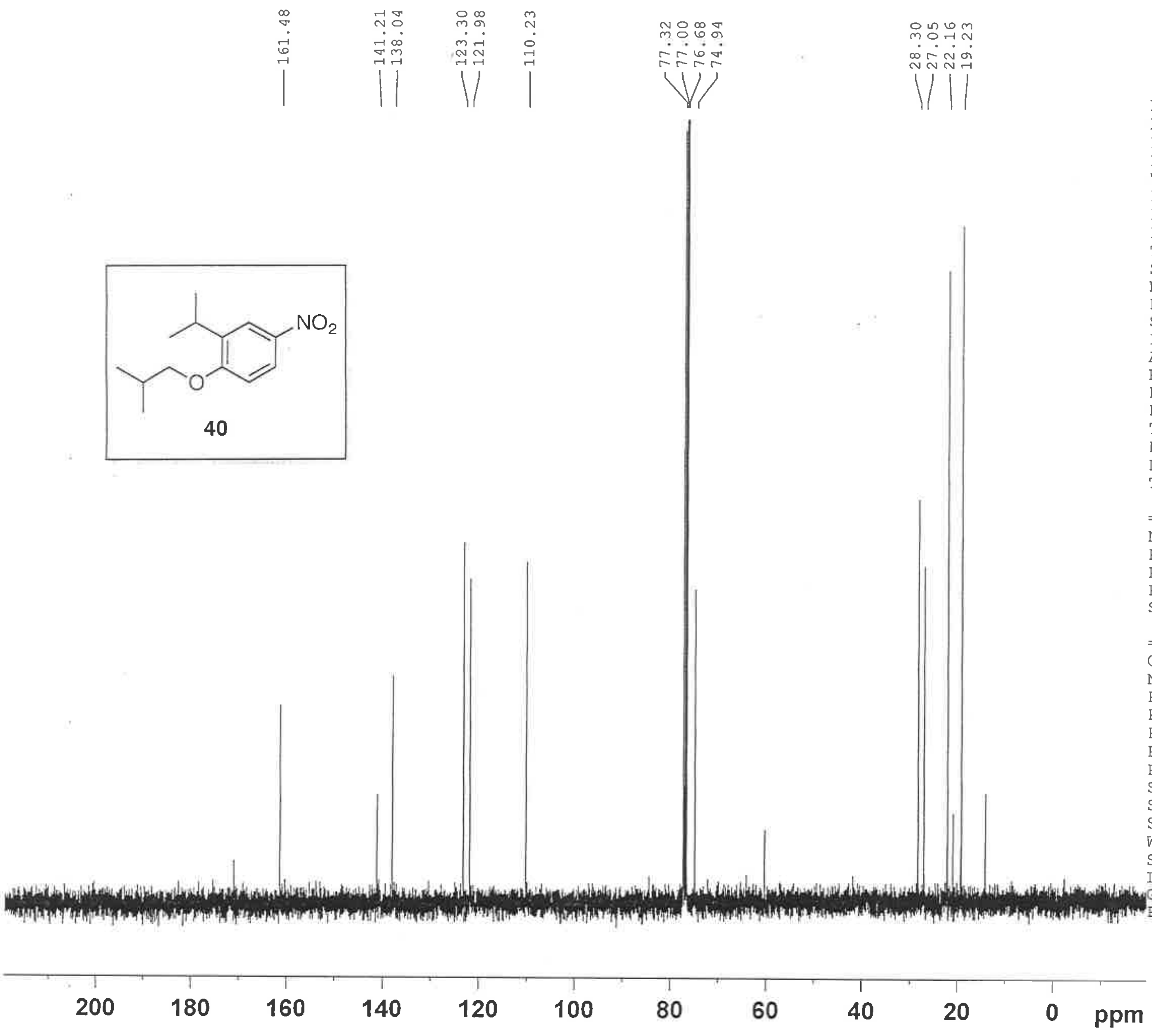

BRUKER

NAME	CWV-197
EXPNO	8
PROCNO	1
Date_	20150108
Time	9.54
INSTRUM	spect
PROBHD	5 mm PABBO BB-
PULPROG	zgig30
TD	65536
SOLVENT	CDCl3
NS	63
DS	4
SWH	24038.461 Hz
FIDRES	0.366798 Hz
AQ	1.3631988 sec
RG	2050
DW	20.800 usec
DE	6.50 usec
TE	298.2 K
D1	2.00000000 sec
D11	0.03000000 sec
TD0	1
======== CHANNEL f1 ========	
NUC1	13C
P1	8.50 usec
PL1	-2.10 dB
PL1W	60.29227829 W
SFO1	100.6228298 MHz
======== CHANNEL f2 ========	
CPDPRG2	waltz16
NUC2	1H
PCPD2	90.00 usec
PL2	-1.80 dB
PL12	17.28 dB
PL2W	21.67079544 W
PL12W	0.26783961 W
SFO2	400.1316005 MHz
SI	32768
SF	100.6127722 MHz
WDW	EM
SSB	0
LB	1.00 Hz
GB	0
PC	1.40

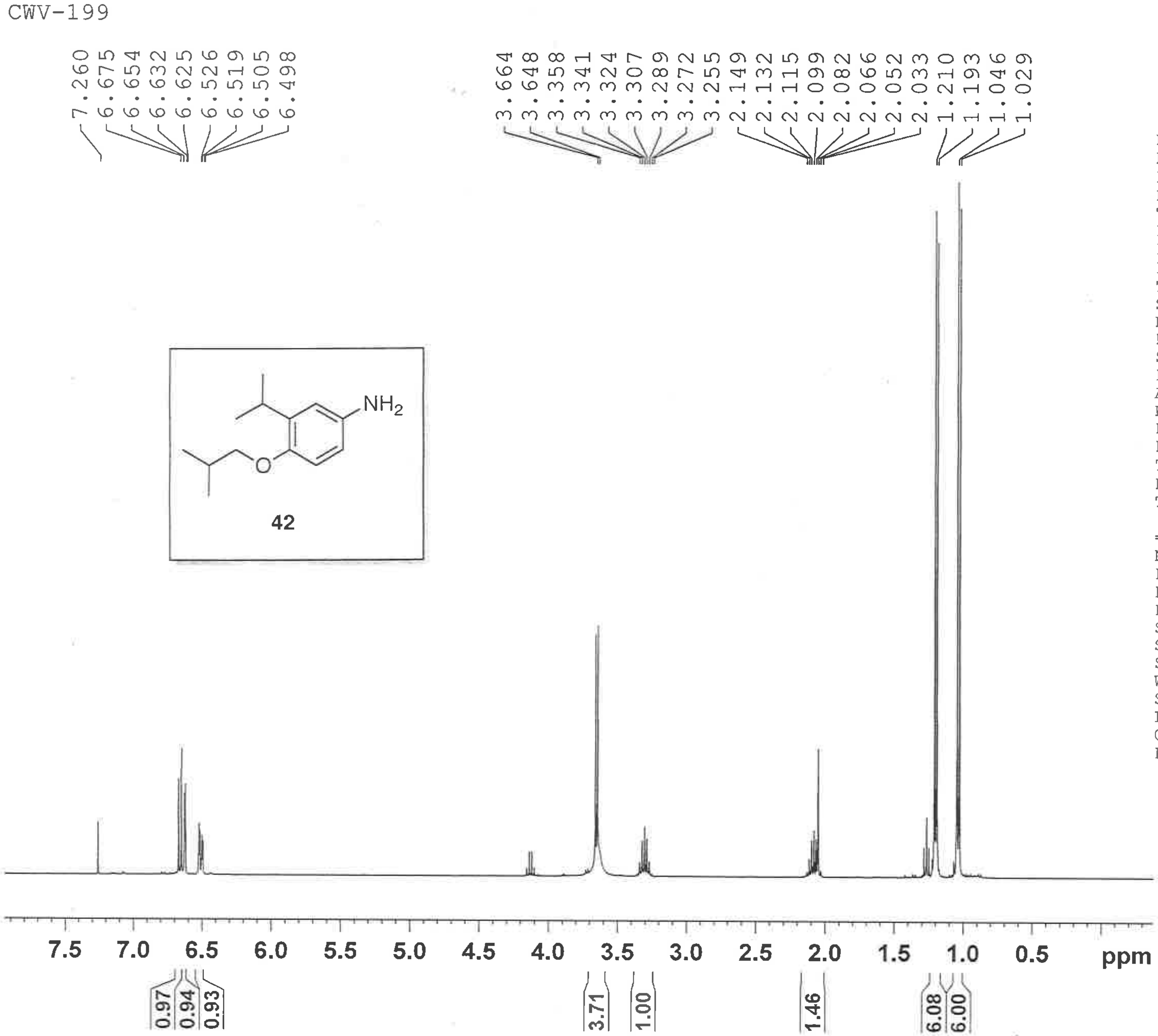

NAME	CWV-199
EXPNO	1
PROCNO	1
Date_	20150112
Time	15.06
INSTRUM	spect
PROBHD	5 mm PABBO BB-
PULPROG	zg30
TD	65536
SOLVENT	CDCl3
NS	16
DS	2
SWH	8223.685 Hz
FIDRES	0.125483 Hz
AQ	3.9846387 sec
RG	4
DW	60.800 usec
DE	6.50 usec
TE	298.2 K
D1	1.00000000 sec
TD0	1

======== CHANNEL f1 ========

NUC1	1H
P1	14.00 usec
PL1	0.50 dB
PL1W	12.76071072 W
SFO1	400.1324710 MHz
SI	32768
SF	400.1300099 MHz
WDW	EM
SSB	0
LB	0.30 Hz
GB	0
PC	1.00

CWV-199

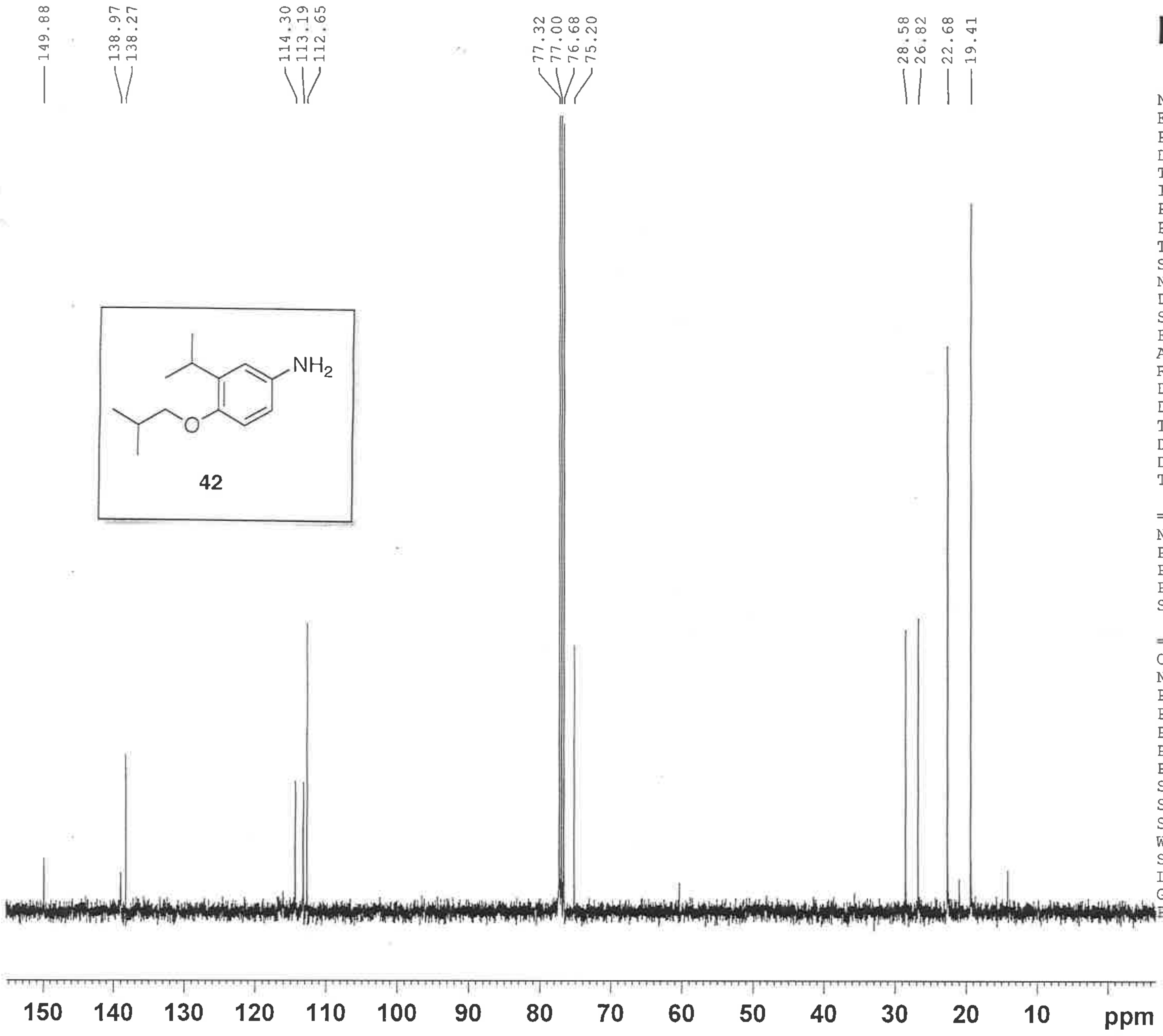

BRUKER

NAME	CWV-199	
EXPNO	2	
PROCNO	1	
Date_	20150112	
Time	15.22	
INSTRUM	spect	
PROBHD	5 mm PABBO BB-	
PULPROG	zgig30	
TD	65536	
SOLVENT	CDCl3	
NS	160	
DS	4	
SWH	24038.461	Hz
FIDRES	0.366798	Hz
AQ	1.3631988	sec
RG	2050	
DW	20.800	usec
DE	6.50	usec
TE	298.2	K
D1	2.00000000	sec
D11	0.03000000	sec
TD0	1	
======== CHANNEL f1 ========		
NUC1	13C	
P1	8.50	usec
PL1	-2.10	dB
PL1W	60.29227829	W
SFO1	100.6228298	MHz
======== CHANNEL f2 ========		
CPDPRG2	waltz16	
NUC2	1H	
PCPD2	90.00	usec
PL2	-1.80	dB
PL12	17.28	dB
PL2W	21.67079544	W
PL12W	0.26783961	W
SFO2	400.1316005	MHz
SI	32768	
SF	100.6127729	MHz
WDW	EM	
SSB	0	
LB	1.00	Hz
GB	0	
PC	1.40	

CWV-219

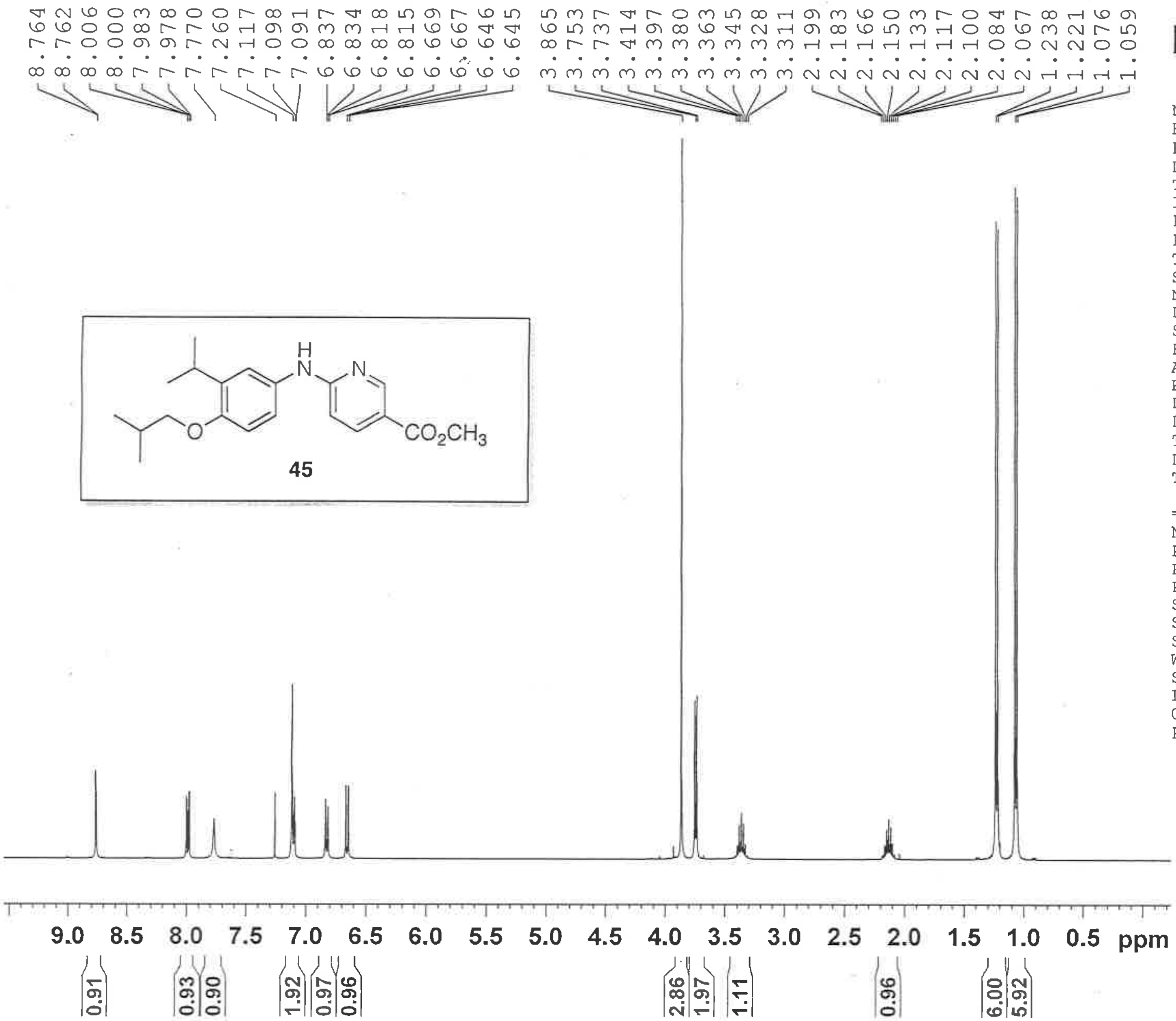

BRUKER

NAME	CWV-219	
EXPNO	1	
PROCNO	1	
Date_	20150331	
Time	12.26	
INSTRUM	spect	
PROBHD	5 mm PABBO BB-	
PULPROG	zg30	
TD	65536	
SOLVENT	CDCl3	
NS	16	
DS	2	
SWH	8223.685	Hz
FIDRES	0.125483	Hz
AQ	3.9846387	sec
RG	4	
DW	60.800	usec
DE	6.50	usec
TE	298.2	K
D1	1.00000000	sec
TD0	1	

======== CHANNEL f1 ========

NUC1	1H	
P1	14.50	usec
PL1	0.50	dB
PL1W	12.76071072	W
SFO1	400.1324710	MHz
SI	32768	
SF	400.1300096	MHz
WDW	EM	
SSB	0	
LB	0.30	Hz
GB	0	
PC	1.00	

CWV-219

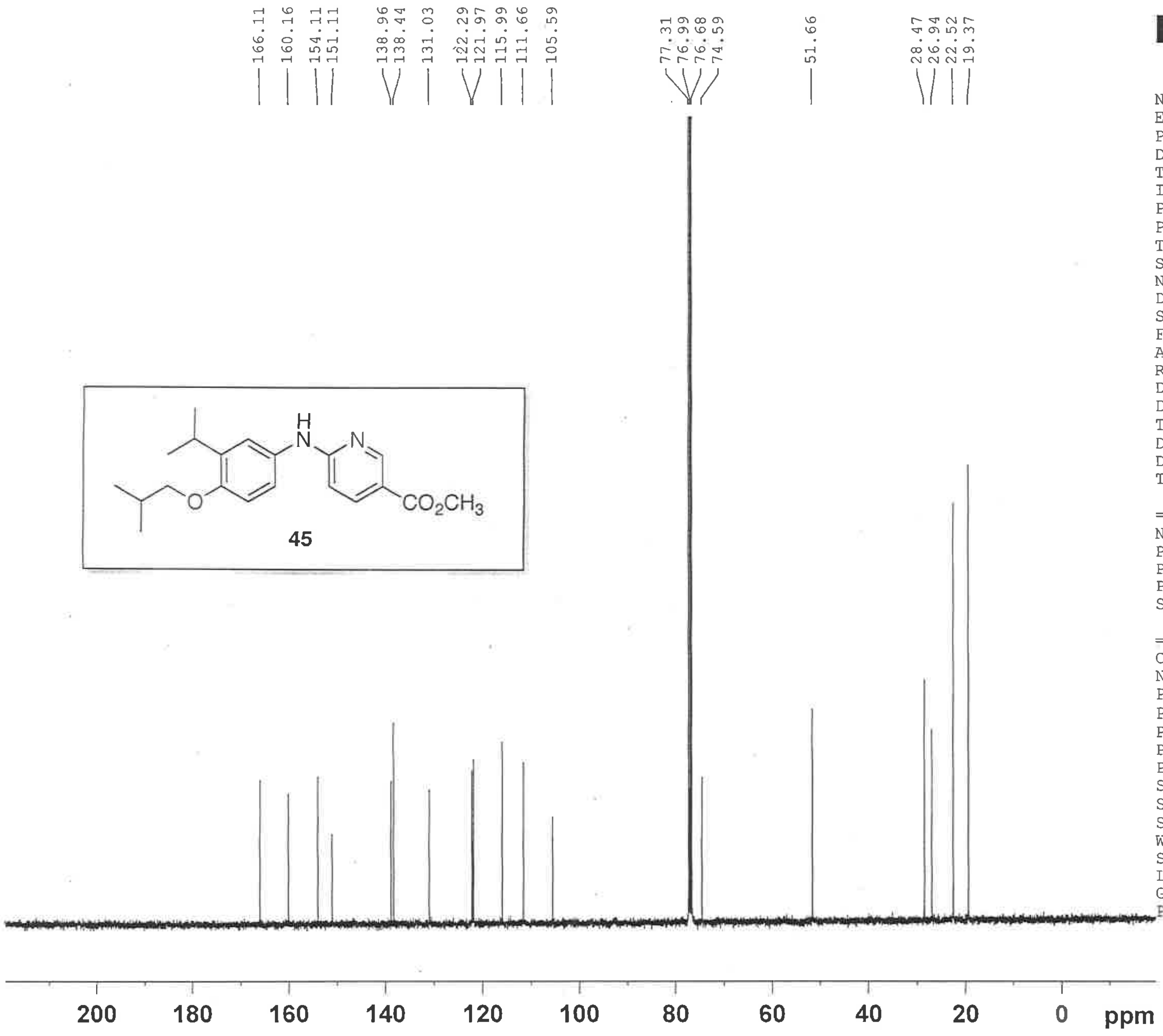

45

BRUKER

NAME	CWV-219
EXPNO	2
PROCNO	1
Date_	20150331
Time	12.32
INSTRUM	spect
PROBHD	5 mm PABBO BB-
PULPROG	zgig30
TD	65536
SOLVENT	CDCl3
NS	1024
DS	4
SWH	24038.461 Hz
FIDRES	0.366798 Hz
AQ	1.3631988 sec
RG	2050
DW	20.800 usec
DE	6.50 usec
TE	298.5 K
D1	2.00000000 sec
D11	0.03000000 sec
TD0	1

======== CHANNEL f1 ========

NUC1	13C
P1	8.50 usec
PL1	-2.10 dB
PL1W	60.29227829 W
SFO1	100.6228298 MHz

======== CHANNEL f2 ========

CPDPRG2	waltz16
NUC2	1H
PCPD2	90.00 usec
PL2	-1.80 dB
PL12	17.28 dB
PL2W	21.67079544 W
PL12W	0.26783961 W
SFO2	400.1316005 MHz
SI	32768
SF	100.6127729 MHz
WDW	EM
SSB	0
LB	1.00 Hz
GB	0
PC	1.40

CWV-201

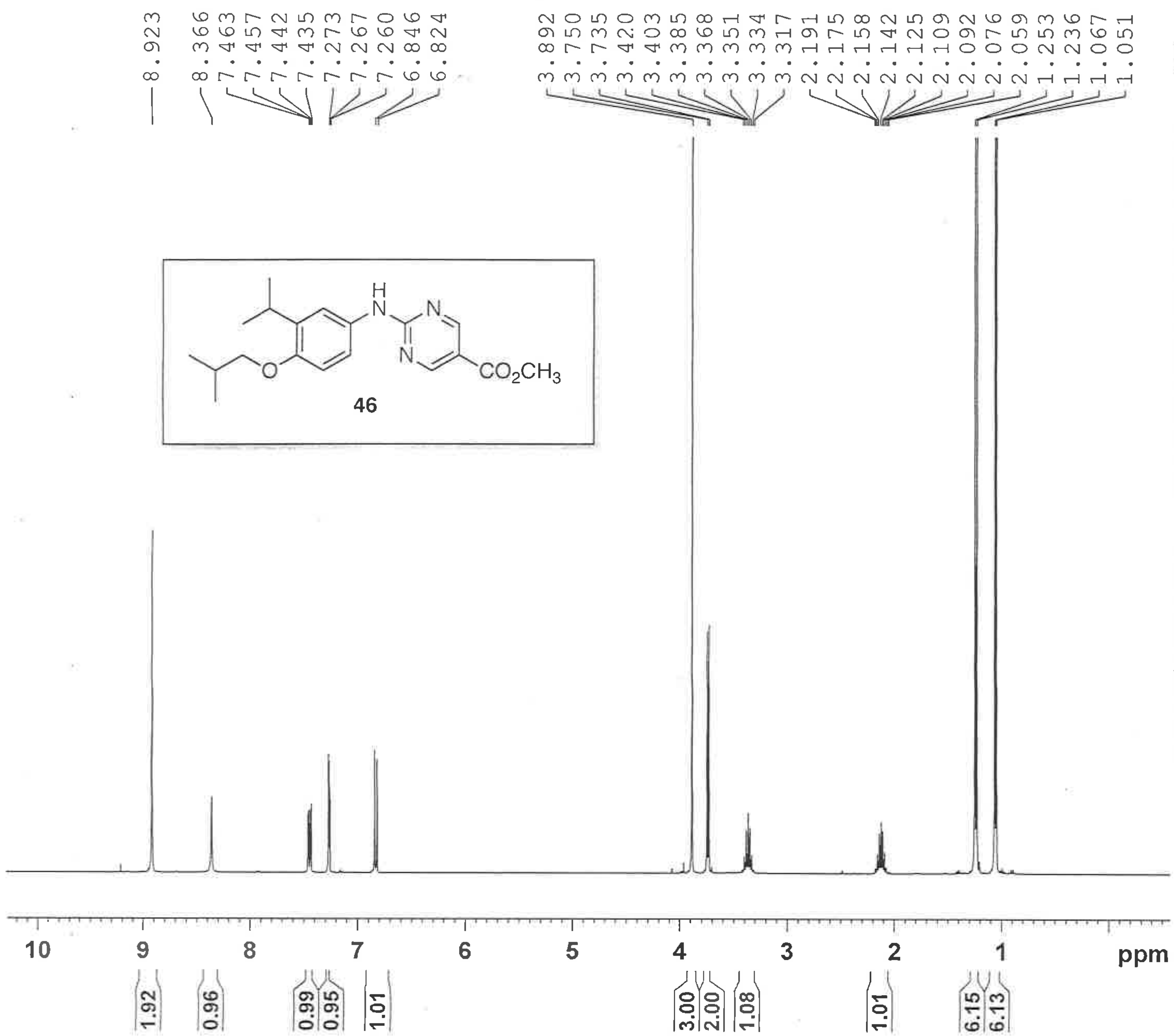

BRUKER

NAME	CWV-201
EXPNO	1
PROCNO	1
Date_	20150116
Time	16.58
INSTRUM	spect
PROBHD	5 mm PABBO BB-
PULPROG	zg30
TD	65536
SOLVENT	CDCl3
NS	16
DS	2
SWH	8223.685 Hz
FIDRES	0.125483 Hz
AQ	3.9846387 sec
RG	4
DW	60.800 usec
DE	6.50 usec
TE	298.2 K
D1	1.00000000 sec
TD0	1

======== CHANNEL f1 ========

NUC1	1H
P1	14.00 usec
PL1	0.50 dB
PL1W	12.76071072 W
SFO1	400.1324710 MHz
SI	32768
SF	400.1300099 MHz
WDW	EM
SSB	0
LB	0.30 Hz
GB	0
PC	1.00

CWV-201

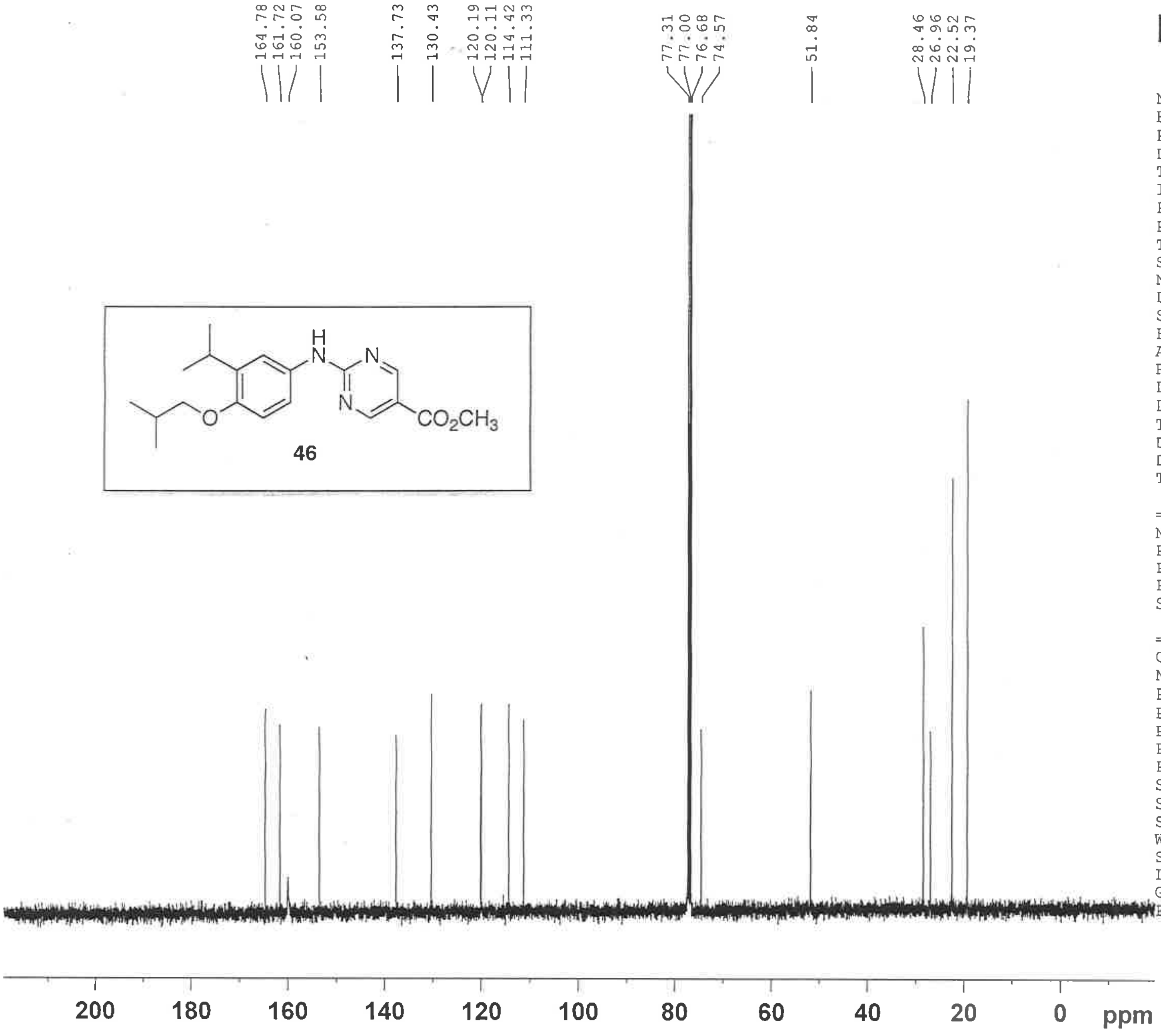

46

NAME	CWV-201
EXPNO	2
PROCNO	1
Date_	20150116
Time	17.07
INSTRUM	spect
PROBHD	5 mm PABBO BB-
PULPROG	zgig30
TD	65536
SOLVENT	CDCl3
NS	208
DS	4
SWH	24038.461 Hz
FIDRES	0.366798 Hz
AQ	1.3631988 sec
RG	2050
DW	20.800 usec
DE	6.50 usec
TE	298.2 K
D1	2.00000000 sec
D11	0.03000000 sec
TD0	1
======== CHANNEL f1 ========	
NUC1	13C
P1	8.50 usec
PL1	-2.10 dB
PL1W	60.29227829 W
SFO1	100.6228298 MHz
======== CHANNEL f2 ========	
CPDPRG2	waltz16
NUC2	1H
PCPD2	90.00 usec
PL2	-1.80 dB
PL12	17.28 dB
PL2W	21.67079544 W
PL12W	0.26783961 W
SFO2	400.1316005 MHz
SI	32768
SF	100.6127736 MHz
WDW	EM
SSB	0
LB	1.00 Hz
GB	0
PC	1.40

CWV-215

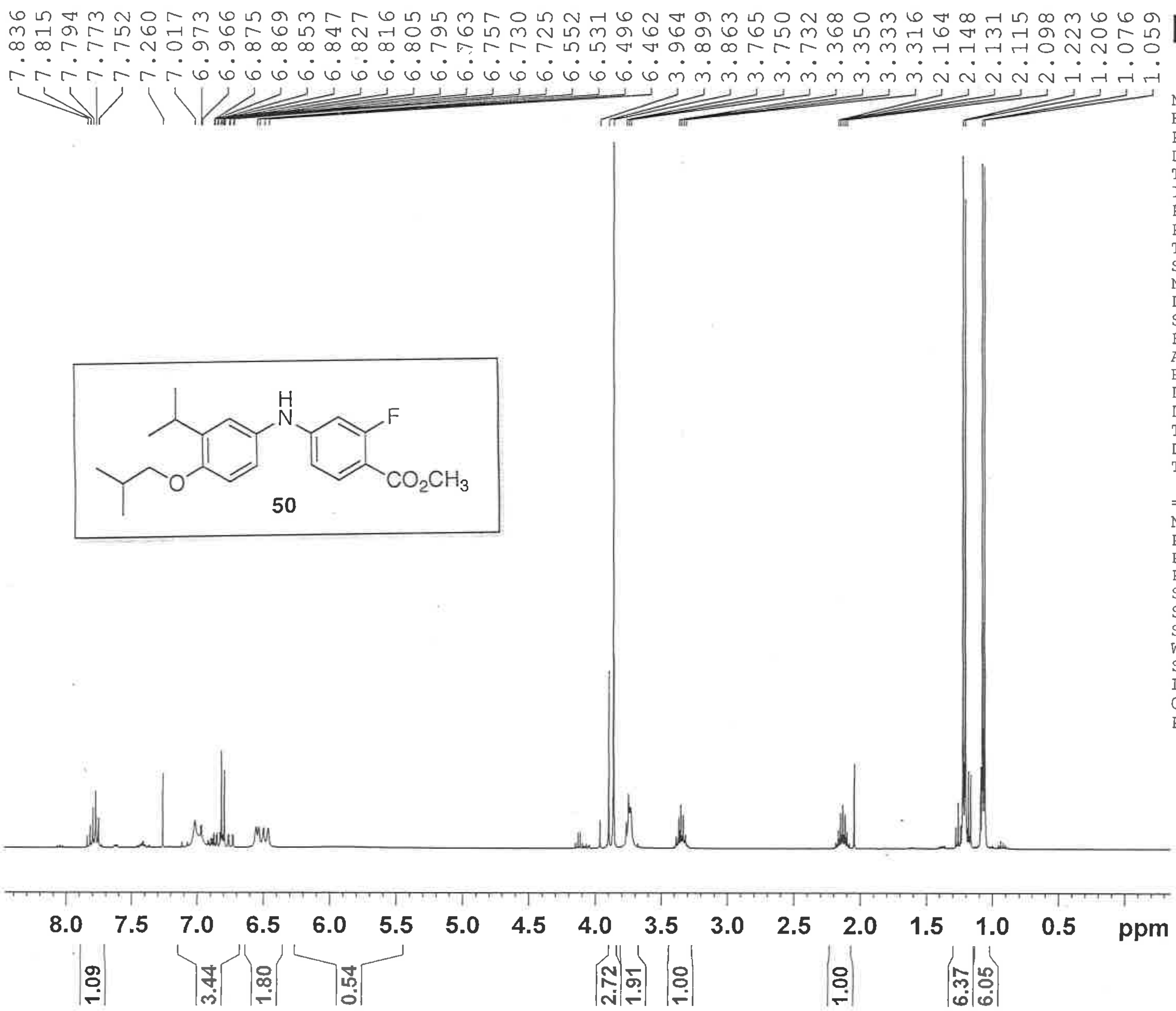

NAME	CWV-215	
EXPNO	1	
PROCNO	1	
Date_	20150331	
Time	13.42	
INSTRUM	spect	
PROBHD	5 mm PABBO BB-	
PULPROG	zg30	
TD	65536	
SOLVENT	CDCl3	
NS	16	
DS	2	
SWH	8223.685	Hz
FIDRES	0.125483	Hz
AQ	3.9846387	sec
RG	4	
DW	60.800	usec
DE	6.50	usec
TE	298.0	K
D1	1.00000000	sec
TD0	1	

======== CHANNEL f1 ========

NUC1	1H	
P1	14.50	usec
PL1	0.50	dB
PL1W	12.76071072	W
SFO1	400.1324710	MHz
SI	32768	
SF	400.1300096	MHz
WDW	EM	
SSB	0	
LB	0.30	Hz
GB	0	
PC	1.00	

CWV-215

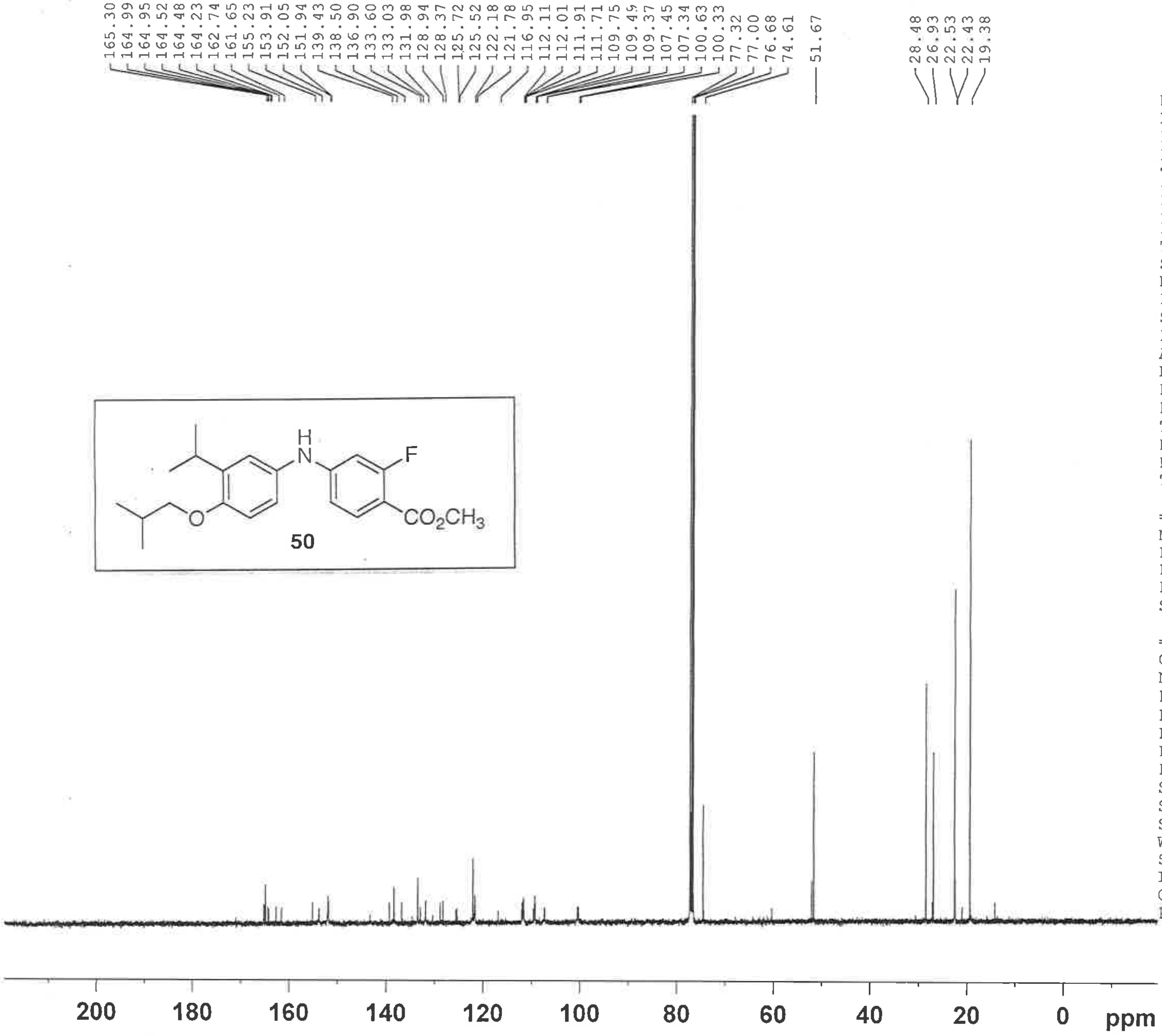

50

BRUKER

NAME	CWV-215
EXPNO	4
PROCNO	1
Date_	20150401
Time	8.55
INSTRUM	spect
PROBHD	5 mm PABBO BB-
PULPROG	zgig30
TD	65536
SOLVENT	CDCl3
NS	2048
DS	4
SWH	24038.461 Hz
FIDRES	0.366798 Hz
AQ	1.3631988 sec
RG	2050
DW	20.800 usec
DE	6.50 usec
TE	298.2 K
D1	2.00000000 sec
D11	0.03000000 sec
TD0	1

======== CHANNEL f1 ========

NUC1	13C
P1	8.50 usec
PL1	-2.10 dB
PL1W	60.29227829 W
SFO1	100.6228298 MHz

======== CHANNEL f2 ========

CPDPRG2	waltz16
NUC2	1H
PCPD2	90.00 usec
PL2	-1.80 dB
PL12	17.28 dB
PL2W	21.67079544 W
PL12W	0.26783961 W
SFO2	400.1316005 MHz
SI	32768
SF	100.6127722 MHz
WDW	EM
SSB	0
LB	1.00 Hz
GB	0
PC	1.40

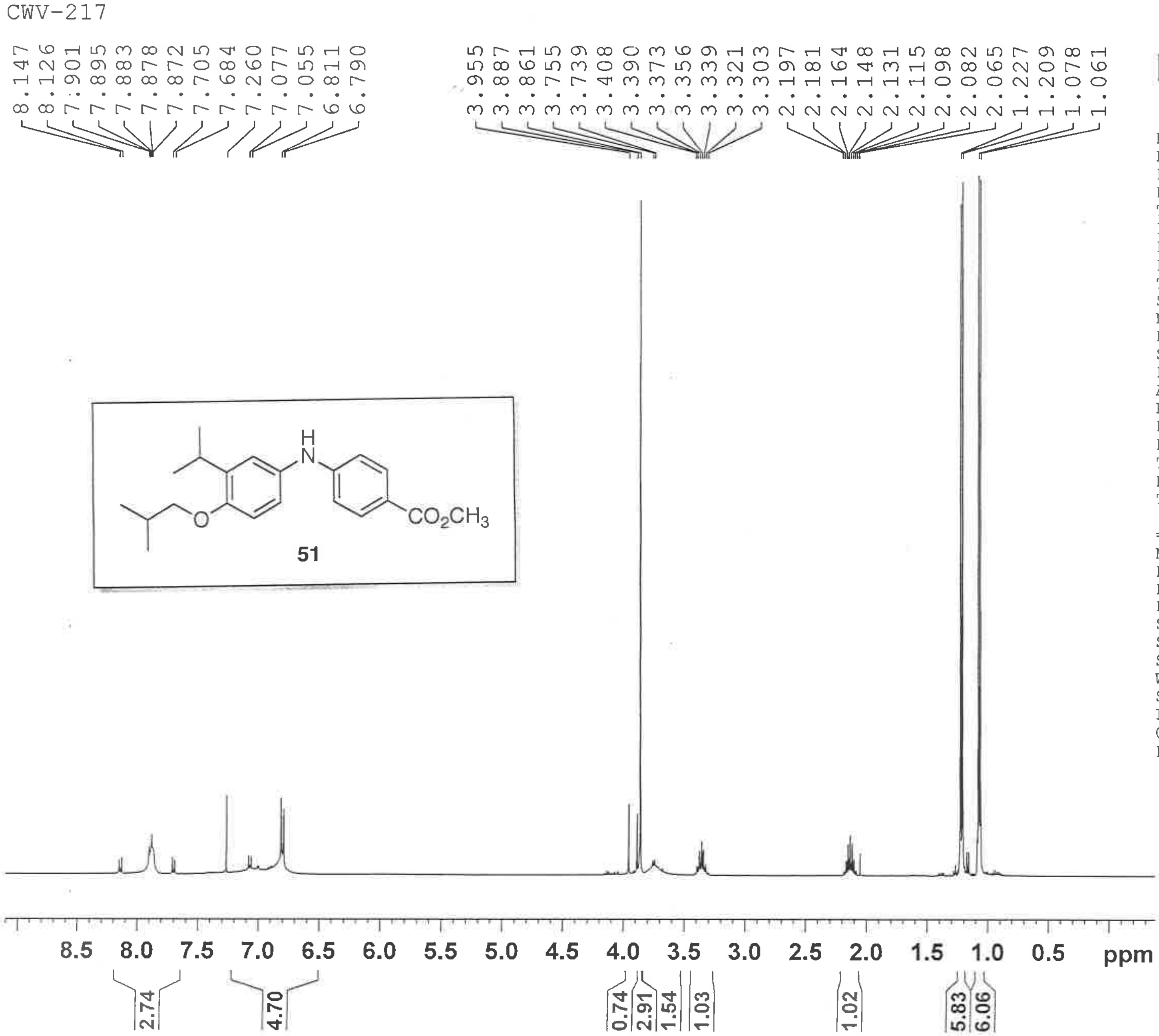

BRUKER

NAME	CWV-217
EXPNO	1
PROCNO	1
Date_	20150325
Time	17.50
INSTRUM	spect
PROBHD	5 mm PABBO BB-
PULPROG	zg30
TD	65536
SOLVENT	CDCl3
NS	16
DS	2
SWH	8223.685 Hz
FIDRES	0.125483 Hz
AQ	3.9846387 sec
RG	4
DW	60.800 usec
DE	6.50 usec
TE	298.1 K
D1	1.00000000 sec
TD0	1

======== CHANNEL f1 ========

NUC1	1H
P1	14.50 usec
PL1	0.50 dB
PL1W	12.76071072 W
SFO1	400.1324710 MHz
SI	32768
SF	400.1300096 MHz
WDW	EM
SSB	0
LB	0.30 Hz
GB	0
PC	1.00

CWV-217

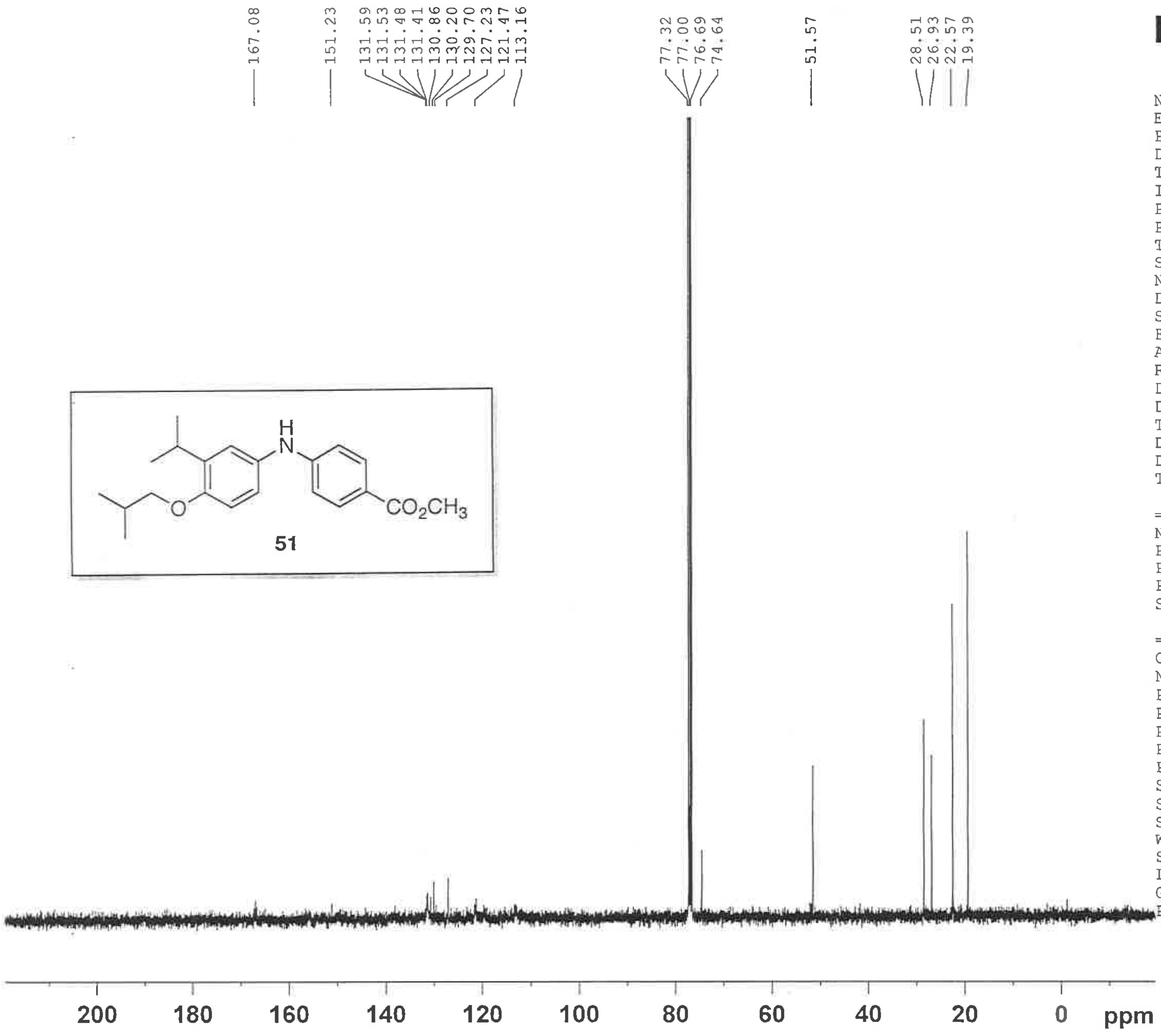

BRUKER

NAME	CWV-217
EXPNO	2
PROCNO	1
Date_	20150325
Time	18.20
INSTRUM	spect
PROBHD	5 mm PABBO BB-
PULPROG	zgig30
TD	65536
SOLVENT	CDCl3
NS	365
DS	4
SWH	24038.461 Hz
FIDRES	0.366798 Hz
AQ	1.3631988 sec
RG	2050
DW	20.800 usec
DE	6.50 usec
TE	298.5 K
D1	2.00000000 sec
D11	0.03000000 sec
TD0	1
======== CHANNEL f1 ========	
NUC1	13C
P1	8.50 usec
PL1	-2.10 dB
PL1W	60.29227829 W
SFO1	100.6228298 MHz
======== CHANNEL f2 ========	
CPDPRG2	waltz16
NUC2	1H
PCPD2	90.00 usec
PL2	-1.80 dB
PL12	17.28 dB
PL2W	21.67079544 W
PL12W	0.26783961 W
SFO2	400.1316005 MHz
SI	32768
SF	100.6127722 MHz
WDW	EM
SSB	0
LB	1.00 Hz
GB	0
PC	1.40

CWV-231

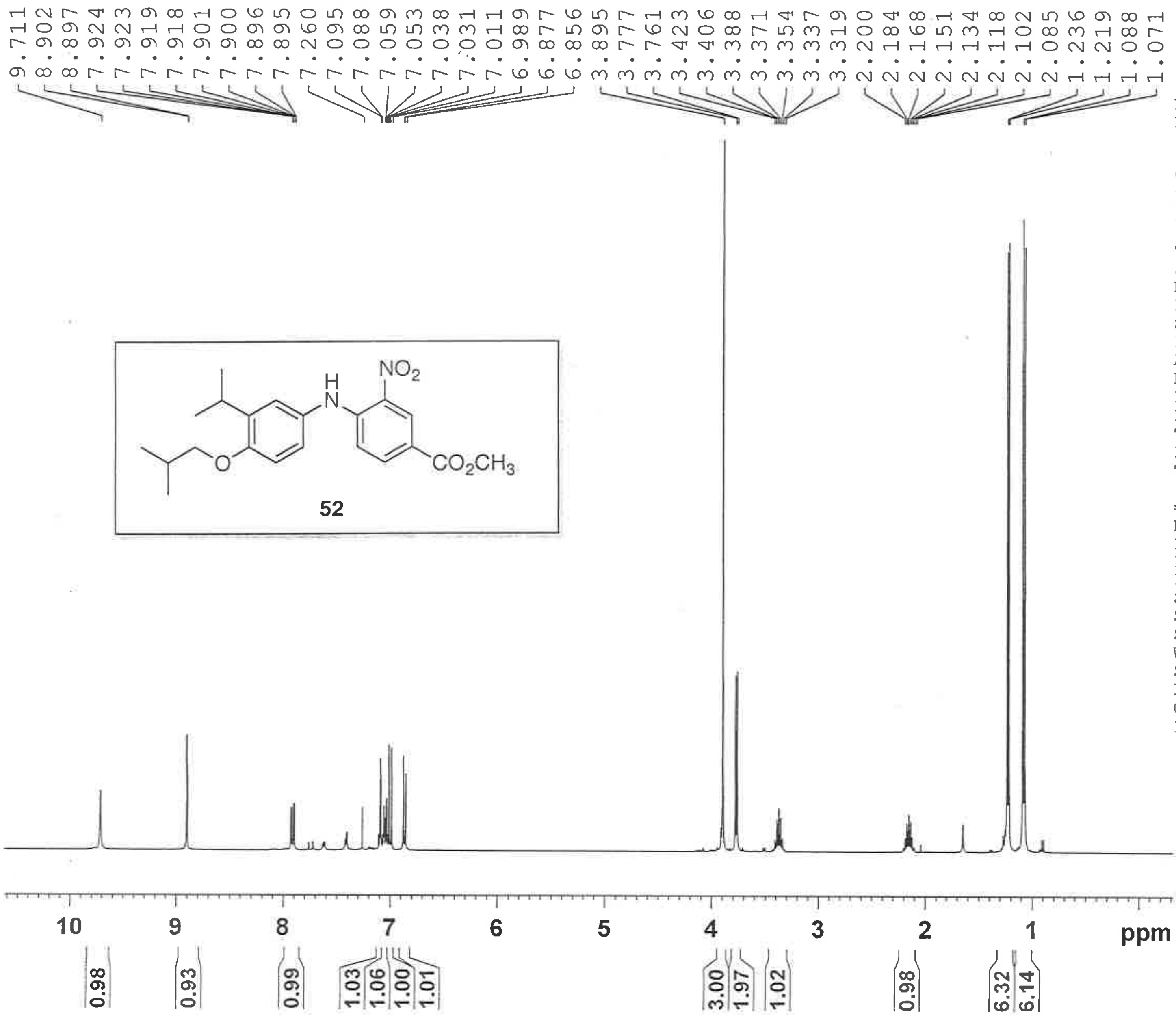

NAME	CWV-231
EXPNO	1
PROCNO	1
Date_	20150706
Time	9.23
INSTRUM	spect
PROBHD	5 mm PABBO BB-
PULPROG	zg30
TD	65536
SOLVENT	CDCl3
NS	16
DS	2
SWH	8223.685 Hz
FIDRES	0.125483 Hz
AQ	3.9846387 sec
RG	4
DW	60.800 usec
DE	6.50 usec
TE	298.2 K
D1	1.00000000 sec
TD0	1

======== CHANNEL f1 ========

NUC1	1H
P1	14.50 usec
PL1	0.50 dB
PL1W	12.76071072 W
SFO1	400.1324710 MHz
SI	32768
SF	400.1300099 MHz
WDW	EM
SSB	0
LB	0.30 Hz
GB	0
PC	1.00

CWV-231

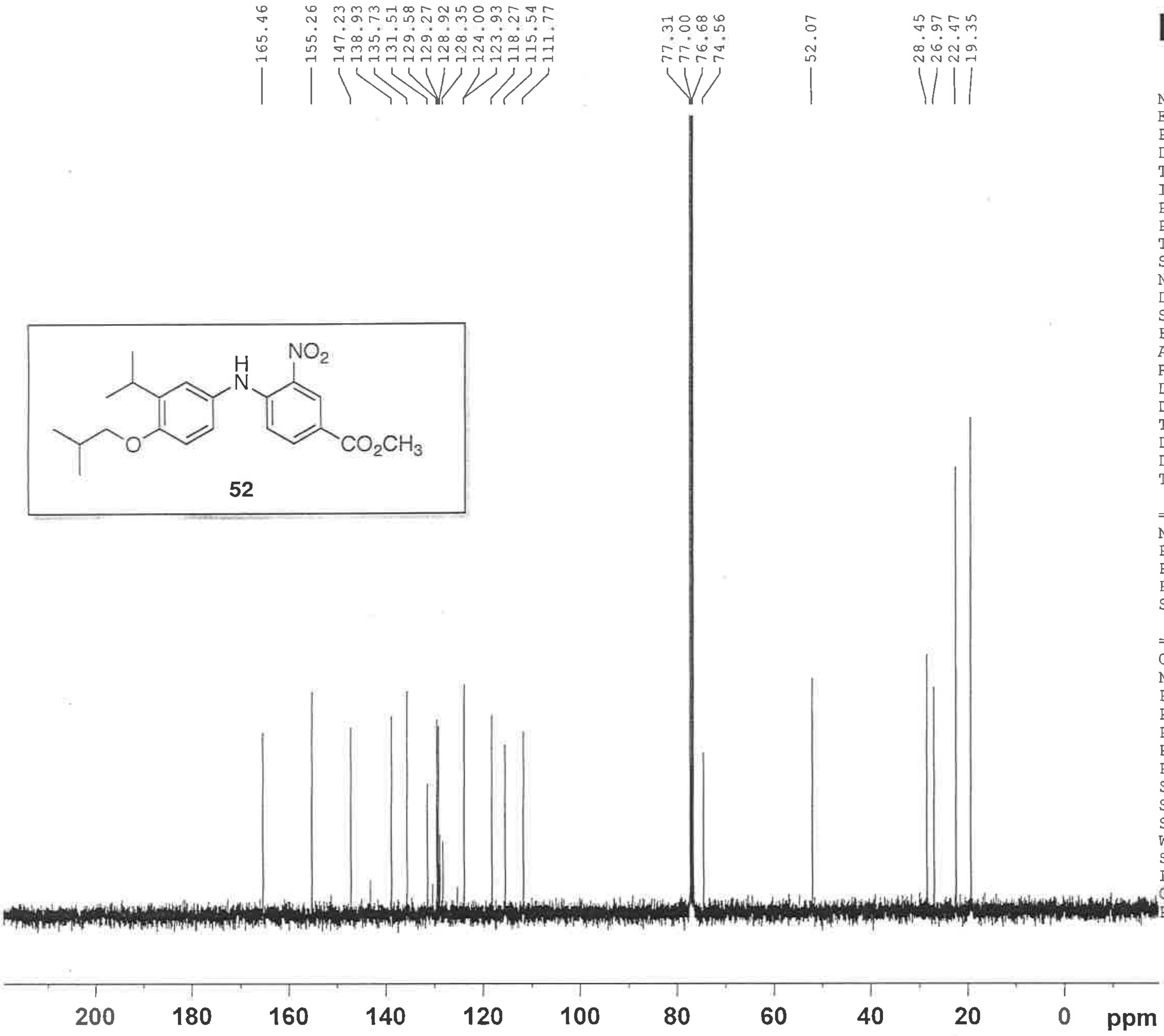

BRUKER

NAME	CWV-231
EXPNO	2
PROCNO	1
Date_	20150706
Time	9.32
INSTRUM	spect
PROBHD	5 mm PABBO BB-
PULPROG	zgig30
TD	65536
SOLVENT	CDC13
NS	158
DS	4
SWH	24038.461 Hz
FIDRES	0.366798 Hz
AQ	1.3631988 sec
RG	2050
DW	20.800 usec
DE	6.50 usec
TE	298.5 K
D1	2.00000000 sec
D11	0.03000000 sec
TD0	1

======== CHANNEL f1 ========

NUC1	13C
P1	8.50 usec
PL1	-2.10 dB
PL1W	60.29227829 W
SFO1	100.6228298 MHz

======== CHANNEL f2 ========

CPDPRG2	waltz16
NUC2	1H
PCPD2	90.00 usec
PL2	-1.80 dB
PL12	17.28 dB
PL2W	21.67079544 W
PL12W	0.26783961 W
SFO2	400.1316005 MHz
SI	32768
SF	100.6127736 MHz
WDW	EM
SSB	0
LB	1.00 Hz
GB	0
PC	1.40

CWV-221

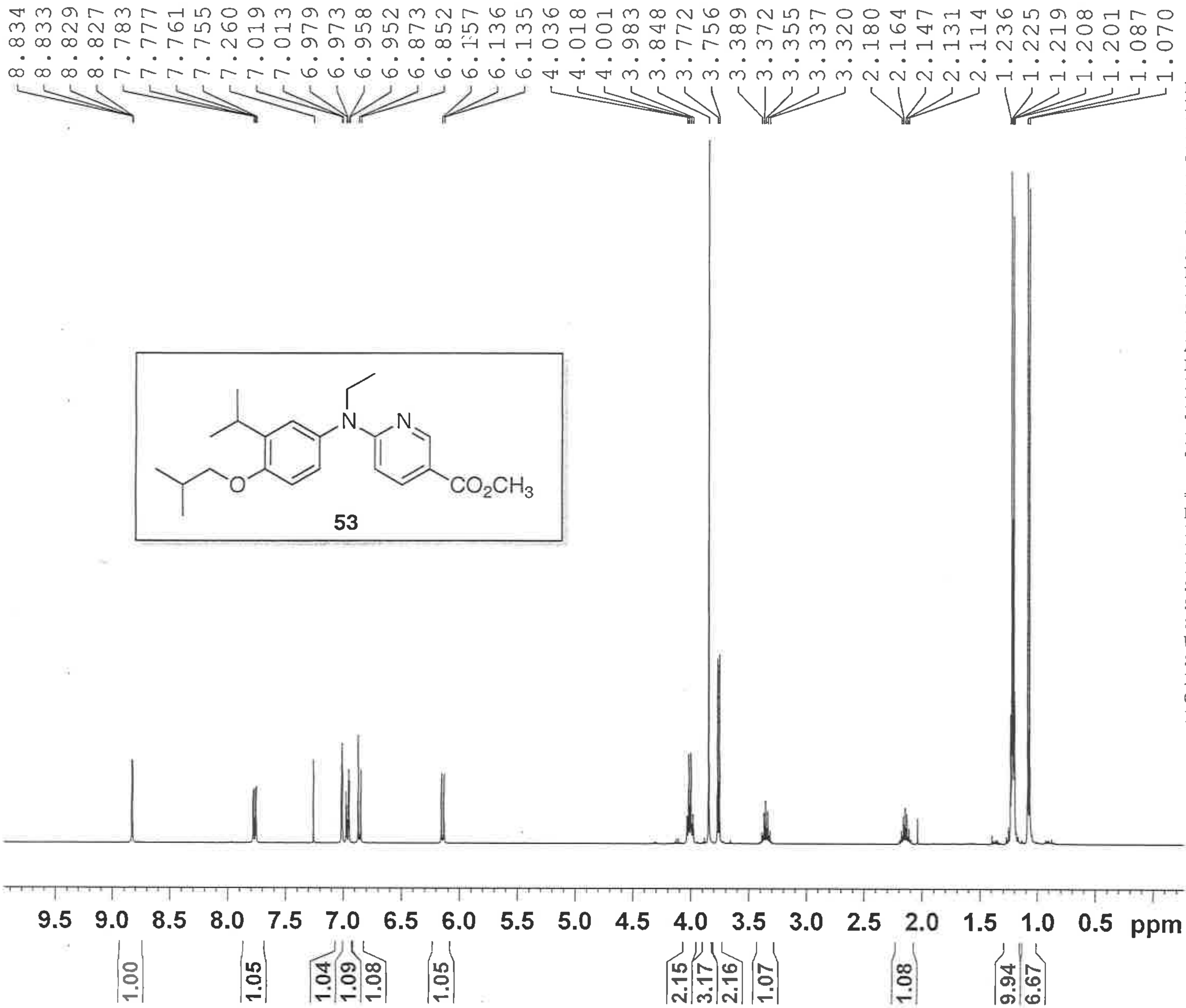

53

NAME	CWV-221
EXPNO	1
PROCNO	1
Date_	20150420
Time	13.00
INSTRUM	spect
PROBHD	5 mm PABBO BB-
PULPROG	zg30
TD	65536
SOLVENT	CDCl3
NS	16
DS	2
SWH	8223.685 Hz
FIDRES	0.125483 Hz
AQ	3.9846387 sec
RG	4
DW	60.800 usec
DE	6.50 usec
TE	298.0 K
D1	1.00000000 sec
TD0	1

======== CHANNEL f1 ========

NUC1	1H
P1	14.50 usec
PL1	0.50 dB
PL1W	12.76071072 W
SFO1	400.1324710 MHz
SI	32768
SF	400.1300096 MHz
WDW	EM
SSB	0
LB	0.30 Hz
GB	0
PC	1.00

CWV-221

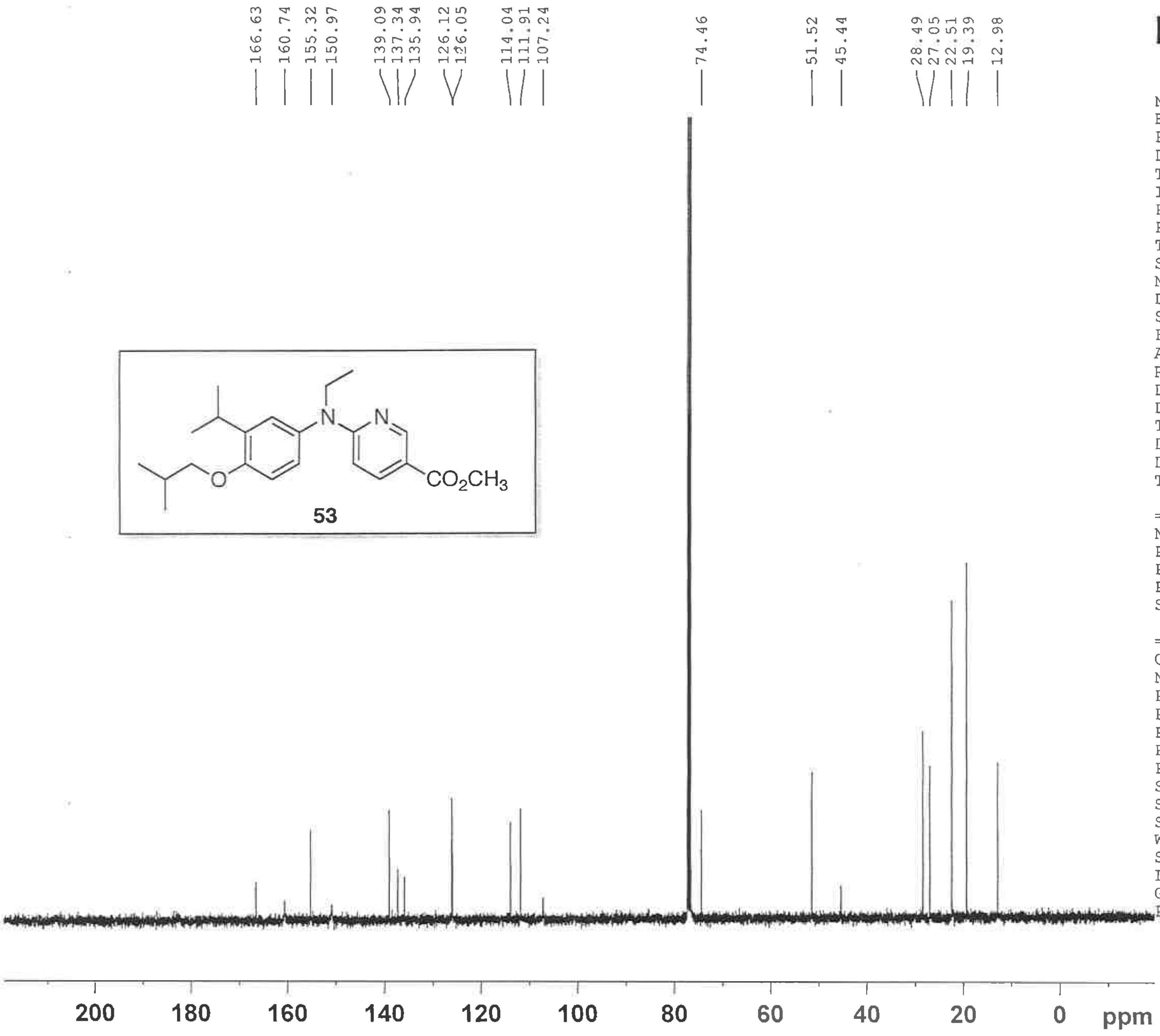

53

```
NAME            CWV-221
EXPNO                 2
PROCNO                1
Date_          20150420
Time              13.16
INSTRUM           spect
PROBHD   5 mm PABBO BB-
PULPROG         zgig30
TD                65536
SOLVENT           CDCl3
NS                  464
DS                    4
SWH           24038.461 Hz
FIDRES         0.366798 Hz
AQ            1.3631988 sec
RG                 2050
DW               20.800 usec
DE                 6.50 usec
TE                298.5 K
D1           2.00000000 sec
D11          0.03000000 sec
TD0                   1

======== CHANNEL f1 ========
NUC1                13C
P1                 8.50 usec
PL1               -2.10 dB
PL1W        60.29227829 W
SFO1        100.6228298 MHz

======== CHANNEL f2 ========
CPDPRG2         waltz16
NUC2                 1H
PCPD2             90.00 usec
PL2               -1.80 dB
PL12              17.28 dB
PL2W        21.67079544 W
PL12W        0.26783961 W
SFO2        400.1316005 MHz
SI                32768
SF          100.6127714 MHz
WDW                  EM
SSB                   0
LB                 1.00 Hz
GB                    0
PC                 1.40
```

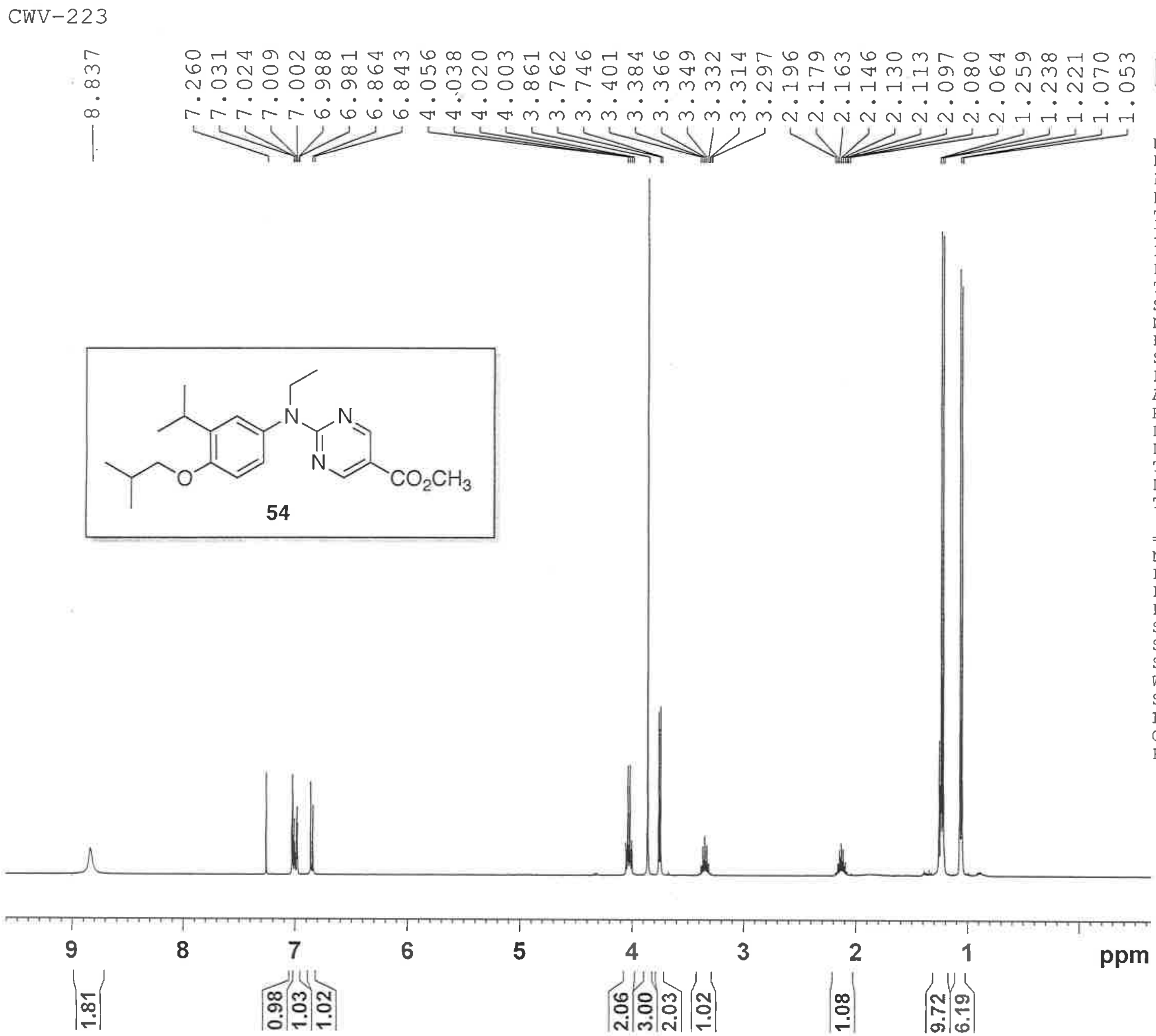

BRUKER

NAME	CWV-223
EXPNO	1
PROCNO	1
Date_	20150413
Time	12.17
INSTRUM	spect
PROBHD	5 mm PABBO BB-
PULPROG	zg30
TD	65536
SOLVENT	CDCl3
NS	16
DS	2
SWH	8223.685 Hz
FIDRES	0.125483 Hz
AQ	3.9846387 sec
RG	4
DW	60.800 usec
DE	6.50 usec
TE	298.2 K
D1	1.00000000 sec
TD0	1

======== CHANNEL f1 ========

NUC1	1H
P1	14.50 usec
PL1	0.50 dB
PL1W	12.76071072 W
SFO1	400.1324710 MHz
SI	32768
SF	400.1300096 MHz
WDW	EM
SSB	0
LB	0.30 Hz
GB	0
PC	1.00

CWV-223

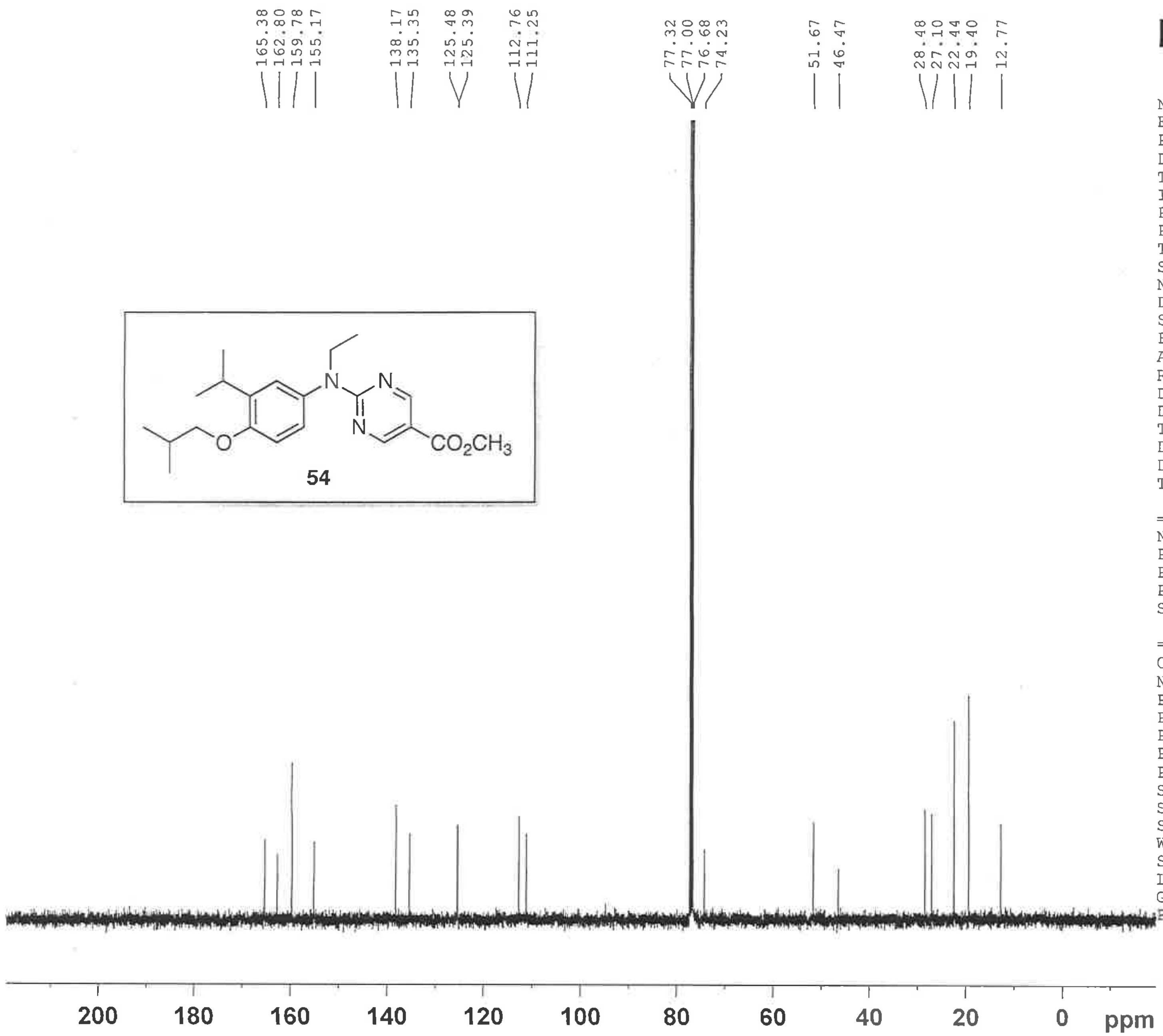

BRUKER

NAME	CWV-223
EXPNO	2
PROCNO	1
Date_	20150413
Time	12.37
INSTRUM	spect
PROBHD	5 mm PABBO BB-
PULPROG	zgig30
TD	65536
SOLVENT	CDCl3
NS	284
DS	4
SWH	24038.461 Hz
FIDRES	0.366798 Hz
AQ	1.3631988 sec
RG	2050
DW	20.800 usec
DE	6.50 usec
TE	298.6 K
D1	2.00000000 sec
D11	0.03000000 sec
TD0	1

======== CHANNEL f1 ========

NUC1	13C
P1	8.50 usec
PL1	-2.10 dB
PL1W	60.29227829 W
SFO1	100.6228298 MHz

======== CHANNEL f2 ========

CPDPRG2	waltz16
NUC2	1H
PCPD2	90.00 usec
PL2	-1.80 dB
PL12	17.28 dB
PL2W	21.67079544 W
PL12W	0.26783961 W
SFO2	400.1316005 MHz
SI	32768
SF	100.6127714 MHz
WDW	EM
SSB	0
LB	1.00 Hz
GB	0
PC	1.40

CWV-229

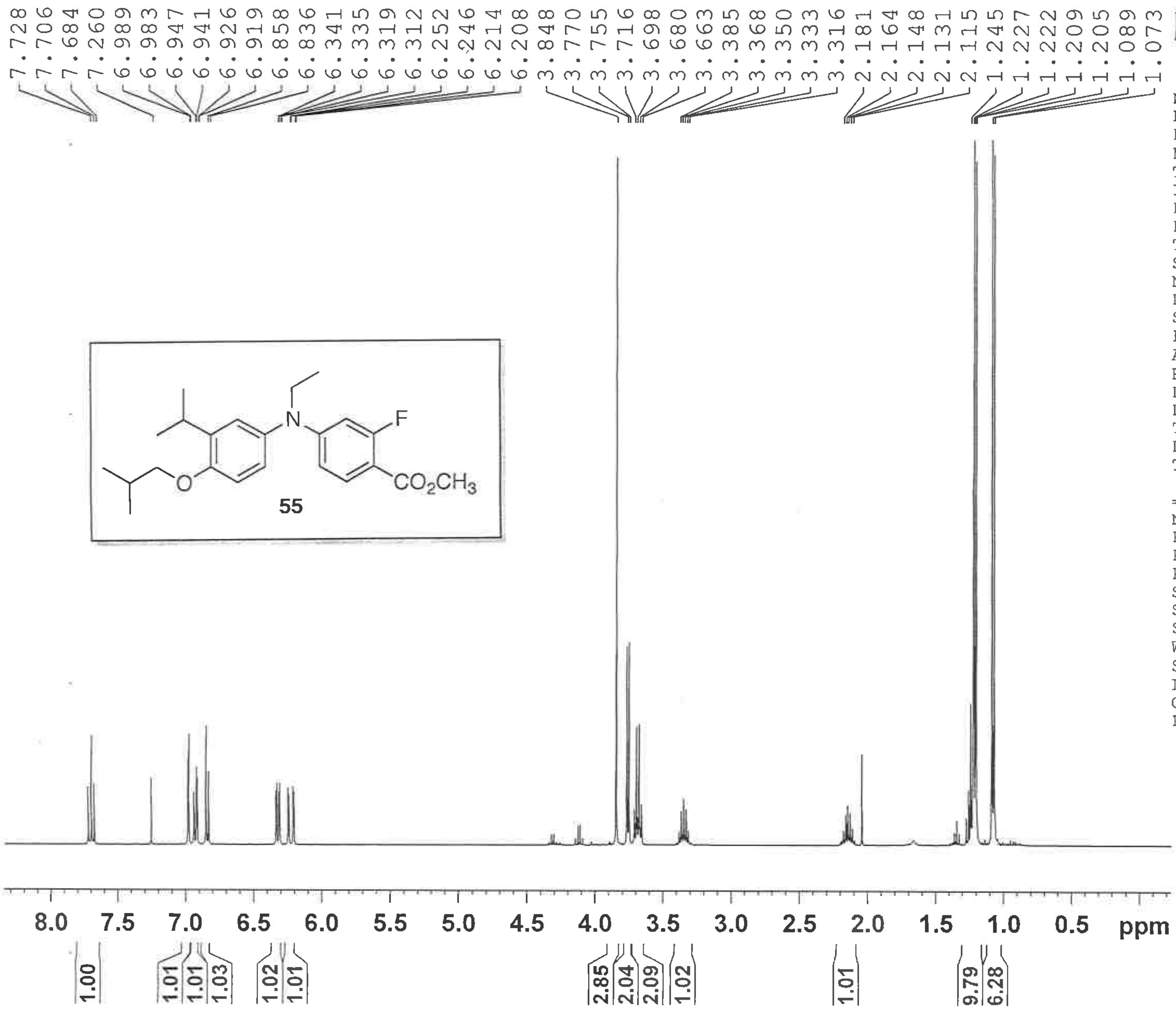

55

BRUKER

NAME	CWV-229
EXPNO	1
PROCNO	1
Date_	20150420
Time	11.56
INSTRUM	spect
PROBHD	5 mm PABBO BB-
PULPROG	zg30
TD	65536
SOLVENT	CDCl3
NS	16
DS	2
SWH	8223.685 Hz
FIDRES	0.125483 Hz
AQ	3.9846387 sec
RG	4
DW	60.800 usec
DE	6.50 usec
TE	298.2 K
D1	1.00000000 sec
TD0	1
======== CHANNEL f1 ========	
NUC1	1H
P1	14.50 usec
PL1	0.50 dB
PL1W	12.76071072 W
SFO1	400.1324710 MHz
SI	32768
SF	400.1300096 MHz
WDW	EM
SSB	0
LB	0.30 Hz
GB	0
PC	1.00

CWV-229

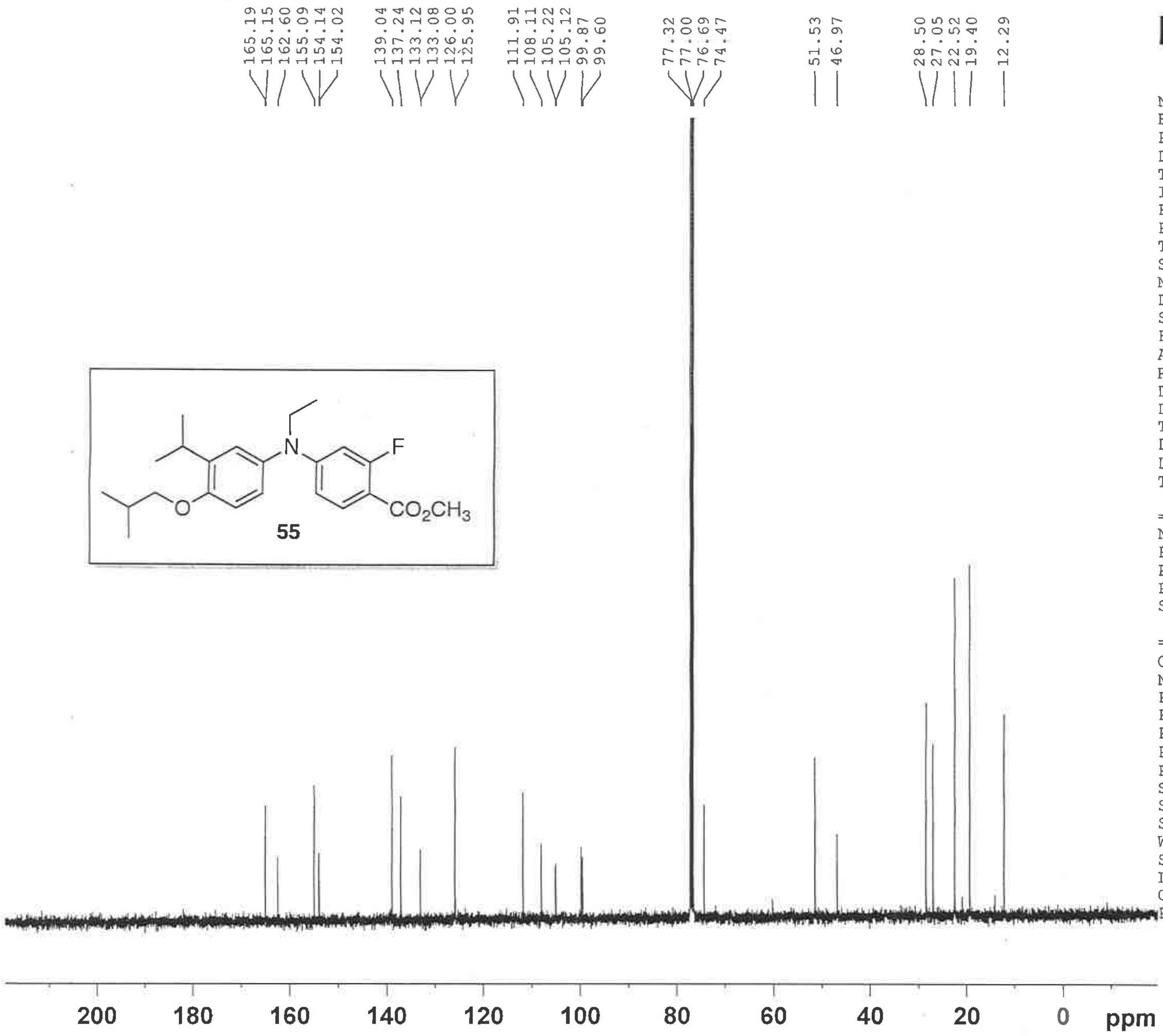

BRUKER

NAME	CWV-229
EXPNO	2
PROCNO	1
Date_	20150420
Time	12.14
INSTRUM	spect
PROBHD	5 mm PABBO BB-
PULPROG	zgig30
TD	65536
SOLVENT	CDCl3
NS	376
DS	4
SWH	24038.461 Hz
FIDRES	0.366798 Hz
AQ	1.3631988 sec
RG	2050
DW	20.800 usec
DE	6.50 usec
TE	298.5 K
D1	2.00000000 sec
D11	0.03000000 sec
TD0	1

======== CHANNEL f1 ========

NUC1	13C
P1	8.50 usec
PL1	-2.10 dB
PL1W	60.29227829 W
SFO1	100.6228298 MHz

======== CHANNEL f2 ========

CPDPRG2	waltz16
NUC2	1H
PCPD2	90.00 usec
PL2	-1.80 dB
PL12	17.28 dB
PL2W	21.67079544 W
PL12W	0.26783961 W
SFO2	400.1316005 MHz
SI	32768
SF	100.6127714 MHz
WDW	EM
SSB	0
LB	1.00 Hz
GB	0
PC	1.40

CWV-227

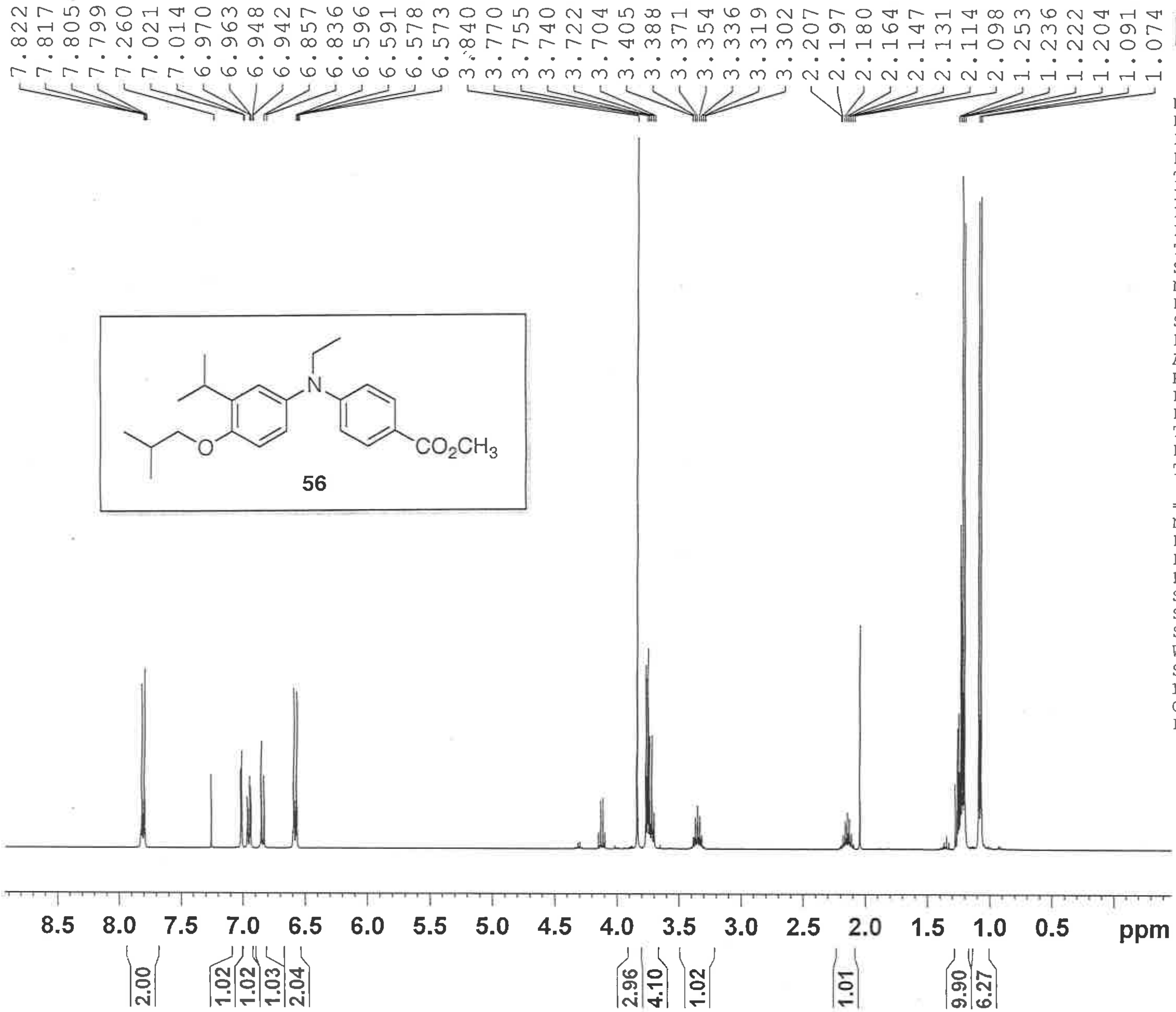

NAME	CWV-227	
EXPNO	1	
PROCNO	1	
Date_	20150420	
Time	12.27	
INSTRUM	spect	
PROBHD	5 mm PABBO BB-	
PULPROG	zg30	
TD	65536	
SOLVENT	CDCl3	
NS	16	
DS	2	
SWH	8223.685	Hz
FIDRES	0.125483	Hz
AQ	3.9846387	sec
RG	4	
DW	60.800	usec
DE	6.50	usec
TE	298.0	K
D1	1.00000000	sec
TD0	1	
======== CHANNEL f1 ========		
NUC1	1H	
P1	14.50	usec
PL1	0.50	dB
PL1W	12.76071072	W
SFO1	400.1324710	MHz
SI	32768	
SF	400.1300096	MHz
WDW	EM	
SSB	0	
LB	0.30	Hz
GB	0	
PC	1.00	

CWV-227

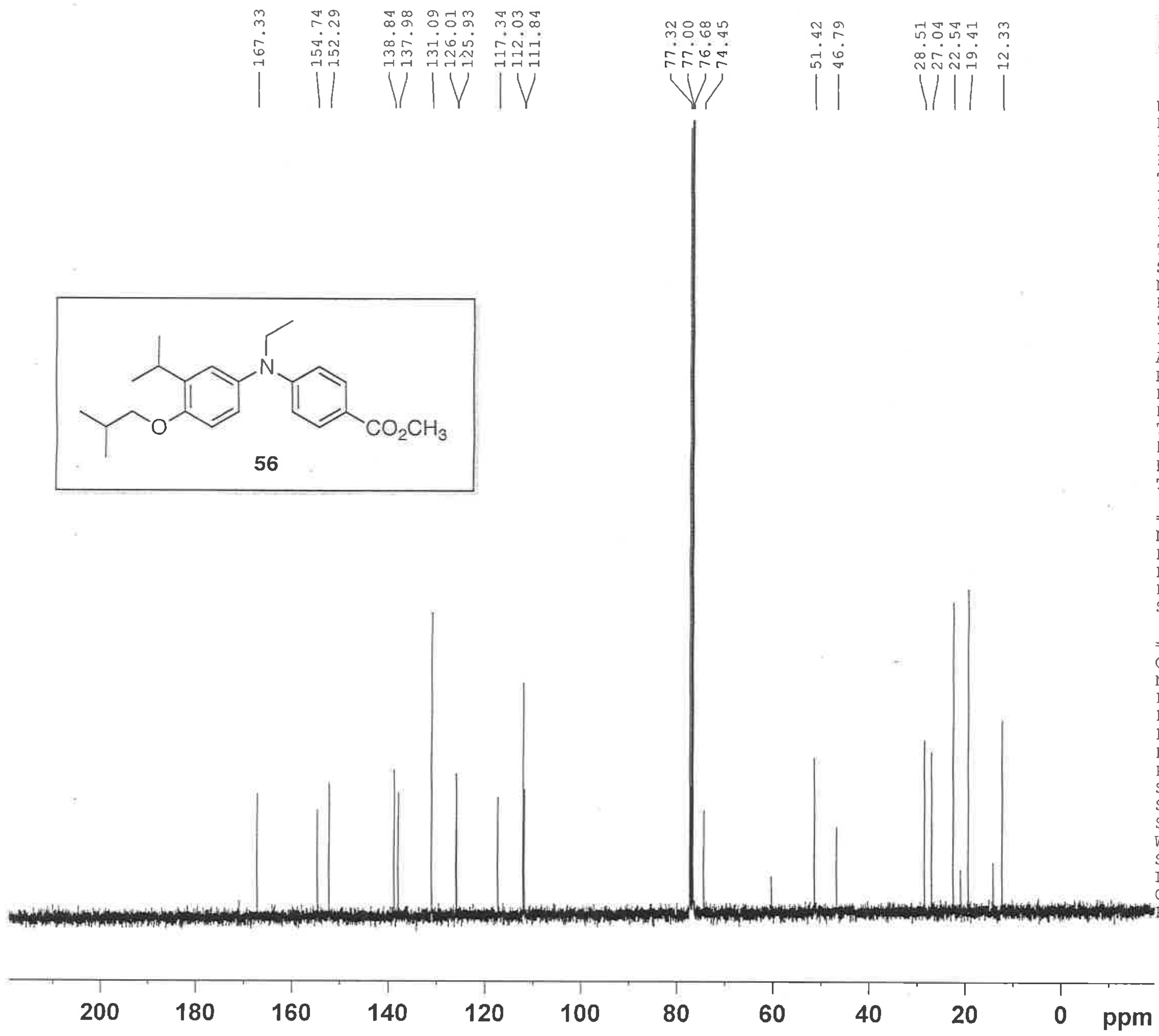

NAME	CWV-227	
EXPNO	2	
PROCNO	1	
Date_	20150420	
Time	12.45	
INSTRUM	spect	
PROBHD	5 mm PABBO BB-	
PULPROG	zgig30	
TD	65536	
SOLVENT	CDCl3	
NS	267	
DS	4	
SWH	24038.461	Hz
FIDRES	0.366798	Hz
AQ	1.3631988	sec
RG	2050	
DW	20.800	usec
DE	6.50	usec
TE	298.4	K
D1	2.00000000	sec
D11	0.03000000	sec
TD0	1	
======== CHANNEL f1 ========		
NUC1	13C	
P1	8.50	usec
PL1	-2.10	dB
PL1W	60.29227829	W
SFO1	100.6228298	MHz
======== CHANNEL f2 ========		
CPDPRG2	waltz16	
NUC2	1H	
PCPD2	90.00	usec
PL2	-1.80	dB
PL12	17.28	dB
PL2W	21.67079544	W
PL12W	0.26783961	W
SFO2	400.1316005	MHz
SI	32768	
SF	100.6127722	MHz
WDW	EM	
SSB	0	
LB	1.00	Hz
GB	0	
PC	1.40	

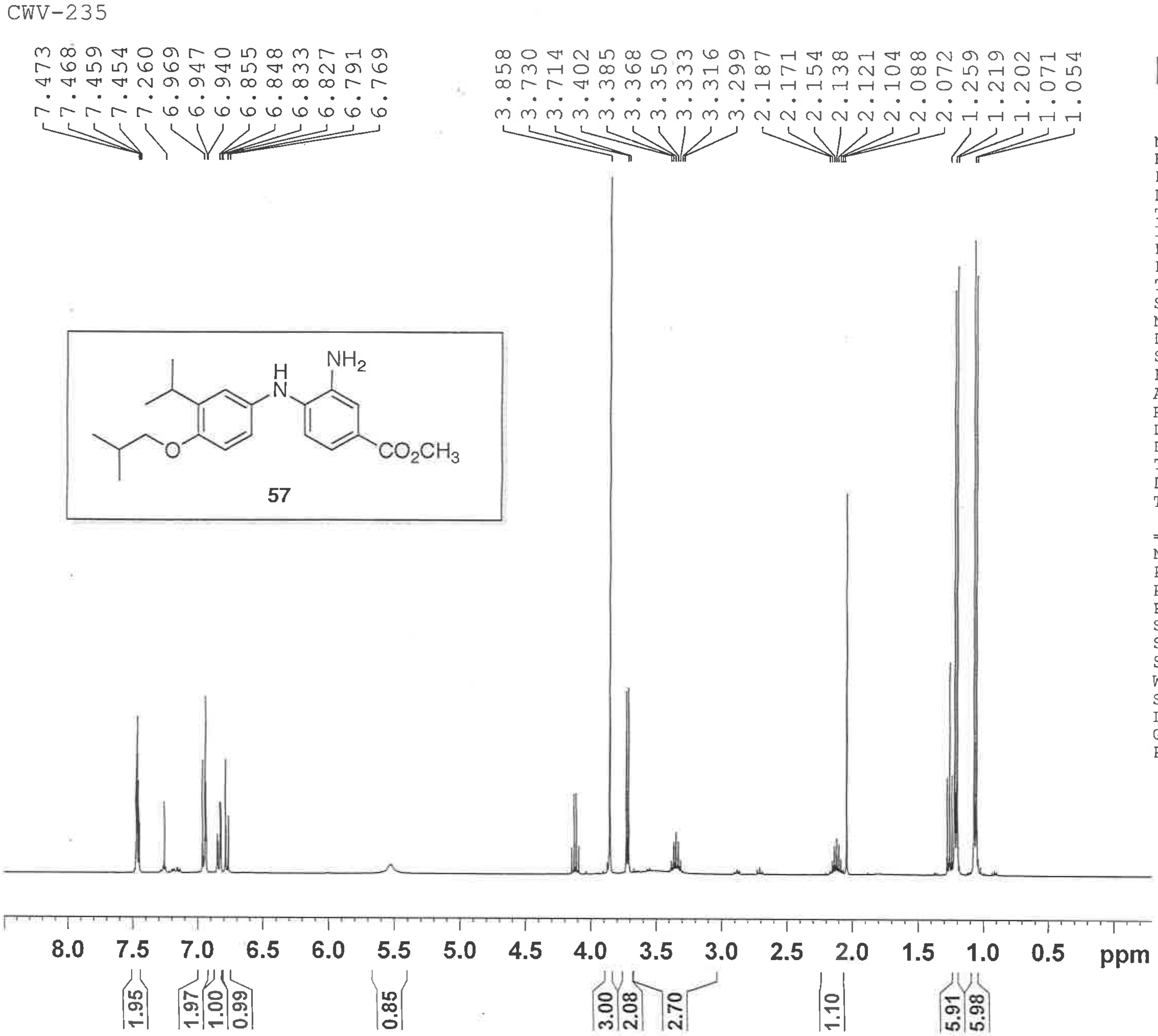

NAME	CWV-235
EXPNO	1
PROCNO	1
Date_	20150706
Time	15.52
INSTRUM	spect
PROBHD	5 mm PABBO BB-
PULPROG	zg30
TD	65536
SOLVENT	CDCl3
NS	16
DS	2
SWH	8223.685 Hz
FIDRES	0.125483 Hz
AQ	3.9846387 sec
RG	4
DW	60.800 usec
DE	6.50 usec
TE	298.2 K
D1	1.00000000 sec
TD0	1

======== CHANNEL f1 ========

NUC1	1H
P1	14.50 usec
PL1	0.50 dB
PL1W	12.76071072 W
SFO1	400.1324710 MHz
SI	32768
SF	400.1300099 MHz
WDW	EM
SSB	0
LB	0.30 Hz
GB	0
PC	1.00

CWV-235

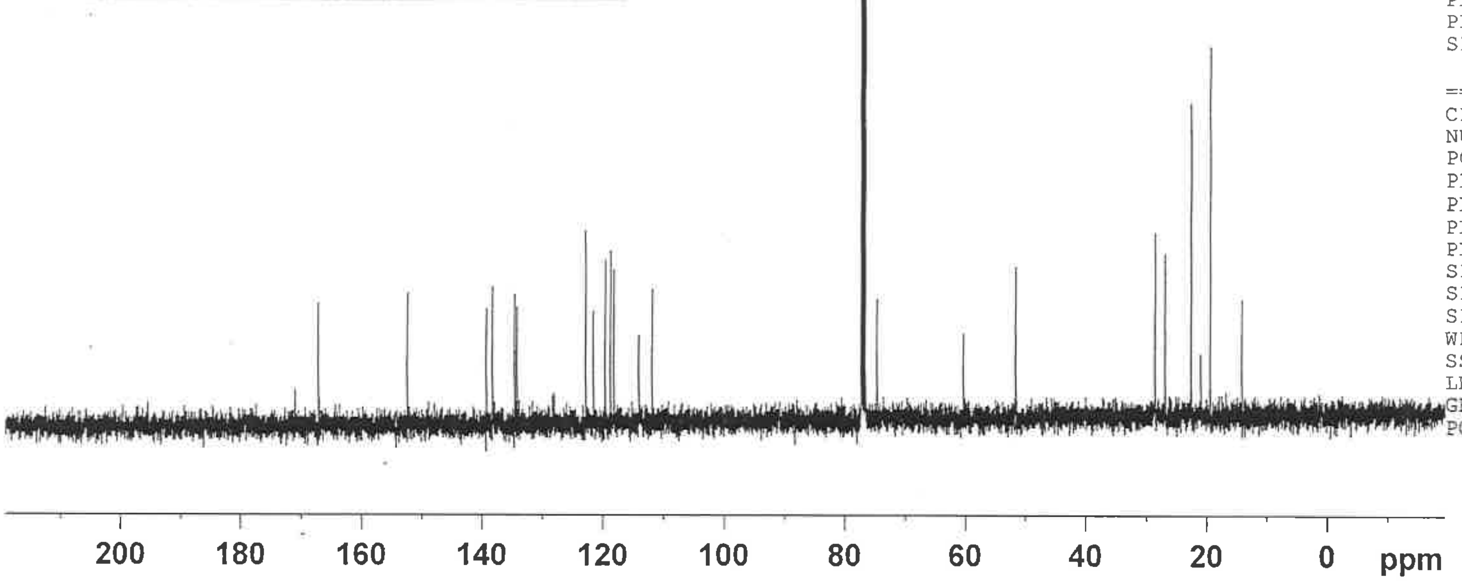

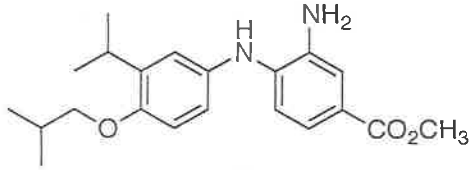

57

BRUKER

NAME	CWV-235
EXPNO	2
PROCNO	1
Date_	20150706
Time	15.59
INSTRUM	spect
PROBHD	5 mm PABBO BB-
PULPROG	zgig30
TD	65536
SOLVENT	CDCl3
NS	118
DS	4
SWH	24038.461 Hz
FIDRES	0.366798 Hz
AQ	1.3631988 sec
RG	2050
DW	20.800 usec
DE	6.50 usec
TE	298.5 K
D1	2.00000000 sec
D11	0.03000000 sec
TD0	1
======== CHANNEL f1 ========	
NUC1	13C
P1	8.50 usec
PL1	-2.10 dB
PL1W	60.29227829 W
SFO1	100.6228298 MHz
======== CHANNEL f2 ========	
CPDPRG2	waltz16
NUC2	1H
PCPD2	90.00 usec
PL2	-1.80 dB
PL12	17.28 dB
PL2W	21.67079544 W
PL12W	0.26783961 W
SFO2	400.1316005 MHz
SI	32768
SF	100.6127729 MHz
WDW	EM
SSB	0
LB	1.00 Hz
GB	0
PC	1.40

CWI-237

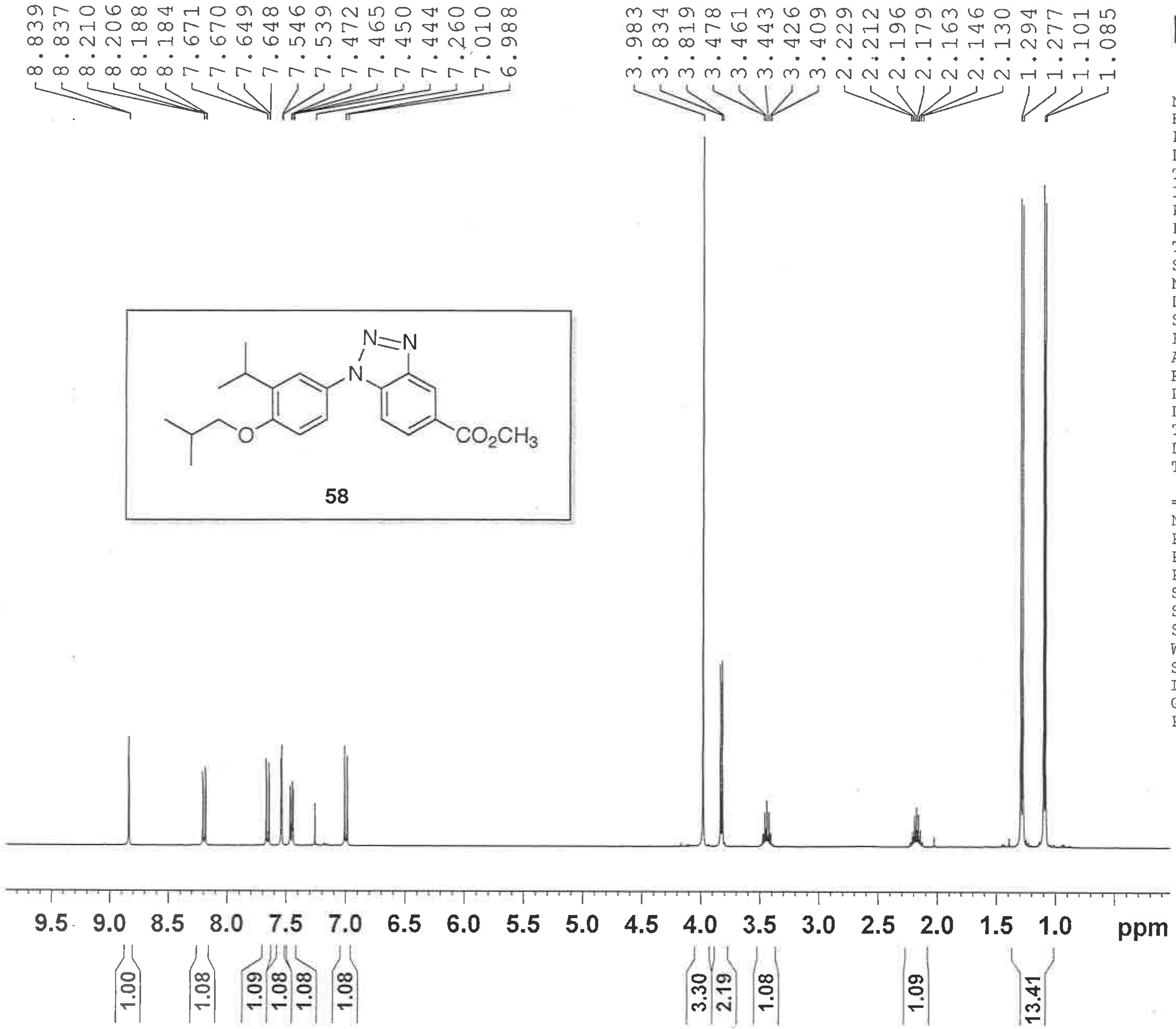

58

NAME	CWV-237
EXPNO	1
PROCNO	1
Date_	20150805
Time	16.05
INSTRUM	spect
PROBHD	5 mm PABBO BB-
PULPROG	zg30
TD	65536
SOLVENT	CDCl3
NS	16
DS	2
SWH	8223.685 Hz
FIDRES	0.125483 Hz
AQ	3.9846387 sec
RG	4
DW	60.800 usec
DE	6.50 usec
TE	298.1 K
D1	1.00000000 sec
TD0	1

======== CHANNEL f1 ========

NUC1	1H
P1	14.50 usec
PL1	0.50 dB
PL1W	12.76071072 W
SFO1	400.1324710 MHz
SI	32768
SF	400.1300096 MHz
WDW	EM
SSB	0
LB	0.30 Hz
GB	0
PC	1.00

CWI-237

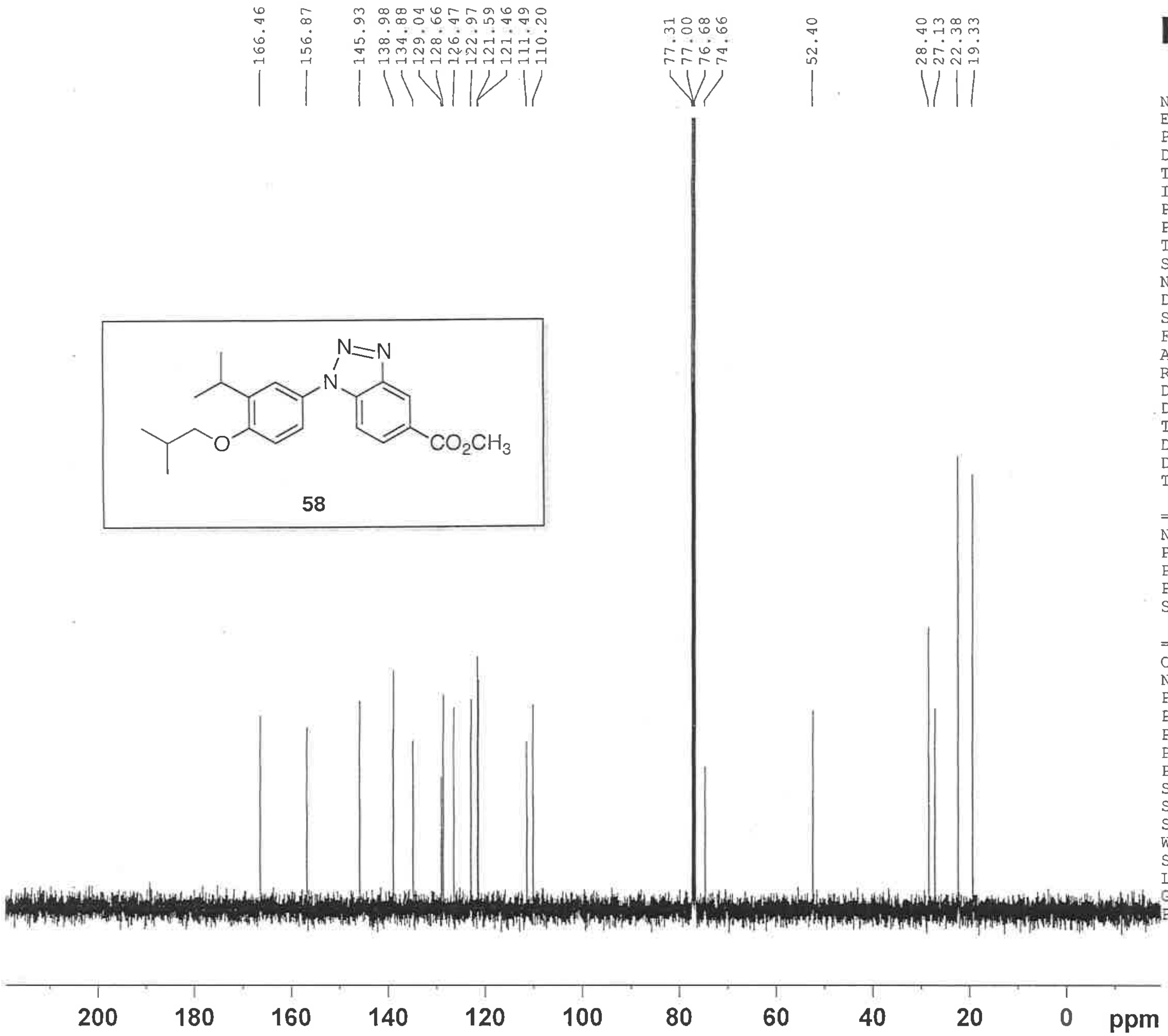

58

BRUKER

NAME	CWV-237
EXPNO	2
PROCNO	1
Date_	20150805
Time	16.20
INSTRUM	spect
PROBHD	5 mm PABBO BB-
PULPROG	zgig30
TD	65536
SOLVENT	CDCl3
NS	63
DS	4
SWH	24038.461 Hz
FIDRES	0.366798 Hz
AQ	1.3631988 sec
RG	2050
DW	20.800 usec
DE	6.50 usec
TE	298.4 K
D1	2.00000000 sec
D11	0.03000000 sec
TD0	1
======== CHANNEL f1 ========	
NUC1	13C
P1	8.50 usec
PL1	-2.10 dB
PL1W	60.29227829 W
SFO1	100.6228298 MHz
======== CHANNEL f2 ========	
CPDPRG2	waltz16
NUC2	1H
PCPD2	90.00 usec
PL2	-1.80 dB
PL12	17.28 dB
PL2W	21.67079544 W
PL12W	0.26783961 W
SFO2	400.1316005 MHz
SI	32768
SF	100.6127744 MHz
WDW	EM
SSB	0
LB	1.00 Hz
GB	0
PC	1.40

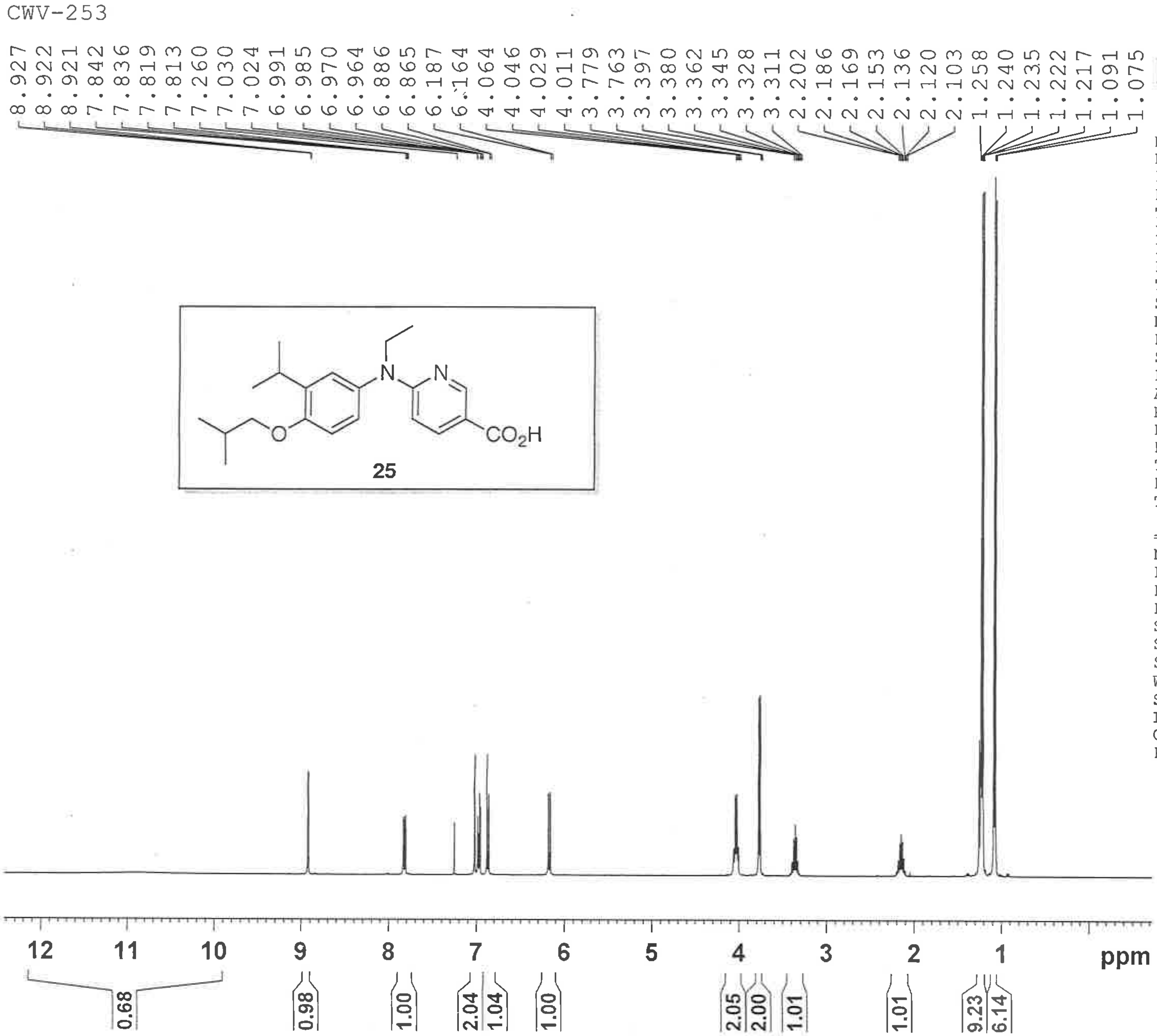

NAME	CWV-253
EXPNO	1
PROCNO	1
Date_	20150930
Time	10.46
INSTRUM	spect
PROBHD	5 mm PABBO BB-
PULPROG	zg30
TD	65536
SOLVENT	CDCl3
NS	16
DS	2
SWH	8223.685 Hz
FIDRES	0.125483 Hz
AQ	3.9846387 sec
RG	4
DW	60.800 usec
DE	6.50 usec
TE	298.1 K
D1	1.00000000 sec
TD0	1

======== CHANNEL f1 ========

NUC1	1H
P1	14.50 usec
PL1	0.50 dB
PL1W	12.76071072 W
SFO1	400.1324710 MHz
SI	32768
SF	400.1300099 MHz
WDW	EM
SSB	0
LB	0.30 Hz
GB	0
PC	1.00

CWV-253

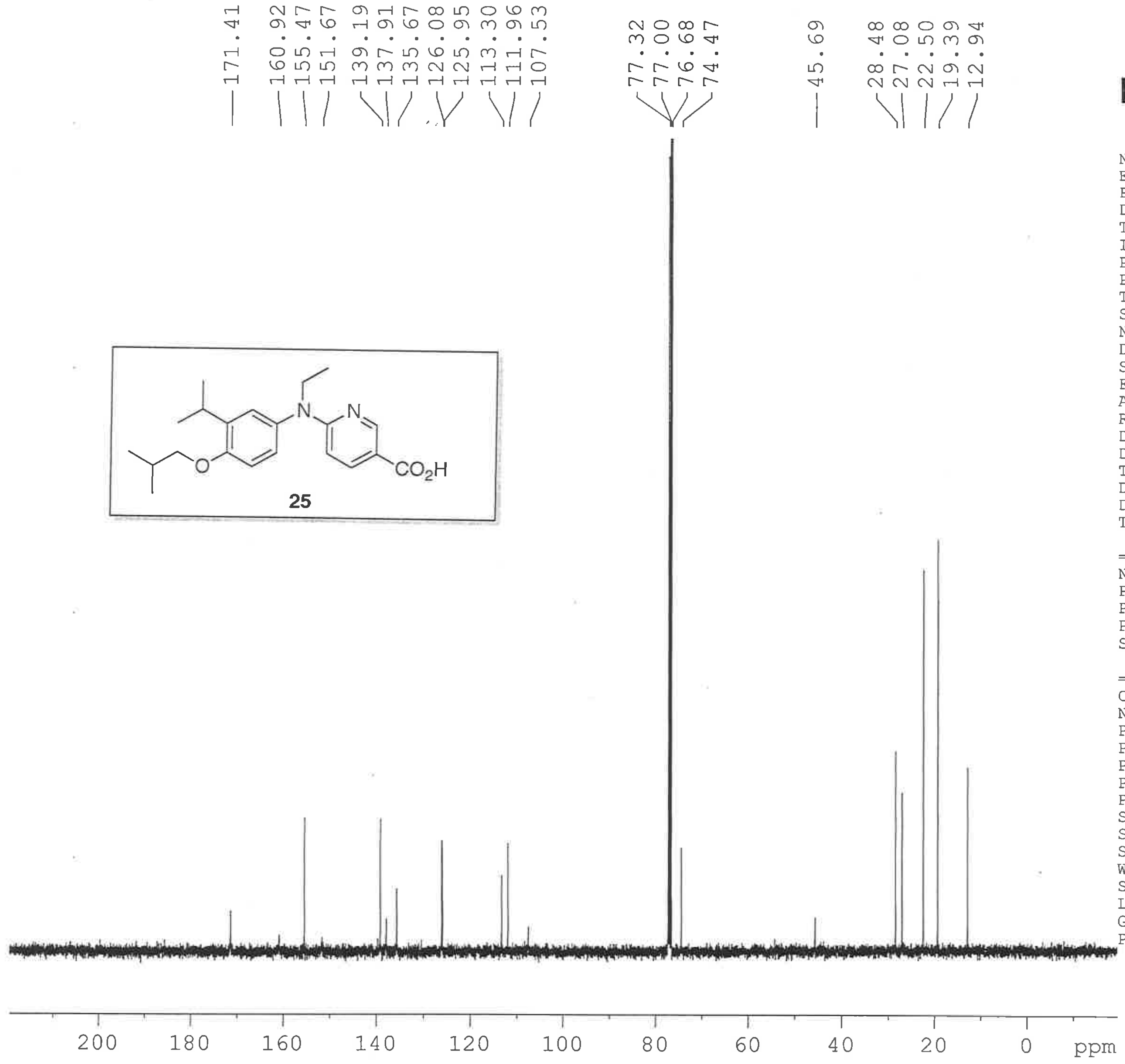

BRUKER

NAME	CWV-253
EXPNO	2
PROCNO	1
Date_	20150930
Time	10.57
INSTRUM	spect
PROBHD	5 mm PABBO BB-
PULPROG	zgig30
TD	65536
SOLVENT	CDCl3
NS	377
DS	4
SWH	24038.461 Hz
FIDRES	0.366798 Hz
AQ	1.3631988 sec
RG	2050
DW	20.800 usec
DE	6.50 usec
TE	298.4 K
D1	2.00000000 sec
D11	0.03000000 sec
TD0	1

======== CHANNEL f1 ========

NUC1	13C
P1	8.50 usec
PL1	-2.10 dB
PL1W	60.29227829 W
SFO1	100.6228298 MHz

======== CHANNEL f2 ========

CPDPRG2	waltz16
NUC2	1H
PCPD2	90.00 usec
PL2	-1.80 dB
PL12	17.28 dB
PL2W	21.67079544 W
PL12W	0.26783961 W
SFO2	400.1316005 MHz
SI	32768
SF	100.6127722 MHz
WDW	EM
SSB	0
LB	1.00 Hz
GB	0
PC	1.40

CWV-259

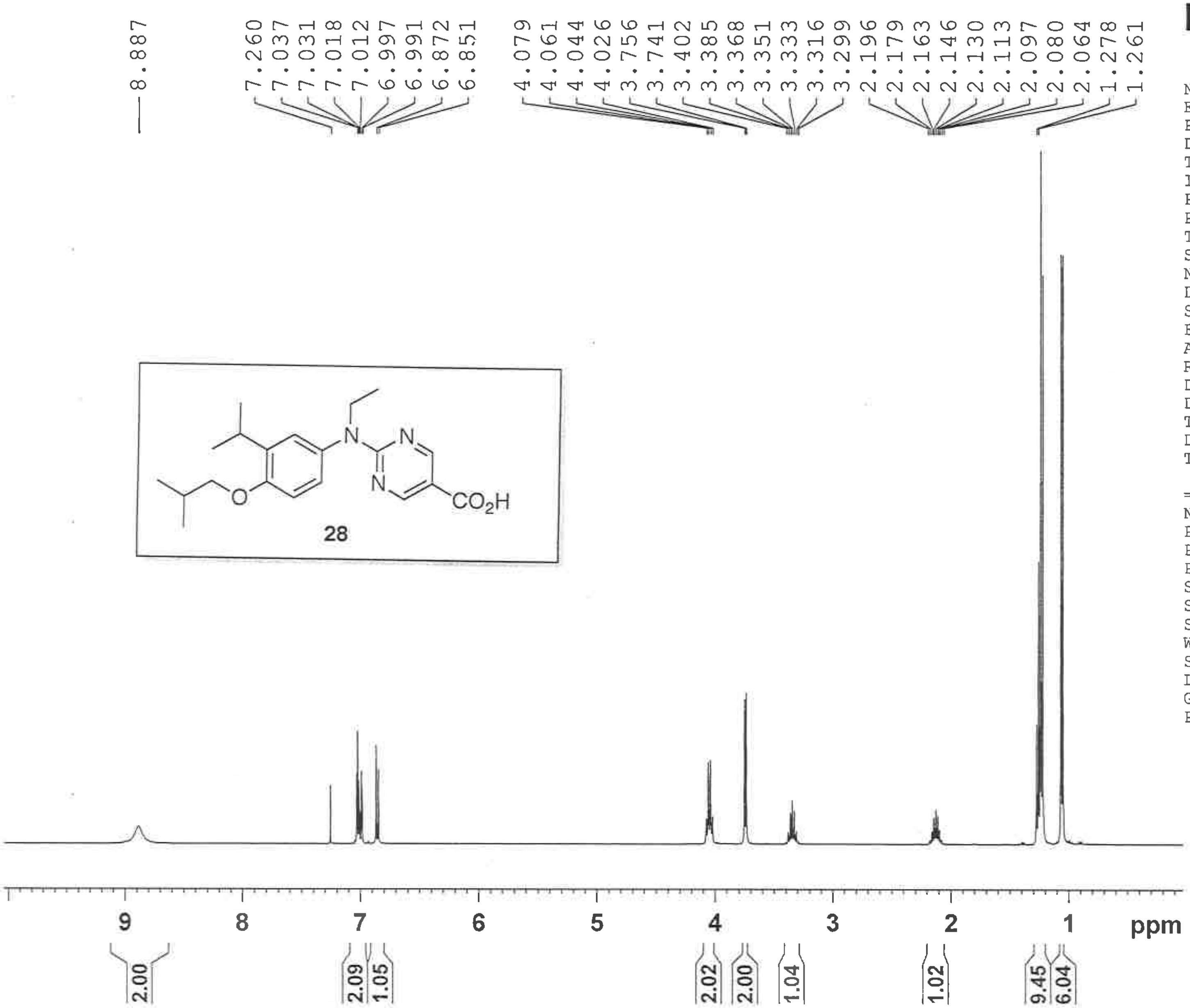

28

NAME	CWV-259
EXPNO	1
PROCNO	1
Date_	20150930
Time_	11.15
INSTRUM	spect
PROBHD	5 mm PABBO BB-
PULPROG	zg30
TD	65536
SOLVENT	CDCl3
NS	16
DS	2
SWH	8223.685 Hz
FIDRES	0.125483 Hz
AQ	3.9846387 sec
RG	4
DW	60.800 usec
DE	6.50 usec
TE	298.0 K
D1	1.00000000 sec
TD0	1
======== CHANNEL f1 ========	
NUC1	1H
P1	14.50 usec
PL1	0.50 dB
PL1W	12.76071072 W
SFO1	400.1324710 MHz
SI	32768
SF	400.1300096 MHz
WDW	EM
SSB	0
LB	0.30 Hz
GB	0
PC	1.00

CWV-259

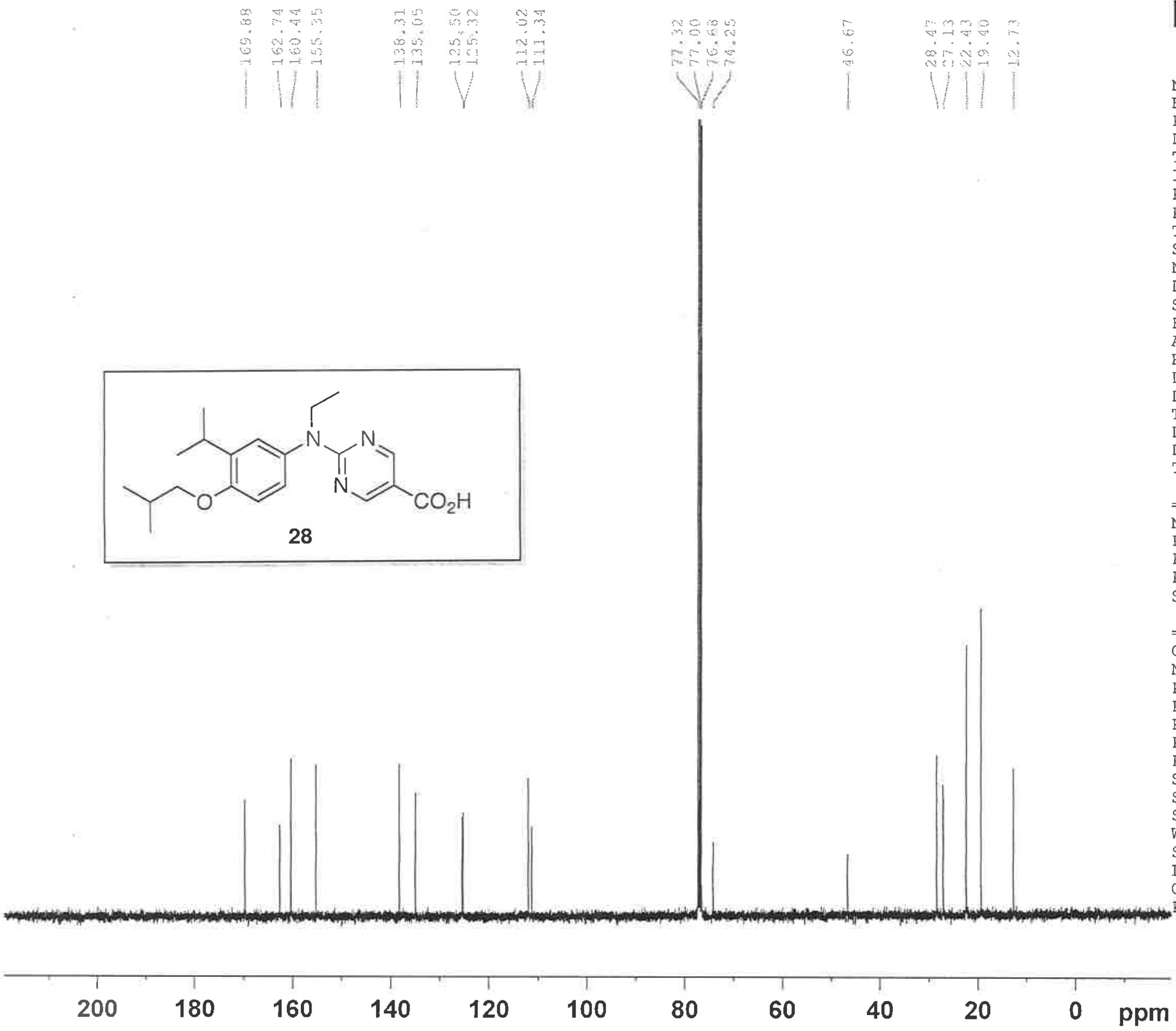

BRUKER

NAME	CWV-259
EXPNO	2
PROCNO	1
Date_	20150930
Time	11.36
INSTRUM	spect
PROBHD	5 mm PABBO BB-
PULPROG	zgig30
TD	65536
SOLVENT	CDCl3
NS	467
DS	4
SWH	24038.461 Hz
FIDRES	0.366798 Hz
AQ	1.3631988 sec
RG	2050
DW	20.800 usec
DE	6.50 usec
TE	298.4 K
D1	2.00000000 sec
D11	0.03000000 sec
TD0	1
======== CHANNEL f1 ========	
NUC1	13C
P1	8.50 usec
PL1	-2.10 dB
PL1W	60.29227829 W
SFO1	100.6228298 MHz
======== CHANNEL f2 ========	
CPDPRG2	waltz16
NUC2	1H
PCPD2	90.00 usec
PL2	-1.80 dB
PL12	17.28 dB
PL2W	21.67079544 W
PL12W	0.26783961 W
SFO2	400.1316005 MHz
SI	32768
SF	100.6127722 MHz
WDW	EM
SSB	0
LB	1.00 Hz
GB	0
PC	1.40

CWV-265

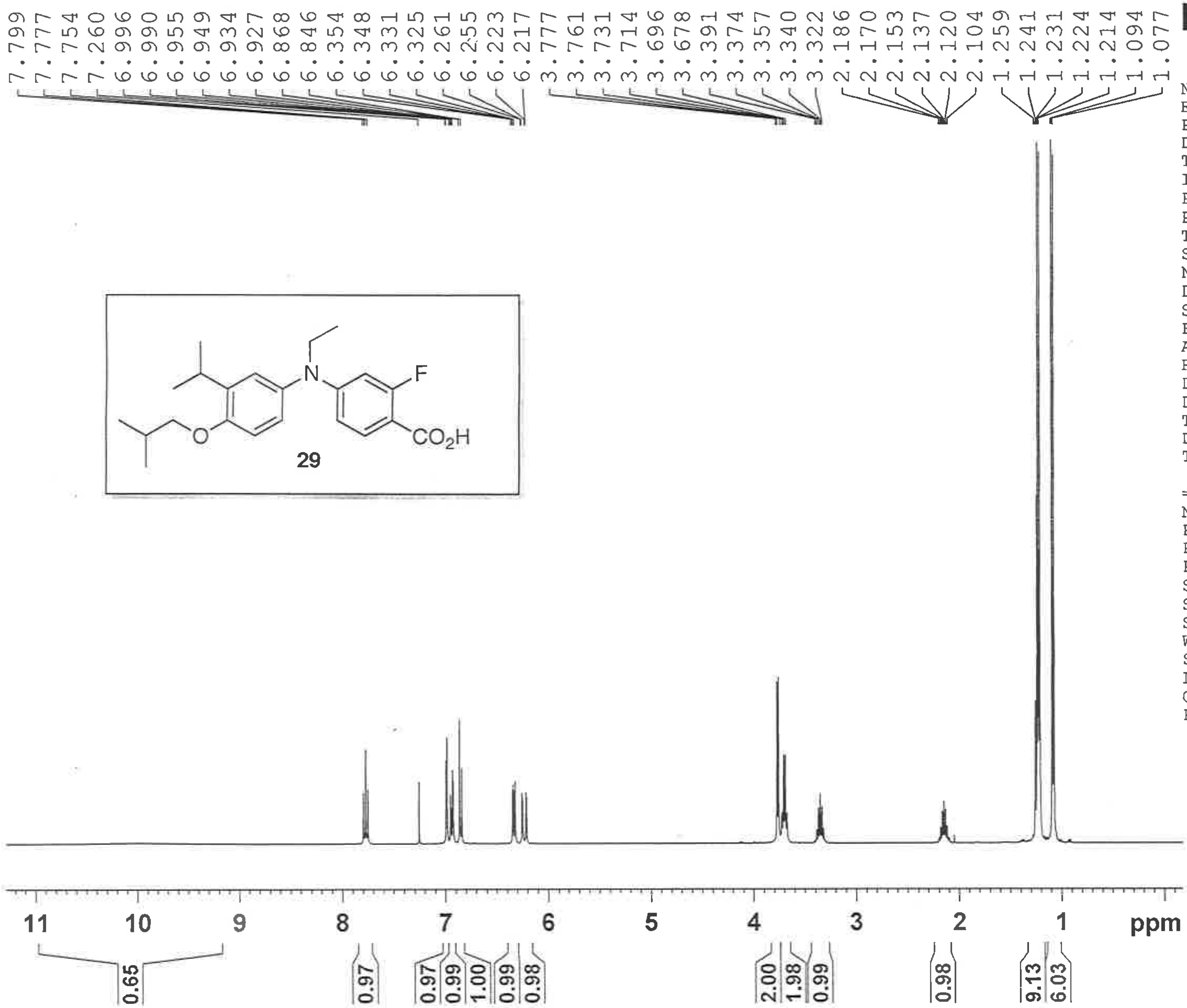

NAME	CWV-265
EXPNO	1
PROCNO	1
Date_	20150930
Time	12.28
INSTRUM	spect
PROBHD	5 mm PABBO BB-
PULPROG	zg30
TD	65536
SOLVENT	CDCl3
NS	16
DS	2
SWH	8223.685 Hz
FIDRES	0.125483 Hz
AQ	3.9846387 sec
RG	4
DW	60.800 usec
DE	6.50 usec
TE	298.1 K
D1	1.00000000 sec
TD0	1

======== CHANNEL f1 ========

NUC1	1H
P1	14.50 usec
PL1	0.50 dB
PL1W	12.76071072 W
SFO1	400.1324710 MHz
SI	32768
SF	400.1300099 MHz
WDW	EM
SSB	0
LB	0.30 Hz
GB	0
PC	1.00

CWV-265

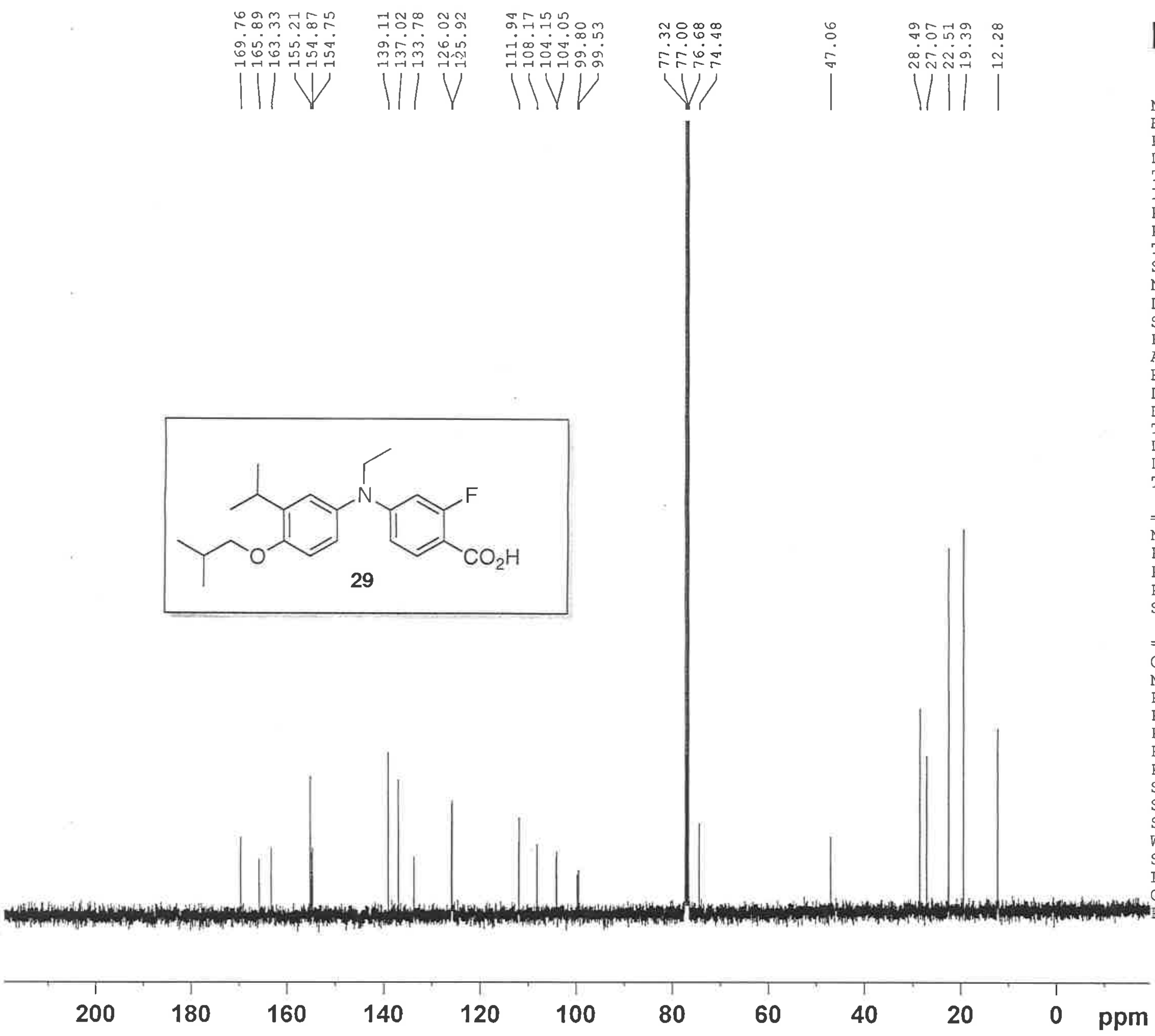

NAME	CWV-265
EXPNO	2
PROCNO	1
Date_	20150930
Time	12.37
INSTRUM	spect
PROBHD	5 mm PABBO BB-
PULPROG	zgig30
TD	65536
SOLVENT	CDCl3
NS	207
DS	4
SWH	24038.461 Hz
FIDRES	0.366798 Hz
AQ	1.3631988 sec
RG	2050
DW	20.800 usec
DE	6.50 usec
TE	298.4 K
D1	2.00000000 sec
D11	0.03000000 sec
TD0	1
======== CHANNEL f1 ========	
NUC1	13C
P1	8.50 usec
PL1	-2.10 dB
PL1W	60.29227829 W
SFO1	100.6228298 MHz
======== CHANNEL f2 ========	
CPDPRG2	waltz16
NUC2	1H
PCPD2	90.00 usec
PL2	-1.80 dB
PL12	17.28 dB
PL2W	21.67079544 W
PL12W	0.26783961 W
SFO2	400.1316005 MHz
SI	32768
SF	100.6127722 MHz
WDW	EM
SSB	0
LB	1.00 Hz
GB	0
PC	1.40

CWV-263

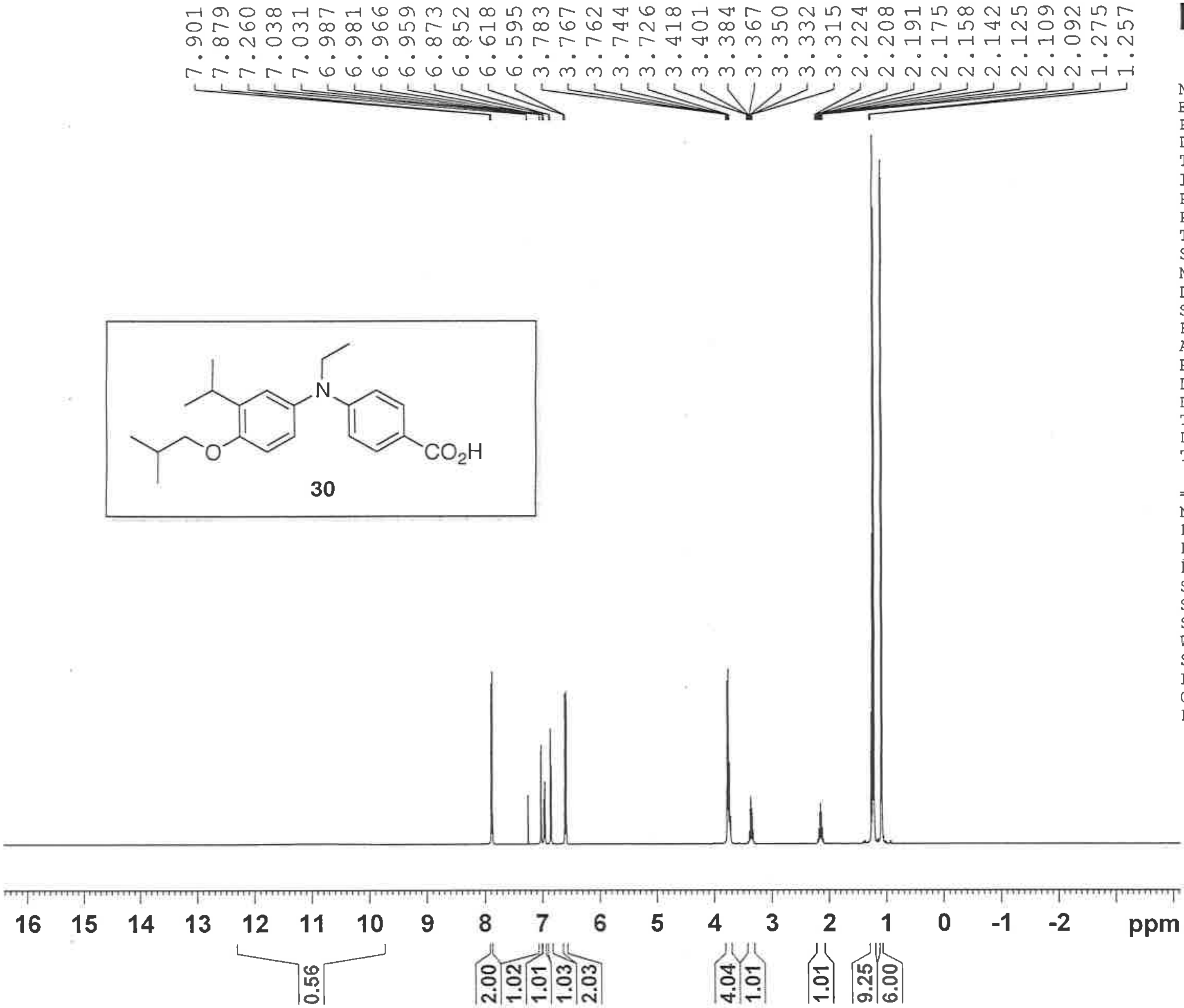

NAME	CWV-263
EXPNO	1
PROCNO	1
Date_	20150930
Time	11.54
INSTRUM	spect
PROBHD	5 mm PABBO BB-
PULPROG	zg30
TD	65536
SOLVENT	CDCl3
NS	16
DS	2
SWH	8223.685 Hz
FIDRES	0.125483 Hz
AQ	3.9846387 sec
RG	4
DW	60.800 usec
DE	6.50 usec
TE	297.9 K
D1	1.00000000 sec
TD0	1

======== CHANNEL f1 ========

NUC1	1H
P1	14.50 usec
PL1	0.50 dB
PL1W	12.76071072 W
SFO1	400.1324710 MHz
SI	32768
SF	400.1300099 MHz
WDW	EM
SSB	0
LB	0.30 Hz
GB	0
PC	1.00

CWV-263

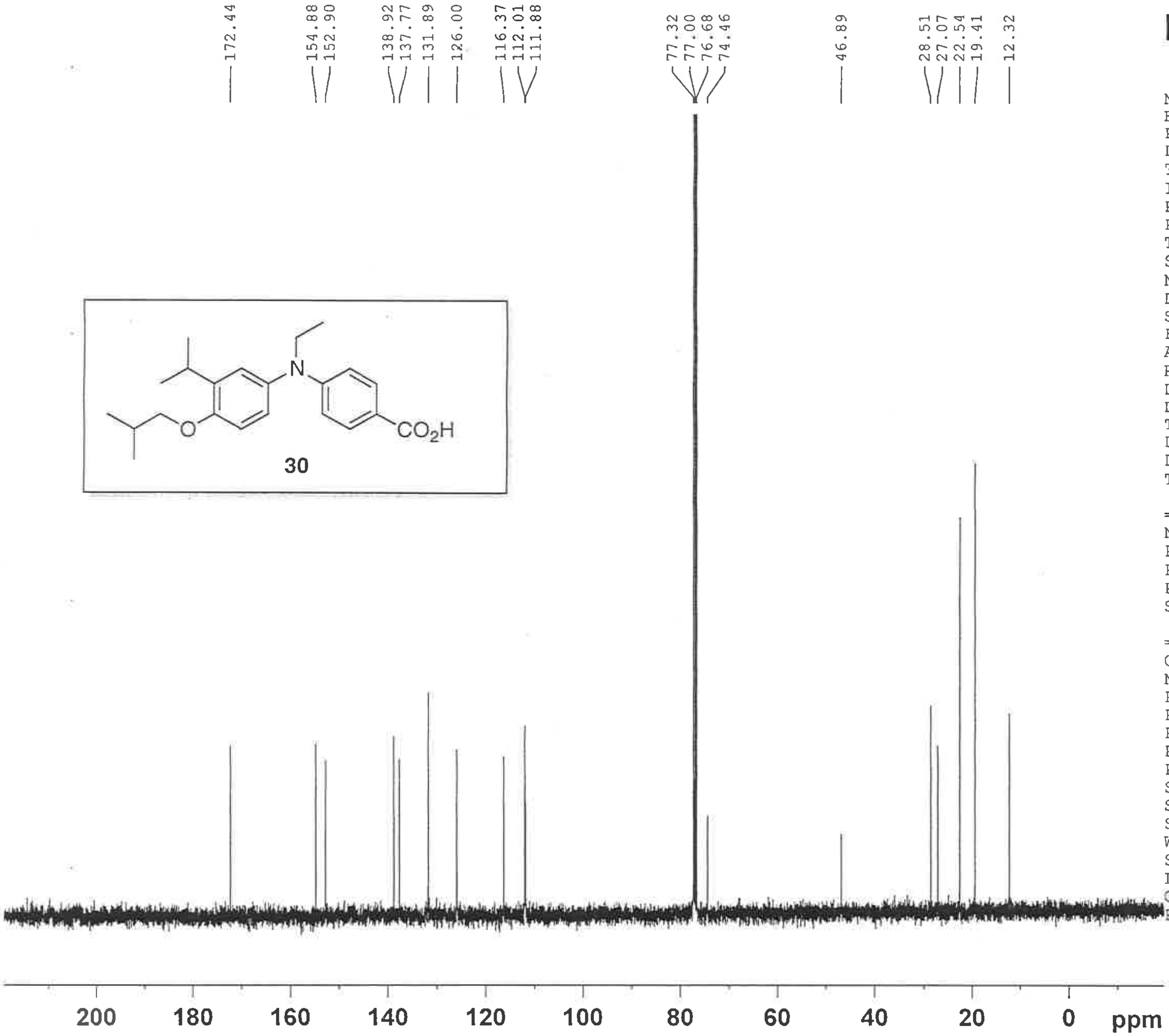

30

BRUKER

NAME	CWV-263
EXPNO	3
PROCNO	1
Date_	20150930
Time	12.13
INSTRUM	spect
PROBHD	5 mm PABBO BB-
PULPROG	zgig30
TD	65536
SOLVENT	CDCl3
NS	155
DS	4
SWH	24038.461 Hz
FIDRES	0.366798 Hz
AQ	1.3631988 sec
RG	2050
DW	20.800 usec
DE	6.50 usec
TE	298.2 K
D1	2.00000000 sec
D11	0.03000000 sec
TD0	1
======== CHANNEL f1 ========	
NUC1	13C
P1	8.50 usec
PL1	-2.10 dB
PL1W	60.29227829 W
SFO1	100.6228298 MHz
======== CHANNEL f2 ========	
CPDPRG2	waltz16
NUC2	1H
PCPD2	90.00 usec
PL2	-1.80 dB
PL12	17.28 dB
PL2W	21.67079544 W
PL12W	0.26783961 W
SFO2	400.1316005 MHz
SI	32768
SF	100.6127729 MHz
WDW	EM
SSB	0
LB	1.00 Hz
GB	0
PC	1.40

CWV-267

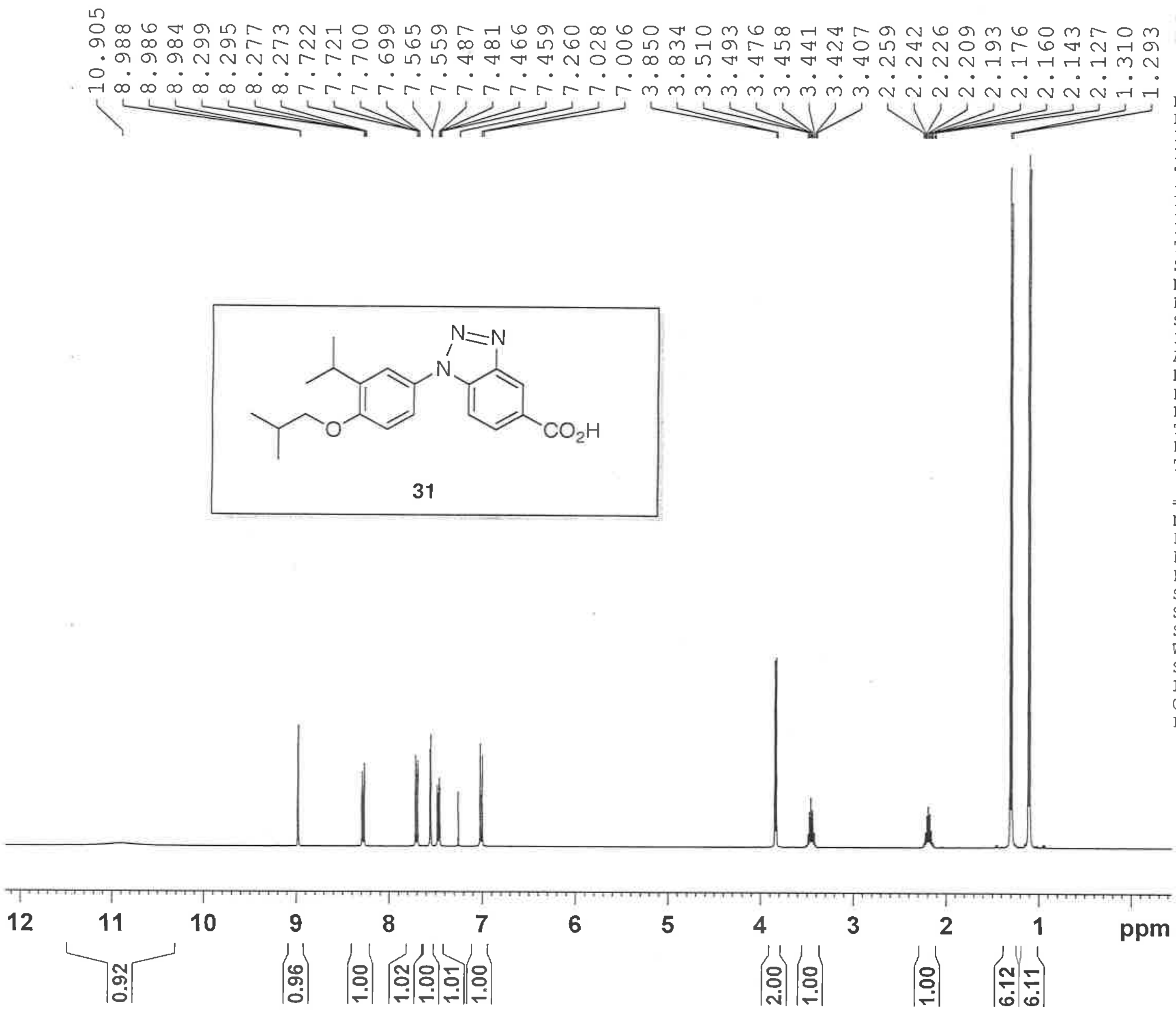

BRUKER

NAME	CWV-267	
EXPNO	1	
PROCNO	1	
Date_	20150930	
Time	12.47	
INSTRUM	spect	
PROBHD	5 mm PABBO BB-	
PULPROG	zg30	
TD	65536	
SOLVENT	CDCl3	
NS	16	
DS	2	
SWH	8223.685	Hz
FIDRES	0.125483	Hz
AQ	3.9846387	sec
RG	4	
DW	60.800	usec
DE	6.50	usec
TE	298.1	K
D1	1.00000000	sec
TD0	1	

======== CHANNEL f1 ========

NUC1	1H	
P1	14.50	usec
PL1	0.50	dB
PL1W	12.76071072	W
SFO1	400.1324710	MHz
SI	32768	
SF	400.1300096	MHz
WDW	EM	
SSB	0	
LB	0.30	Hz
GB	0	
PC	1.00	

CWV-267

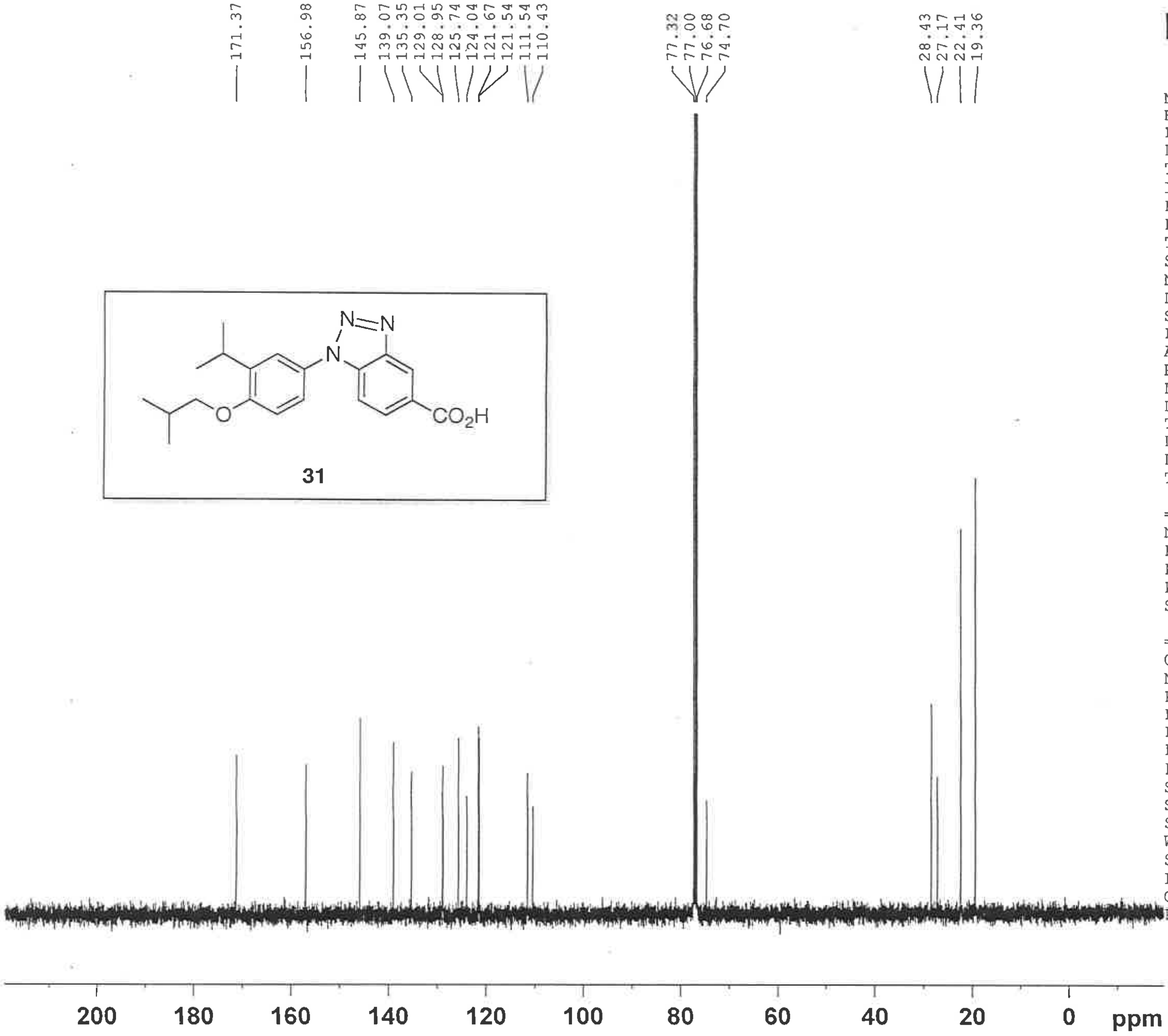

31

BRUKER

NAME	CWV-267
EXPNO	2
PROCNO	1
Date_	20150930
Time	12.55
INSTRUM	spect
PROBHD	5 mm PABBO BB-
PULPROG	zgig30
TD	65536
SOLVENT	CDCl3
NS	148
DS	4
SWH	24038.461 Hz
FIDRES	0.366798 Hz
AQ	1.3631988 sec
RG	2050
DW	20.800 usec
DE	6.50 usec
TE	298.4 K
D1	2.00000000 sec
D11	0.03000000 sec
TD0	1

======== CHANNEL f1 ========

NUC1	13C
P1	8.50 usec
PL1	-2.10 dB
PL1W	60.29227829 W
SFO1	100.6228298 MHz

======== CHANNEL f2 ========

CPDPRG2	waltz16
NUC2	1H
PCPD2	90.00 usec
PL2	-1.80 dB
PL12	17.28 dB
PL2W	21.67079544 W
PL12W	0.26783961 W
SFO2	400.1316005 MHz
SI	32768
SF	100.6127729 MHz
WDW	EM
SSB	0
LB	1.00 Hz
GB	0
PC	1.40

CWV-247

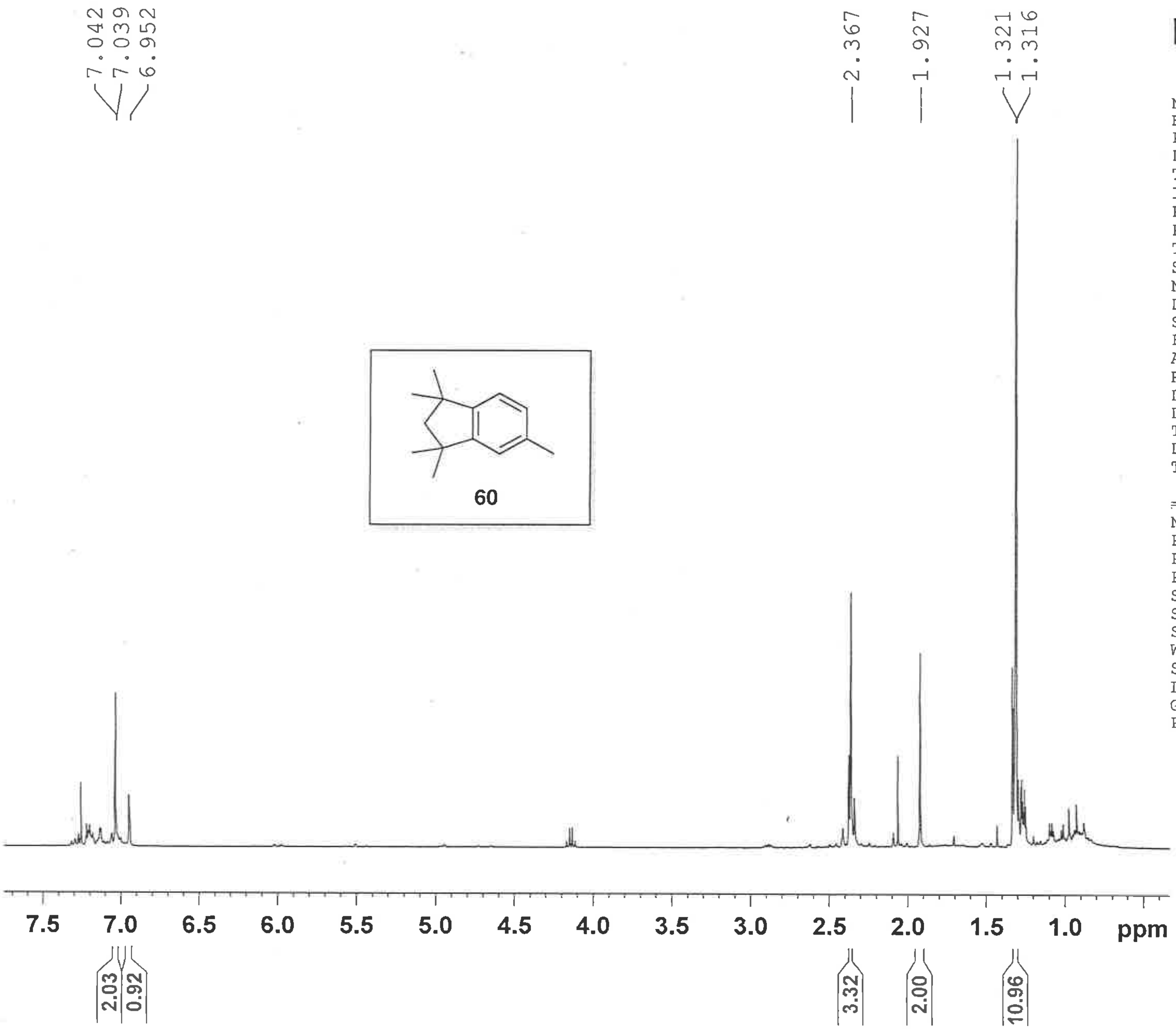

NAME	CWV-247
EXPNO	1
PROCNO	1
Date_	20150723
Time	12.22
INSTRUM	spect
PROBHD	5 mm PABBO BB-
PULPROG	zg30
TD	65536
SOLVENT	CDCl3
NS	16
DS	2
SWH	8223.685 Hz
FIDRES	0.125483 Hz
AQ	3.9846387 sec
RG	4
DW	60.800 usec
DE	6.50 usec
TE	298.2 K
D1	1.00000000 sec
TD0	1

======== CHANNEL f1 ========

NUC1	1H
P1	14.50 usec
PL1	0.50 dB
PL1W	12.76071072 W
SFO1	400.1324710 MHz
SI	32768
SF	400.1300096 MHz
WDW	EM
SSB	0
LB	0.30 Hz
GB	0
PC	1.00

CWV-249 f7-11

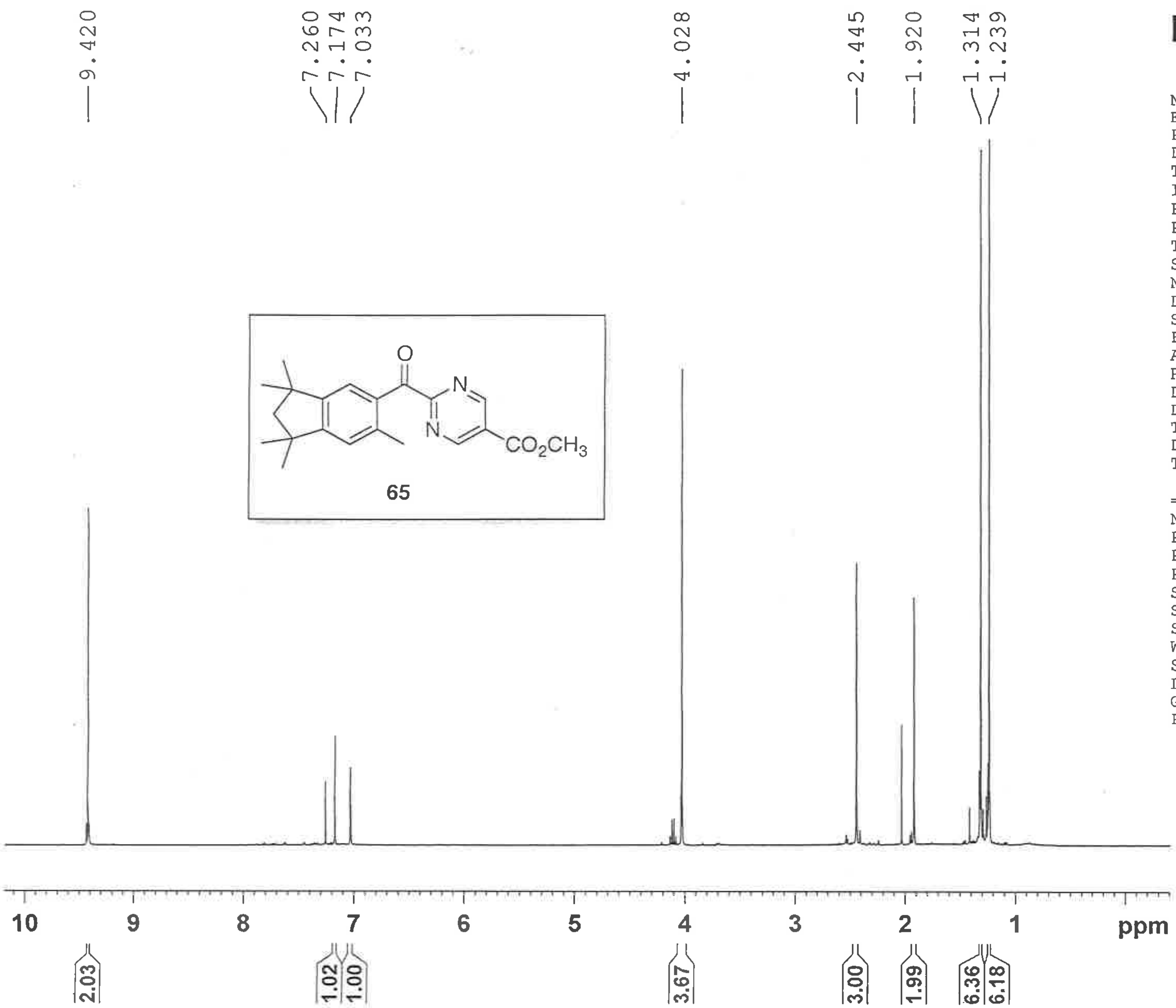

NAME	CWV-249
EXPNO	1
PROCNO	1
Date_	20150730
Time	10.31
INSTRUM	spect
PROBHD	5 mm PABBO BB-
PULPROG	zg30
TD	65536
SOLVENT	CDCl3
NS	16
DS	2
SWH	8223.685 Hz
FIDRES	0.125483 Hz
AQ	3.9846387 sec
RG	4
DW	60.800 usec
DE	6.50 usec
TE	298.2 K
D1	1.00000000 sec
TD0	1

======== CHANNEL f1 ========

NUC1	1H
P1	14.50 usec
PL1	0.50 dB
PL1W	12.76071072 W
SFO1	400.1324710 MHz
SI	32768
SF	400.1300099 MHz
WDW	EM
SSB	0
LB	0.30 Hz
GB	0
PC	1.00

CWV-249 f7-11

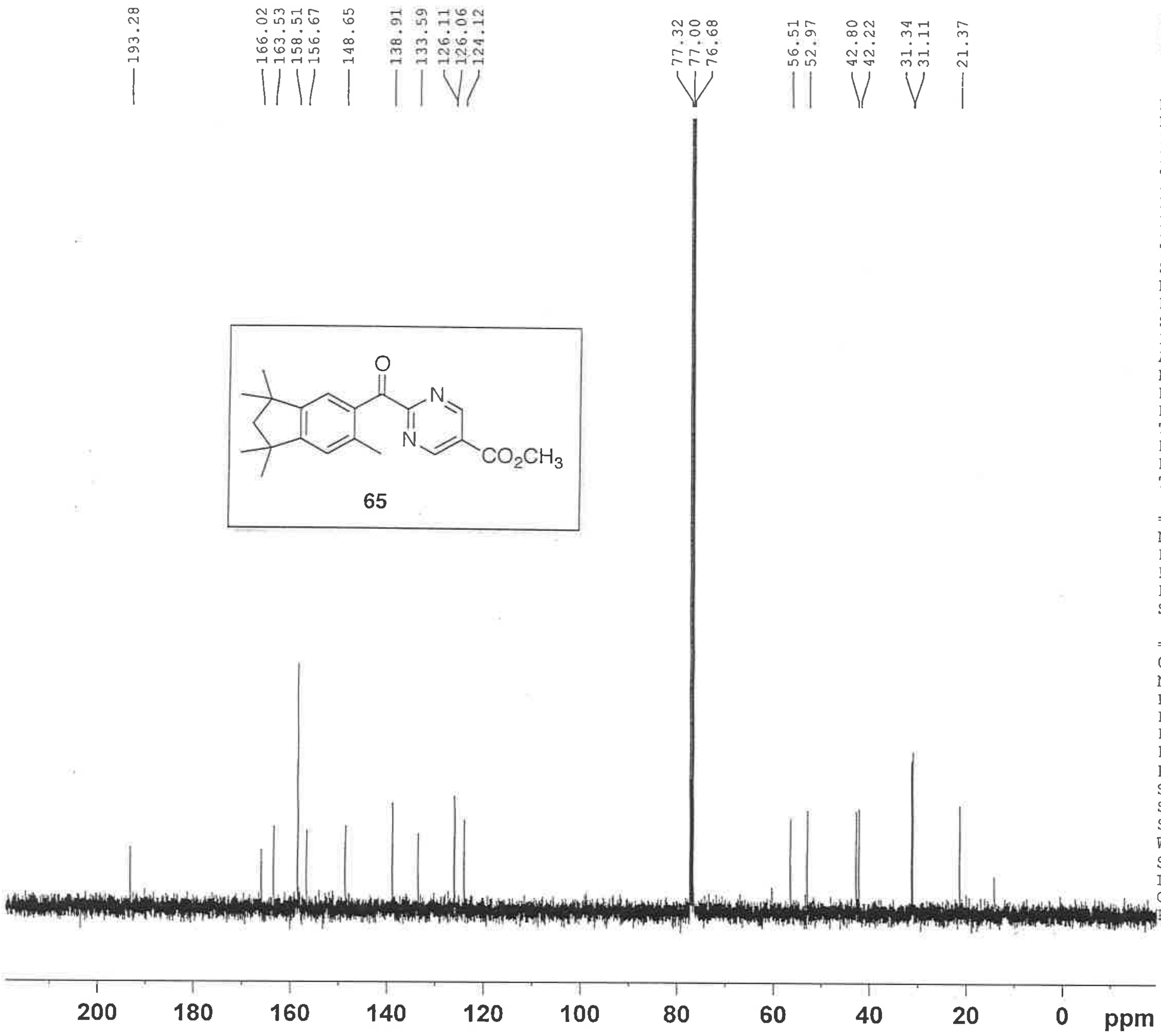

65

BRUKER

NAME	CWV-249
EXPNO	2
PROCNO	1
Date_	20150730
Time	10.40
INSTRUM	spect
PROBHD	5 mm PABBO BB-
PULPROG	zgig30
TD	65536
SOLVENT	CDCl3
NS	110
DS	4
SWH	24038.461 Hz
FIDRES	0.366798 Hz
AQ	1.3631988 sec
RG	2050
DW	20.800 usec
DE	6.50 usec
TE	298.4 K
D1	2.00000000 sec
D11	0.03000000 sec
TD0	1

======== CHANNEL f1 ========

NUC1	13C
P1	8.50 usec
PL1	-2.10 dB
PL1W	60.29227829 W
SFO1	100.6228298 MHz

======== CHANNEL f2 ========

CPDPRG2	waltz16
NUC2	1H
PCPD2	90.00 usec
PL2	-1.80 dB
PL12	17.28 dB
PL2W	21.67079544 W
PL12W	0.26783961 W
SFO2	400.1316005 MHz
SI	32768
SF	100.6127729 MHz
WDW	EM
SSB	0
LB	1.00 Hz
GB	0
PC	1.40

CWVI-027 f4

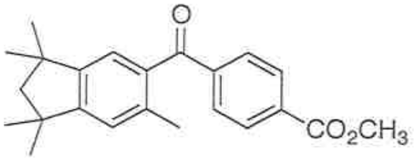

66

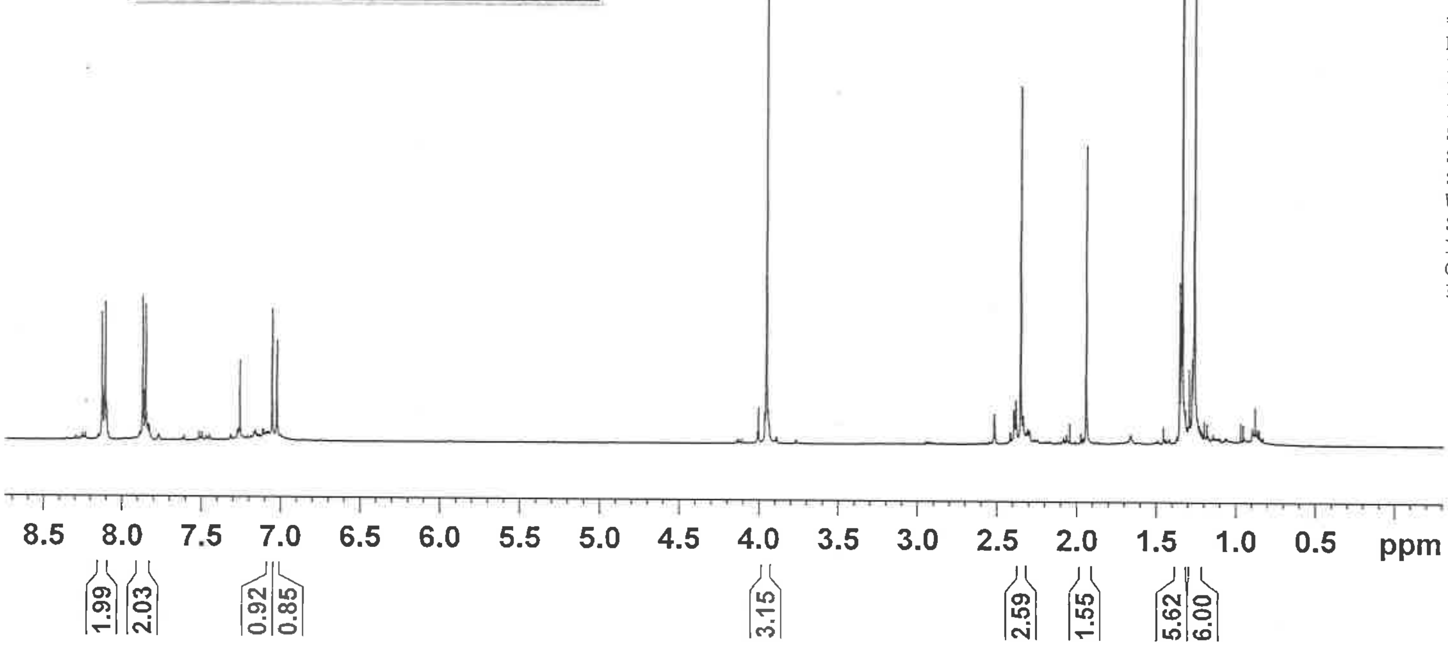

BRUKER

NAME	CWVI-027
EXPNO	1
PROCNO	1
Date_	20160520
Time	12.05
INSTRUM	spect
PROBHD	5 mm PABBO BB-
PULPROG	zg30
TD	65536
SOLVENT	CDCl3
NS	16
DS	2
SWH	8223.685 Hz
FIDRES	0.125483 Hz
AQ	3.9846387 sec
RG	4
DW	60.800 usec
DE	6.50 usec
TE	673.2 K
D1	1.00000000 sec
TD0	1

======== CHANNEL f1 ========

NUC1	1H
P1	14.50 usec
PL1	0.50 dB
PL1W	12.76071072 W
SFO1	400.1324710 MHz
SI	32768
SF	400.1300101 MHz
WDW	EM
SSB	0
LB	0.30 Hz
GB	0
PC	1.00

CWVI-027 f4

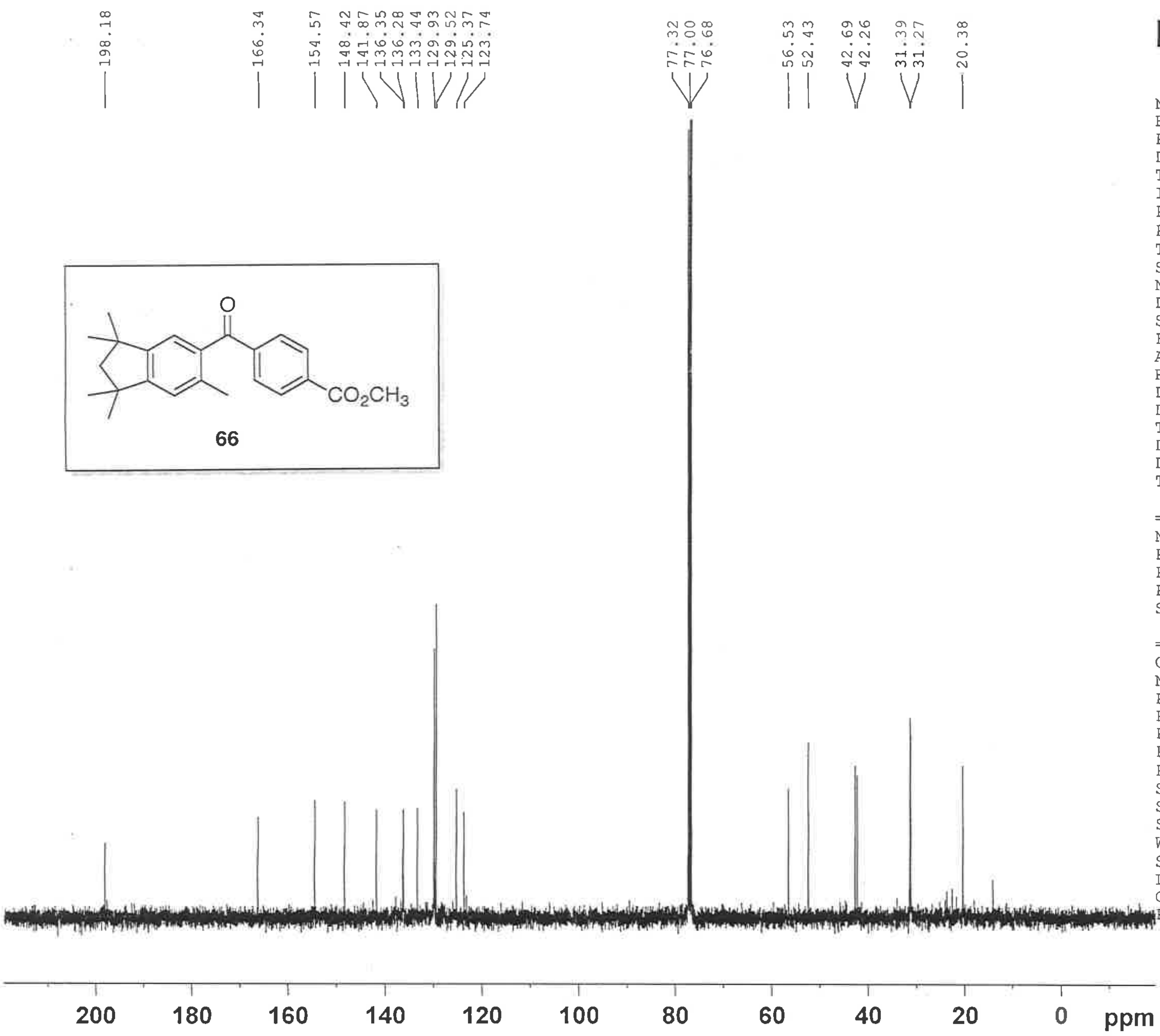

BRUKER

NAME	CWVI-027
EXPNO	2
PROCNO	1
Date_	20160520
Time	12.15
INSTRUM	spect
PROBHD	5 mm PABBO BB-
PULPROG	zgig30
TD	65536
SOLVENT	CDCl3
NS	141
DS	4
SWH	24038.461 Hz
FIDRES	0.366798 Hz
AQ	1.3631988 sec
RG	2050
DW	20.800 usec
DE	6.50 usec
TE	673.2 K
D1	2.00000000 sec
D11	0.03000000 sec
TD0	1
======== CHANNEL f1 ========	
NUC1	13C
P1	8.50 usec
PL1	-2.10 dB
PL1W	60.29227829 W
SFO1	100.6228298 MHz
======== CHANNEL f2 ========	
CPDPRG2	waltz16
NUC2	1H
PCPD2	90.00 usec
PL2	-1.80 dB
PL12	17.28 dB
PL2W	21.67079544 W
PL12W	0.26783961 W
SFO2	400.1316005 MHz
SI	32768
SF	100.6127751 MHz
WDW	EM
SSB	0
LB	1.00 Hz
GB	0
PC	1.40

CWVI-031

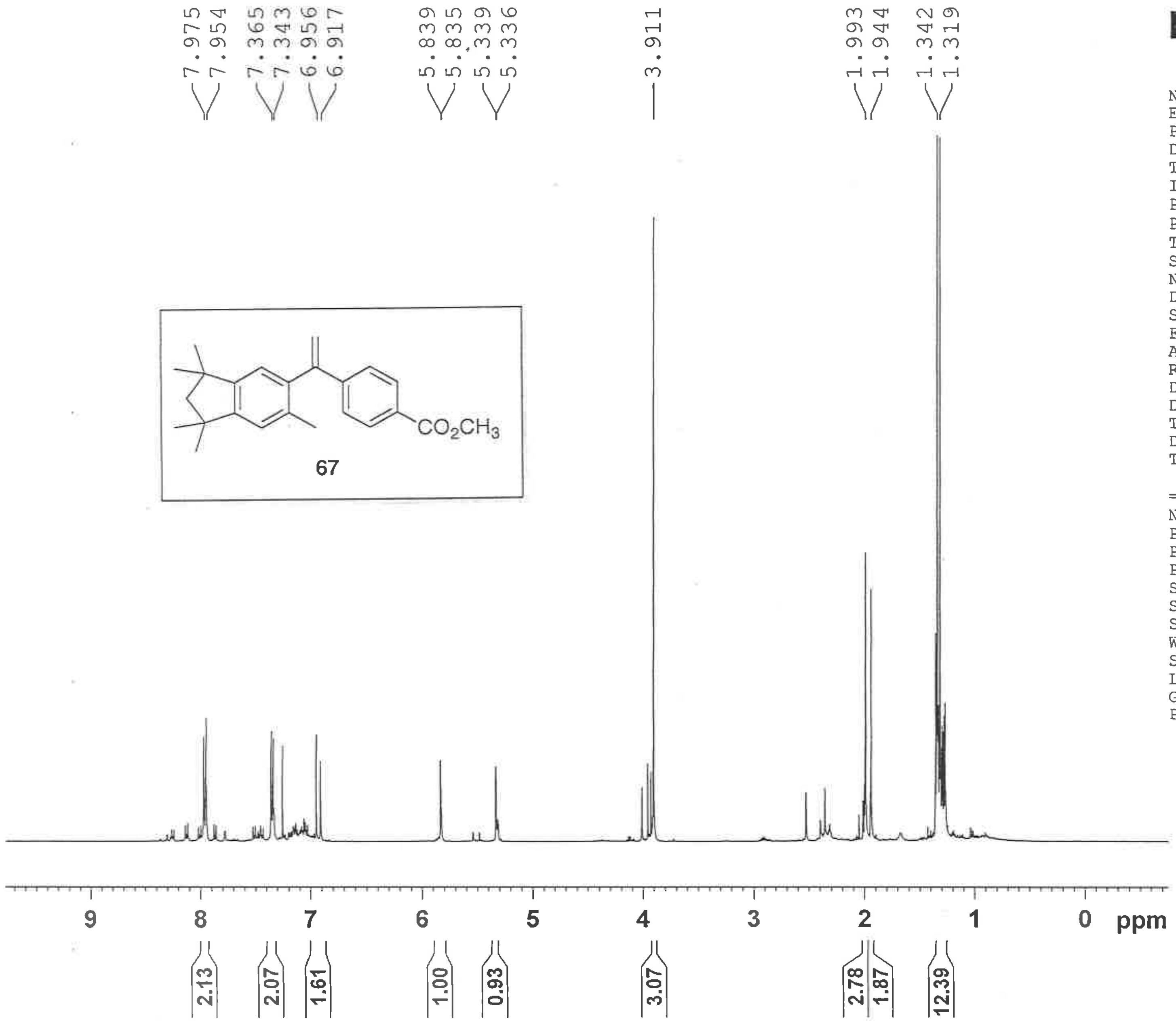

67

BRUKER

NAME	CWVI-031
EXPNO	1
PROCNO	1
Date_	20160718
Time	12.25
INSTRUM	spect
PROBHD	5 mm PABBO BB-
PULPROG	zg30
TD	65536
SOLVENT	CDCl3
NS	16
DS	2
SWH	8223.685 Hz
FIDRES	0.125483 Hz
AQ	3.9846387 sec
RG	4
DW	60.800 usec
DE	6.50 usec
TE	294.9 K
D1	1.00000000 sec
TD0	1

======== CHANNEL f1 ========

NUC1	1H
P1	14.50 usec
PL1	0.50 dB
PL1W	12.76071072 W
SFO1	400.1324710 MHz
SI	32768
SF	400.1300101 MHz
WDW	EM
SSB	0
LB	0.30 Hz
GB	0
PC	1.00

CWVI-031

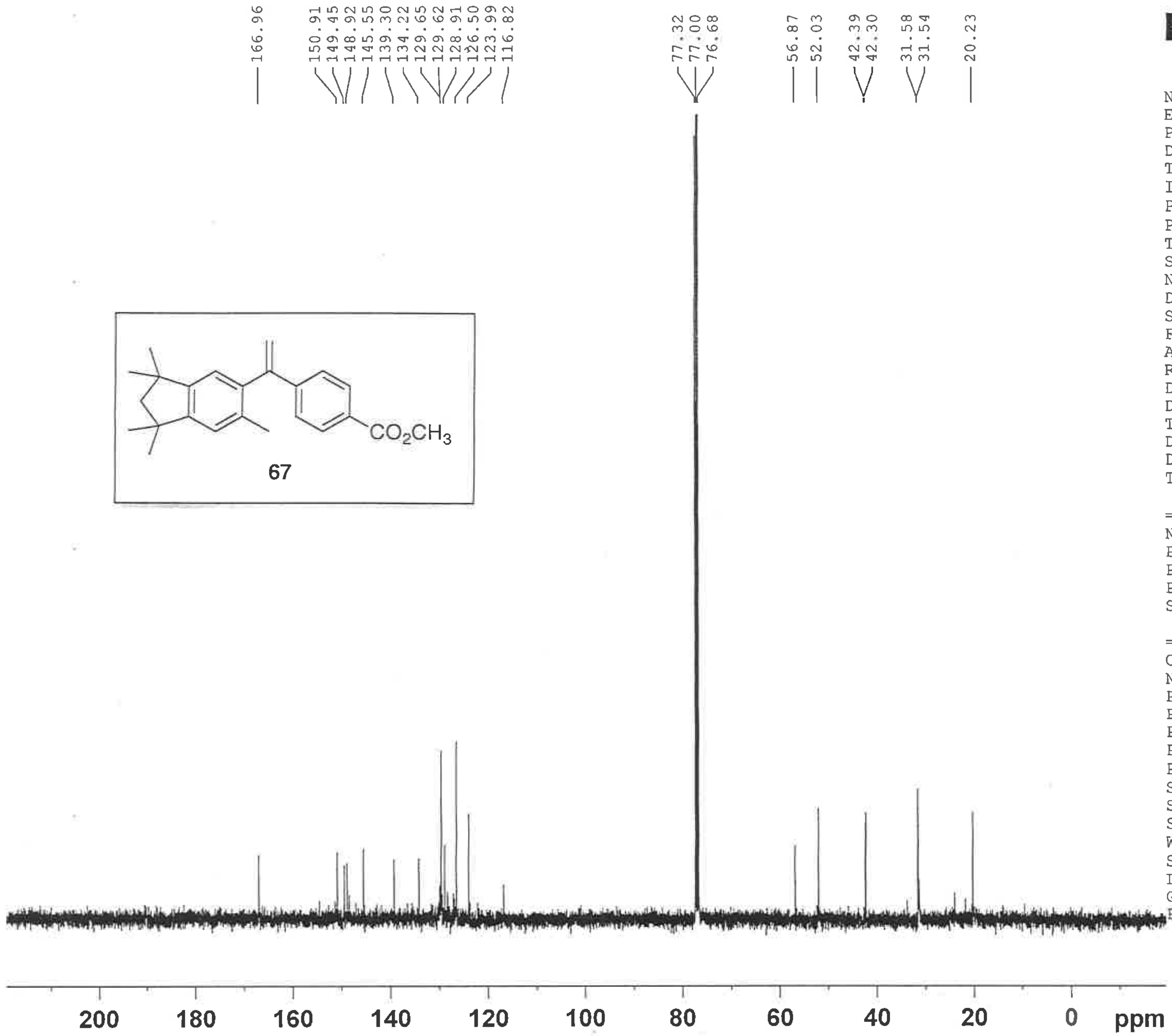

67

NAME	CWVI-031
EXPNO	2
PROCNO	1
Date_	20160718
Time	12.40
INSTRUM	spect
PROBHD	5 mm PABBO BB-
PULPROG	zgig30
TD	65536
SOLVENT	CDCl3
NS	209
DS	4
SWH	24038.461 Hz
FIDRES	0.366798 Hz
AQ	1.3631988 sec
RG	2050
DW	20.800 usec
DE	6.50 usec
TE	295.1 K
D1	2.00000000 sec
D11	0.03000000 sec
TD0	1
======== CHANNEL f1 ========	
NUC1	13C
P1	8.50 usec
PL1	-2.10 dB
PL1W	60.29227829 W
SFO1	100.6228298 MHz
======== CHANNEL f2 ========	
CPDPRG2	waltz16
NUC2	1H
PCPD2	90.00 usec
PL2	-1.80 dB
PL12	17.28 dB
PL2W	21.67079544 W
PL12W	0.26783961 W
SFO2	400.1316005 MHz
SI	32768
SF	100.6127736 MHz
WDW	EM
SSB	0
LB	1.00 Hz
GB	0
PC	1.40

CWVI-035

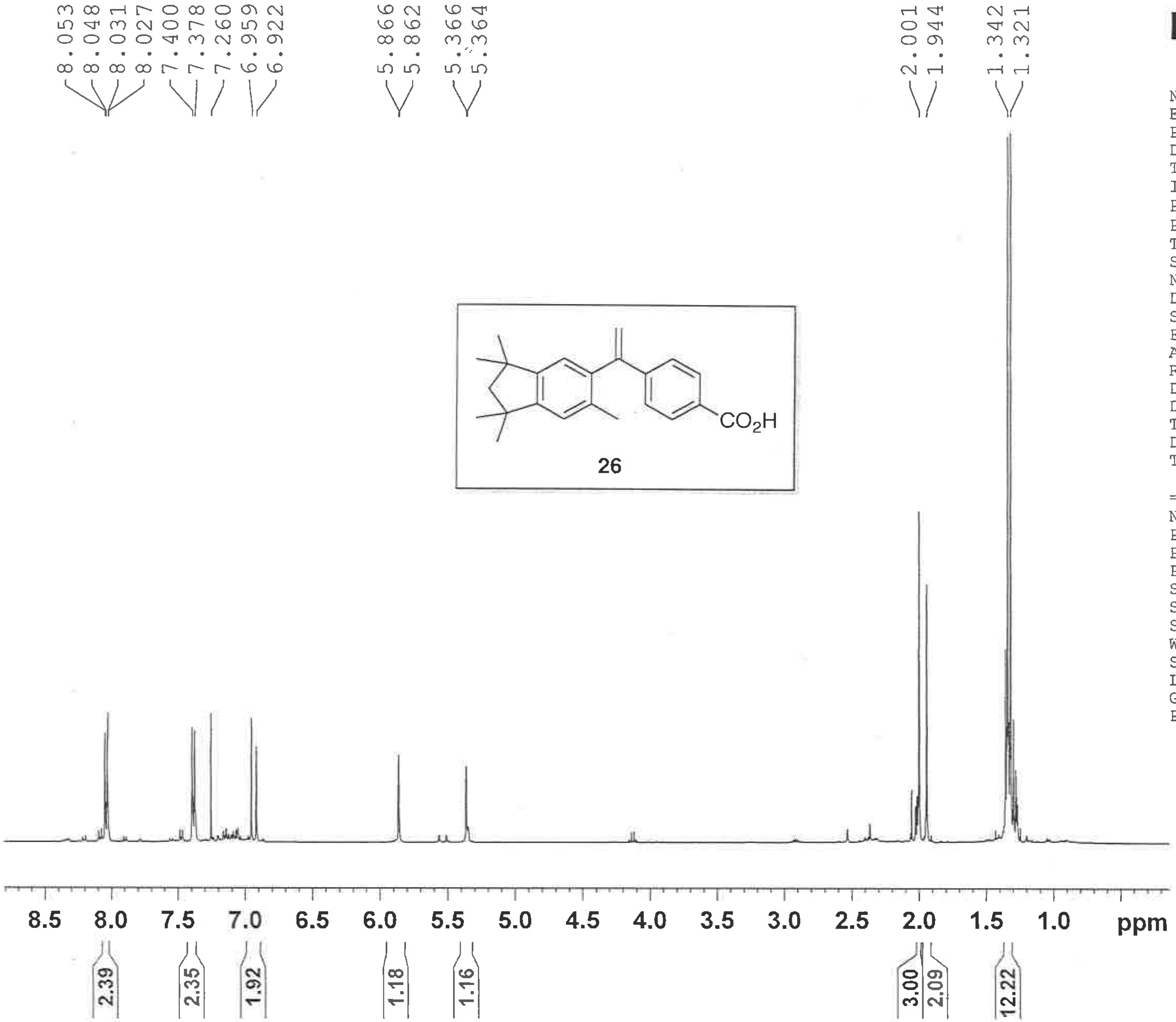

NAME	CWVI-035
EXPNO	1
PROCNO	1
Date_	20160729
Time	12.24
INSTRUM	spect
PROBHD	5 mm PABBO BB-
PULPROG	zg30
TD	65536
SOLVENT	CDCl3
NS	16
DS	2
SWH	8223.685 Hz
FIDRES	0.125483 Hz
AQ	3.9846387 sec
RG	4
DW	60.800 usec
DE	6.50 usec
TE	295.7 K
D1	1.00000000 sec
TD0	1

======== CHANNEL f1 ========

NUC1	1H
P1	14.50 usec
PL1	0.50 dB
PL1W	12.76071072 W
SFO1	400.1324710 MHz
SI	32768
SF	400.1300104 MHz
WDW	EM
SSB	0
LB	0.30 Hz
GB	0
PC	1.00

CWVI-035

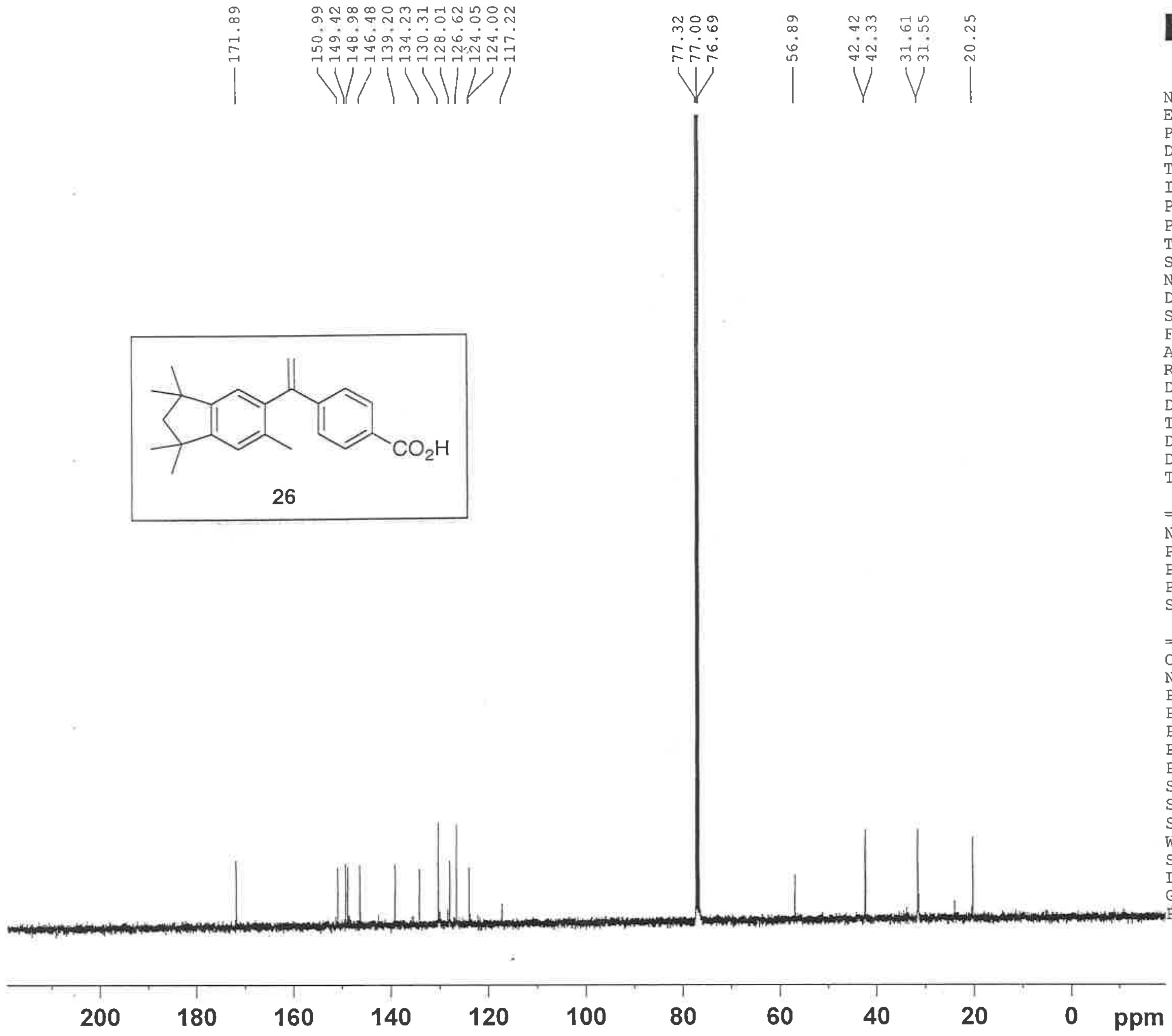

26

NAME	CWVI-035
EXPNO	2
PROCNO	1
Date_	20160729
Time	13.04
INSTRUM	spect
PROBHD	5 mm PABBO BB-
PULPROG	zgig30
TD	65536
SOLVENT	CDCl3
NS	731
DS	4
SWH	24038.461 Hz
FIDRES	0.366798 Hz
AQ	1.3631988 sec
RG	2050
DW	20.800 usec
DE	6.50 usec
TE	296.0 K
D1	2.00000000 sec
D11	0.03000000 sec
TD0	1
======== CHANNEL f1 ========	
NUC1	13C
P1	8.50 usec
PL1	-2.10 dB
PL1W	60.29227829 W
SFO1	100.6228298 MHz
======== CHANNEL f2 ========	
CPDPRG2	waltz16
NUC2	1H
PCPD2	90.00 usec
PL2	-1.80 dB
PL12	17.28 dB
PL2W	21.67079544 W
PL12W	0.26783961 W
SFO2	400.1316005 MHz
SI	32768
SF	100.6127714 MHz
WDW	EM
SSB	0
LB	1.00 Hz
GB	0
PC	1.40

CWV-251

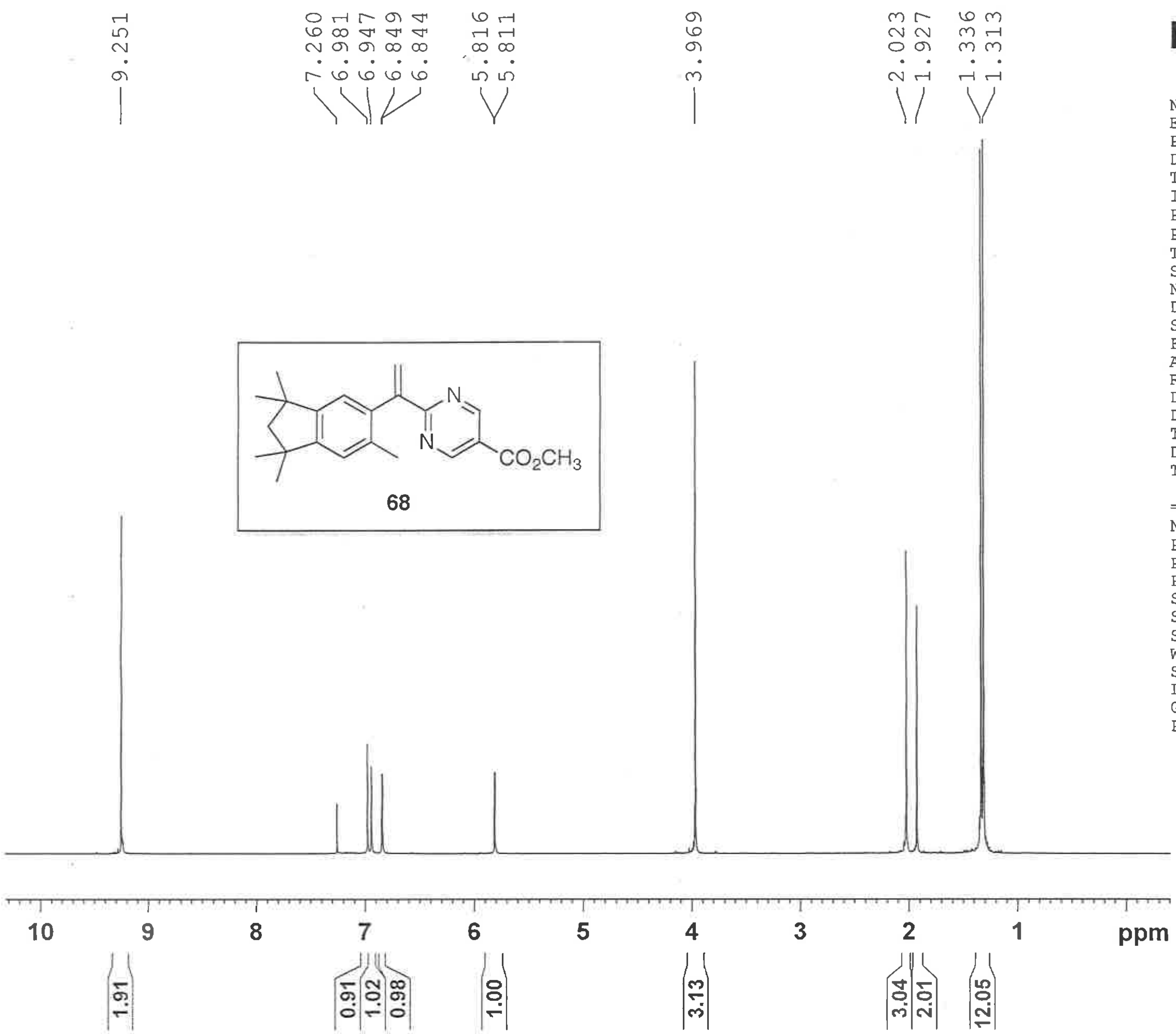

NAME	CWV-251
EXPNO	1
PROCNO	1
Date_	20150902
Time	11.41
INSTRUM	spect
PROBHD	5 mm PABBO BB-
PULPROG	zg30
TD	65536
SOLVENT	CDCl3
NS	16
DS	2
SWH	8223.685 Hz
FIDRES	0.125483 Hz
AQ	3.9846387 sec
RG	4
DW	60.800 usec
DE	6.50 usec
TE	299.3 K
D1	1.00000000 sec
TD0	1
======== CHANNEL f1 ========	
NUC1	1H
P1	14.50 usec
PL1	0.50 dB
PL1W	12.76071072 W
SFO1	400.1324710 MHz
SI	32768
SF	400.1300099 MHz
WDW	EM
SSB	0
LB	0.30 Hz
GB	0
PC	1.00

CWV-251

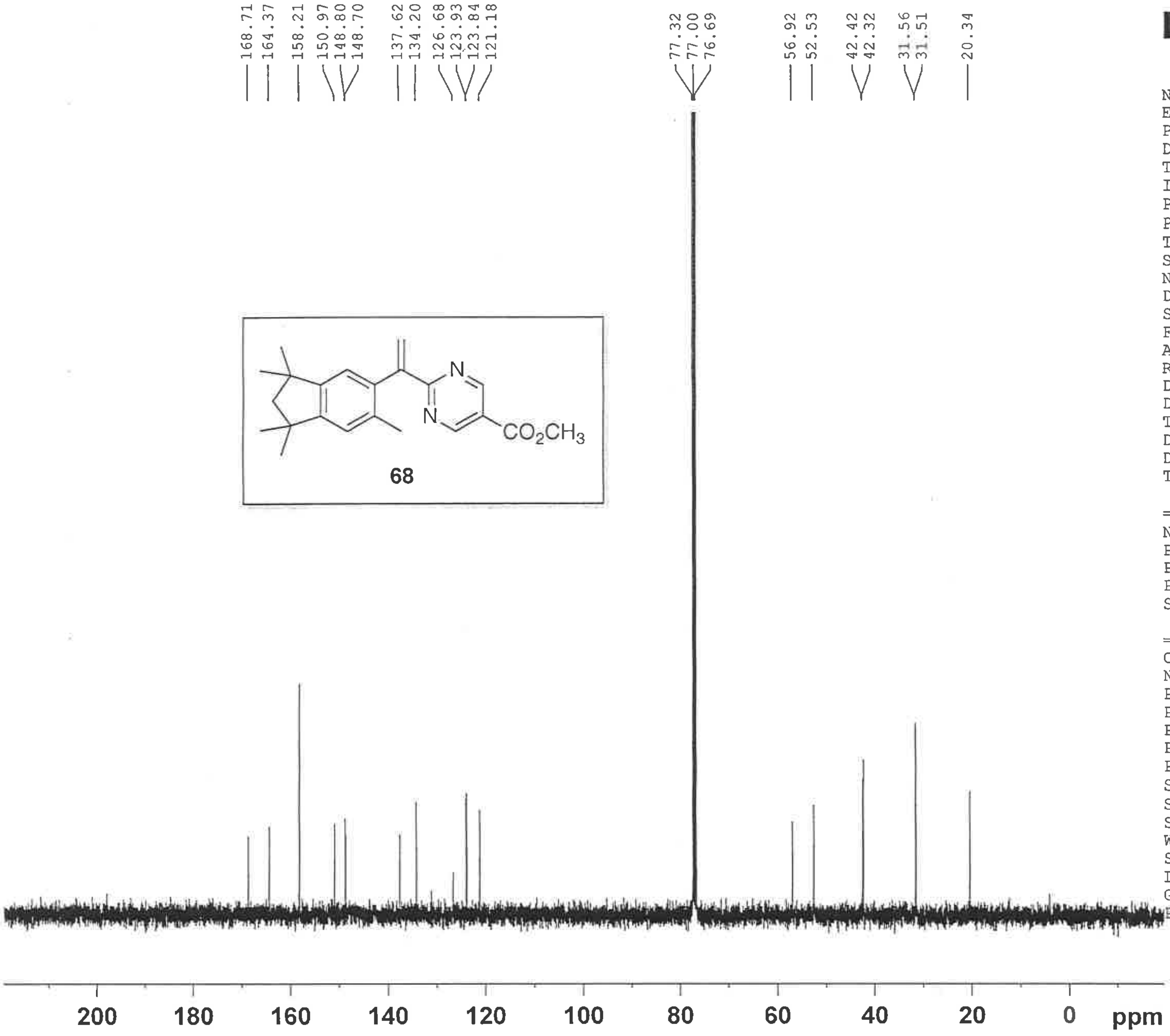

BRUKER

NAME	CWV-251
EXPNO	2
PROCNO	1
Date_	20150902
Time	11.50
INSTRUM	spect
PROBHD	5 mm PABBO BB-
PULPROG	zgig30
TD	65536
SOLVENT	CDCl3
NS	139
DS	4
SWH	24038.461 Hz
FIDRES	0.366798 Hz
AQ	1.3631988 sec
RG	2050
DW	20.800 usec
DE	6.50 usec
TE	300.0 K
D1	2.00000000 sec
D11	0.03000000 sec
TD0	1

======== CHANNEL f1 ========

NUC1	13C
P1	8.50 usec
PL1	-2.10 dB
PL1W	60.29227829 W
SFO1	100.6228298 MHz

======== CHANNEL f2 ========

CPDPRG2	waltz16
NUC2	1H
PCPD2	90.00 usec
PL2	-1.80 dB
PL12	17.28 dB
PL2W	21.67079544 W
PL12W	0.26783961 W
SFO2	400.1316005 MHz
SI	32768
SF	100.6127714 MHz
WDW	EM
SSB	0
LB	1.00 Hz
GB	0
PC	1.40

CWVI-007

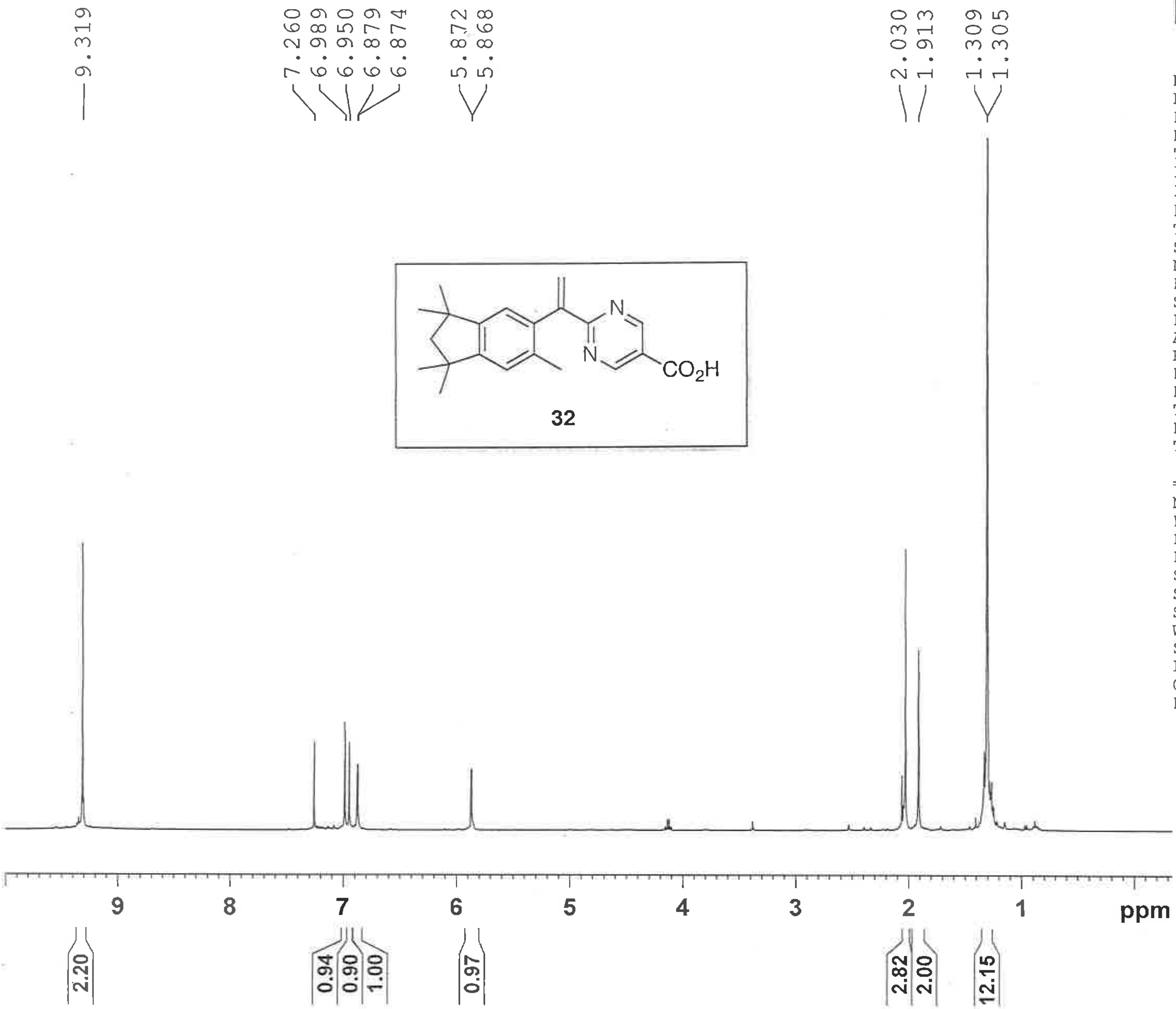

32

NAME	CWVI-007
EXPNO	1
PROCNO	1
Date_	20160328
Time	10.49
INSTRUM	spect
PROBHD	5 mm PABBO BB-
PULPROG	zg30
TD	65536
SOLVENT	CDCl3
NS	16
DS	2
SWH	8223.685 Hz
FIDRES	0.125483 Hz
AQ	3.9846387 sec
RG	4
DW	60.800 usec
DE	6.50 usec
TE	673.2 K
D1	1.00000000 sec
TD0	1

======== CHANNEL f1 ========

NUC1	1H
P1	14.50 usec
PL1	0.50 dB
PL1W	12.76071072 W
SFO1	400.1324710 MHz
SI	32768
SF	400.1300101 MHz
WDW	EM
SSB	0
LB	0.30 Hz
GB	0
PC	1.00

CWVI-007

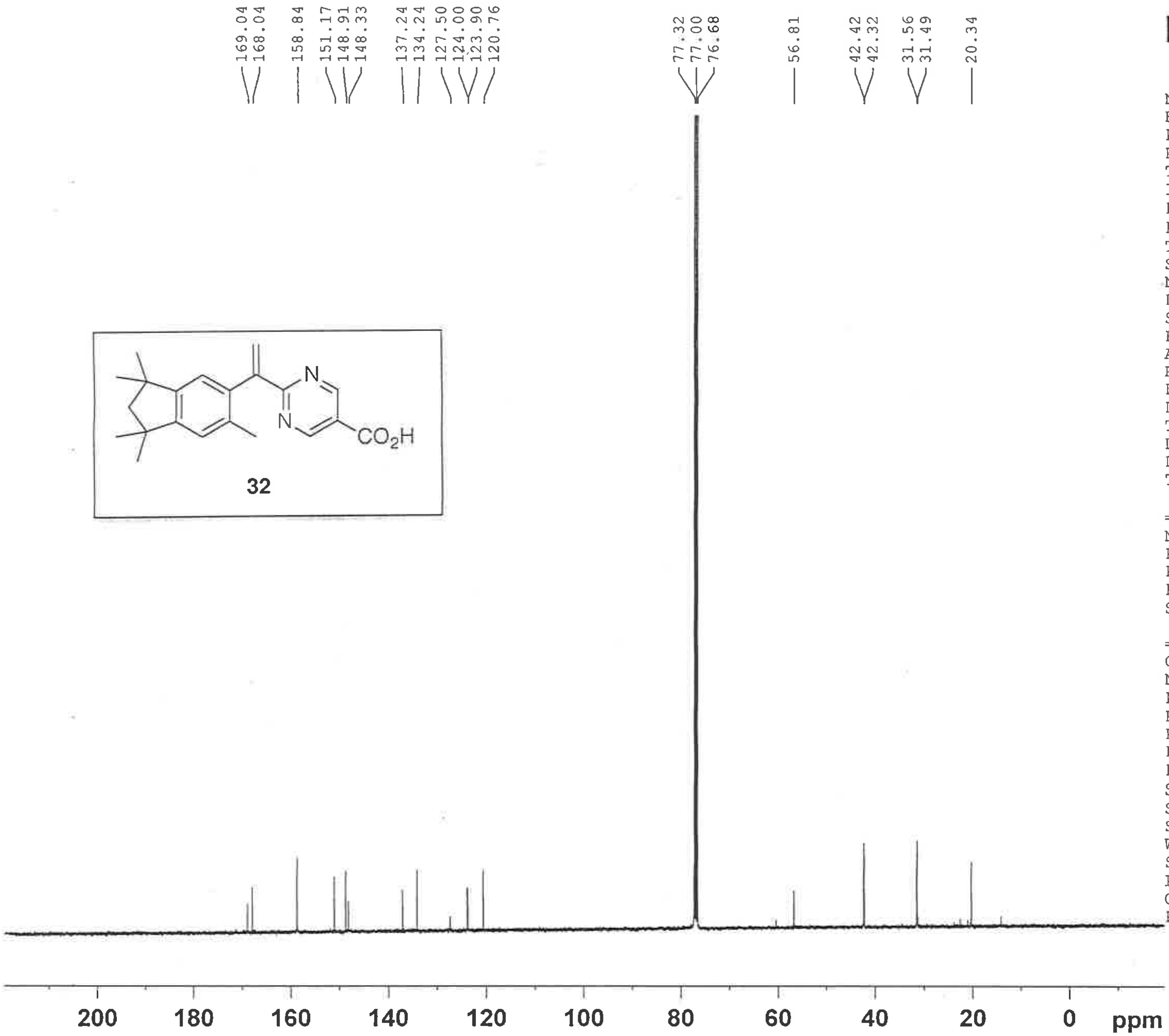

BRUKER

NAME	CWVI-007	
EXPNO	2	
PROCNO	1	
Date_	20160328	
Time	11.08	
INSTRUM	spect	
PROBHD	5 mm PABBO BB-	
PULPROG	zgig30	
TD	65536	
SOLVENT	CDCl3	
NS	3620	
DS	4	
SWH	24038.461	Hz
FIDRES	0.366798	Hz
AQ	1.3631988	sec
RG	2050	
DW	20.800	usec
DE	6.50	usec
TE	673.2	K
D1	2.00000000	sec
D11	0.03000000	sec
TD0	1	
======== CHANNEL f1 ========		
NUC1	13C	
P1	8.50	usec
PL1	-2.10	dB
PL1W	60.29227829	W
SFO1	100.6228298	MHz
======== CHANNEL f2 ========		
CPDPRG2	waltz16	
NUC2	1H	
PCPD2	90.00	usec
PL2	-1.80	dB
PL12	17.28	dB
PL2W	21.67079544	W
PL12W	0.26783961	W
SFO2	400.1316005	MHz
SI	32768	
SF	100.6127729	MHz
WDW	EM	
SSB	0	
LB	1.00	Hz
GB	0	
PC	1.40	

CWV-271

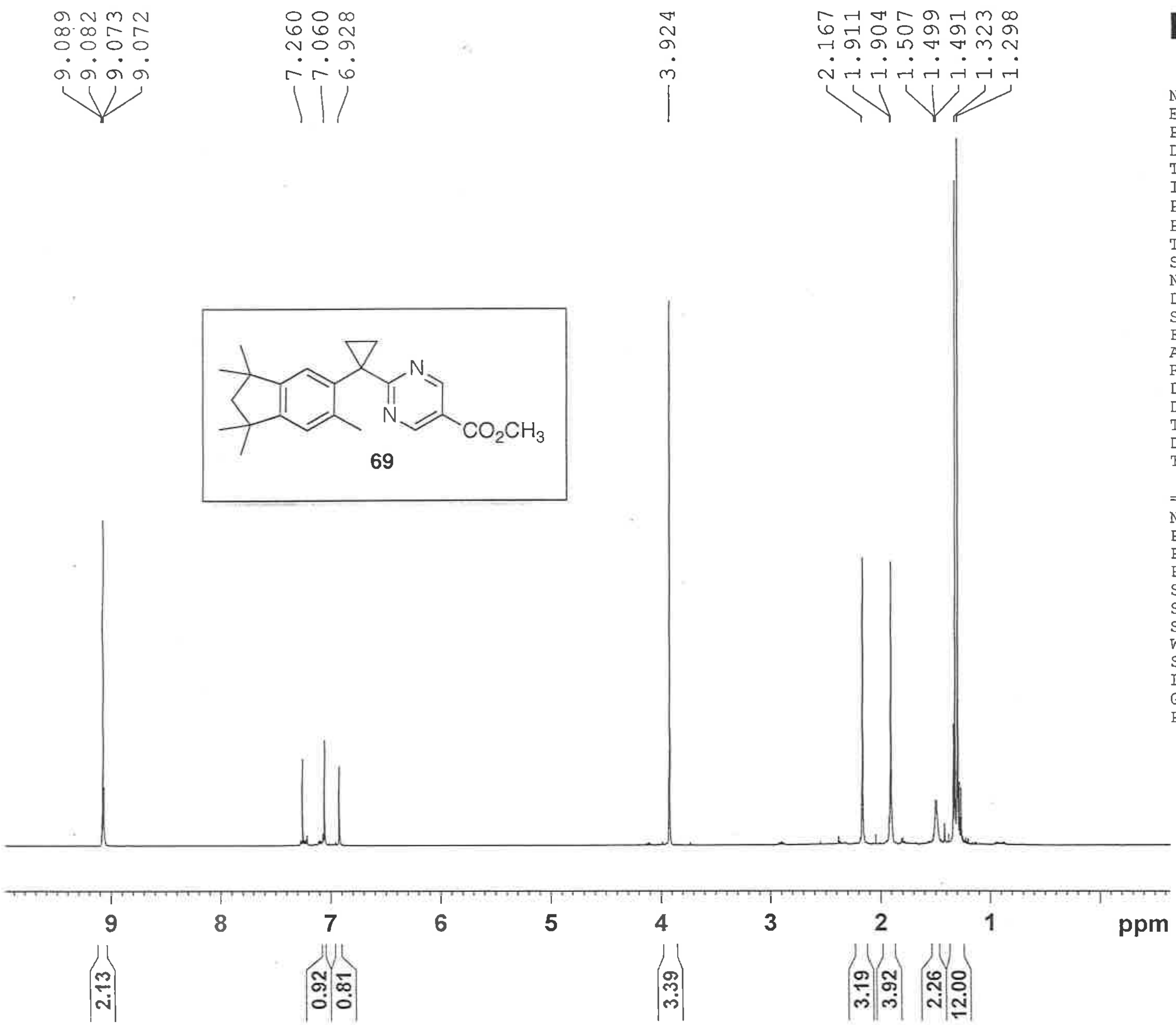

BRUKER

```
NAME          CWV-271
EXPNO               1
PROCNO              1
Date_        20151009
Time            10.50
INSTRUM         spect
PROBHD   5 mm PABBO BB-
PULPROG          zg30
TD              65536
SOLVENT         CDCl3
NS                 16
DS                  2
SWH          8223.685 Hz
FIDRES       0.125483 Hz
AQ          3.9846387 sec
RG                  4
DW             60.800 usec
DE               6.50 usec
TE              298.1 K
D1         1.00000000 sec
TD0                 1

======== CHANNEL f1 ========
NUC1               1H
P1              14.50 usec
PL1              0.50 dB
PL1W      12.76071072 W
SFO1     400.1324710 MHz
SI              32768
SF       400.1300099 MHz
WDW                EM
SSB                 0
LB               0.30 Hz
GB                  0
PC               1.00
```

CWV-271

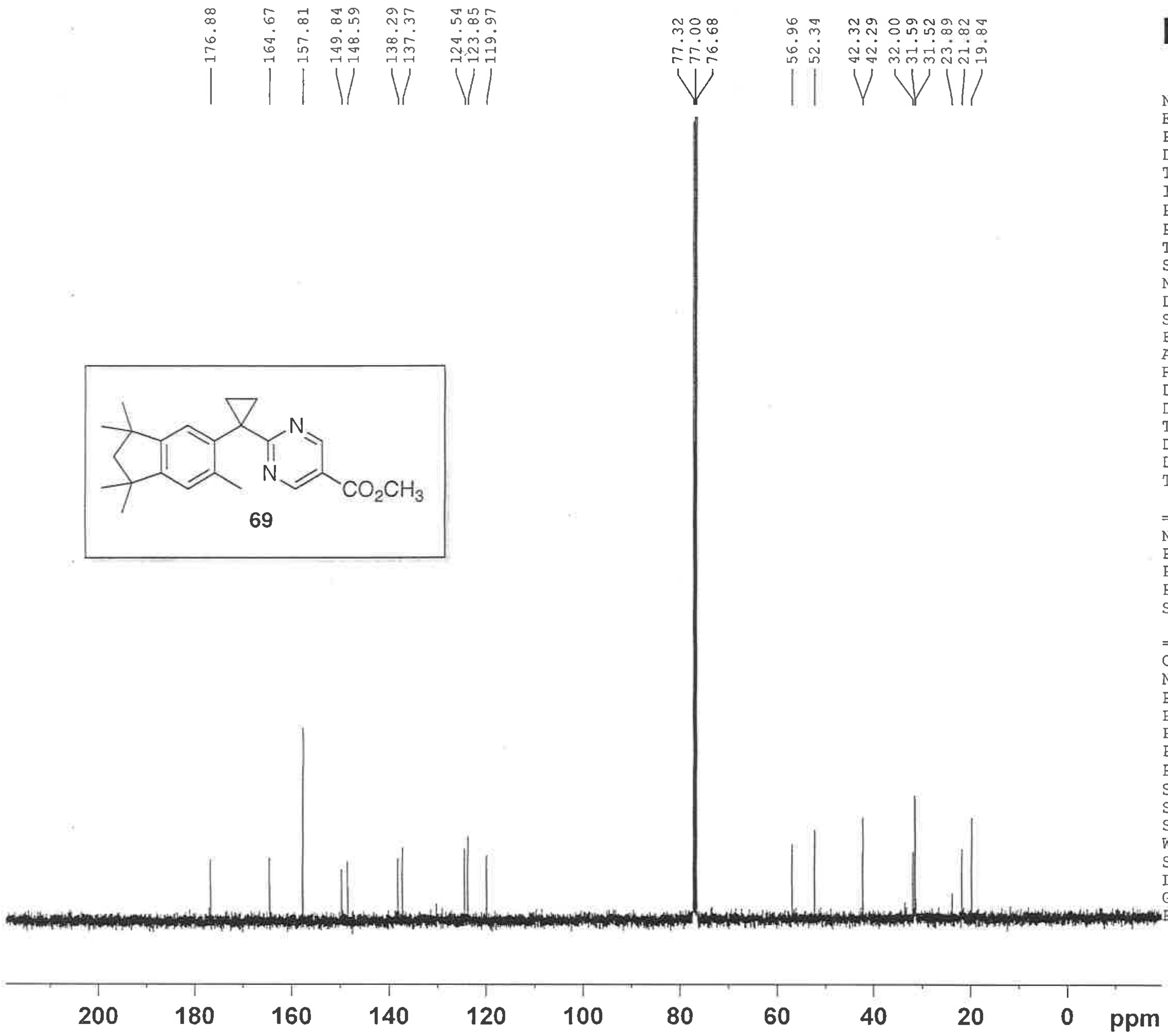

69

BRUKER

NAME	CWV-271	
EXPNO	2	
PROCNO	1	
Date_	20151009	
Time	11.00	
INSTRUM	spect	
PROBHD	5 mm PABBO BB-	
PULPROG	zgig30	
TD	65536	
SOLVENT	CDCl3	
NS	282	
DS	4	
SWH	24038.461	Hz
FIDRES	0.366798	Hz
AQ	1.3631988	sec
RG	2050	
DW	20.800	usec
DE	6.50	usec
TE	298.4	K
D1	2.00000000	sec
D11	0.03000000	sec
TD0	1	
======== CHANNEL f1 ========		
NUC1	13C	
P1	8.50	usec
PL1	-2.10	dB
PL1W	60.29227829	W
SFO1	100.6228298	MHz
======== CHANNEL f2 ========		
CPDPRG2	waltz16	
NUC2	1H	
PCPD2	90.00	usec
PL2	-1.80	dB
PL12	17.28	dB
PL2W	21.67079544	W
PL12W	0.26783961	W
SFO2	400.1316005	MHz
SI	32768	
SF	100.6127714	MHz
WDW	EM	
SSB	0	
LB	1.00	Hz
GB	0	
PC	1.40	

CWVI-003

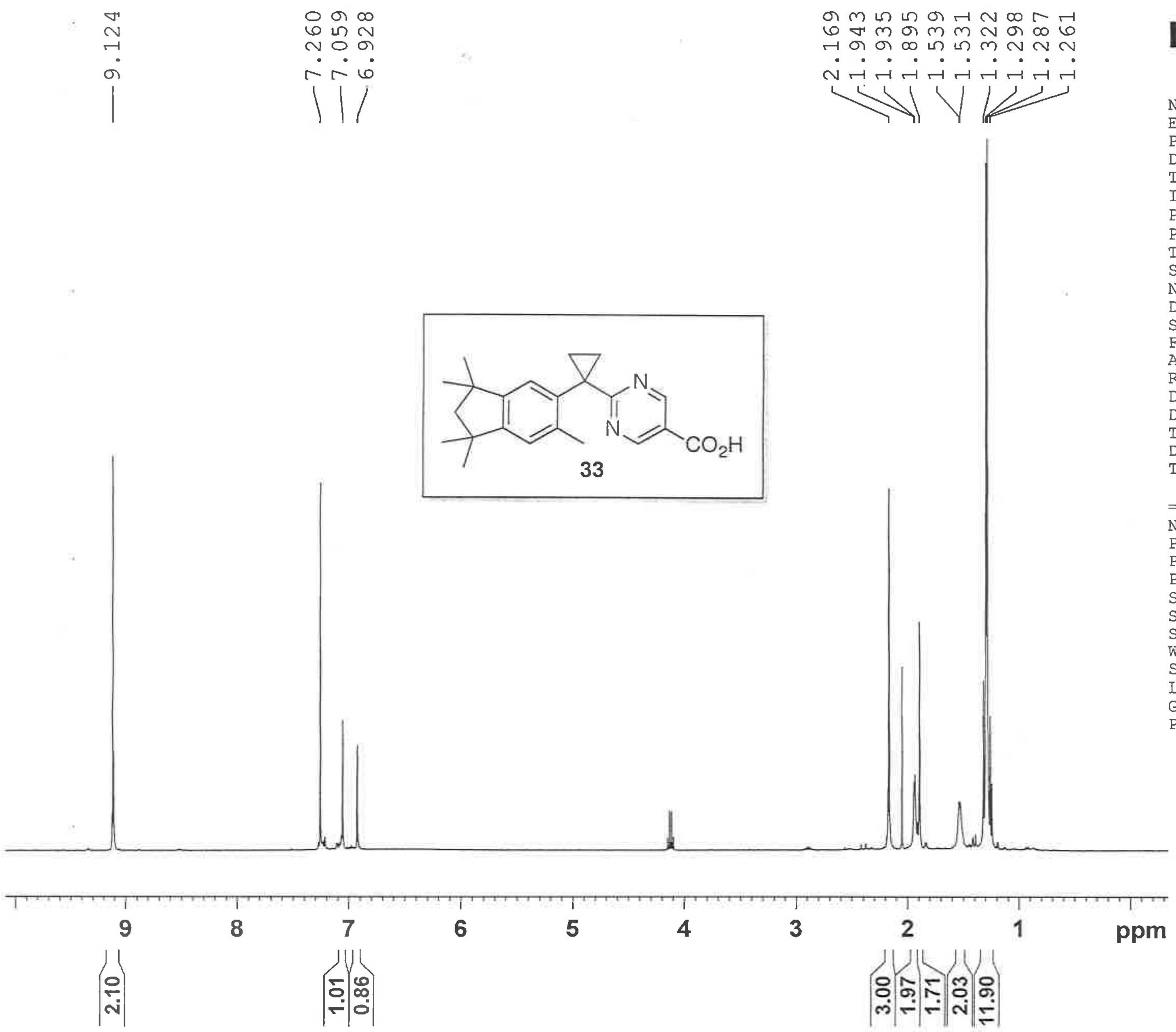

33

NAME	CWVI-003
EXPNO	1
PROCNO	1
Date_	20160328
Time_	10.43
INSTRUM	spect
PROBHD	5 mm PABBO BB-
PULPROG	zg30
TD	65536
SOLVENT	CDCl3
NS	16
DS	2
SWH	8223.685 Hz
FIDRES	0.125483 Hz
AQ	3.9846387 sec
RG	4
DW	60.800 usec
DE	6.50 usec
TE	673.2 K
D1	1.00000000 sec
TD0	1
======== CHANNEL f1 ========	
NUC1	1H
P1	14.50 usec
PL1	0.50 dB
PL1W	12.76071072 W
SFO1	400.1324710 MHz
SI	32768
SF	400.1300101 MHz
WDW	EM
SSB	0
LB	0.30 Hz
GB	0
PC	1.00

CWVI-003

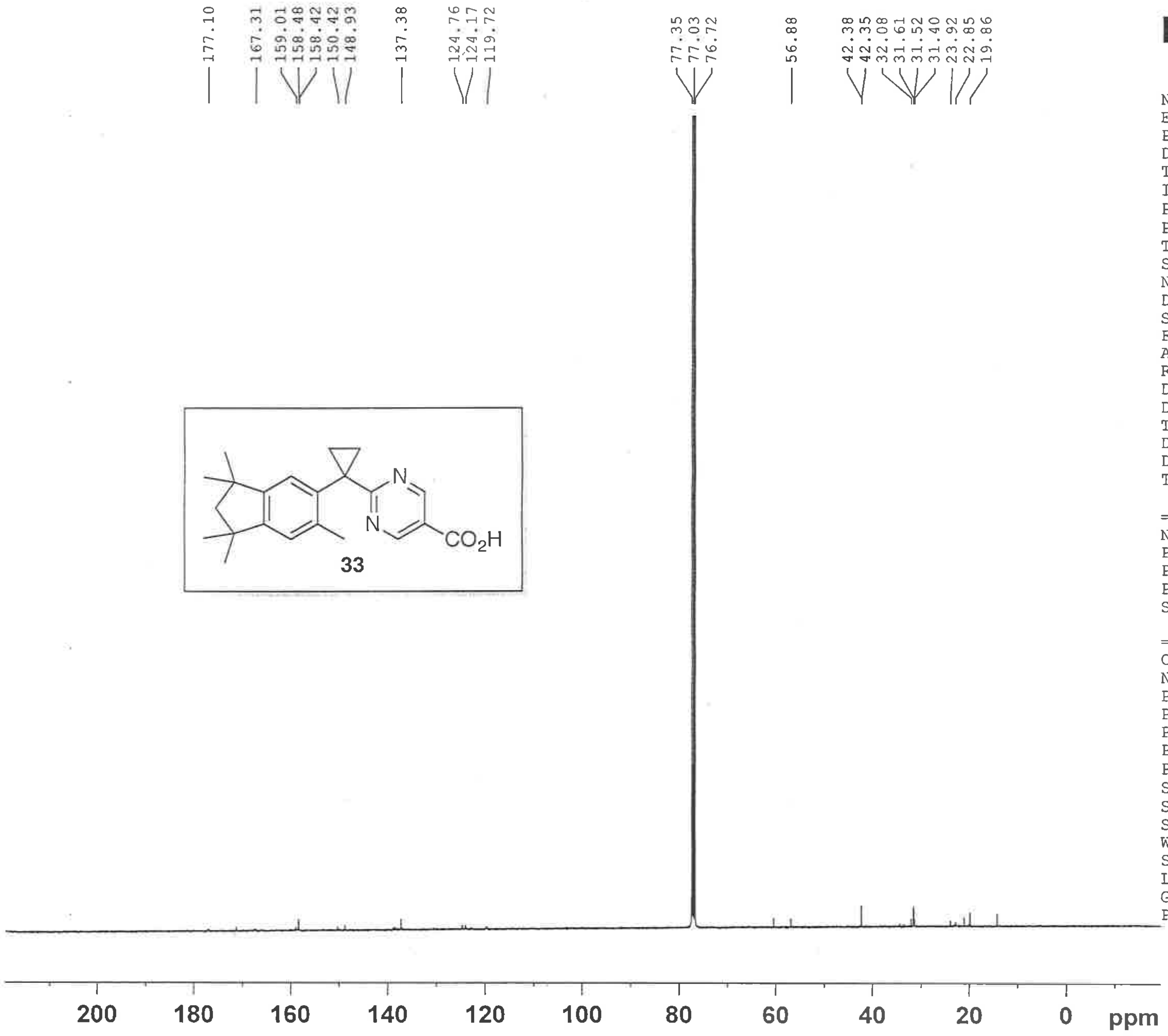

BRUKER

NAME	CWVI-003
EXPNO	2
PROCNO	1
Date_	20160330
Time	14.39
INSTRUM	spect
PROBHD	5 mm PABBO BB-
PULPROG	zgig30
TD	65536
SOLVENT	CDCl3
NS	19951
DS	4
SWH	24038.461 Hz
FIDRES	0.366798 Hz
AQ	1.3631988 sec
RG	2050
DW	20.800 usec
DE	6.50 usec
TE	673.2 K
D1	2.00000000 sec
D11	0.03000000 sec
TD0	1
======== CHANNEL f1 ========	
NUC1	13C
P1	8.50 usec
PL1	-2.10 dB
PL1W	60.29227829 W
SFO1	100.6228298 MHz
======== CHANNEL f2 ========	
CPDPRG2	waltz16
NUC2	1H
PCPD2	90.00 usec
PL2	-1.80 dB
PL12	17.28 dB
PL2W	21.67079544 W
PL12W	0.26783961 W
SFO2	400.1316005 MHz
SI	32768
SF	100.6127690 MHz
WDW	EM
SSB	0
LB	1.00 Hz
GB	0
PC	1.40

CWIII-121

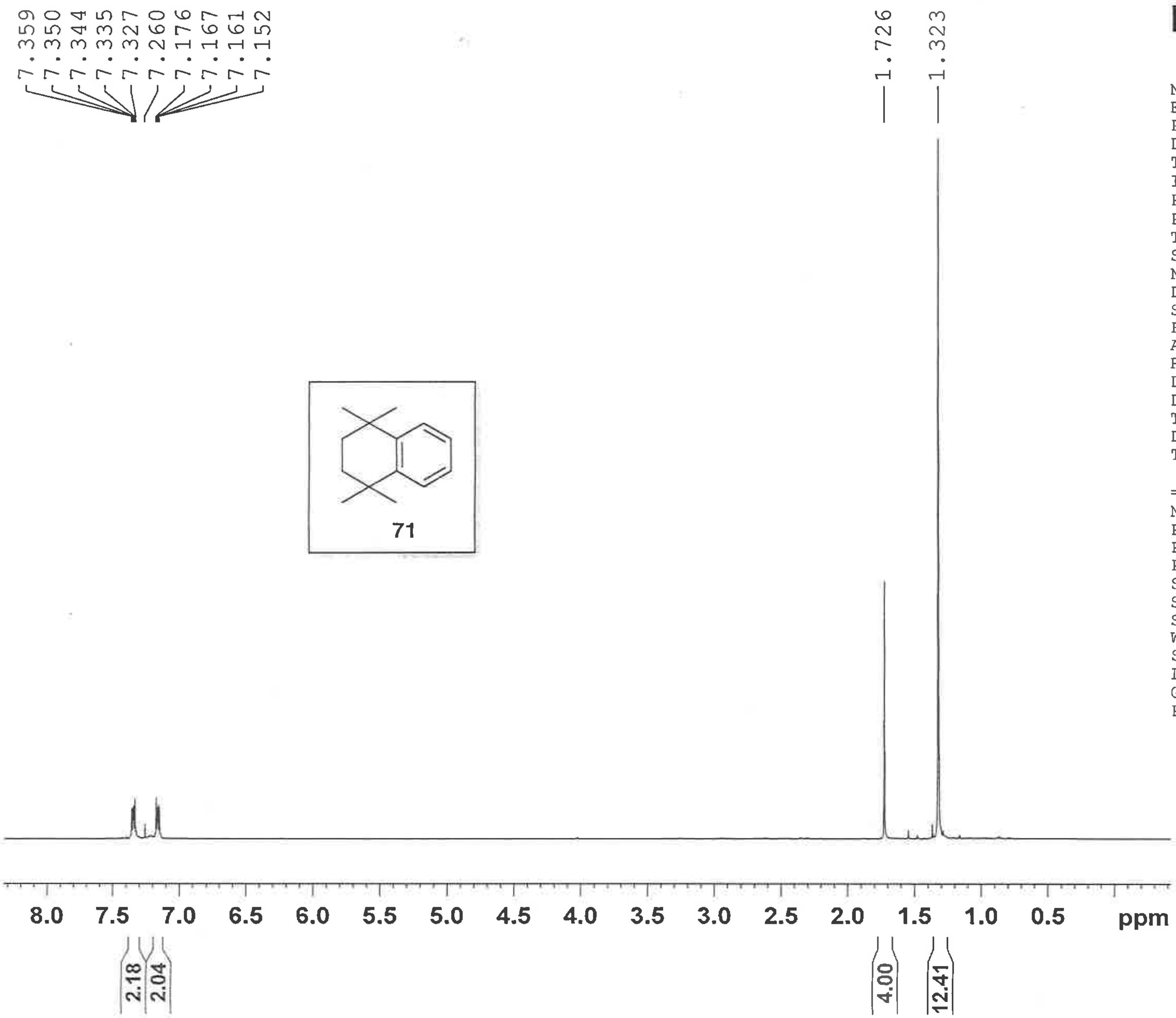

NAME	CWIII-121
EXPNO	1
PROCNO	1
Date_	20100609
Time	11.35
INSTRUM	spect
PROBHD	5 mm PABBO BB-
PULPROG	zg30
TD	65536
SOLVENT	CDCl3
NS	16
DS	2
SWH	8223.685 Hz
FIDRES	0.125483 Hz
AQ	3.9846387 sec
RG	4
DW	60.800 usec
DE	6.50 usec
TE	291.2 K
D1	1.00000000 sec
TD0	1

======== CHANNEL f1 ========

NUC1	1H
P1	10.00 usec
PL1	-1.80 dB
PL1W	21.67079544 W
SFO1	400.1324710 MHz
SI	32768
SF	400.1300104 MHz
WDW	EM
SSB	0
LB	0.30 Hz
GB	0
PC	1.00

CWIII-121

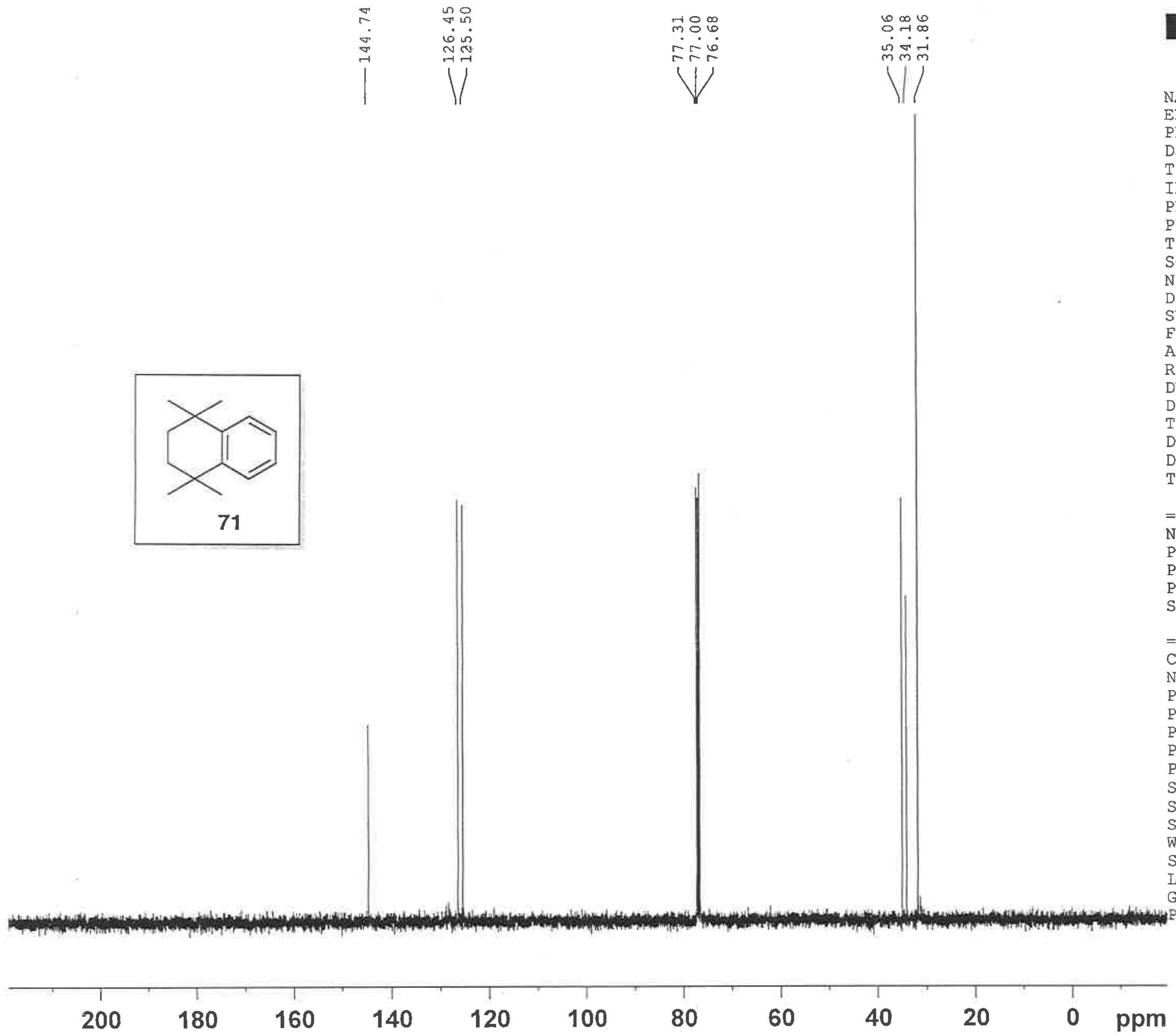

71

BRUKER

NAME	CWIII-121
EXPNO	2
PROCNO	1
Date_	20100609
Time	11.43
INSTRUM	spect
PROBHD	5 mm PABBO BB-
PULPROG	zgig30
TD	65536
SOLVENT	CDCl3
NS	41
DS	4
SWH	24038.461 Hz
FIDRES	0.366798 Hz
AQ	1.3631988 sec
RG	2050
DW	20.800 usec
DE	6.50 usec
TE	291.4 K
D1	2.00000000 sec
D11	0.03000000 sec
TD0	1
======== CHANNEL f1 ========	
NUC1	13C
P1	8.50 usec
PL1	-1.50 dB
PL1W	52.51238251 W
SFO1	100.6228298 MHz
======== CHANNEL f2 ========	
CPDPRG2	waltz16
NUC2	1H
PCPD2	90.00 usec
PL2	-1.80 dB
PL12	17.28 dB
PL2W	21.67079544 W
PL12W	0.26783961 W
SFO2	400.1316005 MHz
SI	32768
SF	100.6127766 MHz
WDW	EM
SSB	0
LB	1.00 Hz
GB	0
PC	1.40

CWV-297

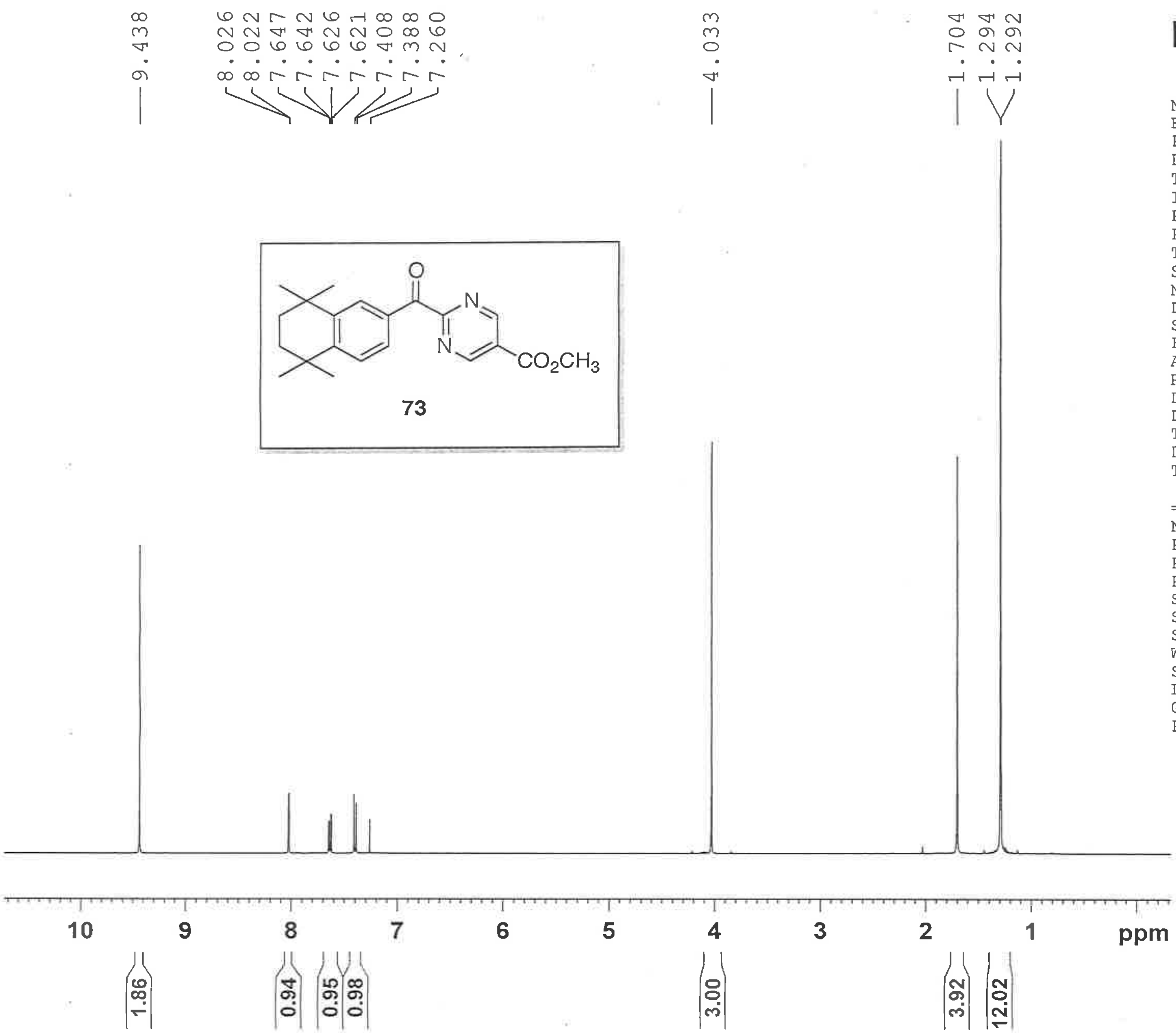


```
NAME              CWV-297
EXPNO                   1
PROCNO                  1
Date_            20151224
Time_               15.10
INSTRUM             spect
PROBHD   5 mm PABBO BB-
PULPROG              zg30
TD                  65536
SOLVENT             CDCl3
NS                     16
DS                      2
SWH              8223.685 Hz
FIDRES           0.125483 Hz
AQ              3.9846387 sec
RG                      4
DW                 60.800 usec
DE                   6.50 usec
TE                  298.2 K
D1             1.00000000 sec
TD0                     1

======== CHANNEL f1 ========
NUC1                   1H
P1                  14.50 usec
PL1                  0.50 dB
PL1W          12.76071072 W
SFO1          400.1324710 MHz
SI                  32768
SF            400.1300096 MHz
WDW                    EM
SSB                     0
LB                   0.30 Hz
GB                      0
PC                   1.00
```

CWV-297

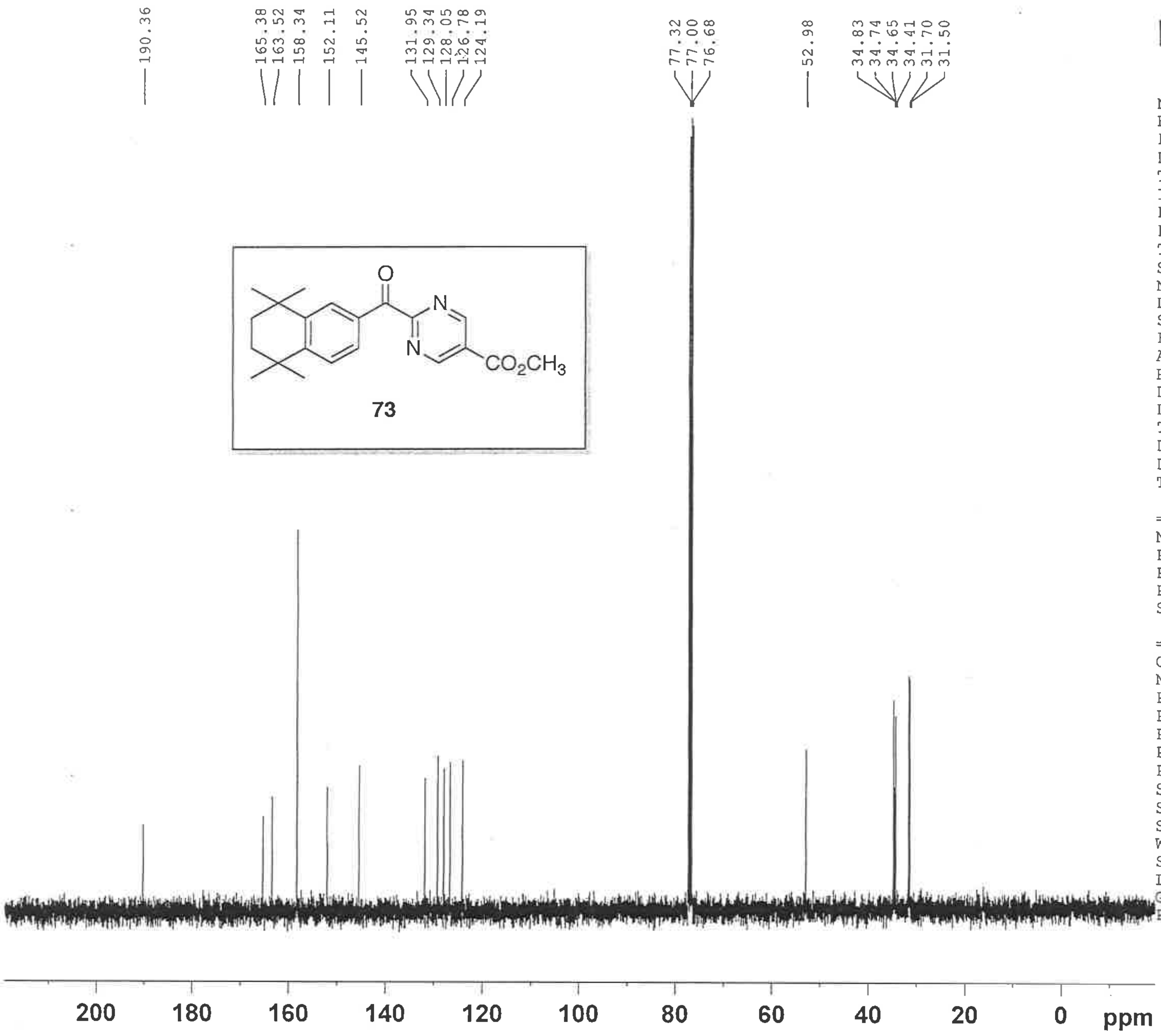

73

BRUKER

NAME	CWV-297
EXPNO	2
PROCNO	1
Date_	20151224
Time	15.15
INSTRUM	spect
PROBHD	5 mm PABBO BB-
PULPROG	zgig30
TD	65536
SOLVENT	CDCl3
NS	64
DS	4
SWH	24038.461 Hz
FIDRES	0.366798 Hz
AQ	1.3631988 sec
RG	2050
DW	20.800 usec
DE	6.50 usec
TE	298.2 K
D1	2.00000000 sec
D11	0.03000000 sec
TD0	1
======== CHANNEL f1 ========	
NUC1	13C
P1	8.50 usec
PL1	-2.10 dB
PL1W	60.29227829 W
SFO1	100.6228298 MHz
======== CHANNEL f2 ========	
CPDPRG2	waltz16
NUC2	1H
PCPD2	90.00 usec
PL2	-1.80 dB
PL12	17.28 dB
PL2W	21.67079544 W
PL12W	0.26783961 W
SFO2	400.1316005 MHz
SI	32768
SF	100.6127736 MHz
WDW	EM
SSB	0
LB	1.00 Hz
GB	0
PC	1.40

CWVI-017

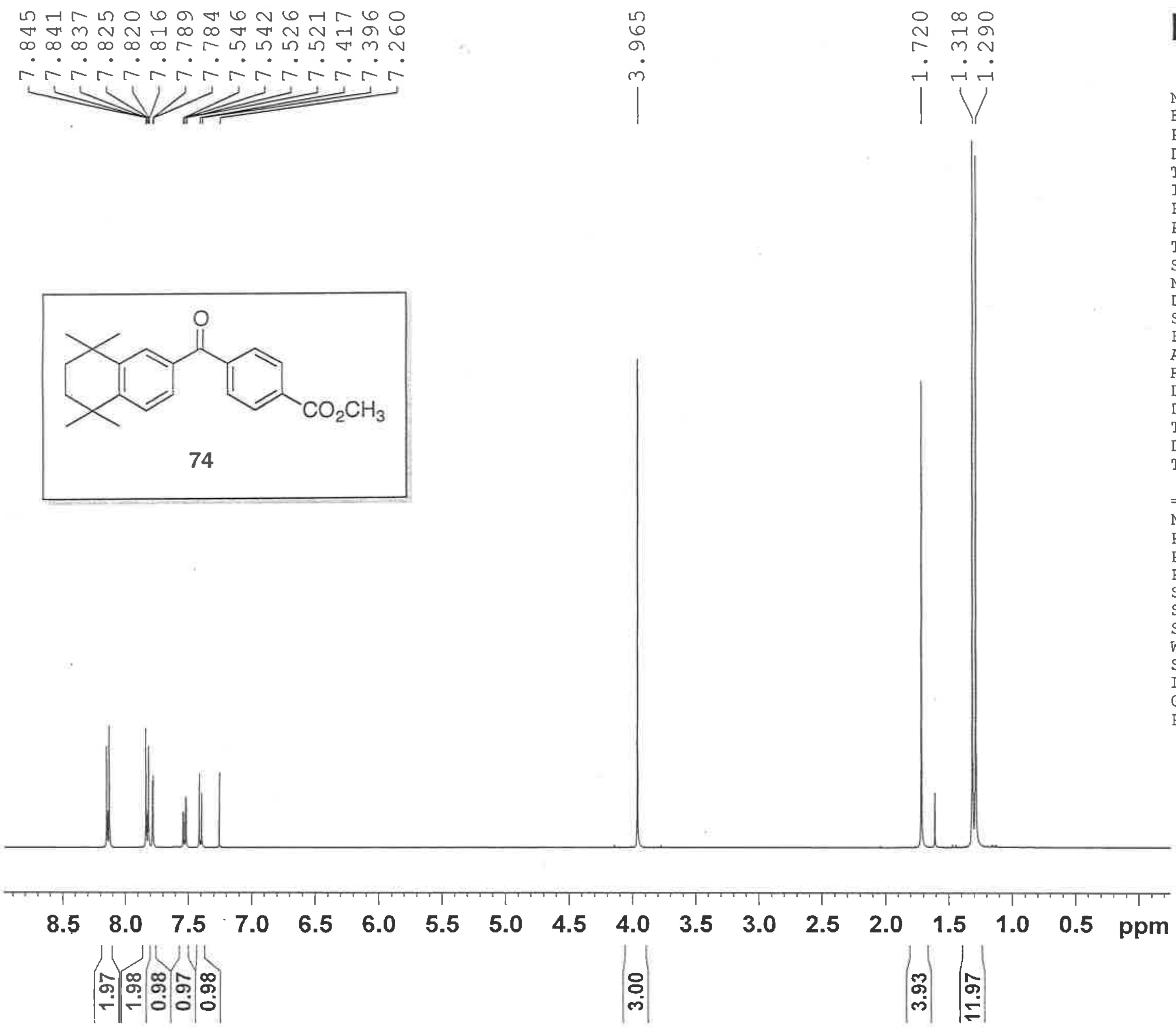

74

BRUKER

NAME	CWVI-017
EXPNO	1
PROCNO	1
Date_	20160728
Time	14.54
INSTRUM	spect
PROBHD	5 mm PABBO BB-
PULPROG	zg30
TD	65536
SOLVENT	CDCl3
NS	16
DS	2
SWH	8223.685 Hz
FIDRES	0.125483 Hz
AQ	3.9846387 sec
RG	4
DW	60.800 usec
DE	6.50 usec
TE	673.2 K
D1	1.00000000 sec
TD0	1

======== CHANNEL f1 ========

NUC1	1H
P1	14.50 usec
PL1	0.50 dB
PL1W	12.76071072 W
SFO1	400.1324710 MHz
SI	32768
SF	400.1300104 MHz
WDW	EM
SSB	0
LB	0.30 Hz
GB	0
PC	1.00

CWVI-017

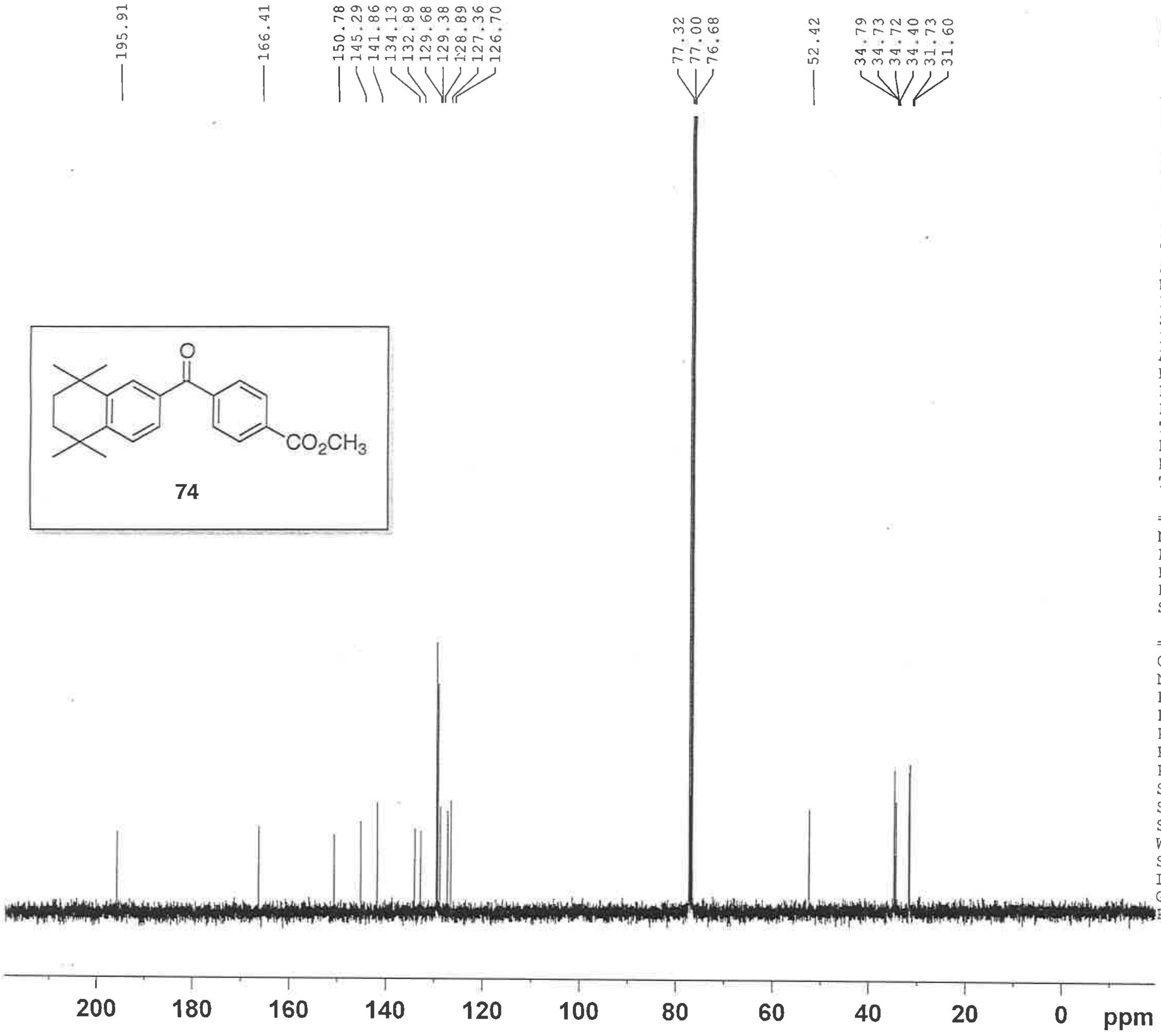

74

NAME	CWVI-017
EXPNO	2
PROCNO	1
Date_	20160728
Time	15.00
INSTRUM	spect
PROBHD	5 mm PABBO BB-
PULPROG	zgig30
TD	65536
SOLVENT	CDCl3
NS	157
DS	4
SWH	24038.461 Hz
FIDRES	0.366798 Hz
AQ	1.3631988 sec
RG	2050
DW	20.800 usec
DE	6.50 usec
TE	673.2 K
D1	2.00000000 sec
D11	0.03000000 sec
TD0	1

======== CHANNEL f1 ========

NUC1	13C
P1	8.50 usec
PL1	-2.10 dB
PL1W	60.29227829 W
SFO1	100.6228298 MHz

======== CHANNEL f2 ========

CPDPRG2	waltz16
NUC2	1H
PCPD2	90.00 usec
PL2	-1.80 dB
PL12	17.28 dB
PL2W	21.67079544 W
PL12W	0.26783961 W
SFO2	400.1316005 MHz
SI	32768
SF	100.6127722 MHz
WDW	EM
SSB	0
LB	1.00 Hz
GB	0
PC	1.40

CWVI-015

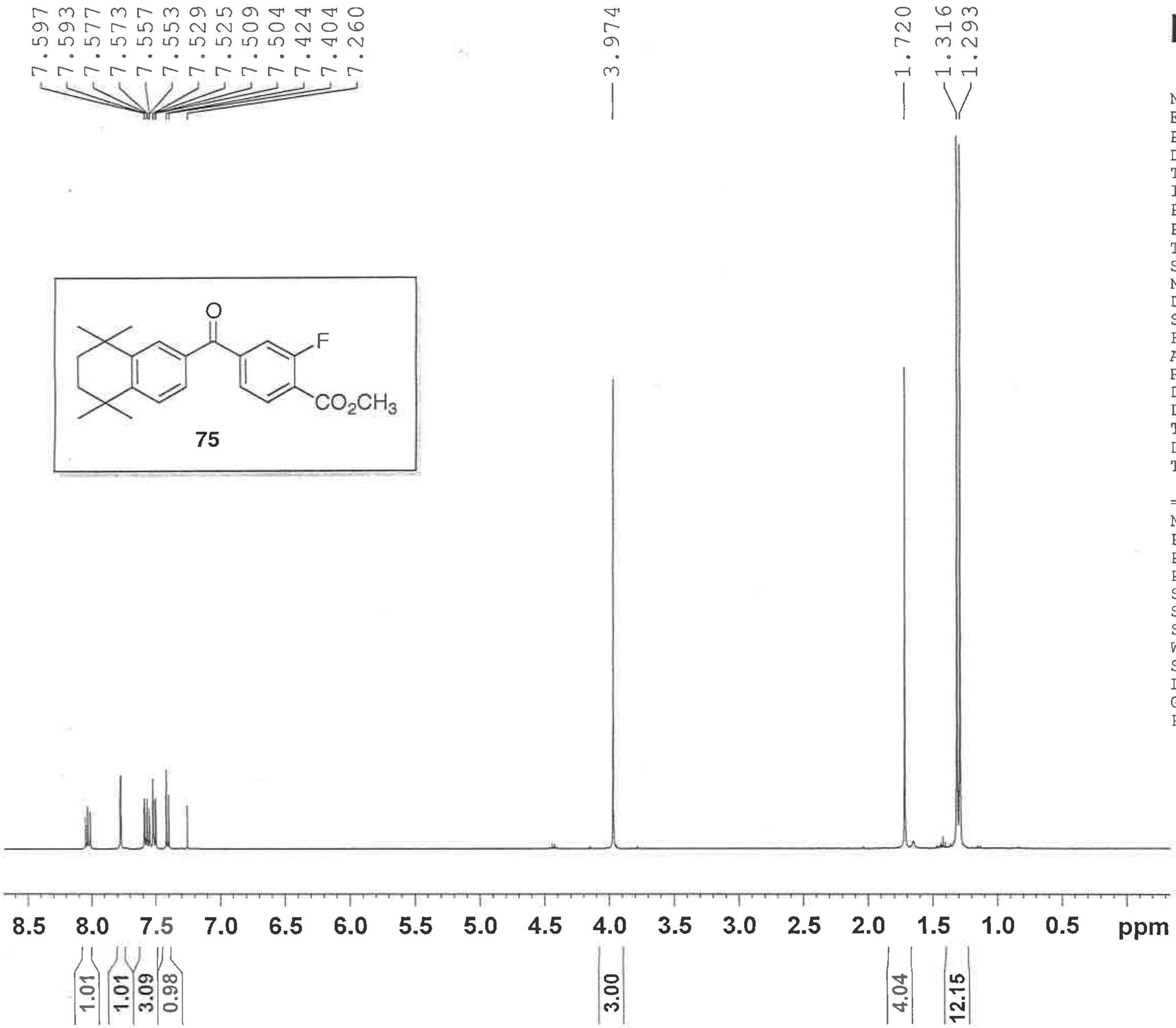

75

NAME	CWVI-015
EXPNO	1
PROCNO	1
Date_	20160728
Time	14.27
INSTRUM	spect
PROBHD	5 mm PABBO BB-
PULPROG	zg30
TD	65536
SOLVENT	CDCl3
NS	16
DS	2
SWH	8223.685 Hz
FIDRES	0.125483 Hz
AQ	3.9846387 sec
RG	4
DW	60.800 usec
DE	6.50 usec
TE	673.2 K
D1	1.00000000 sec
TD0	1

======== CHANNEL f1 ========

NUC1	1H
P1	14.50 usec
PL1	0.50 dB
PL1W	12.76071072 W
SFO1	400.1324710 MHz
SI	32768
SF	400.1300104 MHz
WDW	EM
SSB	0
LB	0.30 Hz
GB	0
PC	1.00

CWVI-015

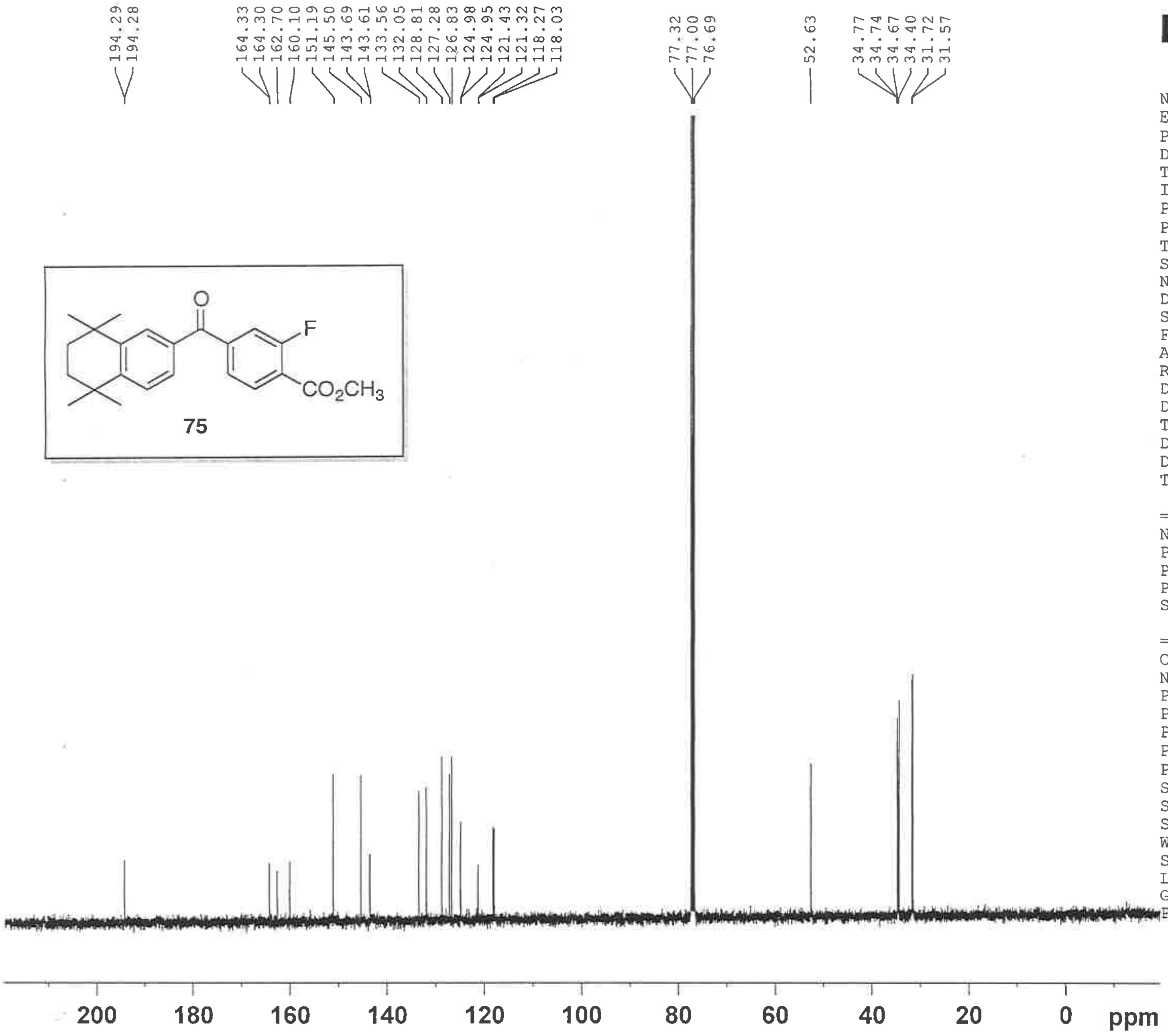

75

BRUKER

NAME	CWVI-015	
EXPNO	2	
PROCNO	1	
Date_	20160728	
Time	14.46	
INSTRUM	spect	
PROBHD	5 mm PABBO BB-	
PULPROG	zgig30	
TD	65536	
SOLVENT	CDCl3	
NS	360	
DS	4	
SWH	24038.461	Hz
FIDRES	0.366798	Hz
AQ	1.3631988	sec
RG	2050	
DW	20.800	usec
DE	6.50	usec
TE	673.2	K
D1	2.00000000	sec
D11	0.03000000	sec
TD0	1	

======== CHANNEL f1 ========

NUC1	13C	
P1	8.50	usec
PL1	-2.10	dB
PL1W	60.29227829	W
SFO1	100.6228298	MHz

======== CHANNEL f2 ========

CPDPRG2	waltz16	
NUC2	1H	
PCPD2	90.00	usec
PL2	-1.80	dB
PL12	17.28	dB
PL2W	21.67079544	W
PL12W	0.26783961	W
SFO2	400.1316005	MHz
SI	32768	
SF	100.6127729	MHz
WDW	EM	
SSB	0	
LB	1.00	Hz
GB	0	
PC	1.40	

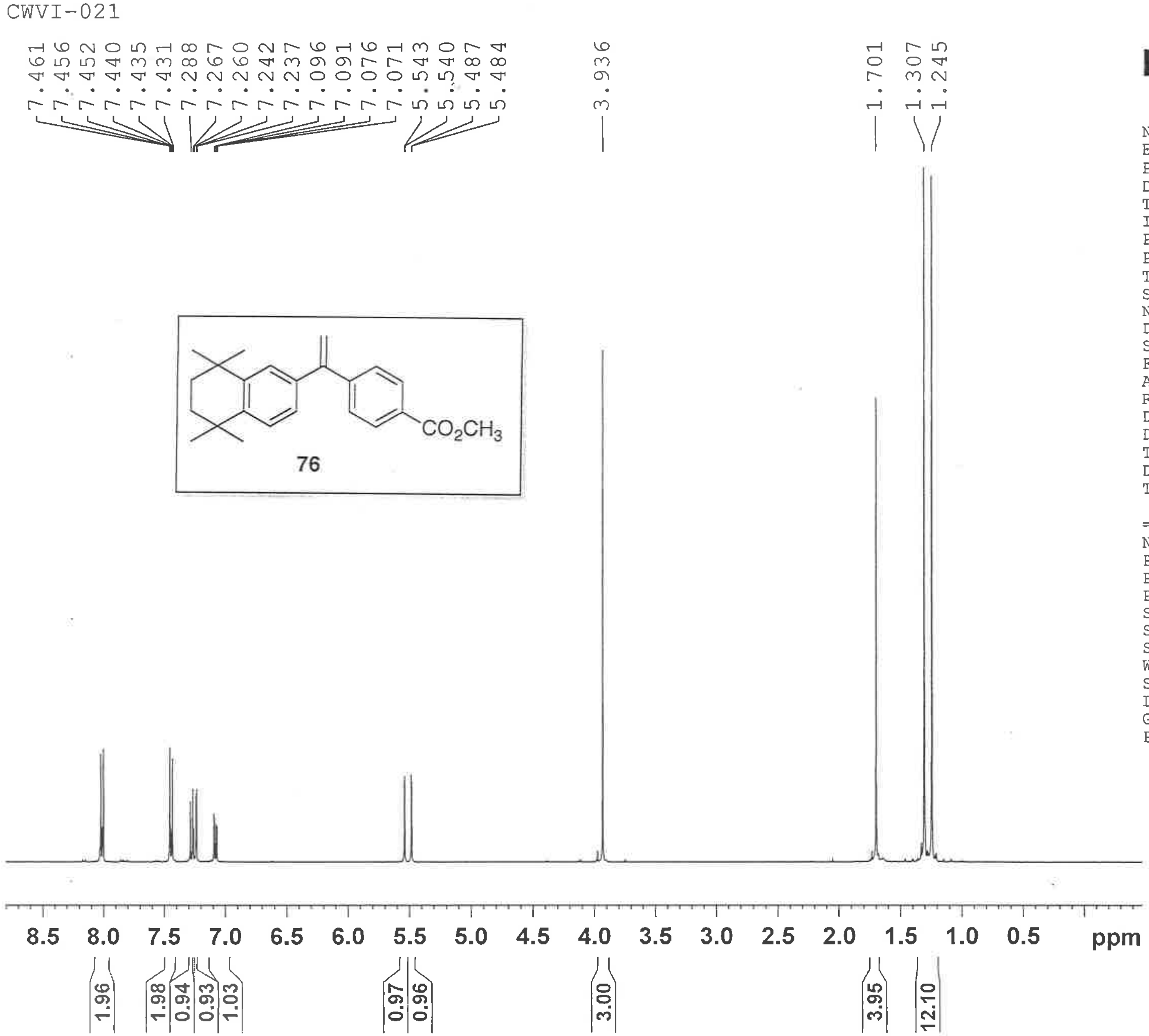

NAME	CWVI-021
EXPNO	1
PROCNO	1
Date_	20160728
Time	15.29
INSTRUM	spect
PROBHD	5 mm PABBO BB-
PULPROG	zg30
TD	65536
SOLVENT	CDCl3
NS	16
DS	2
SWH	8223.685 Hz
FIDRES	0.125483 Hz
AQ	3.9846387 sec
RG	4
DW	60.800 usec
DE	6.50 usec
TE	295.5 K
D1	1.00000000 sec
TD0	1

======== CHANNEL f1 ========

NUC1	1H
P1	14.50 usec
PL1	0.50 dB
PL1W	12.76071072 W
SFO1	400.1324710 MHz
SI	32768
SF	400.1300101 MHz
WDW	EM
SSB	0
LB	0.30 Hz
GB	0
PC	1.00

CWVI-021

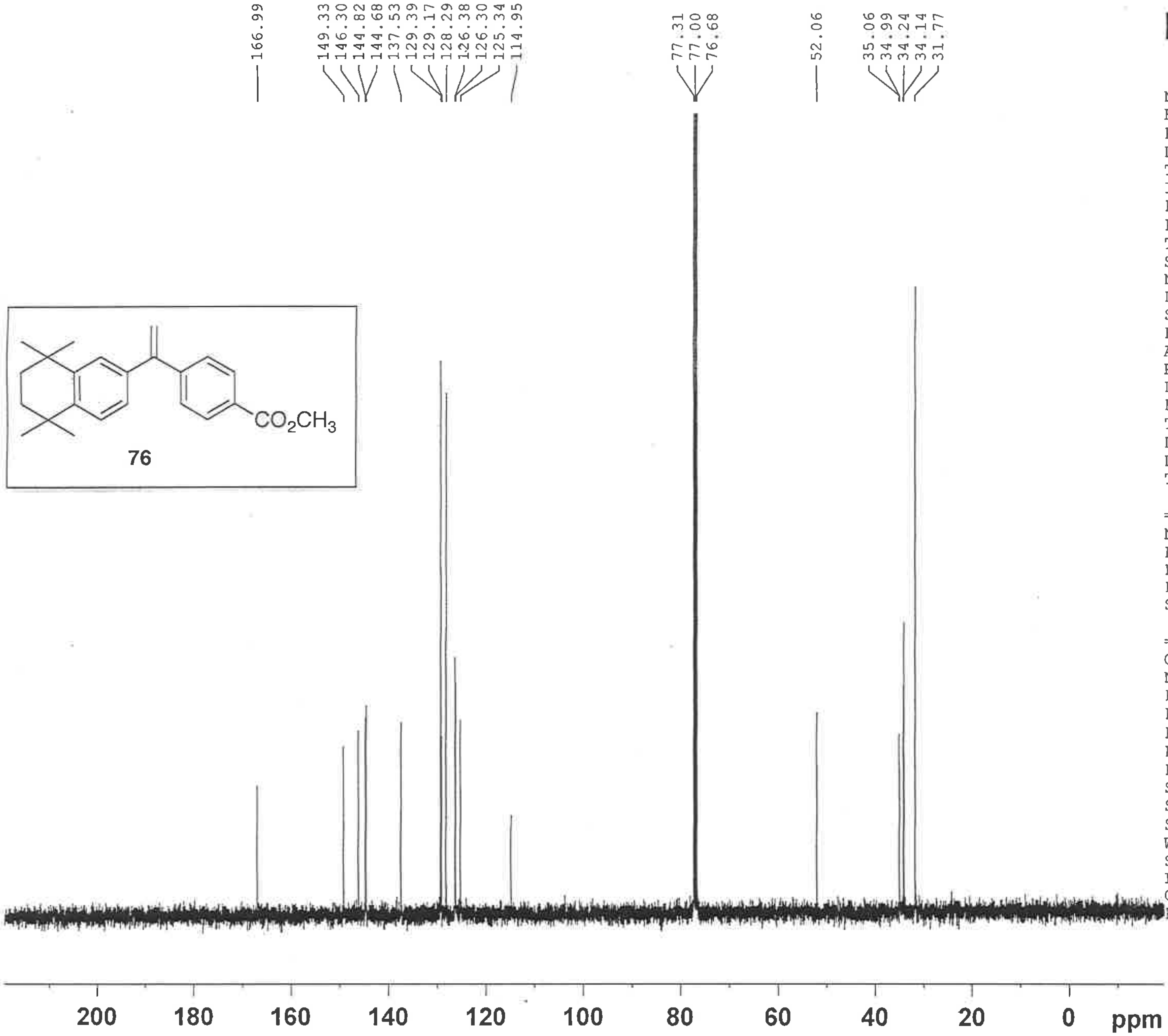

76

NAME	CWVI-021
EXPNO	2
PROCNO	1
Date_	20160728
Time	15.33
INSTRUM	spect
PROBHD	5 mm PABBO BB-
PULPROG	zgig30
TD	65536
SOLVENT	CDCl3
NS	145
DS	4
SWH	24038.461 Hz
FIDRES	0.366798 Hz
AQ	1.3631988 sec
RG	2050
DW	20.800 usec
DE	6.50 usec
TE	295.7 K
D1	2.00000000 sec
D11	0.03000000 sec
TD0	1
======== CHANNEL f1 ========	
NUC1	13C
P1	8.50 usec
PL1	-2.10 dB
PL1W	60.29227829 W
SFO1	100.6228298 MHz
======== CHANNEL f2 ========	
CPDPRG2	waltz16
NUC2	1H
PCPD2	90.00 usec
PL2	-1.80 dB
PL12	17.28 dB
PL2W	21.67079544 W
PL12W	0.26783961 W
SFO2	400.1316005 MHz
SI	32768
SF	100.6127744 MHz
WDW	EM
SSB	0
LB	1.00 Hz
GB	0
PC	1.40

CWVI-025

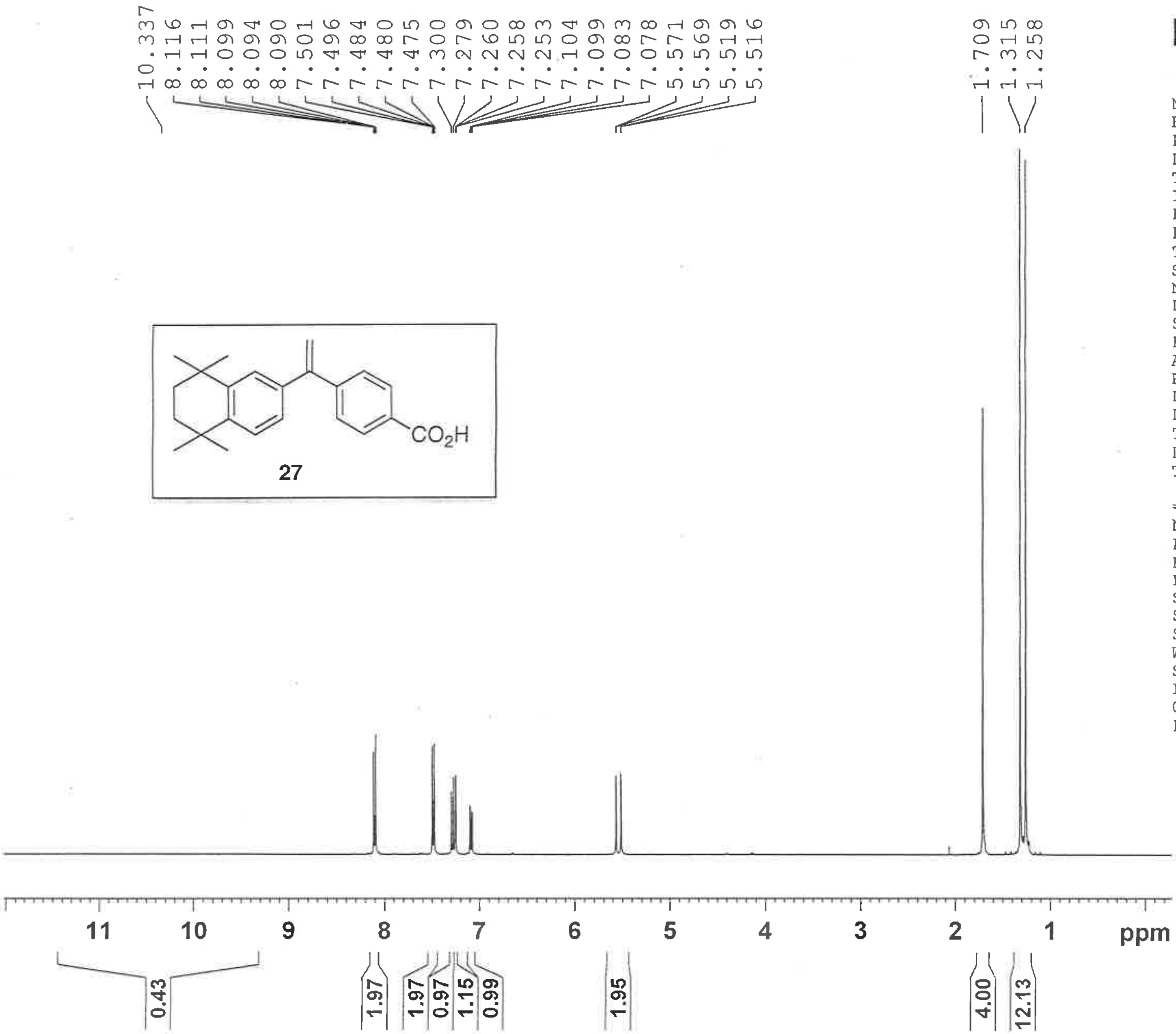

27

BRUKER

NAME	CWVI-025
EXPNO	1
PROCNO	1
Date_	20160729
Time	13.33
INSTRUM	spect
PROBHD	5 mm PABBO BB-
PULPROG	zg30
TD	65536
SOLVENT	CDCl3
NS	16
DS	2
SWH	8223.685 Hz
FIDRES	0.125483 Hz
AQ	3.9846387 sec
RG	4
DW	60.800 usec
DE	6.50 usec
TE	295.8 K
D1	1.00000000 sec
TD0	1

======== CHANNEL f1 ========

NUC1	1H
P1	14.50 usec
PL1	0.50 dB
PL1W	12.76071072 W
SFO1	400.1324710 MHz
SI	32768
SF	400.1300104 MHz
WDW	EM
SSB	0
LB	0.30 Hz
GB	0
PC	1.00

CWVI-025

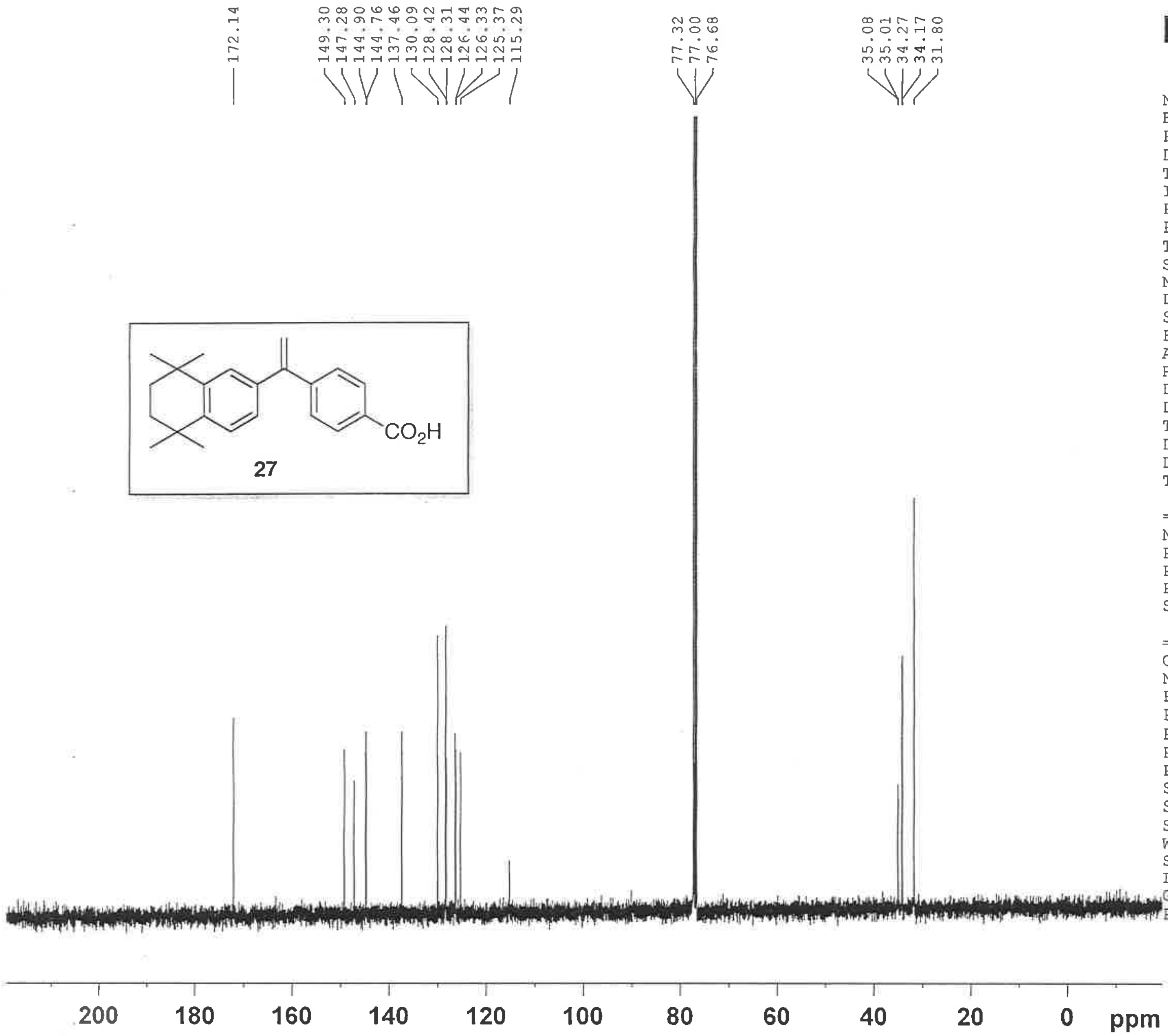

27

NAME	CWVI-025
EXPNO	2
PROCNO	1
Date_	20160729
Time	13.38
INSTRUM	spect
PROBHD	5 mm PABBO BB-
PULPROG	zgig30
TD	65536
SOLVENT	CDCl3
NS	173
DS	4
SWH	24038.461 Hz
FIDRES	0.366798 Hz
AQ	1.3631988 sec
RG	2050
DW	20.800 usec
DE	6.50 usec
TE	296.0 K
D1	2.00000000 sec
D11	0.03000000 sec
TD0	1
======== CHANNEL f1 ========	
NUC1	13C
P1	8.50 usec
PL1	-2.10 dB
PL1W	60.29227829 W
SFO1	100.6228298 MHz
======== CHANNEL f2 ========	
CPDPRG2	waltz16
NUC2	1H
PCPD2	90.00 usec
PL2	-1.80 dB
PL12	17.28 dB
PL2W	21.67079544 W
PL12W	0.26783961 W
SFO2	400.1316005 MHz
SI	32768
SF	100.6127729 MHz
WDW	EM
SSB	0
LB	1.00 Hz
GB	0
PC	1.40

CWV-301

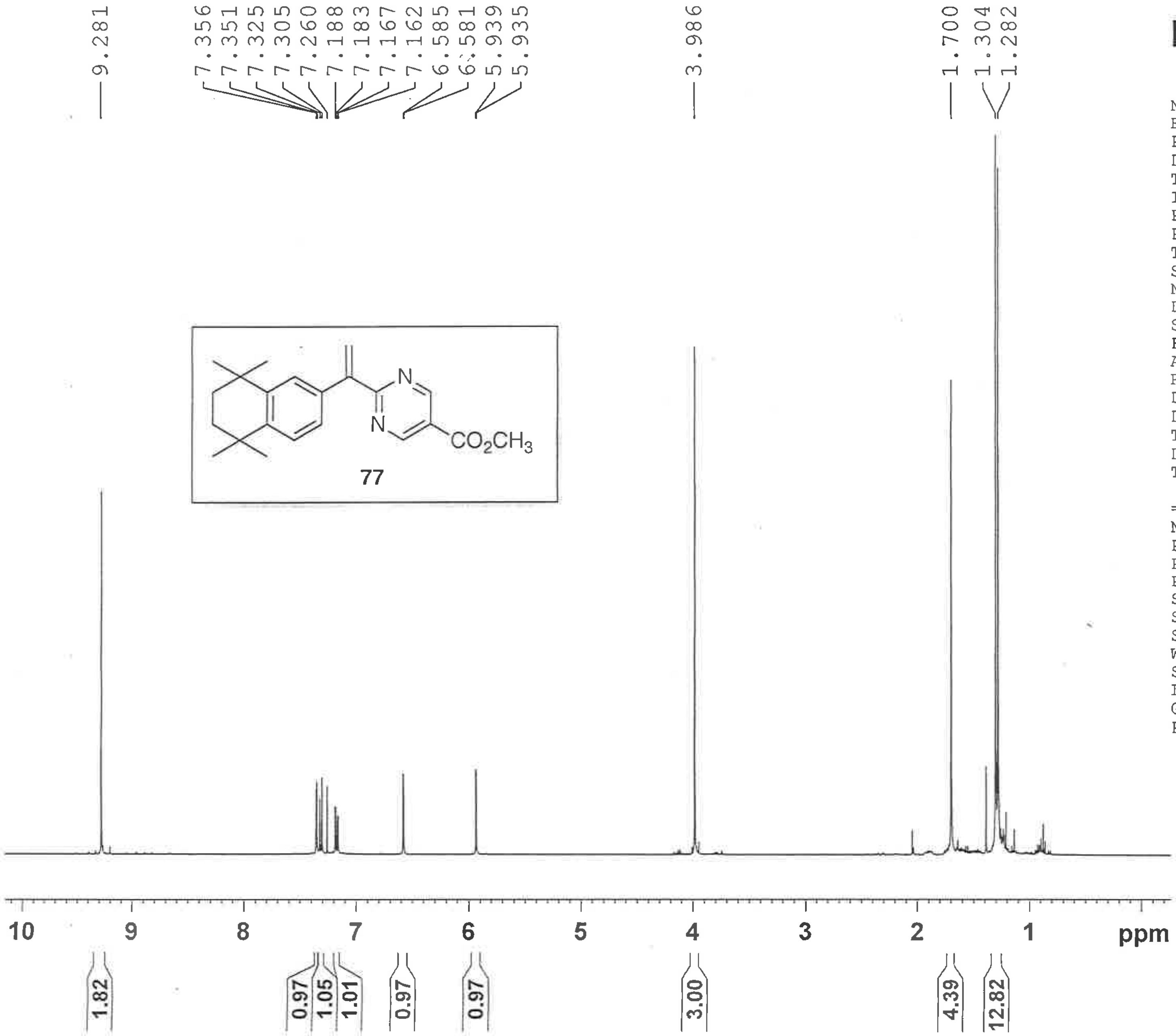

NAME	CWV-301
EXPNO	2
PROCNO	1
Date_	20151224
Time	16.17
INSTRUM	spect
PROBHD	5 mm PABBO BB-
PULPROG	zg30
TD	65536
SOLVENT	CDCl3
NS	16
DS	2
SWH	8223.685 Hz
FIDRES	0.125483 Hz
AQ	3.9846387 sec
RG	4
DW	60.800 usec
DE	6.50 usec
TE	298.2 K
D1	1.00000000 sec
TD0	1

======== CHANNEL f1 ========

NUC1	1H
P1	14.50 usec
PL1	0.50 dB
PL1W	12.76071072 W
SFO1	400.1324710 MHz
SI	32768
SF	400.1300096 MHz
WDW	EM
SSB	0
LB	0.30 Hz
GB	0
PC	1.00

CWV-301

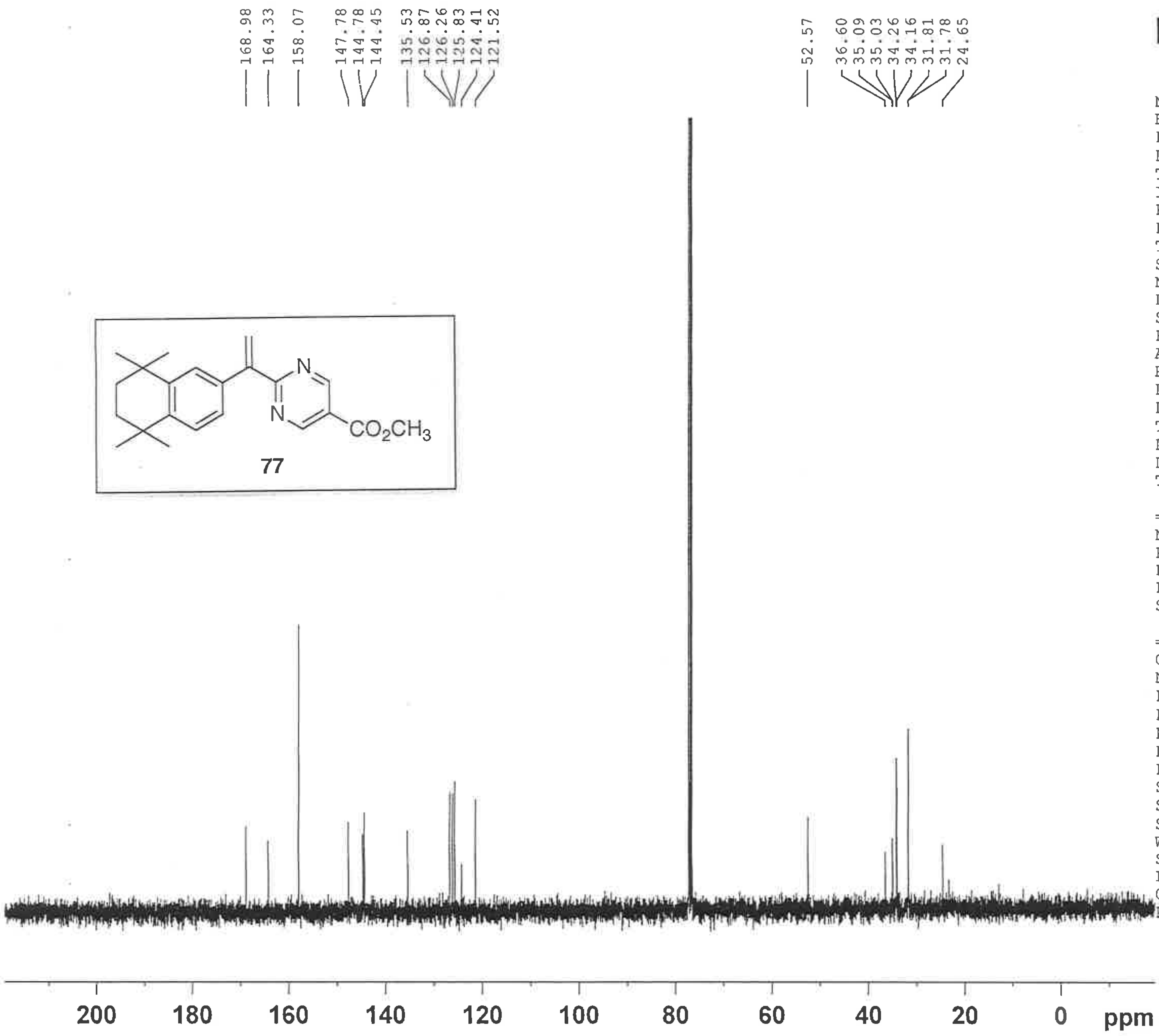

NAME	CWV-301
EXPNO	3
PROCNO	1
Date_	20151224
Time	16.22
INSTRUM	spect
PROBHD	5 mm PABBO BB-
PULPROG	zgig30
TD	65536
SOLVENT	CDCl3
NS	112
DS	4
SWH	24038.461 Hz
FIDRES	0.366798 Hz
AQ	1.3631988 sec
RG	2050
DW	20.800 usec
DE	6.50 usec
TE	298.3 K
D1	2.00000000 sec
D11	0.03000000 sec
TD0	1
======== CHANNEL f1 ========	
NUC1	13C
P1	8.50 usec
PL1	-2.10 dB
PL1W	60.29227829 W
SFO1	100.6228298 MHz
======== CHANNEL f2 ========	
CPDPRG2	waltz16
NUC2	1H
PCPD2	90.00 usec
PL2	-1.80 dB
PL12	17.28 dB
PL2W	21.67079544 W
PL12W	0.26783961 W
SFO2	400.1316005 MHz
SI	32768
SF	100.6127729 MHz
WDW	EM
SSB	0
LB	1.00 Hz
GB	0
PC	1.40

CWVI-009 from EtOAc

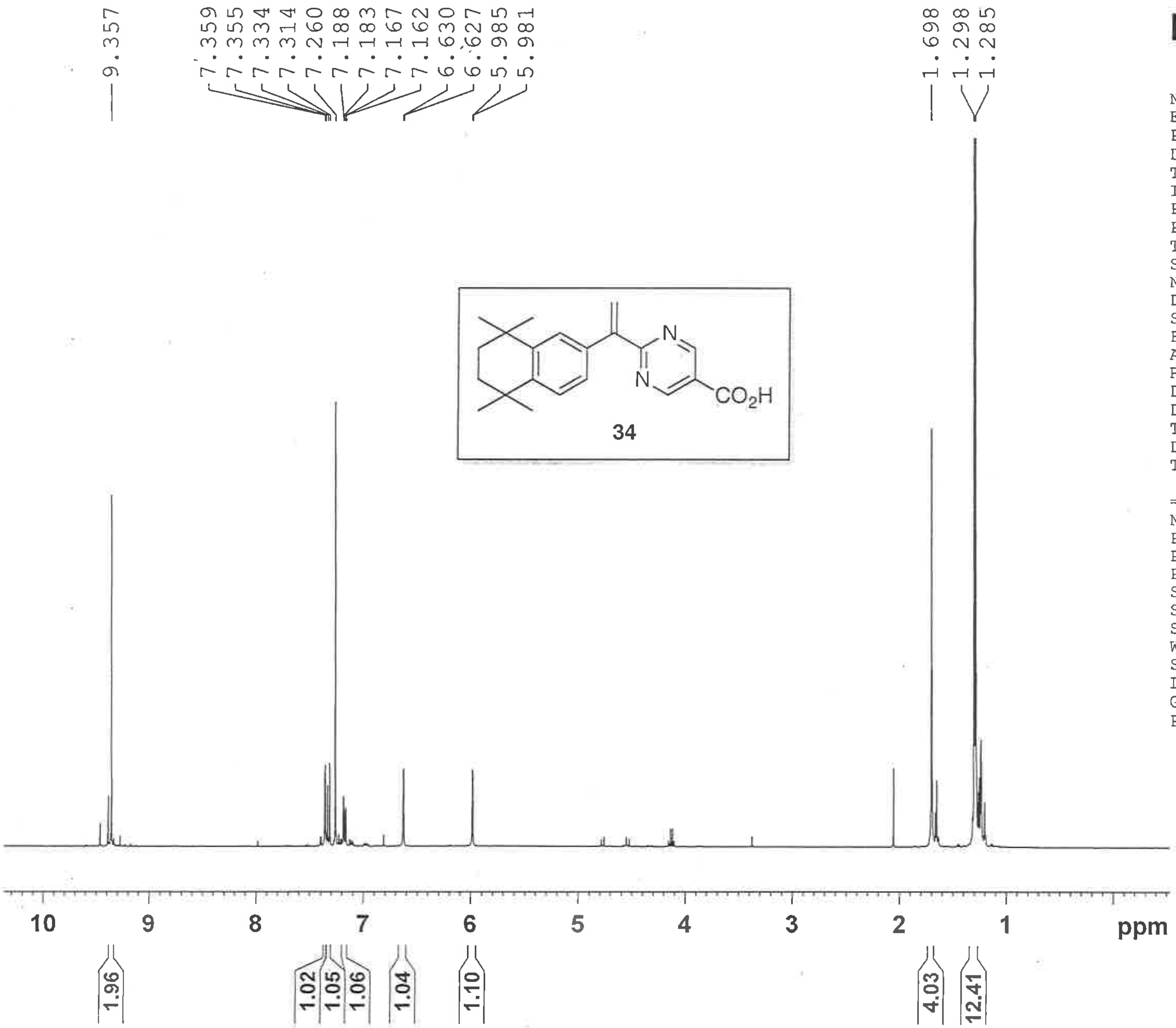

BRUKER

NAME	CWVI-009
EXPNO	2
PROCNO	1
Date_	20160331
Time	9.32
INSTRUM	spect
PROBHD	5 mm PABBO BB-
PULPROG	zg30
TD	65536
SOLVENT	CDCl3
NS	16
DS	2
SWH	8223.685 Hz
FIDRES	0.125483 Hz
AQ	3.9846387 sec
RG	4
DW	60.800 usec
DE	6.50 usec
TE	673.2 K
D1	1.00000000 sec
TD0	1

======== CHANNEL f1 ========

NUC1	1H
P1	14.50 usec
PL1	0.50 dB
PL1W	12.76071072 W
SFO1	400.1324710 MHz
SI	32768
SF	400.1300101 MHz
WDW	EM
SSB	0
LB	0.30 Hz
GB	0
PC	1.00

CWVI-009 from EtOAc

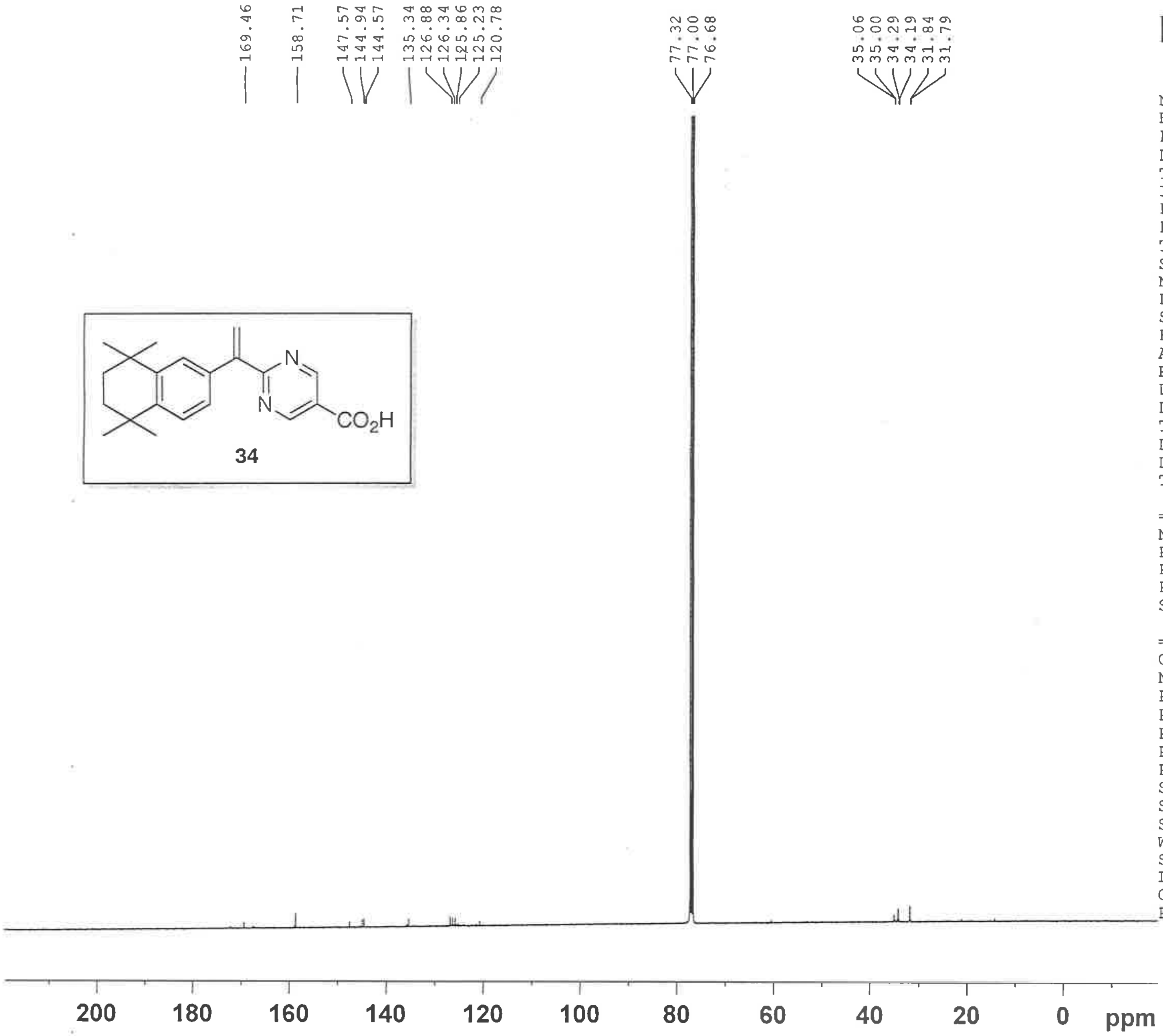

34

BRUKER

NAME	CWVI-009
EXPNO	3
PROCNO	1
Date_	20160401
Time	16.13
INSTRUM	spect
PROBHD	5 mm PABBO BB-
PULPROG	zgig30
TD	65536
SOLVENT	CDCl3
NS	32372
DS	4
SWH	24038.461 Hz
FIDRES	0.366798 Hz
AQ	1.3631988 sec
RG	2050
DW	20.800 usec
DE	6.50 usec
TE	673.2 K
D1	2.00000000 sec
D11	0.03000000 sec
TD0	1

======== CHANNEL f1 ========

NUC1	13C
P1	8.50 usec
PL1	-2.10 dB
PL1W	60.29227829 W
SFO1	100.6228298 MHz

======== CHANNEL f2 ========

CPDPRG2	waltz16
NUC2	1H
PCPD2	90.00 usec
PL2	-1.80 dB
PL12	17.28 dB
PL2W	21.67079544 W
PL12W	0.26783961 W
SFO2	400.1316005 MHz
SI	32768
SF	100.6127722 MHz
WDW	EM
SSB	0
LB	1.00 Hz
GB	0
PC	1.40

CWVI-011

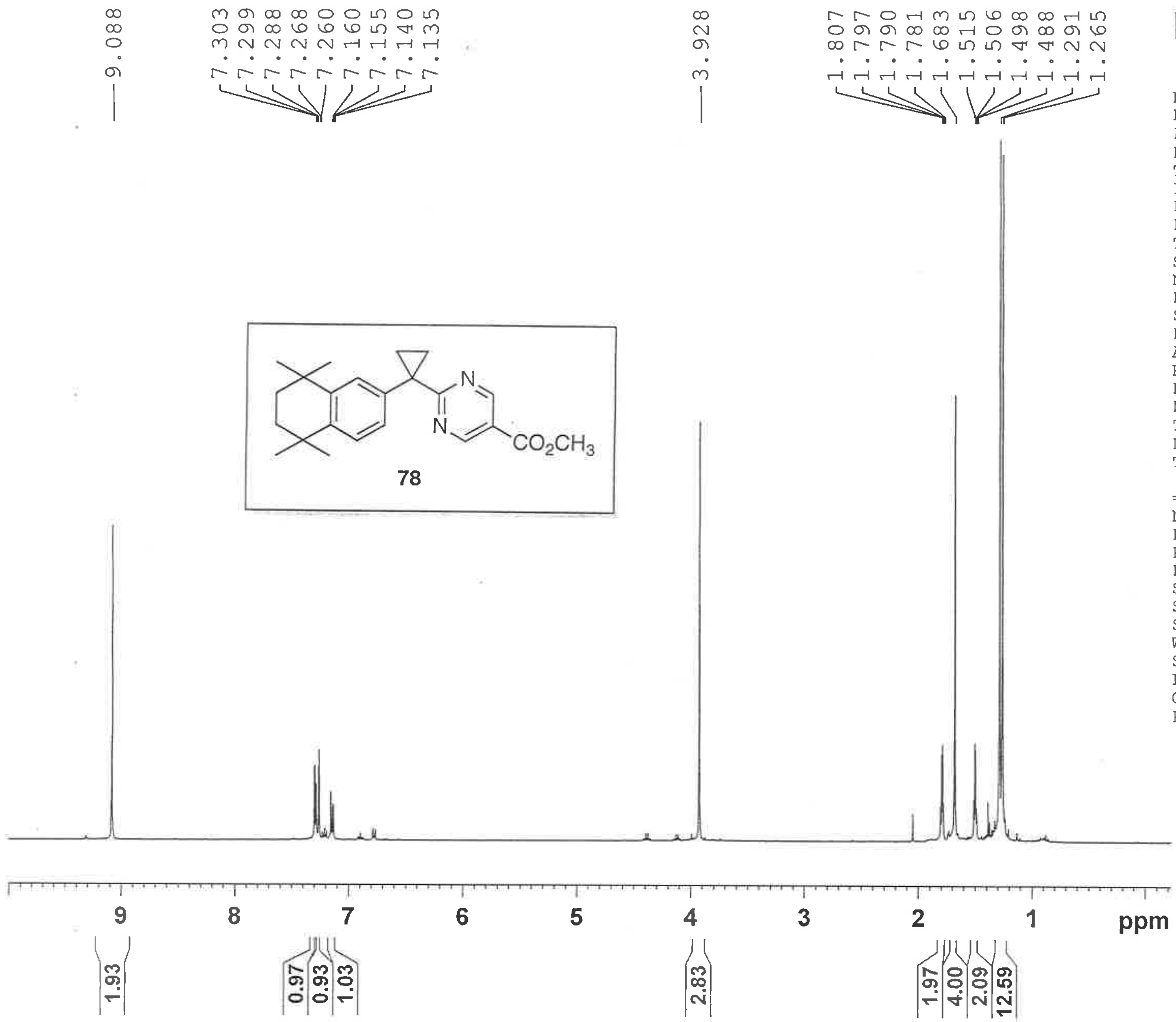

78

NAME	CWVI-011
EXPNO	1
PROCNO	1
Date_	20160317
Time	11.11
INSTRUM	spect
PROBHD	5 mm PABBO BB-
PULPROG	zg30
TD	65536
SOLVENT	CDCl3
NS	16
DS	2
SWH	8223.685 Hz
FIDRES	0.125483 Hz
AQ	3.9846387 sec
RG	4
DW	60.800 usec
DE	6.50 usec
TE	673.2 K
D1	1.00000000 sec
TD0	1

======== CHANNEL f1 ========

NUC1	1H
P1	14.50 usec
PL1	0.50 dB
PL1W	12.76071072 W
SFO1	400.1324710 MHz
SI	32768
SF	400.1300099 MHz
WDW	EM
SSB	0
LB	0.30 Hz
GB	0
PC	1.00

CWVI-011

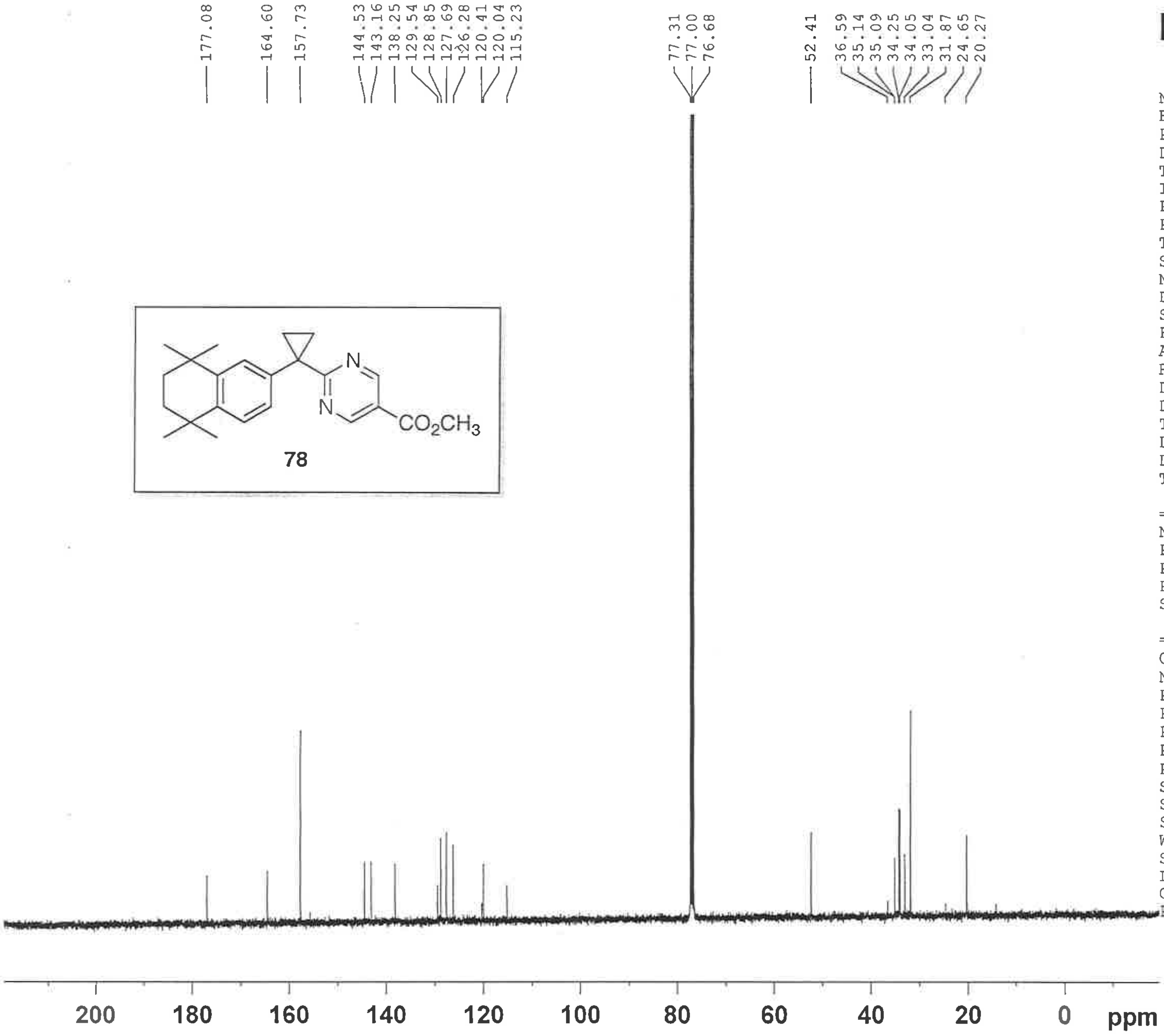

78

BRUKER

NAME	CWVI-011
EXPNO	2
PROCNO	1
Date_	20160317
Time	12.08
INSTRUM	spect
PROBHD	5 mm PABBO BB-
PULPROG	zgig30
TD	65536
SOLVENT	CDCl3
NS	1024
DS	4
SWH	24038.461 Hz
FIDRES	0.366798 Hz
AQ	1.3631988 sec
RG	2050
DW	20.800 usec
DE	6.50 usec
TE	673.2 K
D1	2.00000000 sec
D11	0.03000000 sec
TD0	1

======== CHANNEL f1 ========

NUC1	13C
P1	8.50 usec
PL1	-2.10 dB
PL1W	60.29227829 W
SFO1	100.6228298 MHz

======== CHANNEL f2 ========

CPDPRG2	waltz16
NUC2	1H
PCPD2	90.00 usec
PL2	-1.80 dB
PL12	17.28 dB
PL2W	21.67079544 W
PL12W	0.26783961 W
SFO2	400.1316005 MHz
SI	32768
SF	100.6127736 MHz
WDW	EM
SSB	0
LB	1.00 Hz
GB	0
PC	1.40

CWVI-013

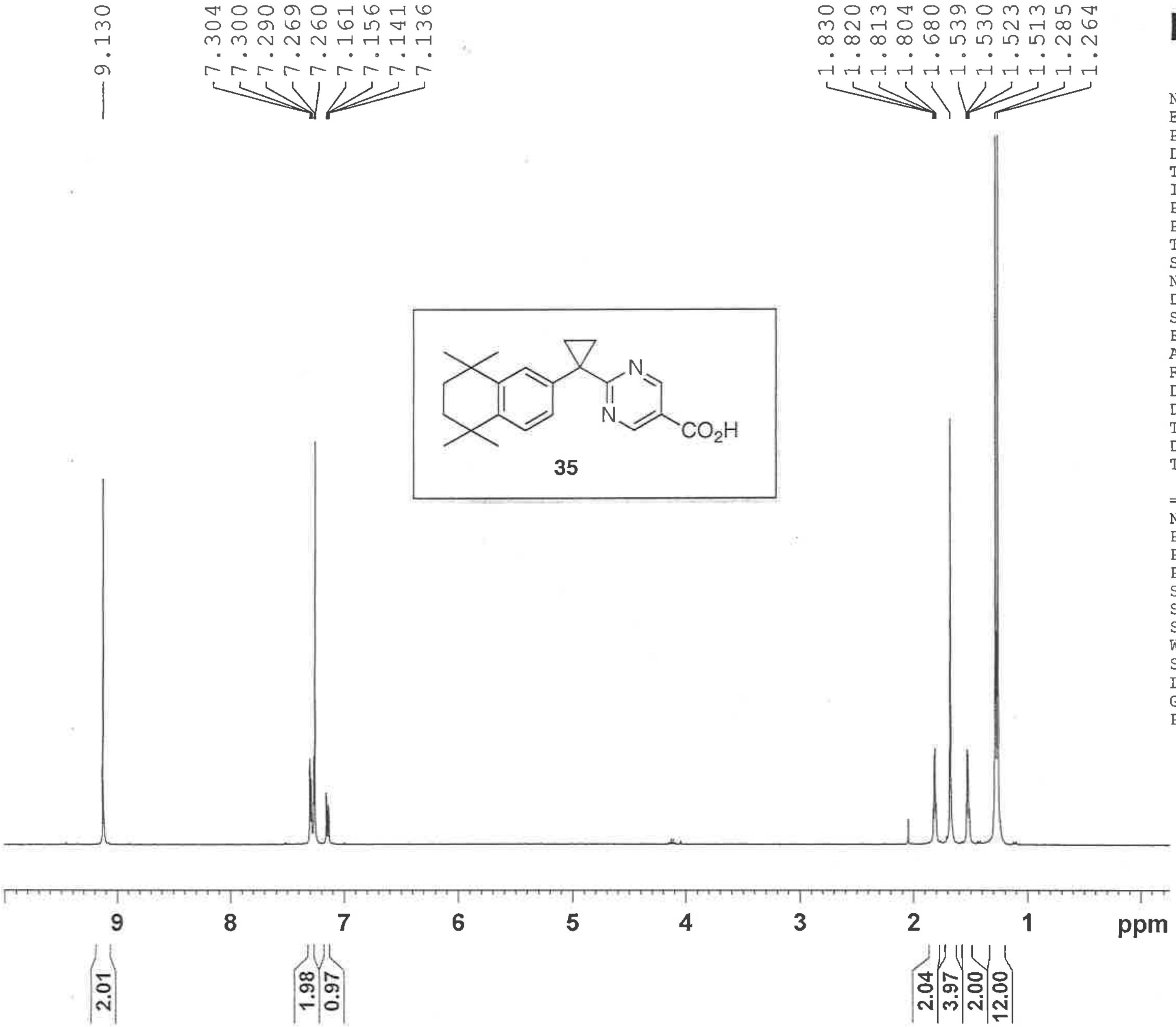

35

NAME	CWVI-013
EXPNO	1
PROCNO	1
Date_	20160323
Time	17.00
INSTRUM	spect
PROBHD	5 mm PABBO BB-
PULPROG	zg30
TD	65536
SOLVENT	CDCl3
NS	16
DS	2
SWH	8223.685 Hz
FIDRES	0.125483 Hz
AQ	3.9846387 sec
RG	4
DW	60.800 usec
DE	6.50 usec
TE	673.2 K
D1	1.00000000 sec
TD0	1
======== CHANNEL f1 ========	
NUC1	1H
P1	14.50 usec
PL1	0.50 dB
PL1W	12.76071072 W
SFO1	400.1324710 MHz
SI	32768
SF	400.1300101 MHz
WDW	EM
SSB	0
LB	0.30 Hz
GB	0
PC	1.00

CWVI-013

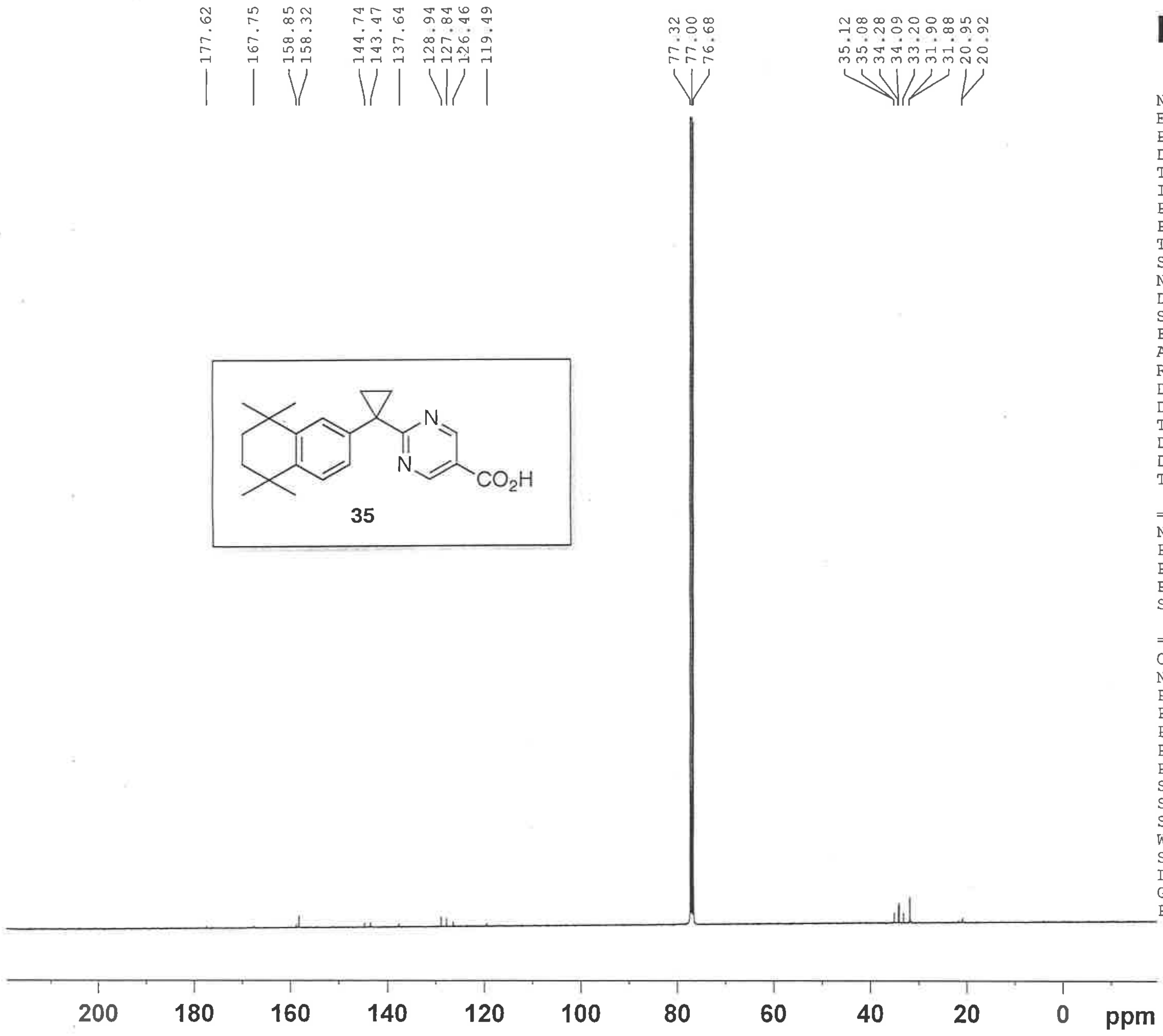

35

BRUKER

NAME	CWVI-013
EXPNO	5
PROCNO	1
Date_	20160329
Time	10.19
INSTRUM	spect
PROBHD	5 mm PABBO BB-
PULPROG	zgig30
TD	65536
SOLVENT	CDCl3
NS	29547
DS	4
SWH	24038.461 Hz
FIDRES	0.366798 Hz
AQ	1.3631988 sec
RG	2050
DW	20.800 usec
DE	6.50 usec
TE	673.2 K
D1	2.00000000 sec
D11	0.03000000 sec
TD0	1

======== CHANNEL f1 ========

NUC1	13C
P1	8.50 usec
PL1	-2.10 dB
PL1W	60.29227829 W
SFO1	100.6228298 MHz

======== CHANNEL f2 ========

CPDPRG2	waltz16
NUC2	1H
PCPD2	90.00 usec
PL2	-1.80 dB
PL12	17.28 dB
PL2W	21.67079544 W
PL12W	0.26783961 W
SFO2	400.1316005 MHz
SI	32768
SF	100.6127722 MHz
WDW	EM
SSB	0
LB	1.00 Hz
GB	0
PC	1.40

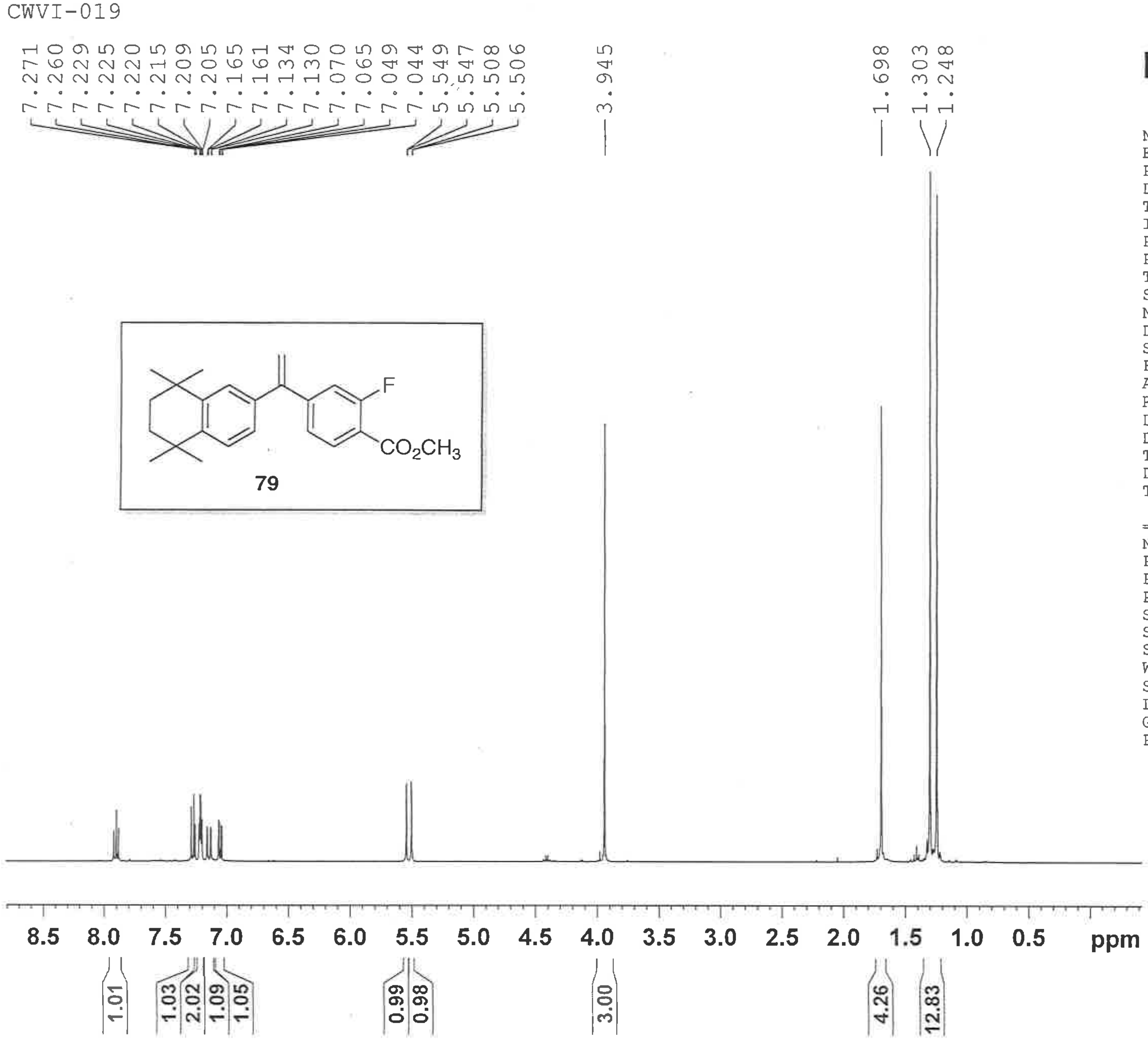

NAME	CWVI-019
EXPNO	1
PROCNO	1
Date_	20160728
Time	15.10
INSTRUM	spect
PROBHD	5 mm PABBO BB-
PULPROG	zg30
TD	65536
SOLVENT	CDCl3
NS	16
DS	2
SWH	8223.685 Hz
FIDRES	0.125483 Hz
AQ	3.9846387 sec
RG	4
DW	60.800 usec
DE	6.50 usec
TE	673.2 K
D1	1.00000000 sec
TD0	1

======== CHANNEL f1 ========

NUC1	1H
P1	14.50 usec
PL1	0.50 dB
PL1W	12.76071072 W
SFO1	400.1324710 MHz
SI	32768
SF	400.1300104 MHz
WDW	EM
SSB	0
LB	0.30 Hz
GB	0
PC	1.00

CWVI-019

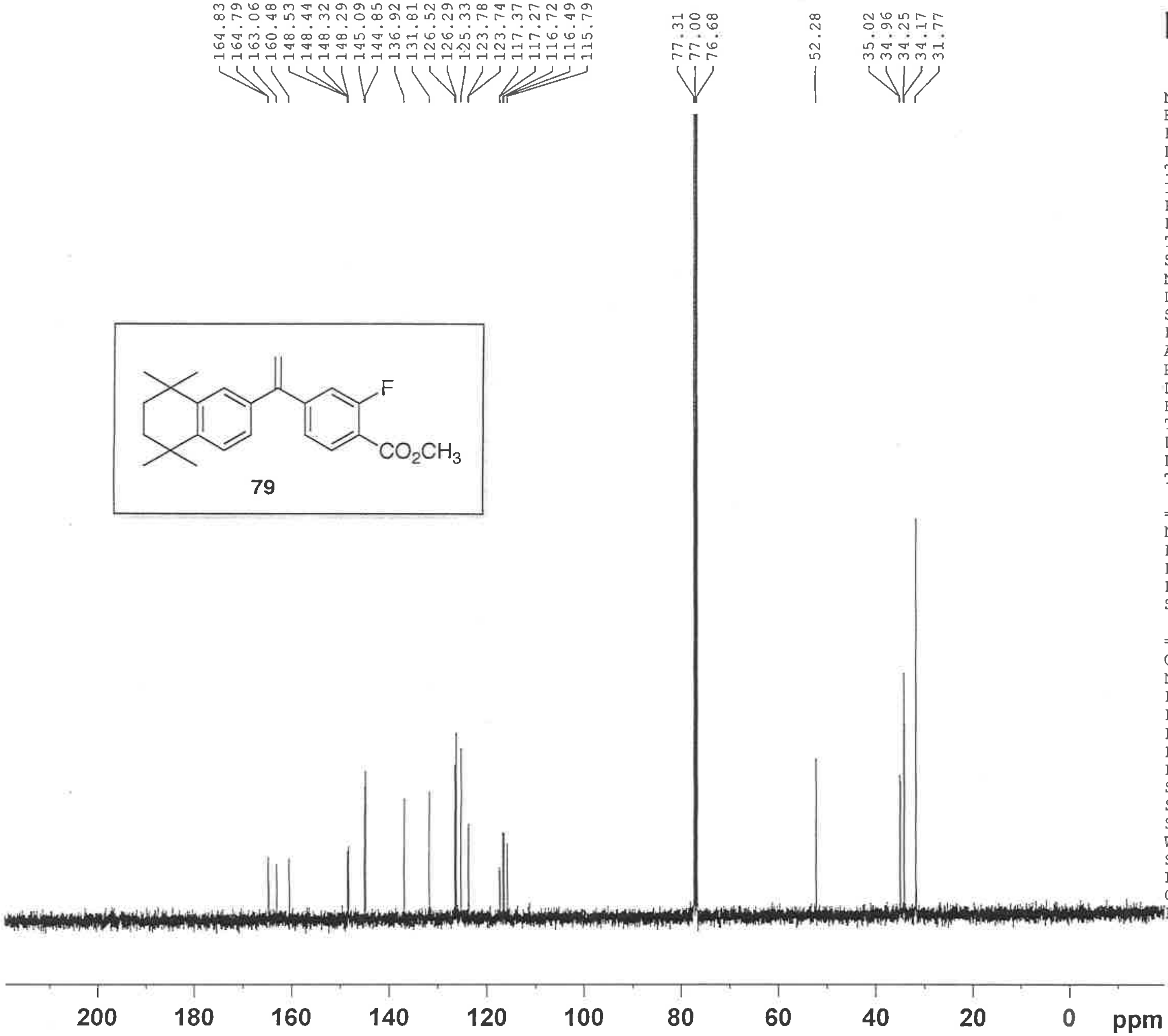

NAME	CWVI-019	
EXPNO	2	
PROCNO	1	
Date_	20160728	
Time	15.17	
INSTRUM	spect	
PROBHD	5 mm PABBO BB-	
PULPROG	zgig30	
TD	65536	
SOLVENT	CDCl3	
NS	219	
DS	4	
SWH	24038.461	Hz
FIDRES	0.366798	Hz
AQ	1.3631988	sec
RG	2050	
DW	20.800	usec
DE	6.50	usec
TE	673.2	K
D1	2.00000000	sec
D11	0.03000000	sec
TD0	1	
======== CHANNEL f1 ========		
NUC1	13C	
P1	8.50	usec
PL1	-2.10	dB
PL1W	60.29227829	W
SFO1	100.6228298	MHz
======== CHANNEL f2 ========		
CPDPRG2	waltz16	
NUC2	1H	
PCPD2	90.00	usec
PL2	-1.80	dB
PL12	17.28	dB
PL2W	21.67079544	W
PL12W	0.26783961	W
SFO2	400.1316005	MHz
SI	32768	
SF	100.6127736	MHz
WDW	EM	
SSB	0	
LB	1.00	Hz
GB	0	
PC	1.40	

CWVI-023

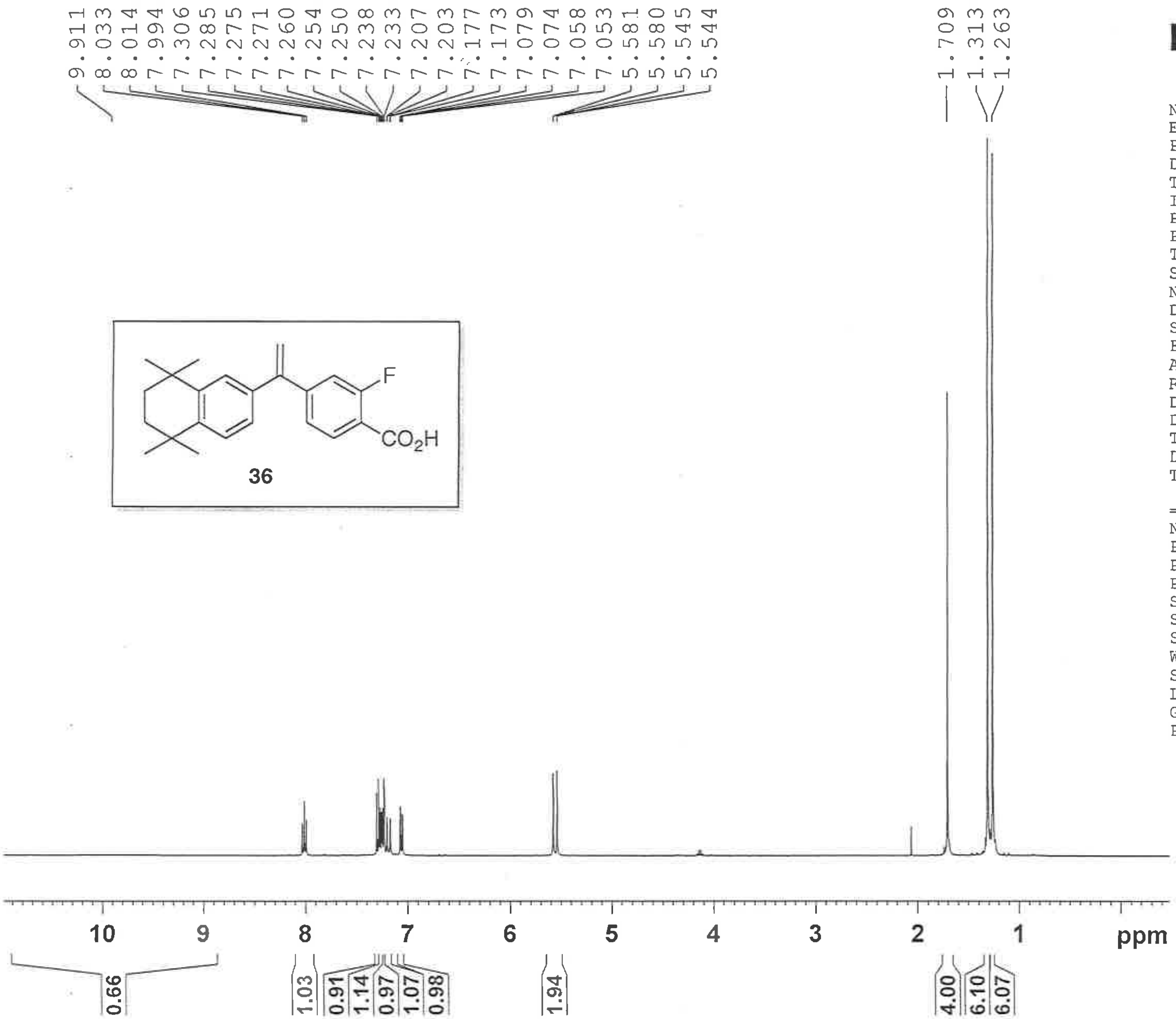

36

NAME	CWVI-023
EXPNO	1
PROCNO	1
Date_	20160729
Time	13.15
INSTRUM	spect
PROBHD	5 mm PABBO BB-
PULPROG	zg30
TD	65536
SOLVENT	CDCl3
NS	16
DS	2
SWH	8223.685 Hz
FIDRES	0.125483 Hz
AQ	3.9846387 sec
RG	4
DW	60.800 usec
DE	6.50 usec
TE	295.8 K
D1	1.00000000 sec
TD0	1

======== CHANNEL f1 ========

NUC1	1H
P1	14.50 usec
PL1	0.50 dB
PL1W	12.76071072 W
SFO1	400.1324710 MHz
SI	32768
SF	400.1300104 MHz
WDW	EM
SSB	0
LB	0.30 Hz
GB	0
PC	1.00

CWVI-023

36

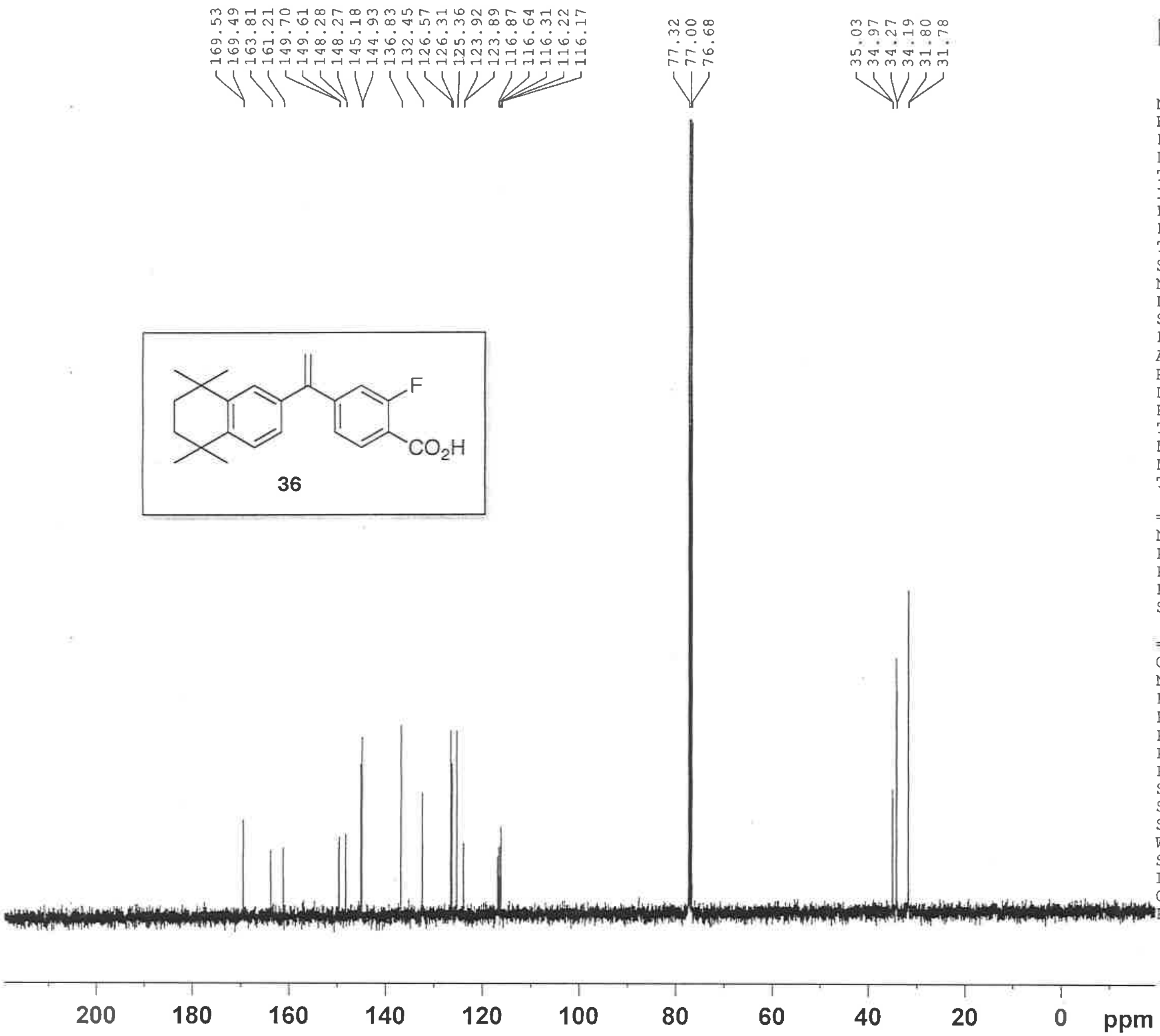

BRUKER

NAME	CWVI-023
EXPNO	2
PROCNO	1
Date_	20160729
Time	13.21
INSTRUM	spect
PROBHD	5 mm PABBO BB-
PULPROG	zgig30
TD	65536
SOLVENT	CDCl3
NS	201
DS	4
SWH	24038.461 Hz
FIDRES	0.366798 Hz
AQ	1.3631988 sec
RG	2050
DW	20.800 usec
DE	6.50 usec
TE	296.0 K
D1	2.00000000 sec
D11	0.03000000 sec
TD0	1
======== CHANNEL f1 ========	
NUC1	13C
P1	8.50 usec
PL1	-2.10 dB
PL1W	60.29227829 W
SFO1	100.6228298 MHz
======== CHANNEL f2 ========	
CPDPRG2	waltz16
NUC2	1H
PCPD2	90.00 usec
PL2	-1.80 dB
PL12	17.28 dB
PL2W	21.67079544 W
PL12W	0.26783961 W
SFO2	400.1316005 MHz
SI	32768
SF	100.6127729 MHz
WDW	EM
SSB	0
LB	1.00 Hz
GB	0
PC	1.40

CWVI-053

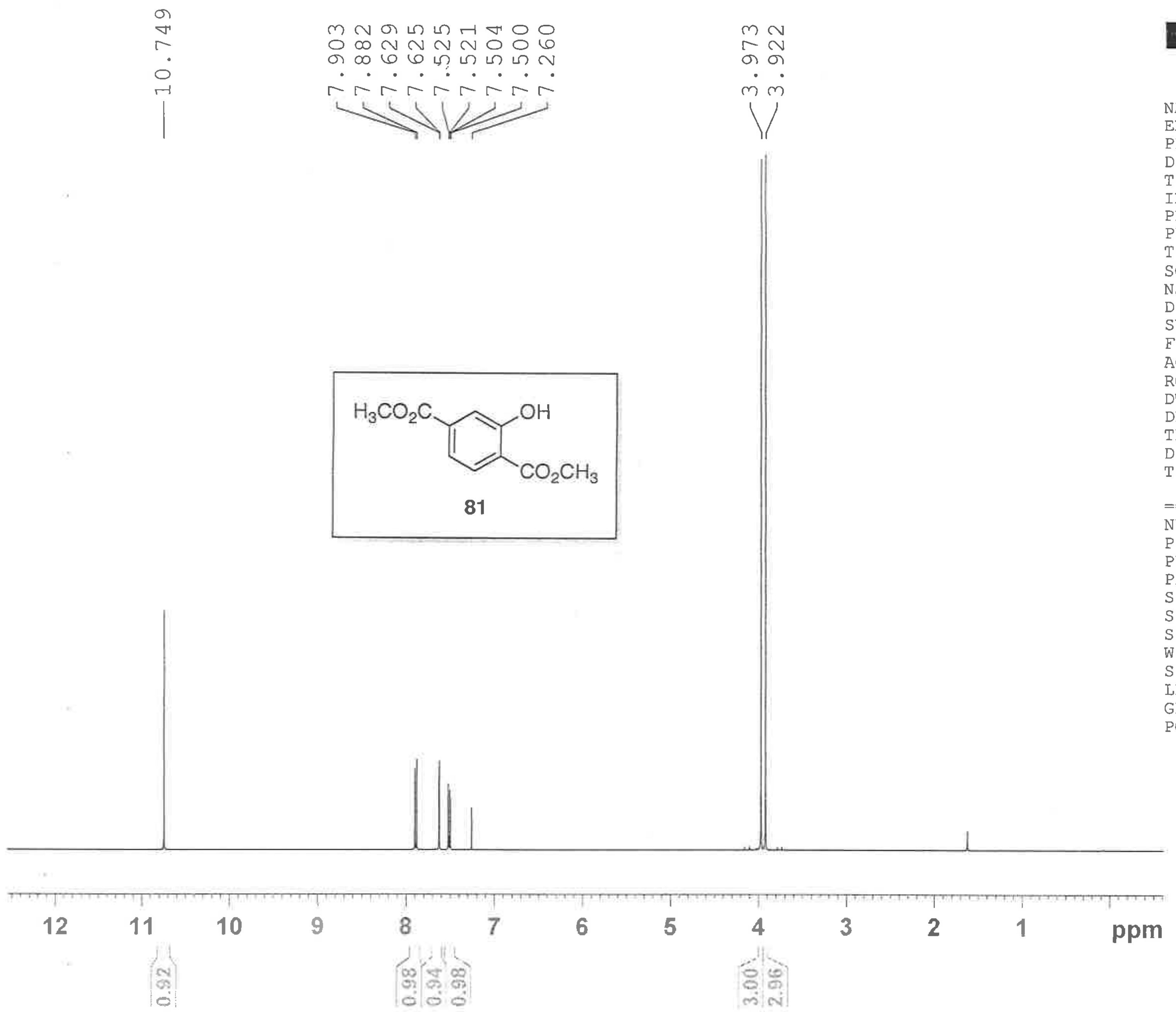

NAME	CWVI-053
EXPNO	1
PROCNO	1
Date_	20161021
Time	10.55
INSTRUM	spect
PROBHD	5 mm PABBO BB-
PULPROG	zg30
TD	65536
SOLVENT	CDCl3
NS	16
DS	2
SWH	8223.685 Hz
FIDRES	0.125483 Hz
AQ	3.9846387 sec
RG	4
DW	60.800 usec
DE	6.50 usec
TE	673.2 K
D1	1.00000000 sec
TD0	1

======== CHANNEL f1 ========

NUC1	1H
P1	14.50 usec
PL1	0.50 dB
PL1W	12.76071072 W
SFO1	400.1324710 MHz
SI	32768
SF	400.1300101 MHz
WDW	EM
SSB	0
LB	0.30 Hz
GB	0
PC	1.00

CWVI-053

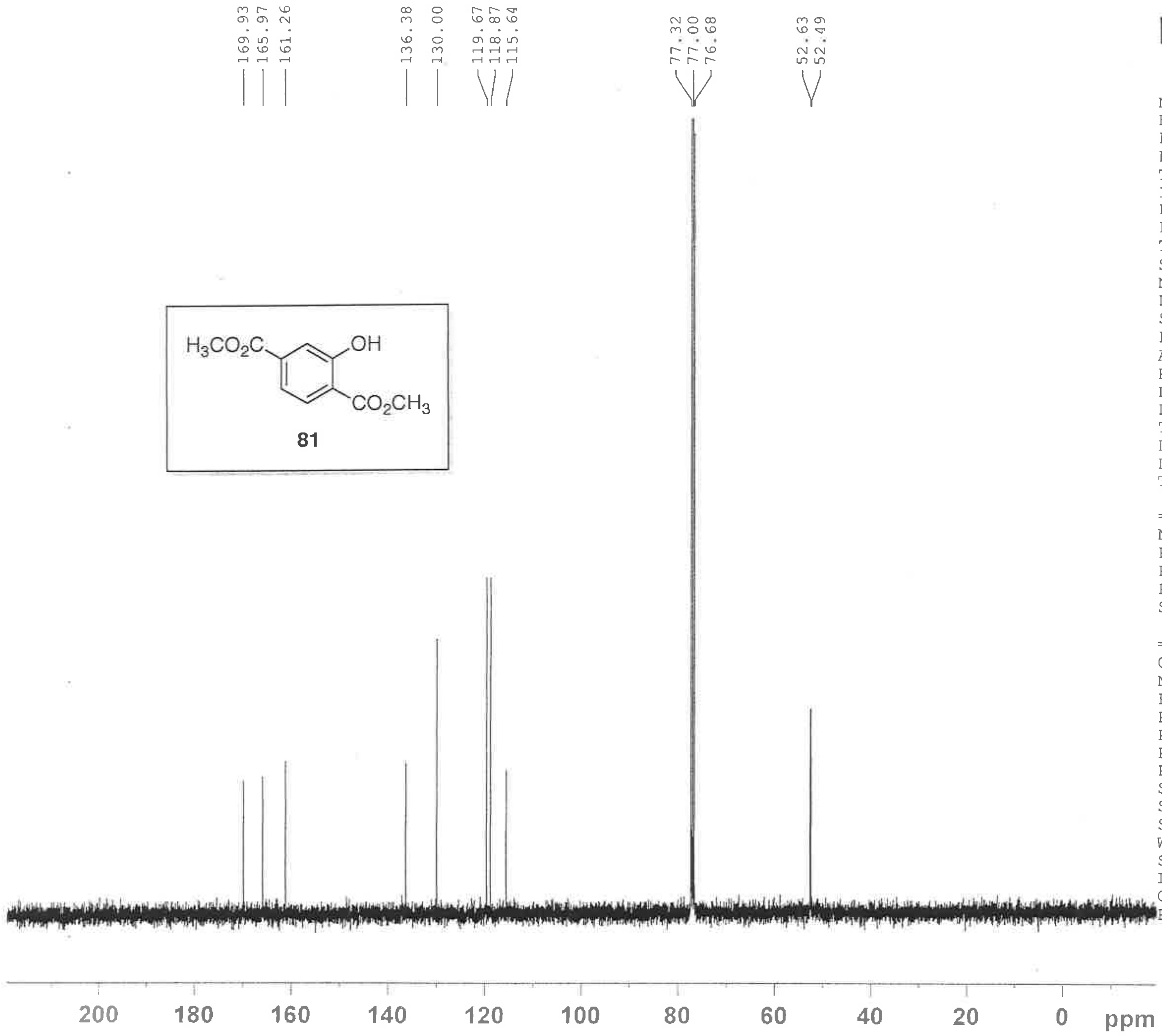

BRUKER

NAME	CWVI-053
EXPNO	2
PROCNO	1
Date_	20161021
Time	11.09
INSTRUM	spect
PROBHD	5 mm PABBO BB-
PULPROG	zgig30
TD	65536
SOLVENT	CDCl3
NS	118
DS	4
SWH	24038.461 Hz
FIDRES	0.366798 Hz
AQ	1.3631988 sec
RG	2050
DW	20.800 usec
DE	6.50 usec
TE	673.2 K
D1	2.00000000 sec
D11	0.03000000 sec
TD0	1
======== CHANNEL f1 ========	
NUC1	13C
P1	8.50 usec
PL1	-2.10 dB
PL1W	60.29227829 W
SFO1	100.6228298 MHz
======== CHANNEL f2 ========	
CPDPRG2	waltz16
NUC2	1H
PCPD2	90.00 usec
PL2	-1.80 dB
PL12	17.28 dB
PL2W	21.67079544 W
PL12W	0.26783961 W
SFO2	400.1316005 MHz
SI	32768
SF	100.6127729 MHz
WDW	EM
SSB	0
LB	1.00 Hz
GB	0
PC	1.40

CWVI-059 Middle Spot

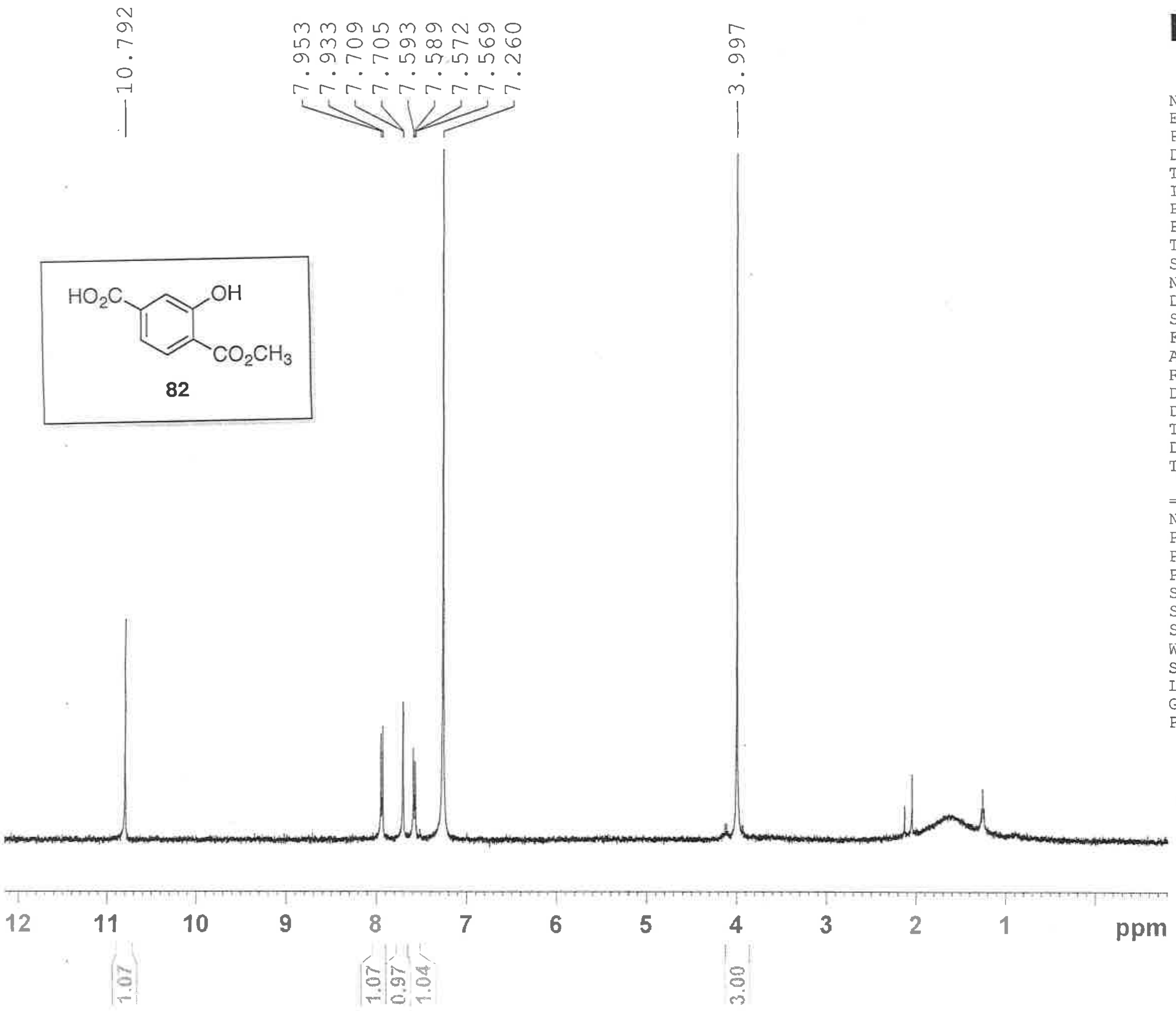

82

NAME	CWVI-059
EXPNO	2
PROCNO	1
Date_	20161107
Time	14.30
INSTRUM	spect
PROBHD	5 mm PABBO BB-
PULPROG	zg30
TD	65536
SOLVENT	CDCl3
NS	16
DS	2
SWH	8223.685 Hz
FIDRES	0.125483 Hz
AQ	3.9846387 sec
RG	4
DW	60.800 usec
DE	6.50 usec
TE	673.2 K
D1	1.00000000 sec
TD0	1
======== CHANNEL f1 ========	
NUC1	1H
P1	14.50 usec
PL1	0.50 dB
PL1W	12.76071072 W
SFO1	400.1324710 MHz
SI	32768
SF	400.1300104 MHz
WDW	EM
SSB	0
LB	0.30 Hz
GB	0
PC	1.00

CWVI-061 f12-16

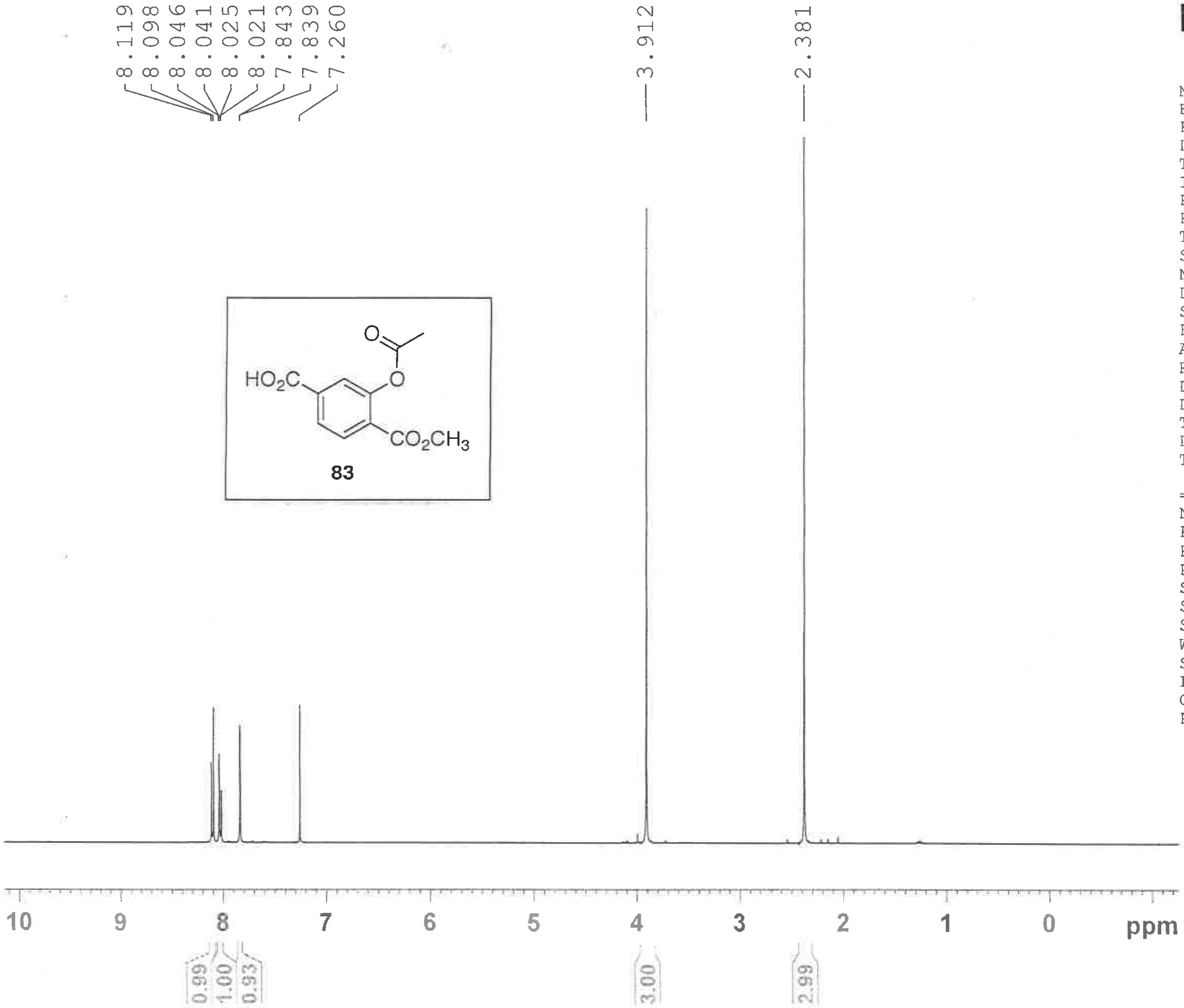

NAME	CWVI-061
EXPNO	2
PROCNO	1
Date_	20161122
Time	12.21
INSTRUM	spect
PROBHD	5 mm PABBO BB-
PULPROG	zg30
TD	65536
SOLVENT	CDCl3
NS	16
DS	2
SWH	8223.685 Hz
FIDRES	0.125483 Hz
AQ	3.9846387 sec
RG	4
DW	60.800 usec
DE	6.50 usec
TE	673.2 K
D1	1.00000000 sec
TD0	1

======== CHANNEL f1 ========

NUC1	1H
P1	14.50 usec
PL1	0.50 dB
PL1W	12.76071072 W
SFO1	400.1324710 MHz
SI	32768
SF	400.1300104 MHz
WDW	EM
SSB	0
LB	0.30 Hz
GB	0
PC	1.00

CWVI-061 f12-16

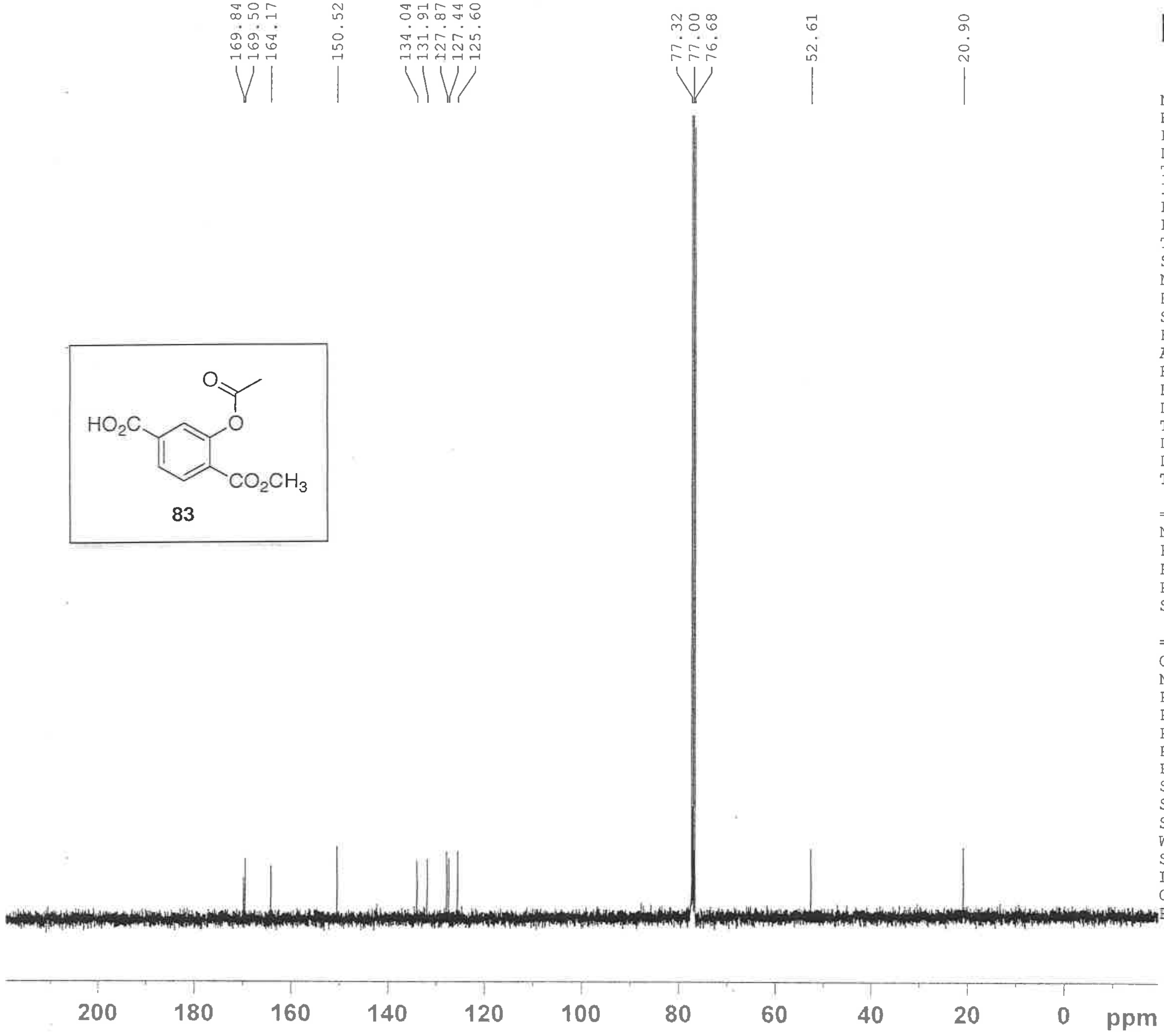

83

BRUKER

NAME	CWVI-061
EXPNO	3
PROCNO	1
Date_	20161122
Time	12.31
INSTRUM	spect
PROBHD	5 mm PABBO BB-
PULPROG	zgig30
TD	65536
SOLVENT	CDCl3
NS	271
DS	4
SWH	24038.461 Hz
FIDRES	0.366798 Hz
AQ	1.3631988 sec
RG	2050
DW	20.800 usec
DE	6.50 usec
TE	673.2 K
D1	2.00000000 sec
D11	0.03000000 sec
TD0	1

======== CHANNEL f1 ========

NUC1	13C
P1	8.50 usec
PL1	-2.10 dB
PL1W	60.29227829 W
SFO1	100.6228298 MHz

======== CHANNEL f2 ========

CPDPRG2	waltz16
NUC2	1H
PCPD2	90.00 usec
PL2	-1.80 dB
PL12	17.28 dB
PL2W	21.67079544 W
PL12W	0.26783961 W
SFO2	400.1316005 MHz
SI	32768
SF	100.6127722 MHz
WDW	EM
SSB	0
LB	1.00 Hz
GB	0
PC	1.40

CWVI-069 f5-9

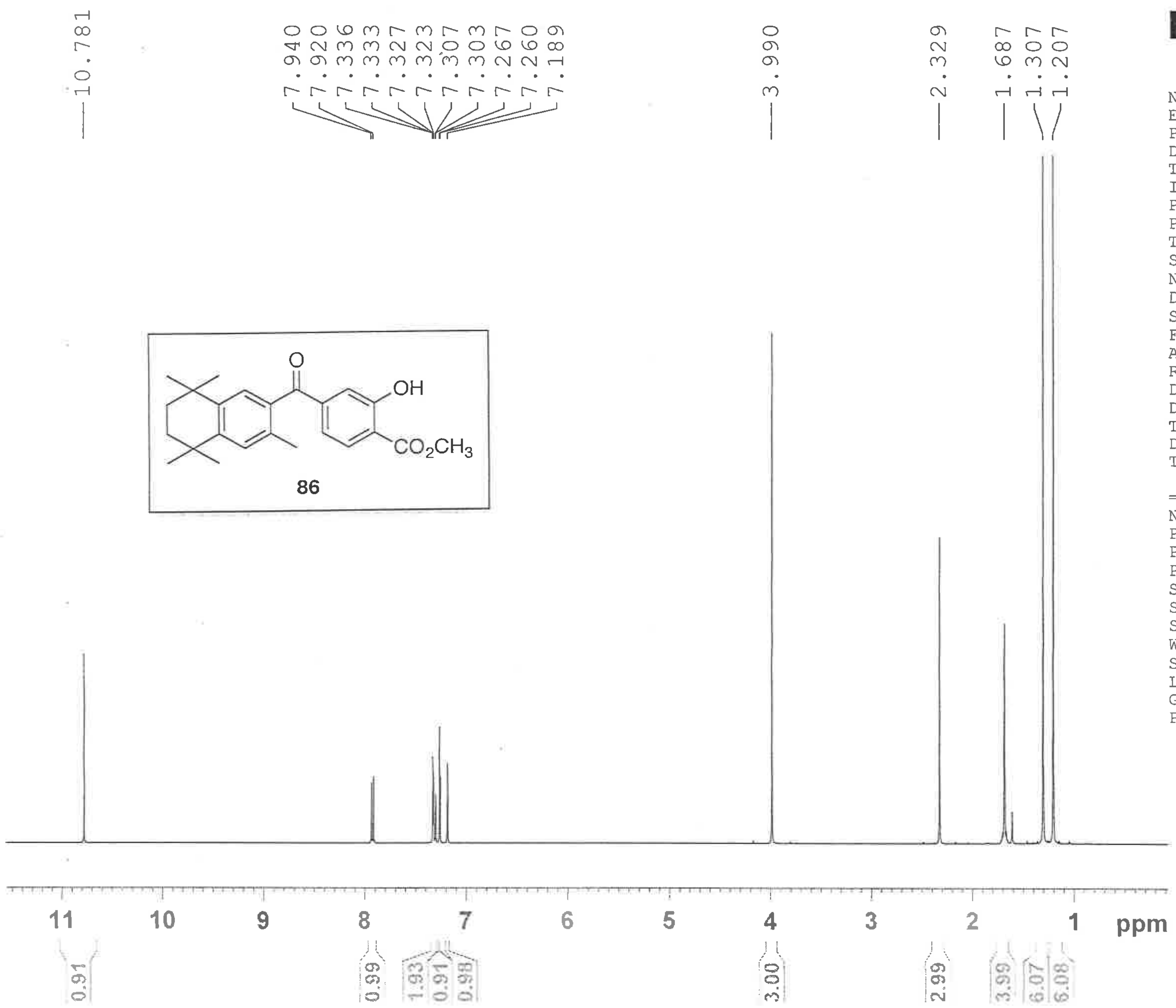

BRUKER

NAME	CWVI-069
EXPNO	1
PROCNO	1
Date_	20170210
Time	16.38
INSTRUM	spect
PROBHD	5 mm PABBO BB-
PULPROG	zg30
TD	65536
SOLVENT	CDCl3
NS	16
DS	2
SWH	8223.685 Hz
FIDRES	0.125483 Hz
AQ	3.9846387 sec
RG	4
DW	60.800 usec
DE	6.50 usec
TE	673.2 K
D1	1.00000000 sec
TD0	1

======== CHANNEL f1 ========

NUC1	1H
P1	14.50 usec
PL1	0.50 dB
PL1W	12.76071072 W
SFO1	400.1324710 MHz
SI	32768
SF	400.1300104 MHz
WDW	EM
SSB	0
LB	0.30 Hz
GB	0
PC	1.00

CWVI-069 f5-9

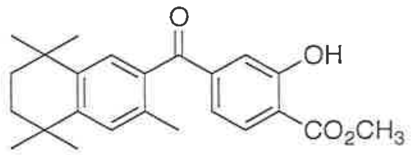

86

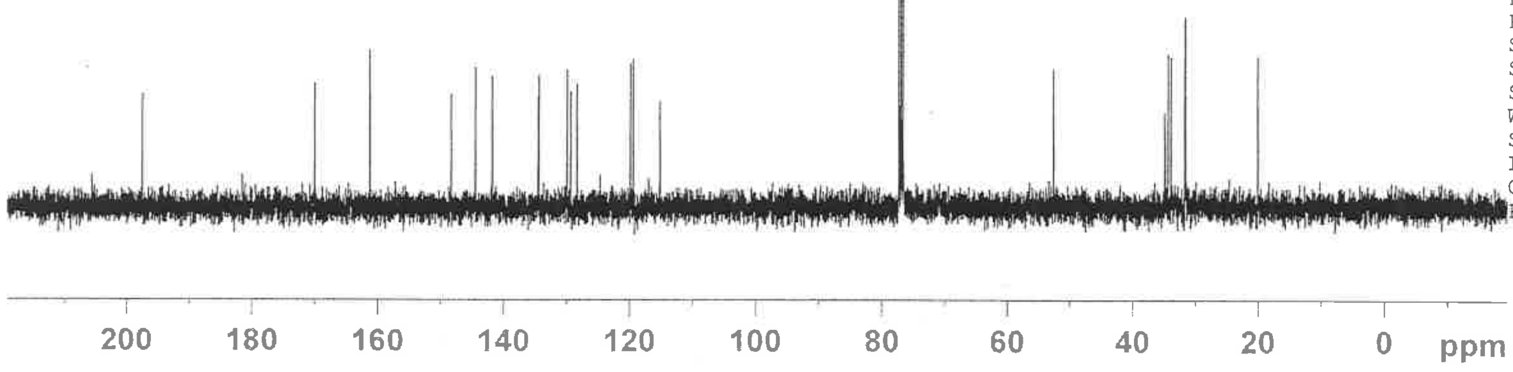

BRUKER

NAME	CWVI-069	
EXPNO	2	
PROCNO	1	
Date_	20170210	
Time	16.46	
INSTRUM	spect	
PROBHD	5 mm PABBO BB-	
PULPROG	zgig30	
TD	65536	
SOLVENT	CDCl3	
NS	79	
DS	4	
SWH	24038.461	Hz
FIDRES	0.366798	Hz
AQ	1.3631988	sec
RG	2050	
DW	20.800	usec
DE	6.50	usec
TE	673.2	K
D1	2.00000000	sec
D11	0.03000000	sec
TD0	1	
======== CHANNEL f1 ========		
NUC1	13C	
P1	8.50	usec
PL1	-2.10	dB
PL1W	60.29227829	W
SFO1	100.6228298	MHz
======== CHANNEL f2 ========		
CPDPRG2	waltz16	
NUC2	1H	
PCPD2	90.00	usec
PL2	-1.80	dB
PL12	17.28	dB
PL2W	21.67079544	W
PL12W	0.26783961	W
SFO2	400.1316005	MHz
SI	32768	
SF	100.6127729	MHz
WDW	EM	
SSB	0	
LB	1.00	Hz
GB	0	
PC	1.40	

CWVI-071 f6B

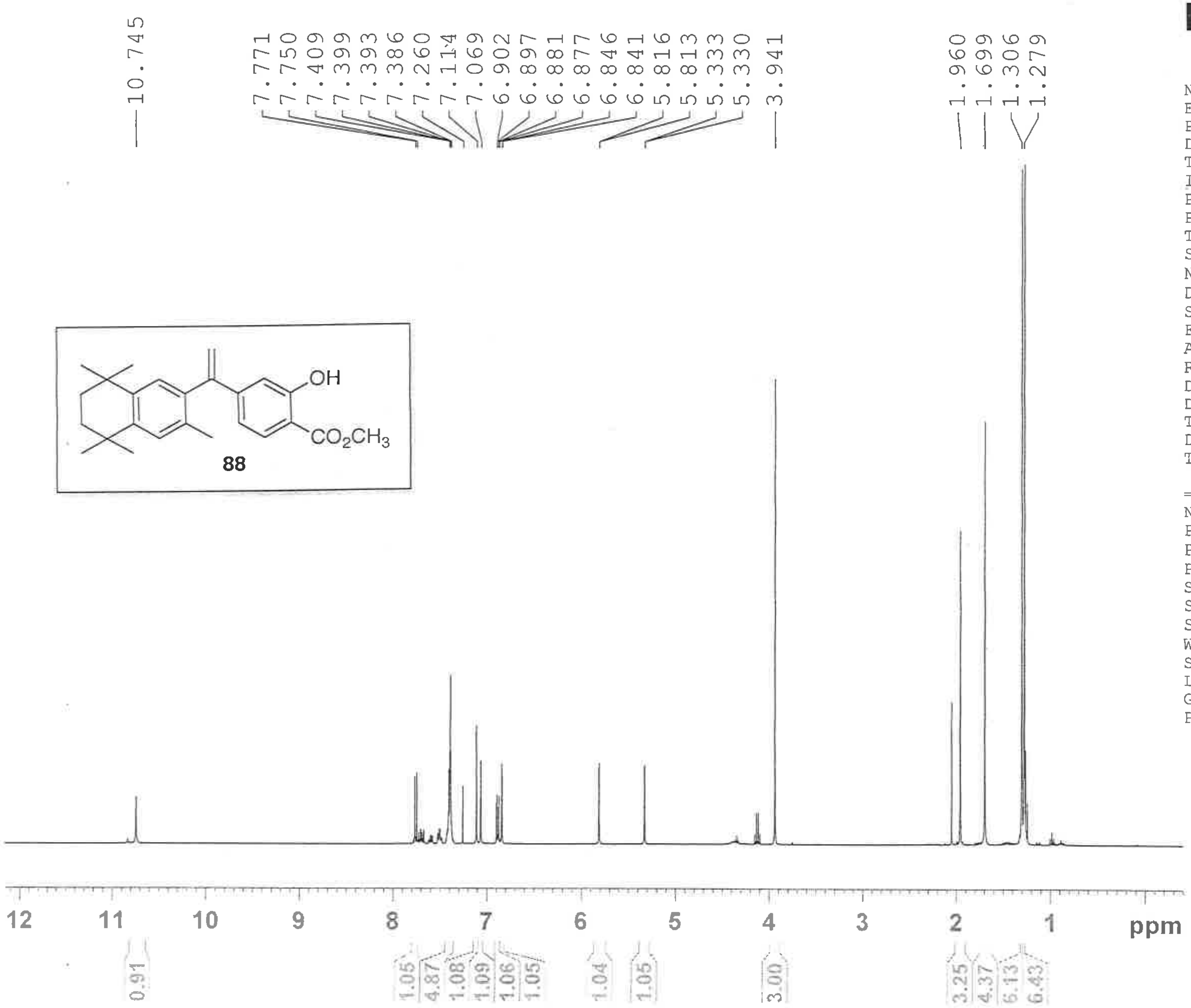

88

BRUKER

NAME	CWVI-071
EXPNO	1
PROCNO	1
Date_	20170222
Time_	14.04
INSTRUM	spect
PROBHD	5 mm PABBO BB-
PULPROG	zg30
TD	65536
SOLVENT	CDCl3
NS	16
DS	2
SWH	8223.685 Hz
FIDRES	0.125483 Hz
AQ	3.9846387 sec
RG	4
DW	60.800 usec
DE	6.50 usec
TE	673.2 K
D1	1.00000000 sec
TD0	1

======== CHANNEL f1 ========

NUC1	1H
P1	14.50 usec
PL1	0.50 dB
PL1W	12.76071072 W
SFO1	400.1324710 MHz
SI	32768
SF	400.1300104 MHz
WDW	EM
SSB	0
LB	0.30 Hz
GB	0
PC	1.00

CWVI-071 f6B

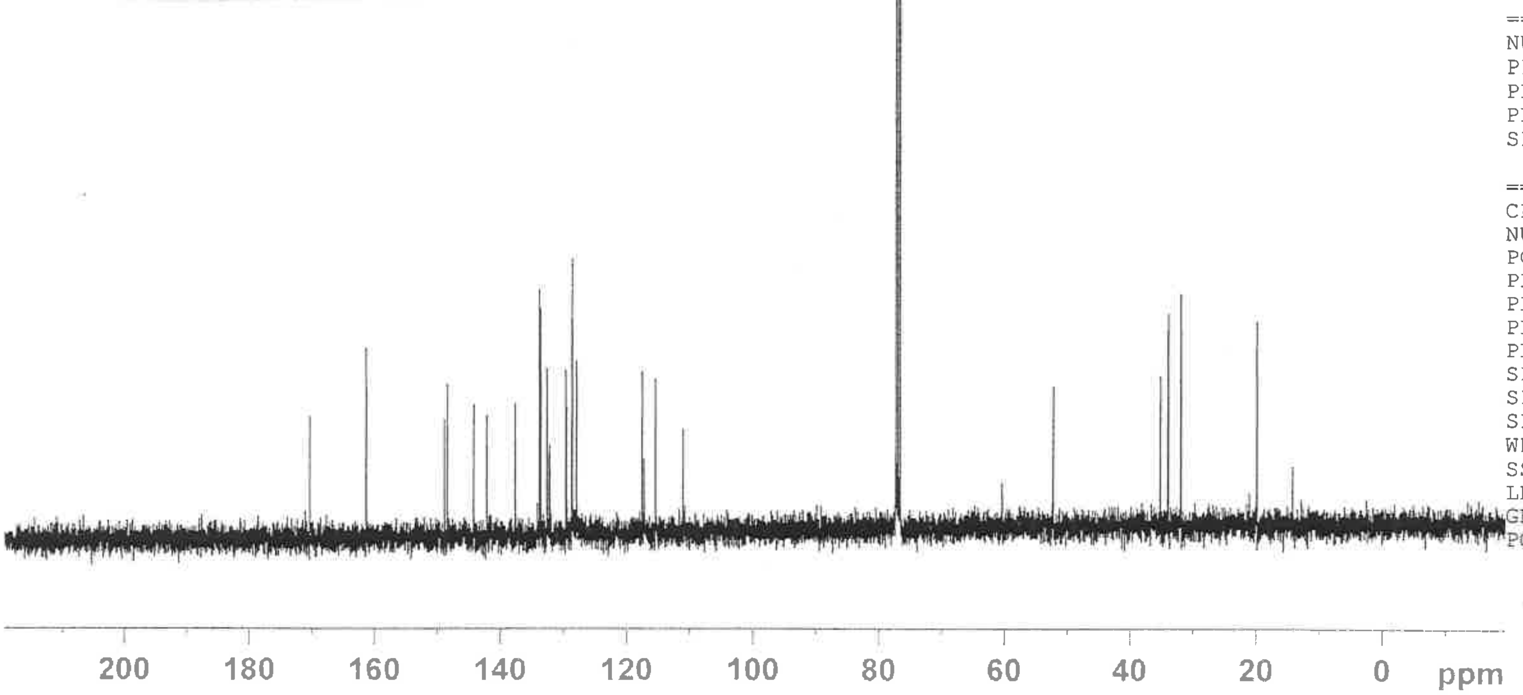

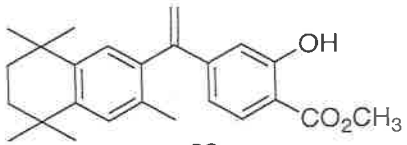

88

BRUKER

NAME	CWVI-071	
EXPNO	2	
PROCNO	1	
Date_	20170222	
Time	14.16	
INSTRUM	spect	
PROBHD	5 mm PABBO BB-	
PULPROG	zgig30	
TD	65536	
SOLVENT	CDCl3	
NS	98	
DS	4	
SWH	24038.461	Hz
FIDRES	0.366798	Hz
AQ	1.3631988	sec
RG	2050	
DW	20.800	usec
DE	6.50	usec
TE	673.2	K
D1	2.00000000	sec
D11	0.03000000	sec
TD0	1	
======== CHANNEL f1 ========		
NUC1	13C	
P1	8.50	usec
PL1	-2.10	dB
PL1W	60.29227829	W
SFO1	100.6228298	MHz
======== CHANNEL f2 ========		
CPDPRG2	waltz16	
NUC2	1H	
PCPD2	90.00	usec
PL2	-1.80	dB
PL12	17.28	dB
PL2W	21.67079544	W
PL12W	0.26783961	W
SFO2	400.1316005	MHz
SI	32768	
SF	100.6127751	MHz
WDW	EM	
SSB	0	
LB	1.00	Hz
GB	0	
PC	1.40	

CWVI-073

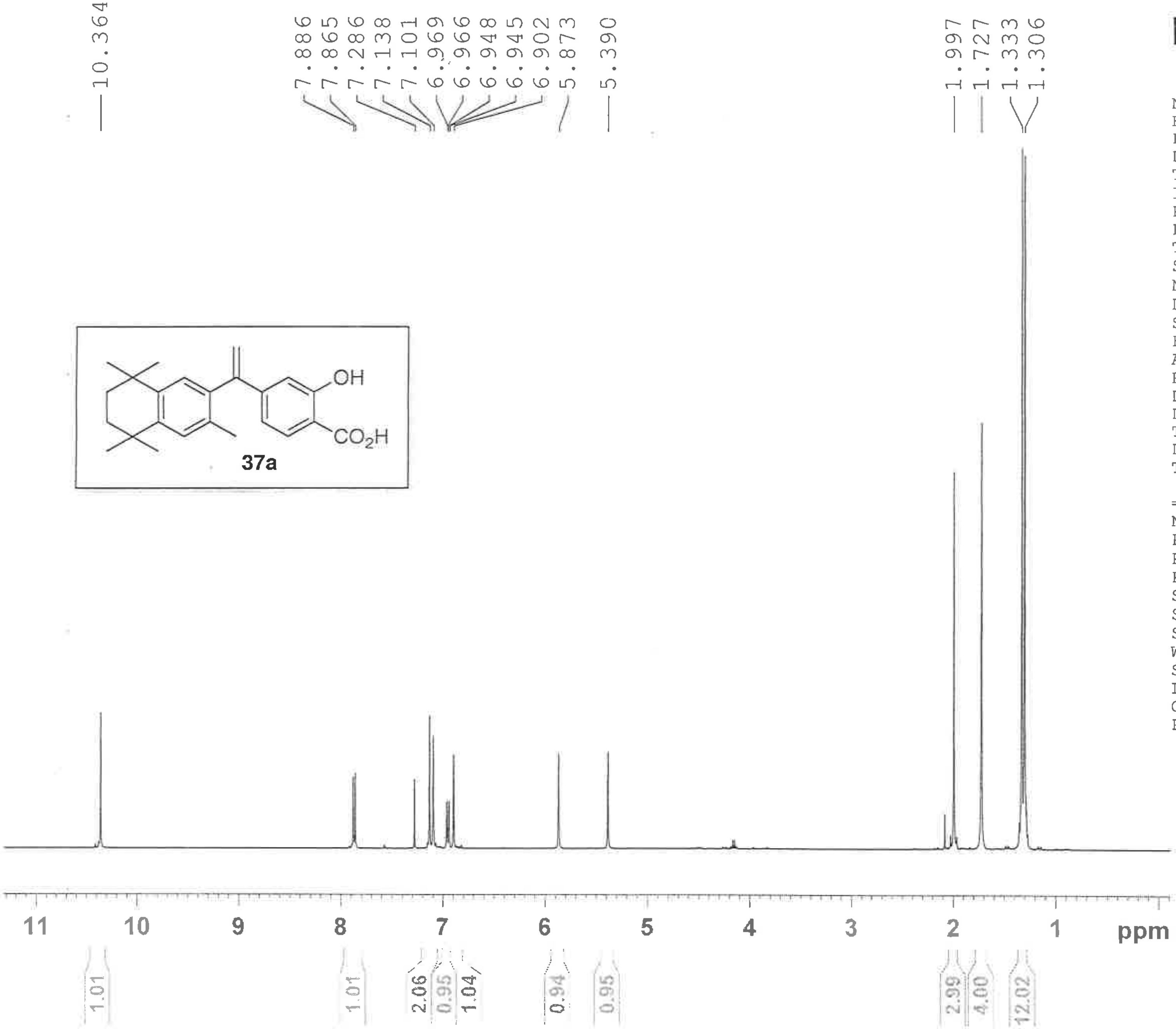

37a

NAME	CWVI-073
EXPNO	3
PROCNO	1
Date_	20170227
Time	14.28
INSTRUM	spect
PROBHD	5 mm PABBO BB-
PULPROG	zg30
TD	65536
SOLVENT	CDCl3
NS	16
DS	2
SWH	8223.685 Hz
FIDRES	0.125483 Hz
AQ	3.9846387 sec
RG	4
DW	60.800 usec
DE	6.50 usec
TE	298.2 K
D1	1.00000000 sec
TD0	1

======== CHANNEL f1 ========

NUC1	1H
P1	14.75 usec
PL1	0.50 dB
PL1W	12.76071072 W
SFO1	400.1324710 MHz
SI	32768
SF	400.1300000 MHz
WDW	EM
SSB	0
LB	0.30 Hz
GB	0
PC	1.00

CWVI-073

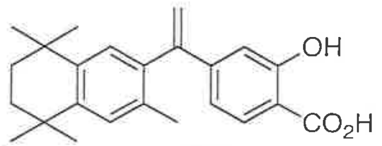

37a

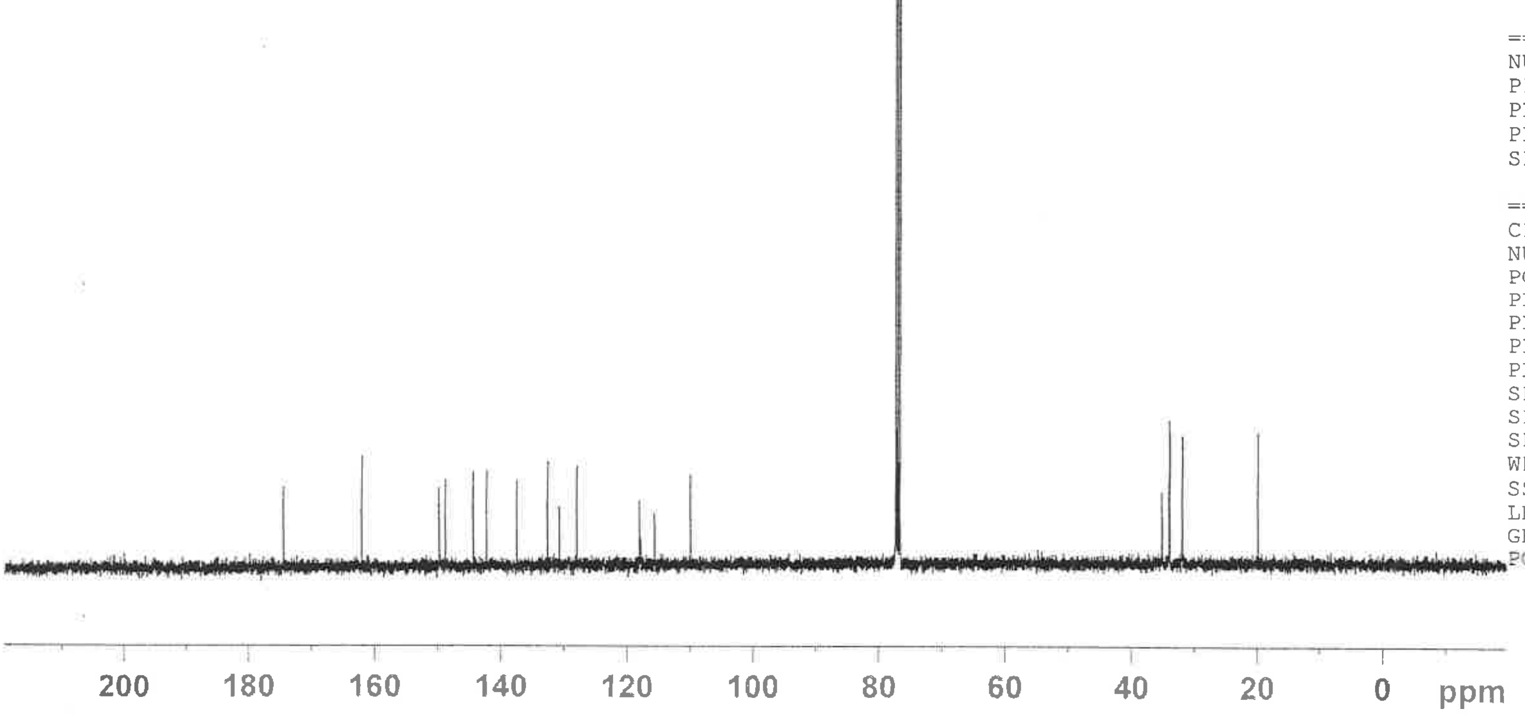

BRUKER

NAME	CWVI-073	
EXPNO	2	
PROCNO	1	
Date_	20170227	
Time	9.58	
INSTRUM	spect	
PROBHD	5 mm PABBO BB-	
PULPROG	zgig30	
TD	65536	
SOLVENT	CDCl3	
NS	500	
DS	4	
SWH	24038.461	Hz
FIDRES	0.366798	Hz
AQ	1.3631988	sec
RG	2050	
DW	20.800	usec
DE	6.50	usec
TE	673.2	K
D1	2.00000000	sec
D11	0.03000000	sec
TD0	1	

======== CHANNEL f1 ========

NUC1	13C	
P1	8.50	usec
PL1	-2.10	dB
PL1W	60.29227829	W
SFO1	100.6228298	MHz

======== CHANNEL f2 ========

CPDPRG2	waltz16	
NUC2	1H	
PCPD2	90.00	usec
PL2	-1.80	dB
PL12	17.28	dB
PL2W	21.67079544	W
PL12W	0.26783961	W
SFO2	400.1316005	MHz
SI	32768	
SF	100.6127729	MHz
WDW	EM	
SSB	0	
LB	1.00	Hz
GB	0	
PC	1.40	

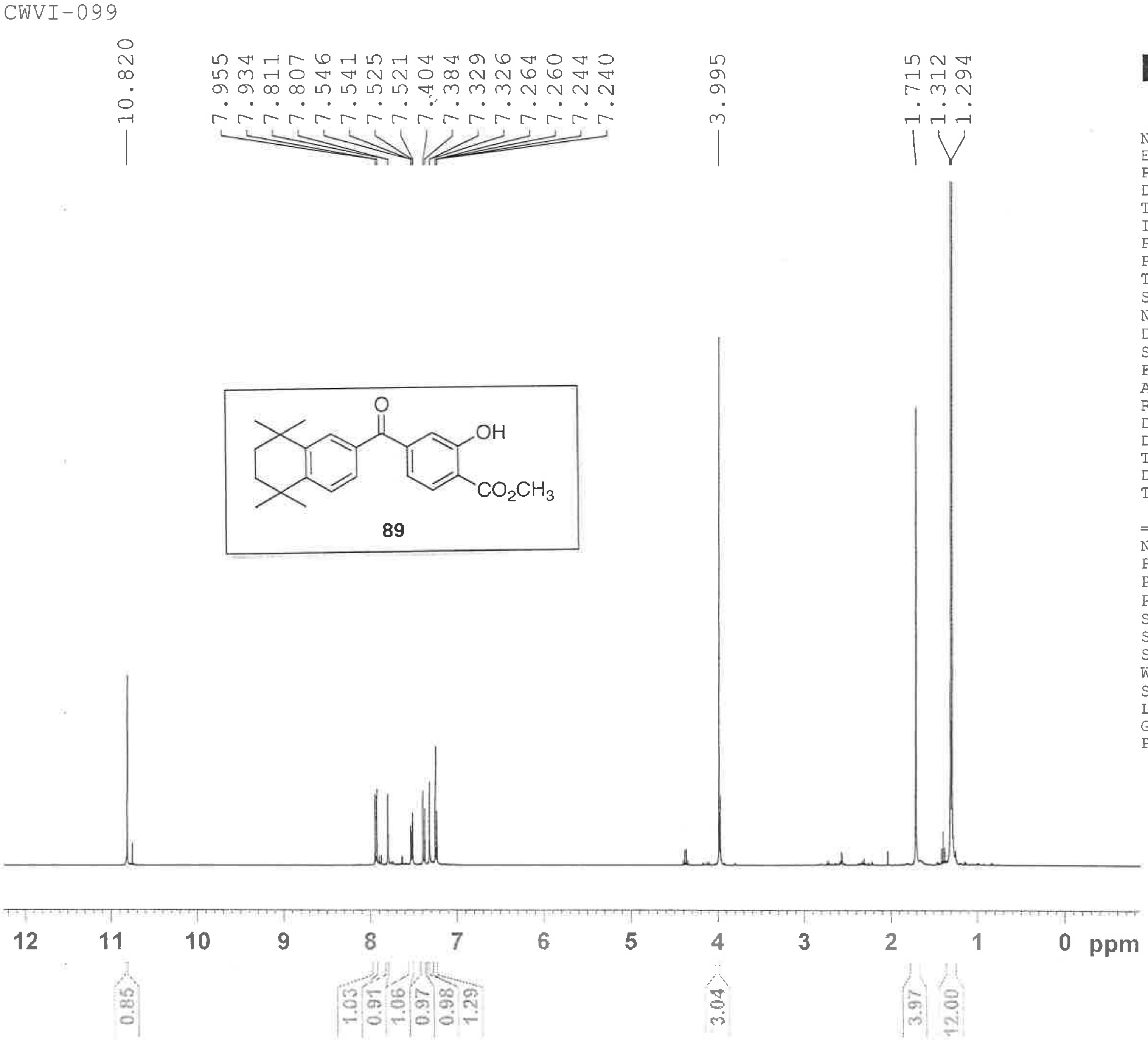

BRUKER

NAME	CWVI-099
EXPNO	1
PROCNO	1
Date_	20180305
Time	10.58
INSTRUM	spect
PROBHD	5 mm PABBO BB-
PULPROG	zg30
TD	65536
SOLVENT	CDCl3
NS	16
DS	2
SWH	8223.685 Hz
FIDRES	0.125483 Hz
AQ	3.9846387 sec
RG	4
DW	60.800 usec
DE	6.50 usec
TE	291.8 K
D1	1.00000000 sec
TD0	1

======== CHANNEL f1 ========

NUC1	1H
P1	14.75 usec
PL1	0.50 dB
PL1W	12.76071072 W
SFO1	400.1324710 MHz
SI	32768
SF	400.1300101 MHz
WDW	EM
SSB	0
LB	0.30 Hz
GB	0
PC	1.00

CWVI-099

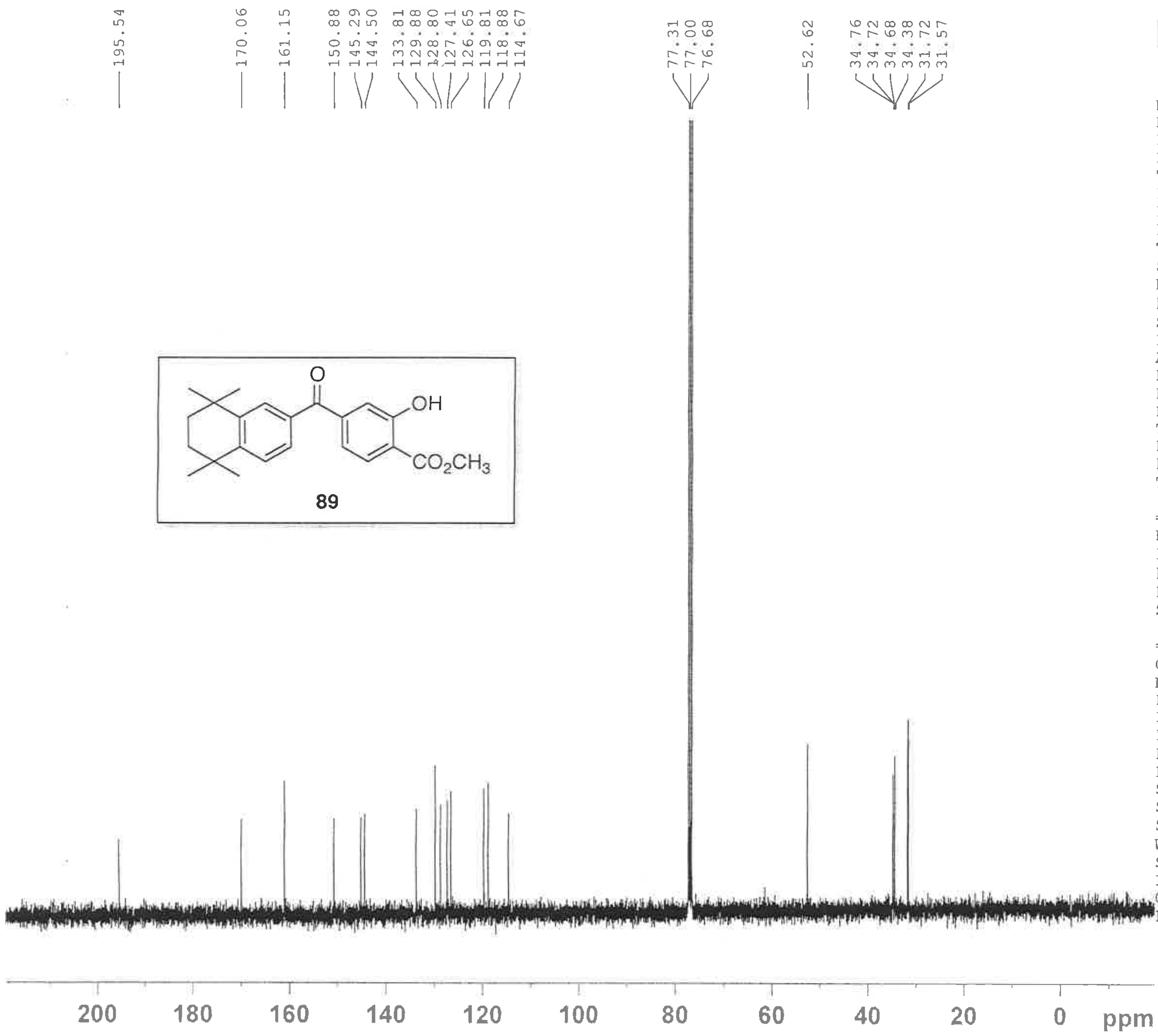

89

BRUKER

NAME	CWVI-099
EXPNO	2
PROCNO	1
Date_	20180305
Time	11.05
INSTRUM	spect
PROBHD	5 mm PABBO BB-
PULPROG	zgig30
TD	65536
SOLVENT	CDCl3
NS	121
DS	4
SWH	24038.461 Hz
FIDRES	0.366798 Hz
AQ	1.3631988 sec
RG	2050
DW	20.800 usec
DE	6.50 usec
TE	292.1 K
D1	2.00000000 sec
D11	0.03000000 sec
TD0	1

======== CHANNEL f1 ========

NUC1	13C
P1	8.50 usec
PL1	-2.10 dB
PL1W	60.29227829 W
SFO1	100.6228298 MHz

======== CHANNEL f2 ========

CPDPRG2	waltz16
NUC2	1H
PCPD2	90.00 usec
PL2	0.50 dB
PL12	16.21 dB
PL2W	12.76071072 W
PL12W	0.34266910 W
SFO2	400.1316005 MHz
SI	32768
SF	100.6127744 MHz
WDW	EM
SSB	0
LB	1.00 Hz
GB	0
PC	1.40

CWVI-101

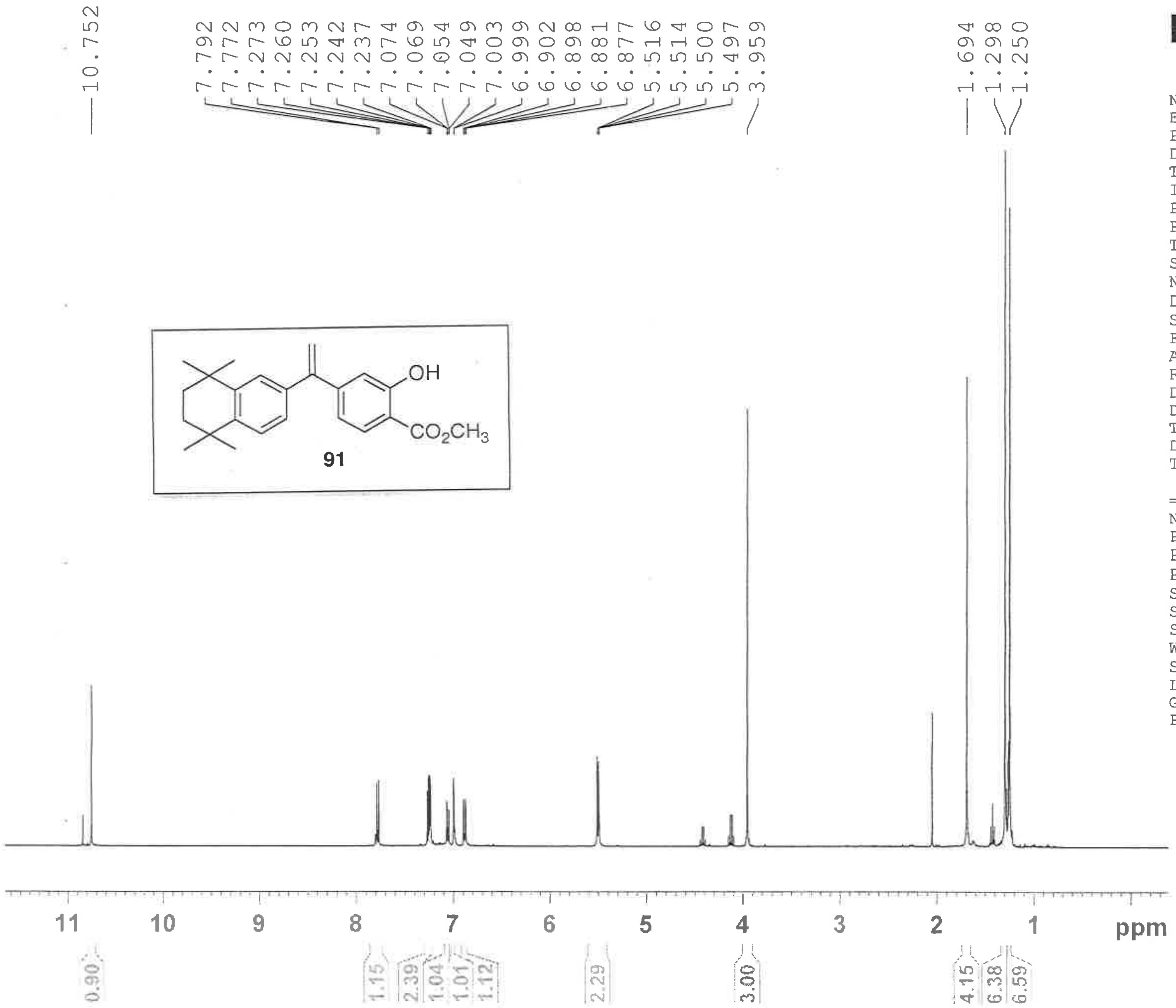

91

BRUKER

NAME	CWVI-101
EXPNO	1
PROCNO	1
Date_	20180305
Time	11.12
INSTRUM	spect
PROBHD	5 mm PABBO BB-
PULPROG	zg30
TD	65536
SOLVENT	CDCl3
NS	16
DS	2
SWH	8223.685 Hz
FIDRES	0.125483 Hz
AQ	3.9846387 sec
RG	4
DW	60.800 usec
DE	6.50 usec
TE	291.9 K
D1	1.00000000 sec
TD0	1

======== CHANNEL f1 ========

NUC1	1H
P1	14.75 usec
PL1	0.50 dB
PL1W	12.76071072 W
SFO1	400.1324710 MHz
SI	32768
SF	400.1300104 MHz
WDW	EM
SSB	0
LB	0.30 Hz
GB	0
PC	1.00

CWVI-101

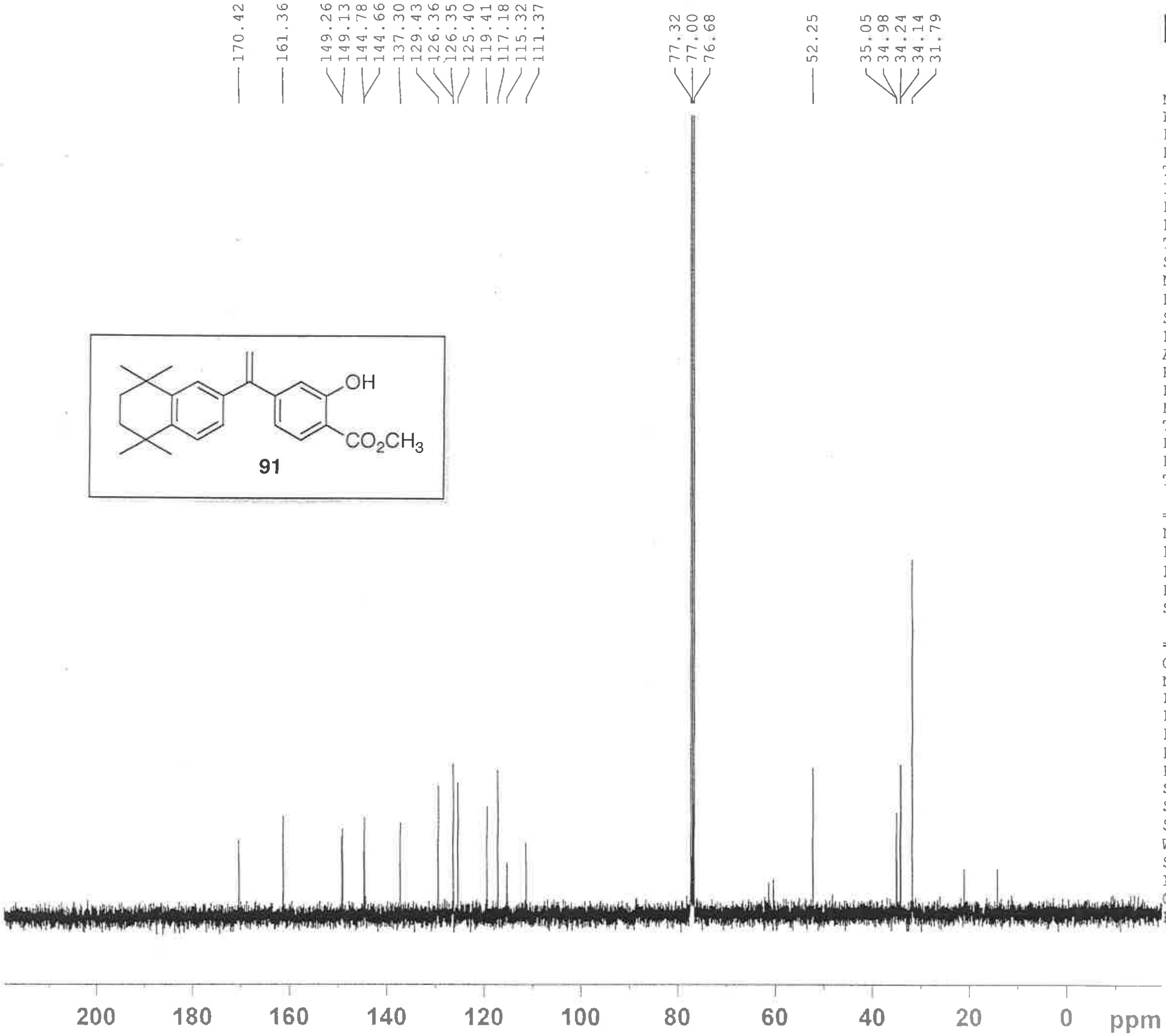

91

BRUKER

NAME	CWVI-101
EXPNO	2
PROCNO	1
Date_	20180305
Time	11.20
INSTRUM	spect
PROBHD	5 mm PABBO BB-
PULPROG	zgig30
TD	65536
SOLVENT	CDCl3
NS	115
DS	4
SWH	24038.461 Hz
FIDRES	0.366798 Hz
AQ	1.3631988 sec
RG	2050
DW	20.800 usec
DE	6.50 usec
TE	292.2 K
D1	2.00000000 sec
D11	0.03000000 sec
TD0	1

======== CHANNEL f1 ========

NUC1	13C
P1	8.50 usec
PL1	-2.10 dB
PL1W	60.29227829 W
SFO1	100.6228298 MHz

======== CHANNEL f2 ========

CPDPRG2	waltz16
NUC2	1H
PCPD2	90.00 usec
PL2	0.50 dB
PL12	16.21 dB
PL2W	12.76071072 W
PL12W	0.34266910 W
SFO2	400.1316005 MHz
SI	32768
SF	100.6127736 MHz
WDW	EM
SSB	0
LB	1.00 Hz
GB	0
PC	1.40

CWVI-103

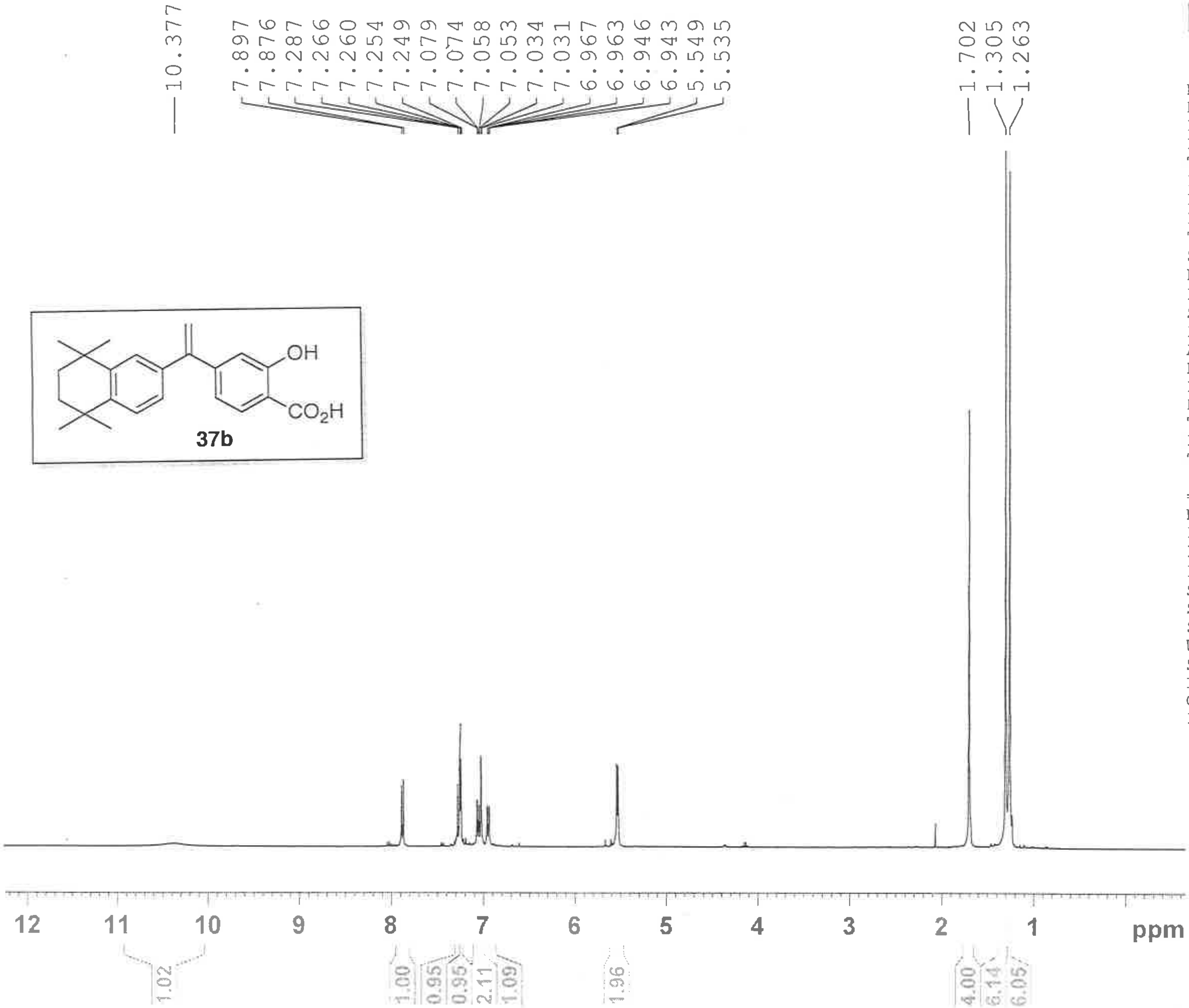

BRUKER

NAME	CWVI-103	
EXPNO	3	
PROCNO	1	
Date_	20181025	
Time	13.57	
INSTRUM	spect	
PROBHD	5 mm PABBO BB-	
PULPROG	zg30	
TD	65536	
SOLVENT	CDCl3	
NS	16	
DS	2	
SWH	8223.685	Hz
FIDRES	0.125483	Hz
AQ	3.9846387	sec
RG	4	
DW	60.800	usec
DE	6.50	usec
TE	292.2	K
D1	1.00000000	sec
TD0	1	

======== CHANNEL f1 ========

NUC1	1H	
P1	14.75	usec
PL1	0.50	dB
PL1W	12.76071072	W
SFO1	400.1324710	MHz
SI	32768	
SF	400.1300099	MHz
WDW	EM	
SSB	0	
LB	0.30	Hz
GB	0	
PC	1.00	

CWVI-103

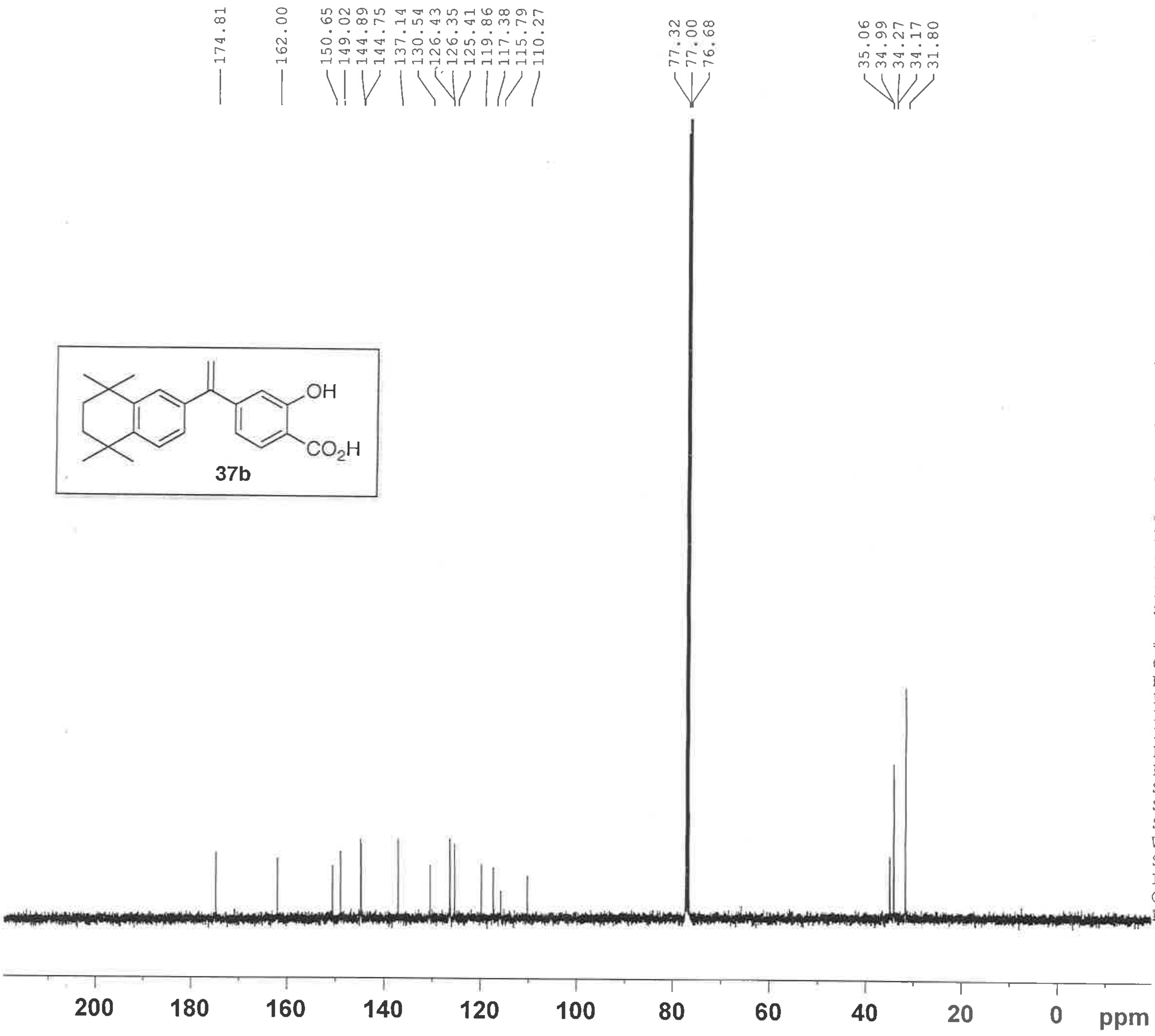

37b

BRUKER

NAME	CWVI-103
EXPNO	4
PROCNO	1
Date_	20181025
Time	14.03
INSTRUM	spect
PROBHD	5 mm PABBO BB-
PULPROG	zgig30
TD	65536
SOLVENT	CDCl3
NS	541
DS	4
SWH	24038.461 Hz
FIDRES	0.366798 Hz
AQ	1.3631988 sec
RG	2050
DW	20.800 usec
DE	6.50 usec
TE	292.5 K
D1	2.00000000 sec
D11	0.03000000 sec
TD0	1
======== CHANNEL f1 ========	
NUC1	13C
P1	8.25 usec
PL1	-2.10 dB
PL1W	60.29227829 W
SFO1	100.6228298 MHz
======== CHANNEL f2 ========	
CPDPRG2	waltz16
NUC2	1H
PCPD2	90.00 usec
PL2	0.50 dB
PL12	16.21 dB
PL2W	12.76071072 W
PL12W	0.34266910 W
SFO2	400.1316005 MHz
SI	32768
SF	100.6127729 MHz
WDW	EM
SSB	0
LB	1.00 Hz
GB	0
PC	1.40

```
Acquisition Experiment Report
File: c:\masslynx\july 21.pro\data\210826_cw-vi-003.raw

Header
Acquired File Name:   210826_CW-VI-003
Acquired Date:        26-Aug-2021
Acquired Time:        09:54:58
Job Code:             July21
Task Code:
User Name:
Laboratory Name:
Instrument:           ACQ-QDA#KAD3226
Conditions:
Submitter:
SampleID:
Bottle Number:        2:1
Description:

Instrument Calibration:
Calibration File: C:\MassLynx\IntelliStart.pro\AcquDB\AutoCal.cal
Parameters
MS1 Static:
Mass:               30 Da to 1250 Da.
Resolution:         -0.1/-0.0
Ion Energy:         -0.3
Reference File:     Internal
Acquisition File:
MS1 Scanning:
Mass:               30 Da to 1250 Da.
Resolution:         -0.1/-0.0
Ion Energy:         -0.3
Reference File:     Internal
Acquisition File:
MS1 Scan Speed Compensation:
Scan:               40 to 10000 amu/sec.
Resolution:         -0.1/-0.0
Ion Energy:         -0.3
Reference File:     Internal
Acquisition File:
Calibration Time: 10:54
Calibration Date: 09/13/17
Coefficients
MS1 Static:         -0.000000000000*x^4 + 0.000000000656*x^3 +
-0.000001077025*x^2 + 1.000599988917*x +-0.085640798905
Function 1:         -0.000000000000*x^4 + 0.000000000433*x^3 +
-0.000000811760*x^2 + 1.000482155999*x +-0.071926097790
Function 2:         None


Parameters for C:\MassLynx\July
```

21.PRO\ACQUDB\NEG_100-500_C_2Hz_5min.EXP

Prescan Statistics:

Initial Average Intensity	379.7862	
Initial Average Std Dev		1.1247
Bunch Zero Level	0.0000	
Bunch Std Dev		0.0000
Bunch Threshold		0.0000
Spike Removal Std Dev		1.1245
Ion Count Threshold:		25.0000

Data Processing:

Targeted Sampling Frequency		2
Actual Sampling Frequency	2.000	
SIR Chromatogram Spike Removal		ON
SIR Smoothing		ON
Smoothing window size (scans)		3
Number of smooths		2

Method Events:

Initial Stop Flow:	No Change	
Initial Switch 1:	No Change	
Timed Events Enabled		

Event	Time(Sec)	Name	Action

Instrument Parameters - Function 1:

Polarity	ES-	
Calibration		Dynamic 2
Capillary (kV)		0.80
Cone (V)		5.00
Source Temperature (∞C)		120
Probe Temperature (∞C)		350
Calibration Temperature (∞C)		0

Engineers Settings:

LM 1 Resolution	-0.12
HM 1 Resolution	-0.02
Low Mass Ion Energy 1	-0.26
High Mass Ion Energy 1	-0.12
Low Mass Position	0.69
High Mass Position	-1.81
Low Mass Setup	0.63
High Mass Setup	-6.13
Detector Gain Positive	1.0
Detector Gain Negative	1.0
Nominal Rod Polarity	Negative

Dynamic Offset Positive Settings:

Dynamic Offset Low Mass Resolution	0.00
Dynamic Offset High Mass Resolution	0.00
Dynamic Offset Low Mass Ion Energy	0.00
Dynamic Offset High Mass Ion Energy	-0.02

Dynamic Offset Negative Settings:

Dynamic Offset Low Mass Resolution	-0.01
Dynamic Offset High Mass Resolution	0.21
Dynamic Offset Low Mass Ion Energy	0.00
Dynamic Offset High Mass Ion Energy	0.19

Dynamic Offset Settings:

Dynamic Offset Low Mass Position	-0.71
Dynamic Offset High Mass Position	-0.46
Dynamic Offset Low Mass Setup	0.55
Dynamic Offset High Mass Setup	-0.04
Dynamic Offset Linearity Gain	4.80

Set Detector Gain 1.0

Instrument Readbacks

Capillary (kV)		0.80
Cone (V)		-9.32
Source Temperature (∞C)		120
Multiplier	355	
Probe Temperature (∞C)		350
Calibration Temperature (∞C)		28

Inter-scan delays:

Automatic Mode

MS Delay Table

	R	delay
<=	16.000	0.005
>	16.000	0.006

Health Check Failures:

All Health Checks Passed

Health Checks all enabled unless shown below:

Resolution Setup Required

Recalibration Required

Service Due

ACE Experimental Record

Inlet Method File: c:\masslynx\july 21.pro\acqudb\col4_pfp_fa_acn_5mins

--------------------- Run method parameters ----------------

-- PUMP --

Waters ACQUITY QSM
Solvent A Name: Water
Solvent B Name: Methanol
Solvent C Name: 0.1% FA
Solvent D Name: Acetonitrile
Low Pressure Limit: 0 psi
High Pressure Limit: 15000 psi
Seal Wash Period: 5.00 min
[Gradient Table]
Time(min) Flow Rate(mL/min) %A %B %C %D Curve
1. Initial 0.500 0.0 0.0 95.0 5.0 Initial
2. 2.00 0.500 0.0 0.0 5.0 95.0 6
3. 3.50 0.500 0.0 0.0 95.0 5.0 11
4. 5.00 0.500 0.0 0.0 95.0 5.0 11
Comment:
Flow Ramp Rate: 0.45 min
D Solvent Selection (if supported): No Change
System Pressure Data Channel: No
Flow Rate Data Channel: No
%A Data Channel: No
%B Data Channel: No
%C Data Channel: No
%D Data Channel: No
Primary Data Channel: No
Accumulator Data Channel: No
Degasser Data Channel: No
Gradient Start: At Injection
Gradient Start Volume: 0 uL
Gradient Start Time: 0.00 min
Participate in pre-analysis: No

-- END PUMP --

-- DETECTOR --

Waters Acquity CM

Target Column Temperature: 50.0 C
Temperature Alarm Band: 5.0 C
: No

Column Valve Position: Column 4
Equilibration Time: 0.1 min
External Valve 1: No Change
External Valve 2: No Change
External Valve 3: No Change
Comment:
Column Temperature Data Channel: No
Preheater Temperature Data Channel: No

-- END DETECTOR --

-- DETECTOR --

Waters Acquity PDA

Run Time: 5.00 min
PDA Detector Type: UPLC LG 500 nm
Lamp: On
Sampling Rate: 20 points/sec
Filter Time Constant: 0.2000 sec
Exposure Time: Auto msec
Interpolate 2nd order filter Region: No
Use UV Blocking Filter: Yes
3D Channel...
Range: 210 - 499
Resolution: 2.4 nm
Channel 1...
Data Mode: Absorbance at 214
Resolution: 2.4 nm
Channel 2...
Data Mode: Absorbance at 380
Resolution: 2.4 nm
Initial Switch 1: No Change
Initial Switch 2: No Change

-- END DETECTOR --

-- AUTOSAMPLER --

Waters ACQUITY FTN AutoSampler
Run Time: 5.00 min
Comment:
Load Ahead: Disabled
Loop Offline: Automatic min
Wash Solvent Name: Weak Wash
Pre-Inject Wash Time: 0.0 sec
Post-Inject Wash Time: 6.0 sec
Purge Solvent Name: Strong Wash

Maximum System Pressure: 5734.0 psi

Average System Pressure: 4515.0 psi

----------------------------- oOo -----------------------------

------------------- Waters ACQUITY FTN Postrun Report ---------------

Software Version: 1.60.1774

Firmware Version: 1.60.364 (Sep 20 2013)

Checksum: 0x35d5392b

Serial Number: D17SDI835G

Sample Syringe Size: 100.0

Extension Loop Size: 0.0

Needle Size: 30.0

Minimum Sample Temperature: 10.0

Maximum Sample Temperature: 10.2

Average Sample Temperature: 10.1

Minimum Column Temperature: -0.2

Maximum Column Temperature: 0.0

Average Column Temperature: -0.2

----------------------------- oOo -----------------------------

------------------- Waters Acquity CM Postrun Report ---------------

Software Version: 1.60.2072

Firmware Version: 1.65.142 (Apr 02 2015)

Checksum: 0x18bfb4ea

Serial Number: D17CMP604G

Valve Position: 4

Minimum Column Temperature: 50.0

Maximum Column Temperature: 50.0

Average Column Temperature: 50.0

------------------------------ oOo ------------------------------

------------------- Active eCord Data ---------------

Valve Current Position: 4
Failed to retrive Active eCord ColumnData Data

------------------------------ oOo ------------------------------

------------------- Generic Instrument Postrun Report ---------------

Software Version: 1.60.1390

Firmware Version: 1.60.6169 (Aug 13 2013)

Checksum: 0xdcfe9340

Serial Number: B17UPD132A

Lamp On/Off Event: No

Lamp Life: 3223.00 hours

Lamp Serial Number: 000133684

Exposure Time: 50.000 msec

Lambda1: 187.360

Lambda512: 502.647

Flow Cell Type: Analytical LG

Flow Cell Path Length: 10.000 mm

Flow Cell Volume: 0.500 microliters

Flow Cell Serial Number: PDA10-15625

Flow Cell Part Number: 205015017

Optics Temperature Stabilzation Setting: Normal Temperature

---------------------------- oOo ----------------------------

---------------Failed to get IECordHost2 Interface ---------

Function 1	
Scans in function:	601
Cycle time (secs):	Automatic
Scan duration (secs):	0.495
Inter Scan Delay (secs):	Automatic
Start and End Time(mins):	0.000 to 5.000
Ionization mode:	ES-
Data type:	Accurate Mass
Function type:	Scan
Mass range:	100 to 500

Function 2	
Scans in function:	6001
Function type:	Diode Array
Wavelength range (nm):	210 to 499

mz not observed; see 426

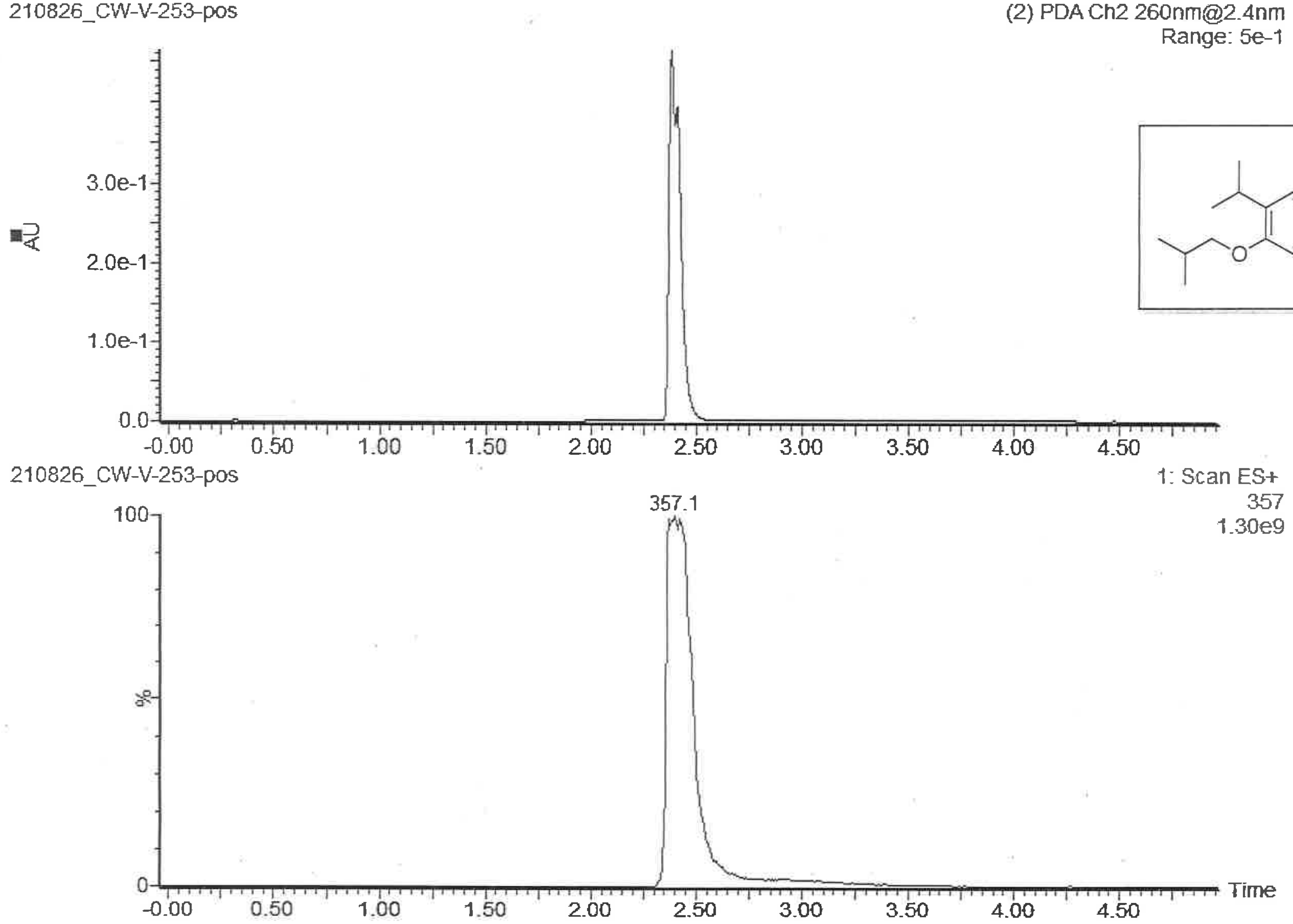

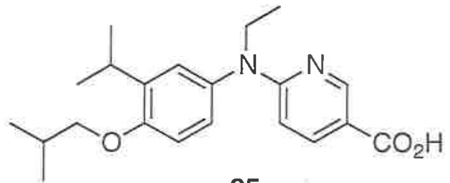

25

mz not observed

210826_CW-VI-035_pos

(2) PDA Ch2 260nm@2.4nm
Range: 7e-1

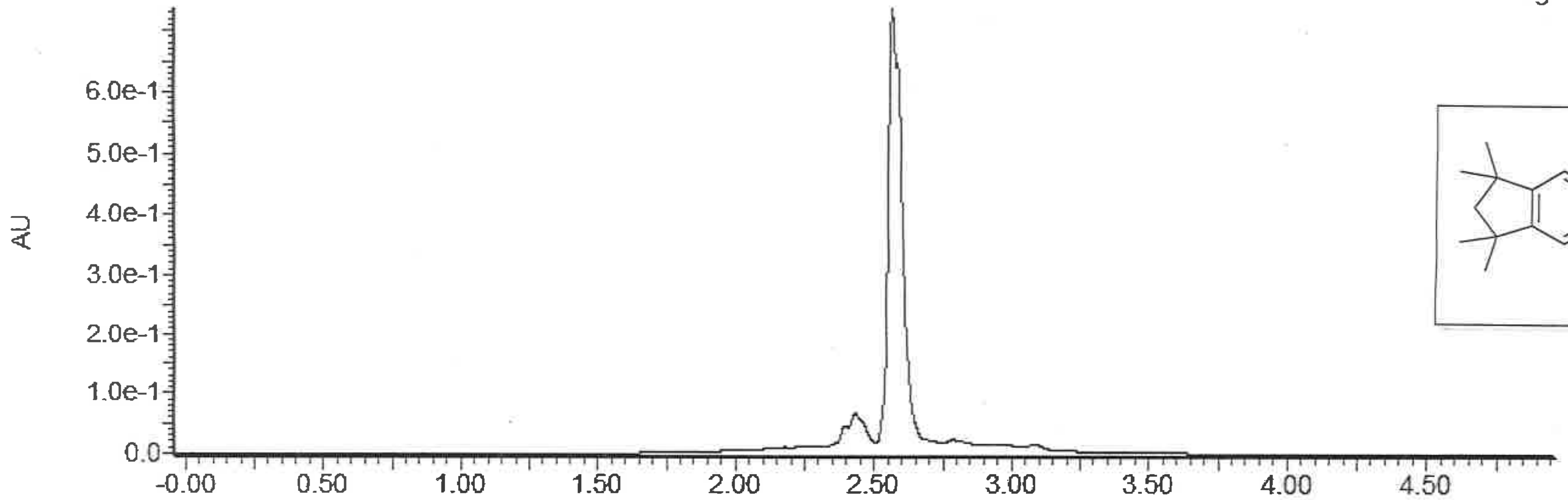

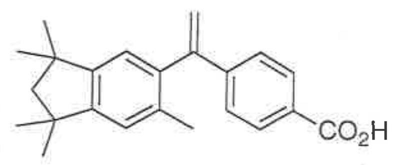

26

210826_CW-VI-035_pos

1: Scan ES+
335
6.96e7

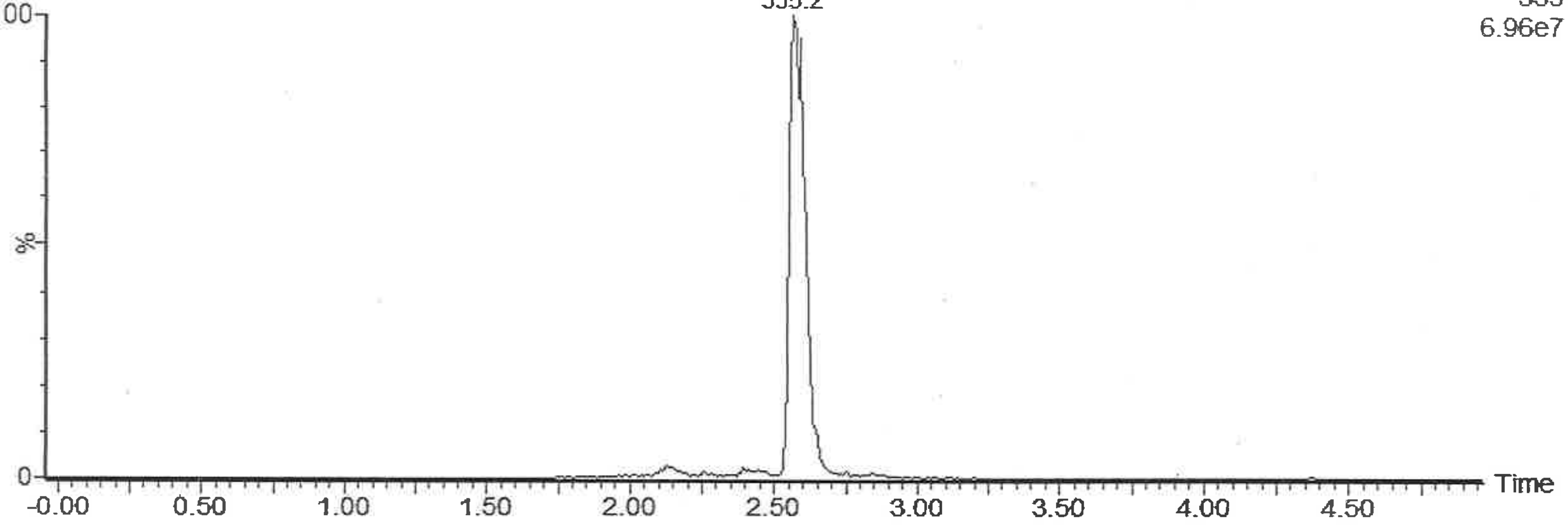

mz not observed

210826_CW-VI-025_pos

(2) PDA Ch2 260nm@2.4nm
Range: 8e-1

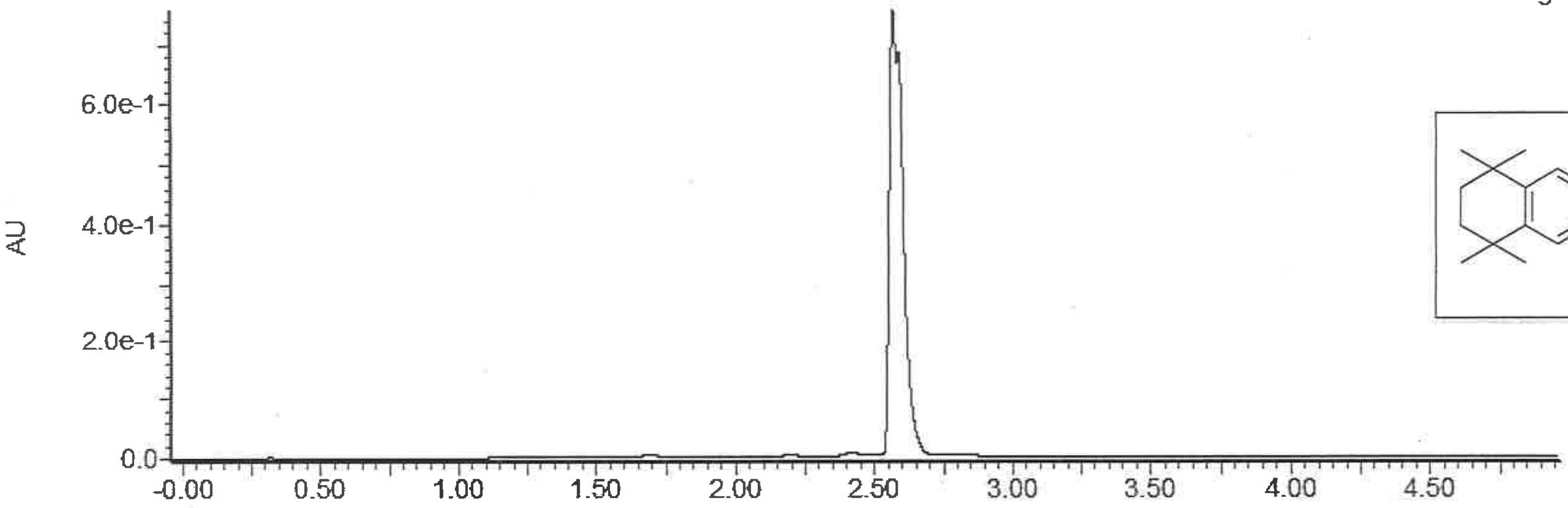

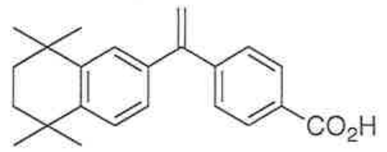

27

210826_CW-VI-025_pos

1: Scan ES+
335
3.15e8

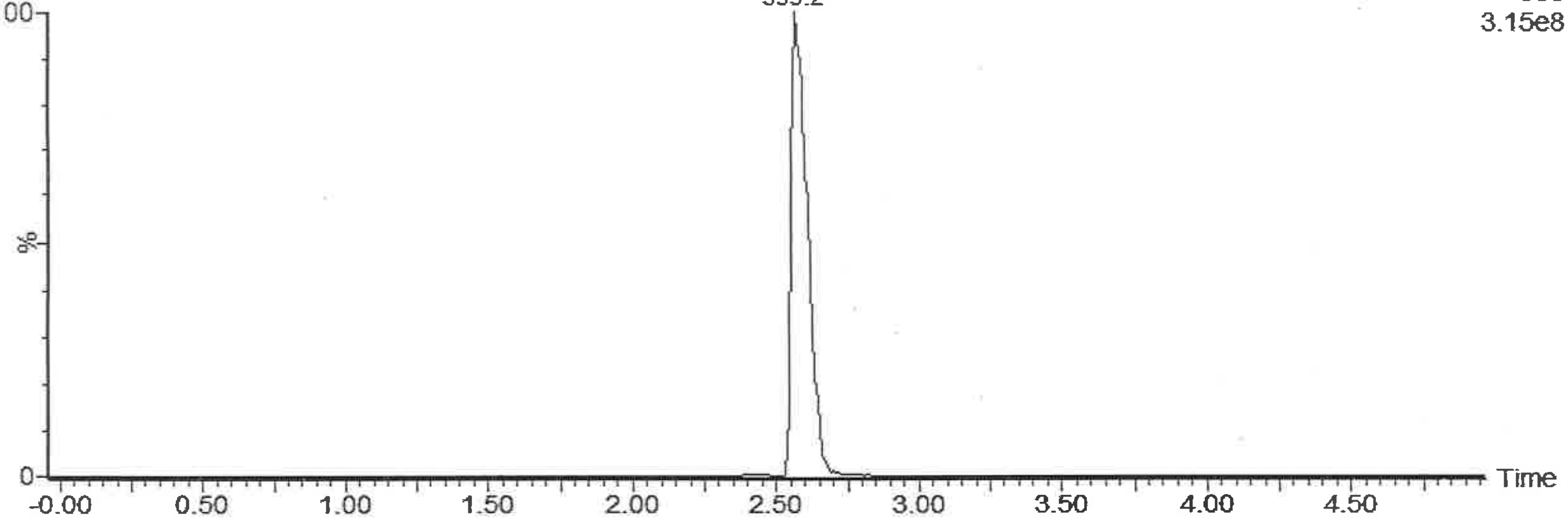

Label - CW-V-259; weak; see 402

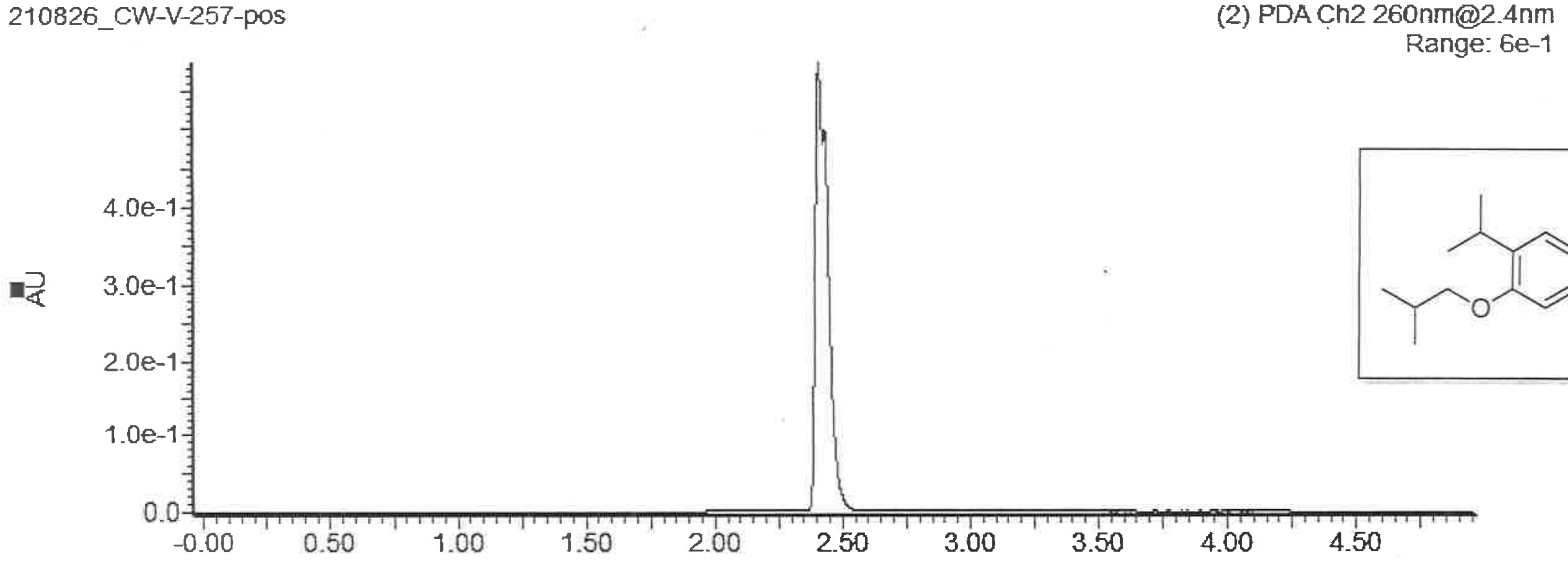

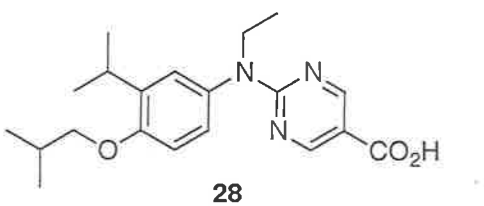

28

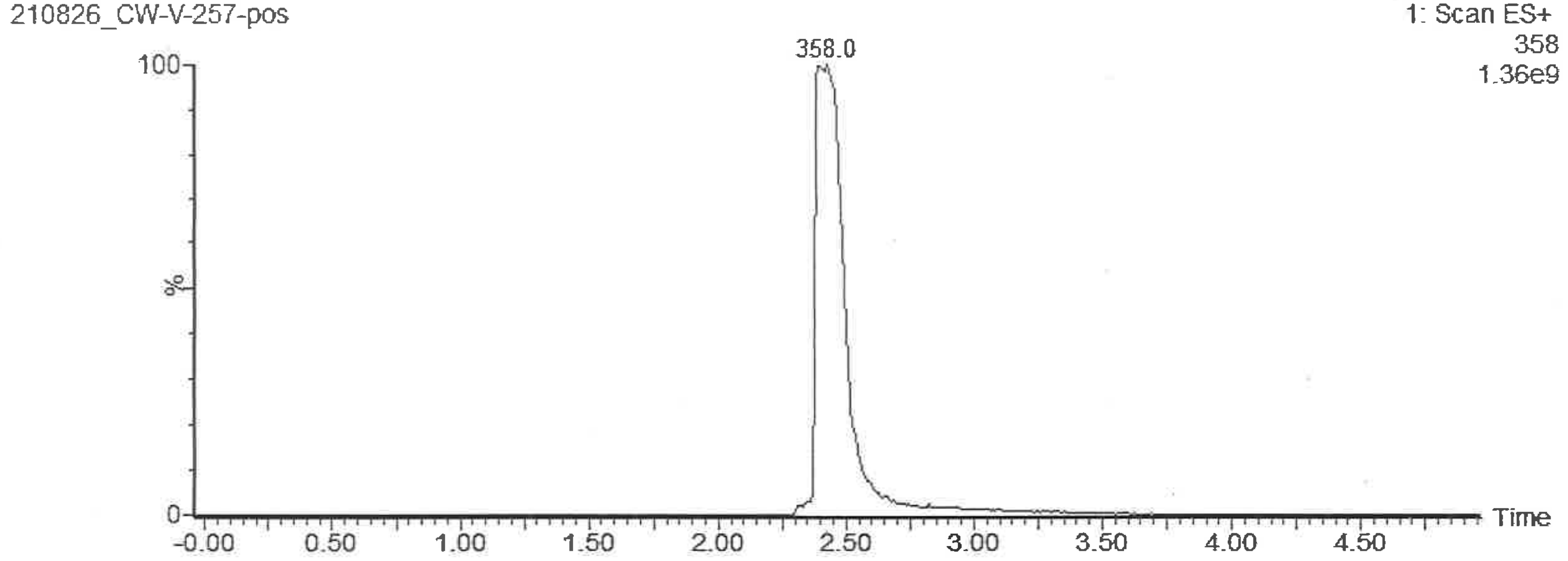

mz not observed

210826_CW-V-265-pos

(2) PDA Ch2 260nm@2.4nm
Range: 2e-1

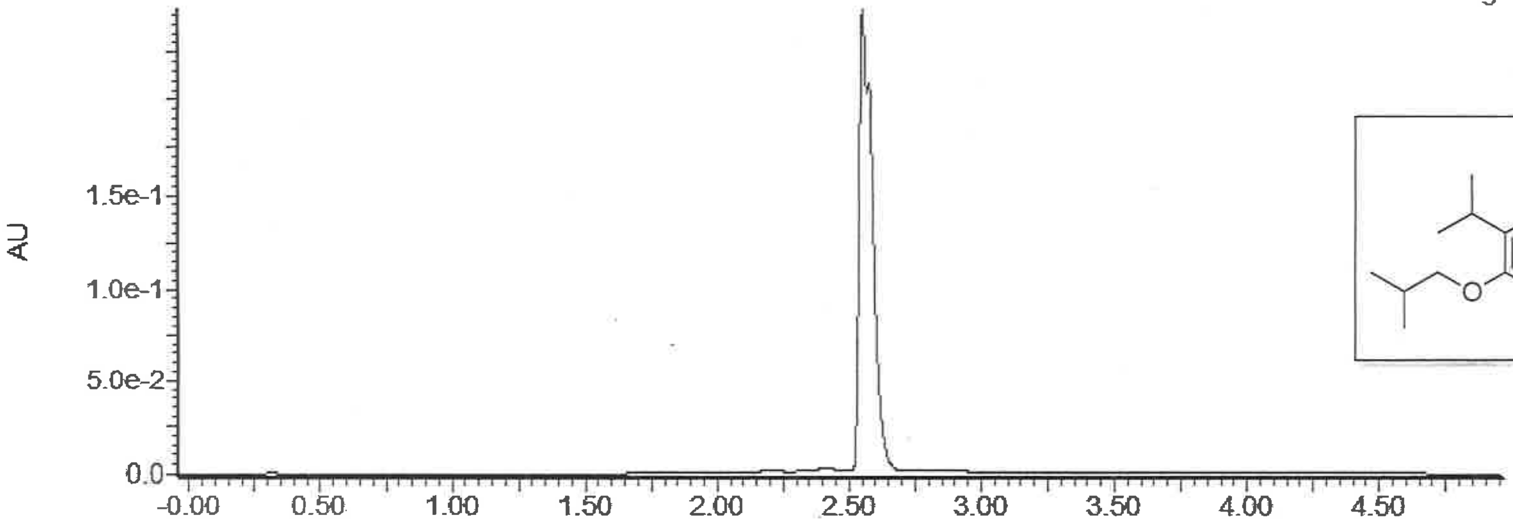

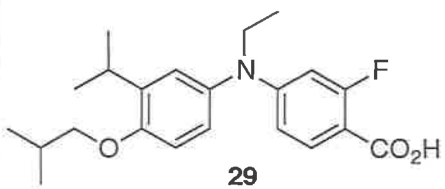

29

210826_CW-V-265-pos

1: Scan ES+
374
1.38e9

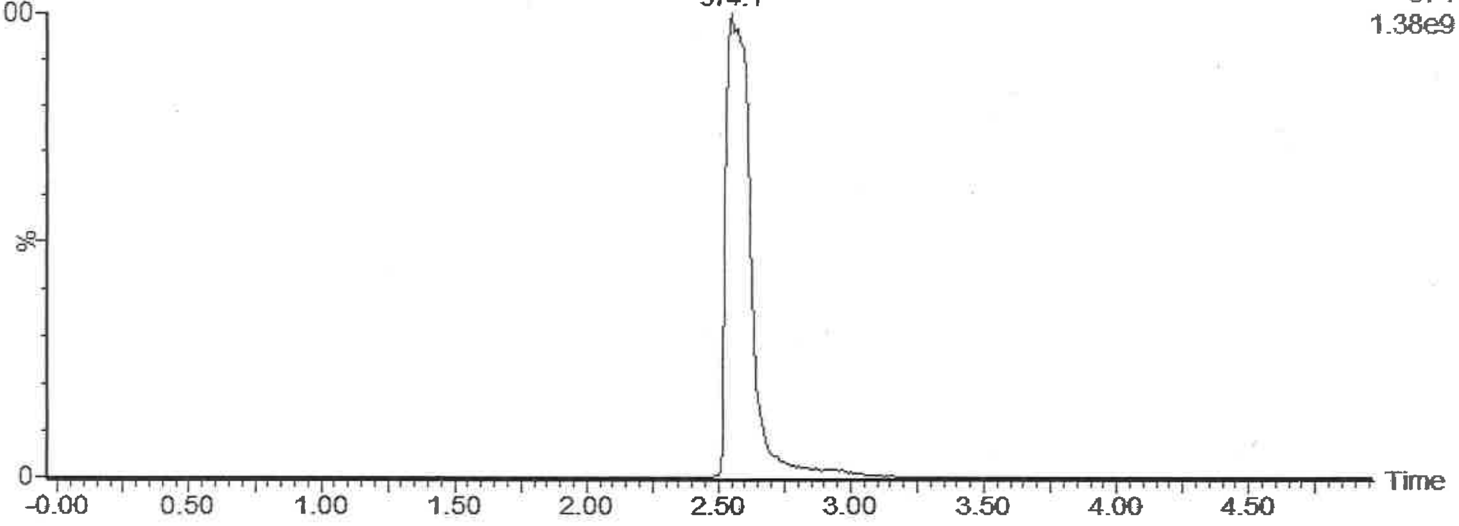

mz not observed

210826_CW-V-263-pos

(2) PDA Ch2 260nm@2.4nm
Range: 2e-1

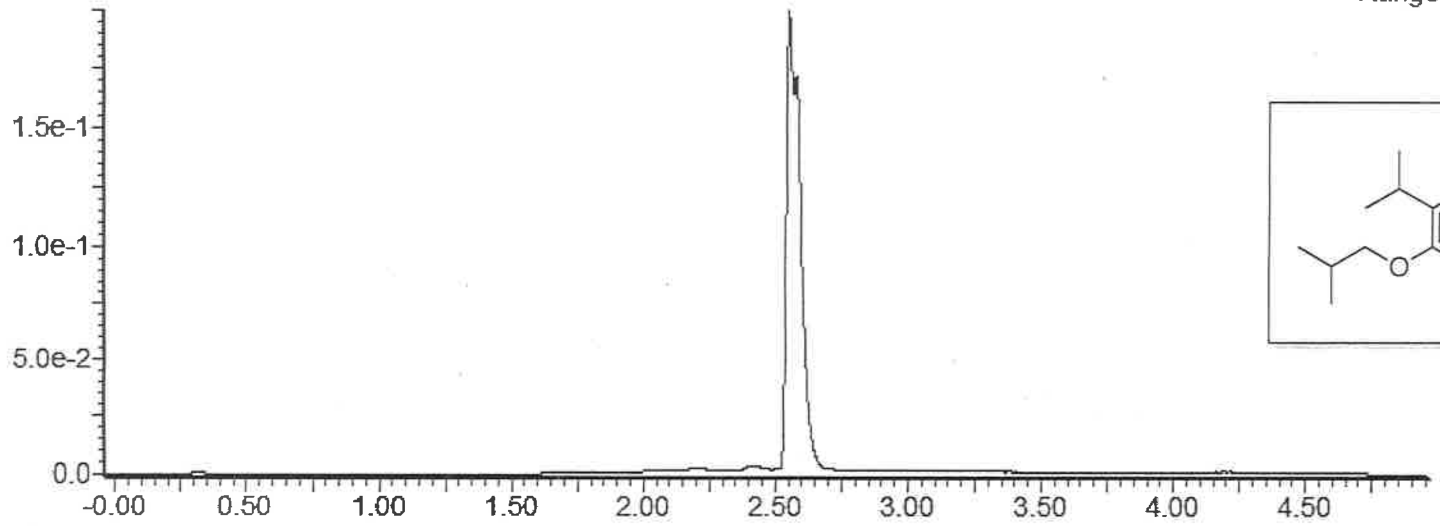

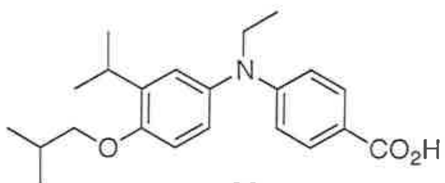

30

210826_CW-V-263-pos

1: Scan ES+
356
1.31e9

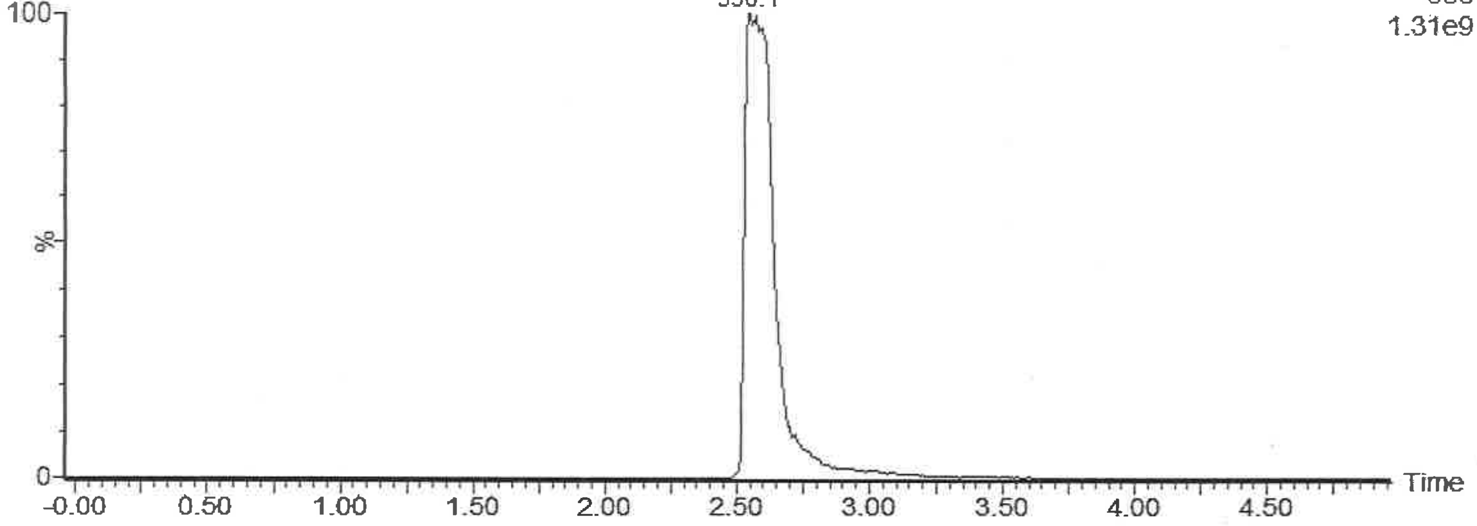

mz weak; see 398

210826_CW-V-267-pos

(2) PDA Ch2 260nm@2.4nm
Range: 9e-1

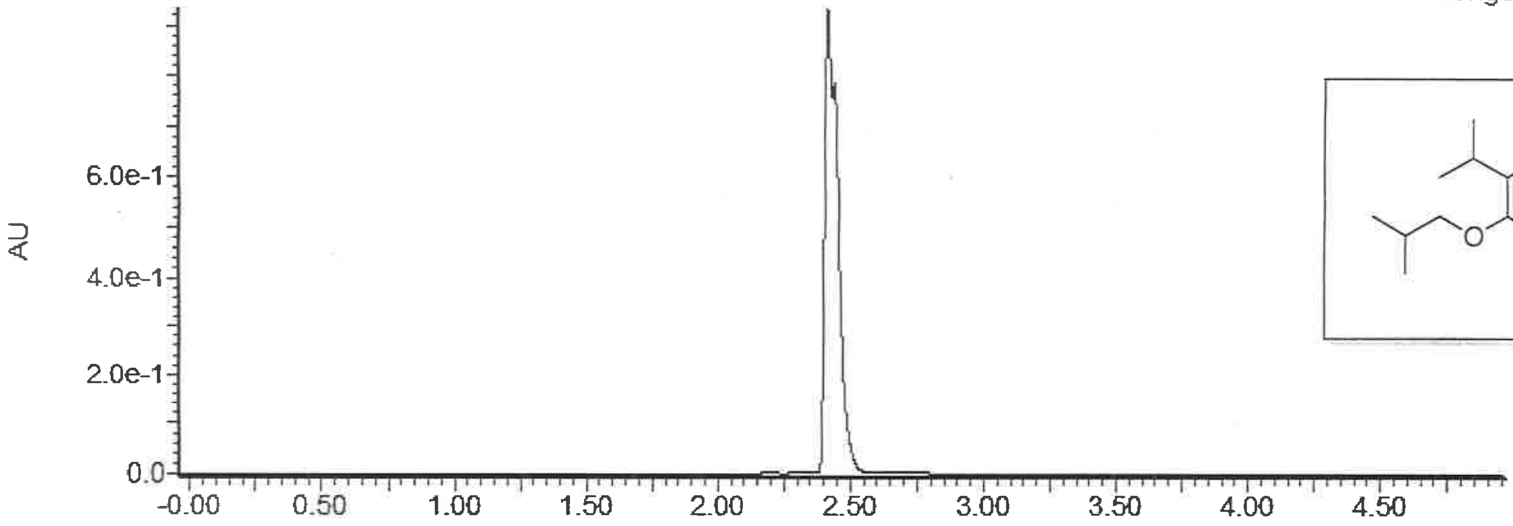

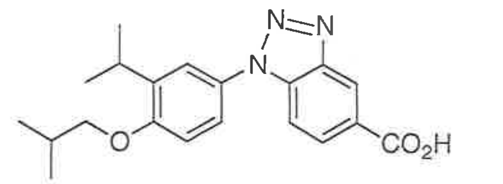

31

210826_CW-V-267-pos

1: Scan ES+
354
1.33e9

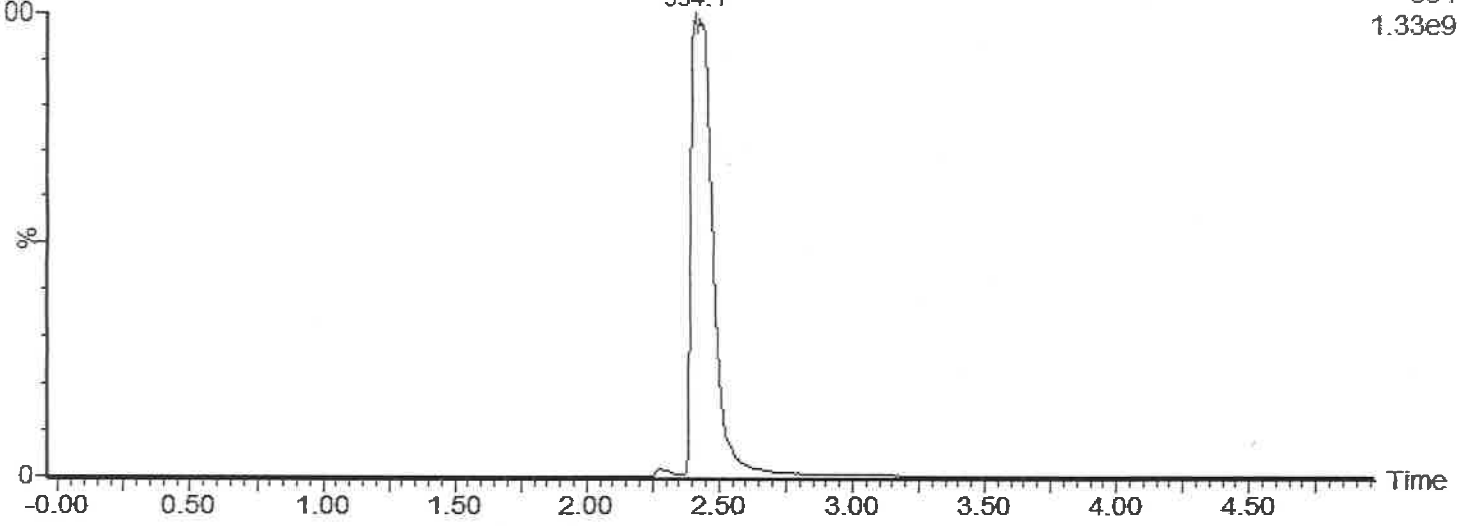

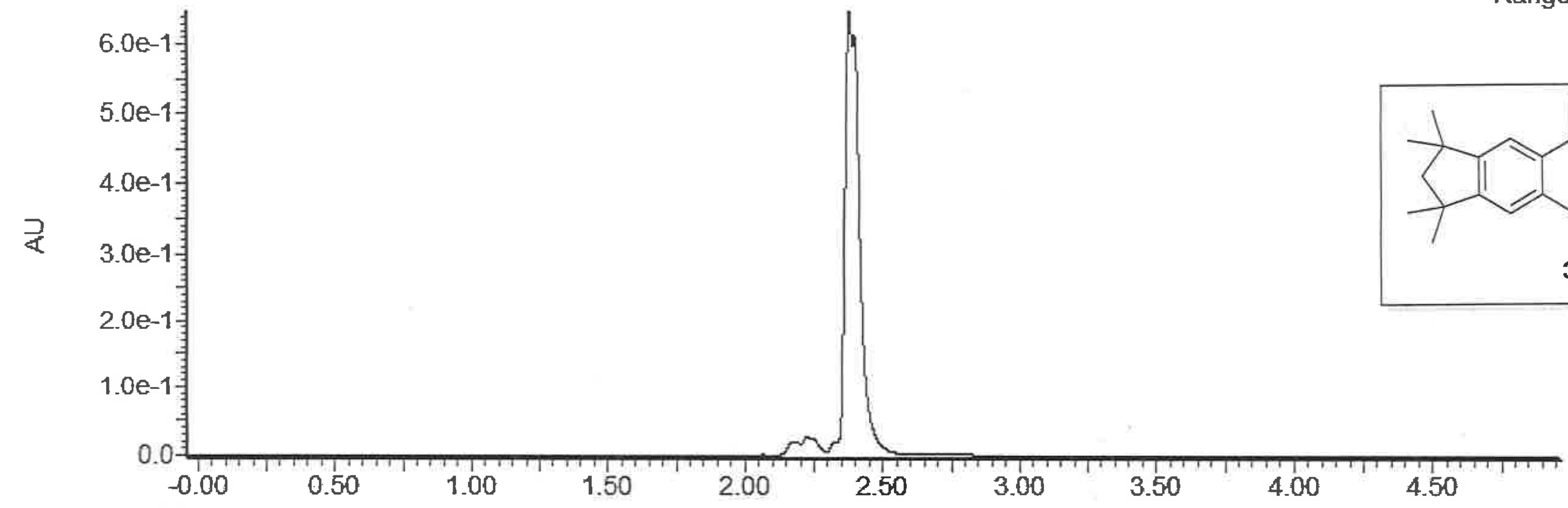
210826_CW-VI-007
(2) PDA Ch2 260nm@2.4nm
Range: 7e-1
AU
6.0e-1
5.0e-1
4.0e-1
3.0e-1
2.0e-1
1.0e-1
0.0
-0.00
0.50
1.00
1.50
2.00
2.50
3.00
3.50
4.00
4.50

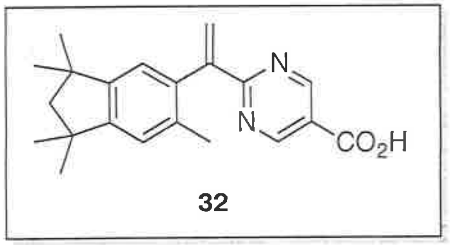
N
N
CO_2H

32

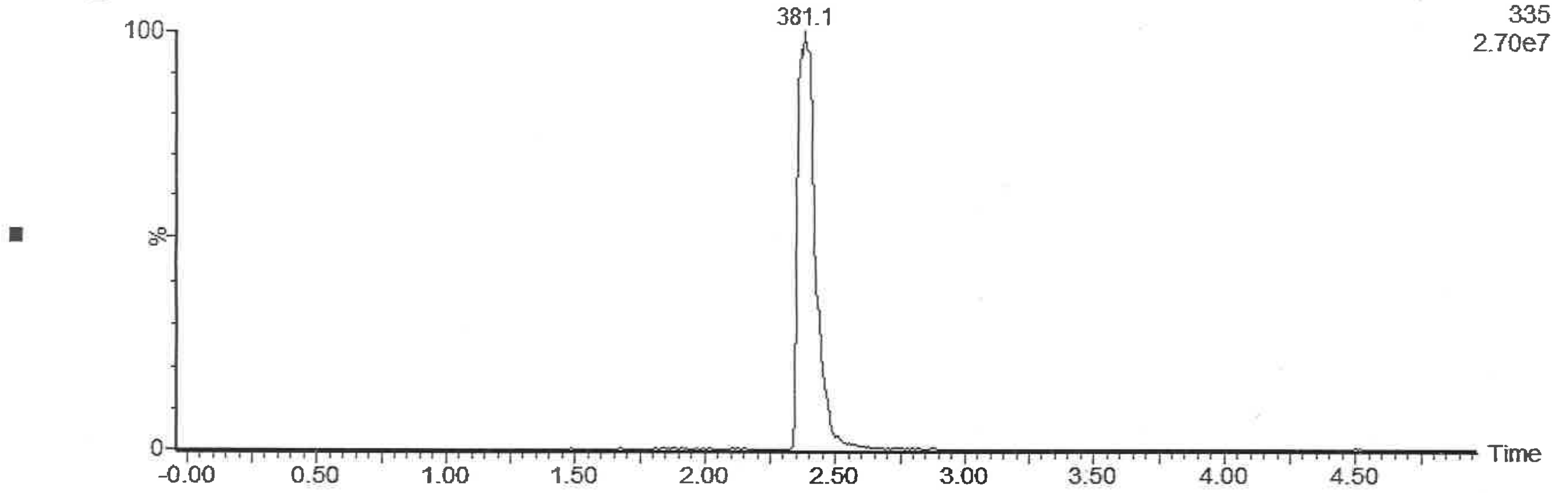
210826_CW-VI-007
1: Scan ES-
335
2.70e7
381.1
100
%
0
-0.00
0.50
1.00
1.50
2.00
2.50
3.00
3.50
4.00
4.50
Time

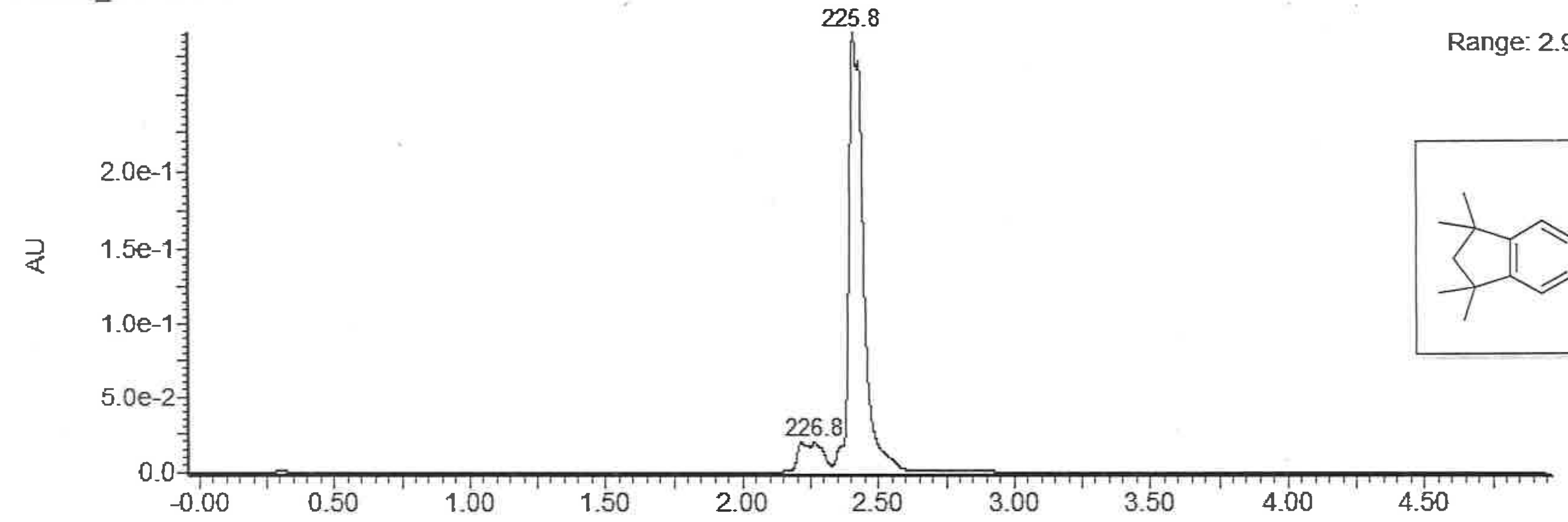
210826_CW-VI-003
2: Diode Array
260
Range: 2.93e-1
AU
225.8
226.8
2.0e-1
1.5e-1
1.0e-1
5.0e-2
0.0
-0.00
0.50
1.00
1.50
2.00
2.50
3.00
3.50
4.00
4.50

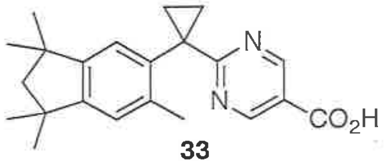
N
N
CO_2H

33

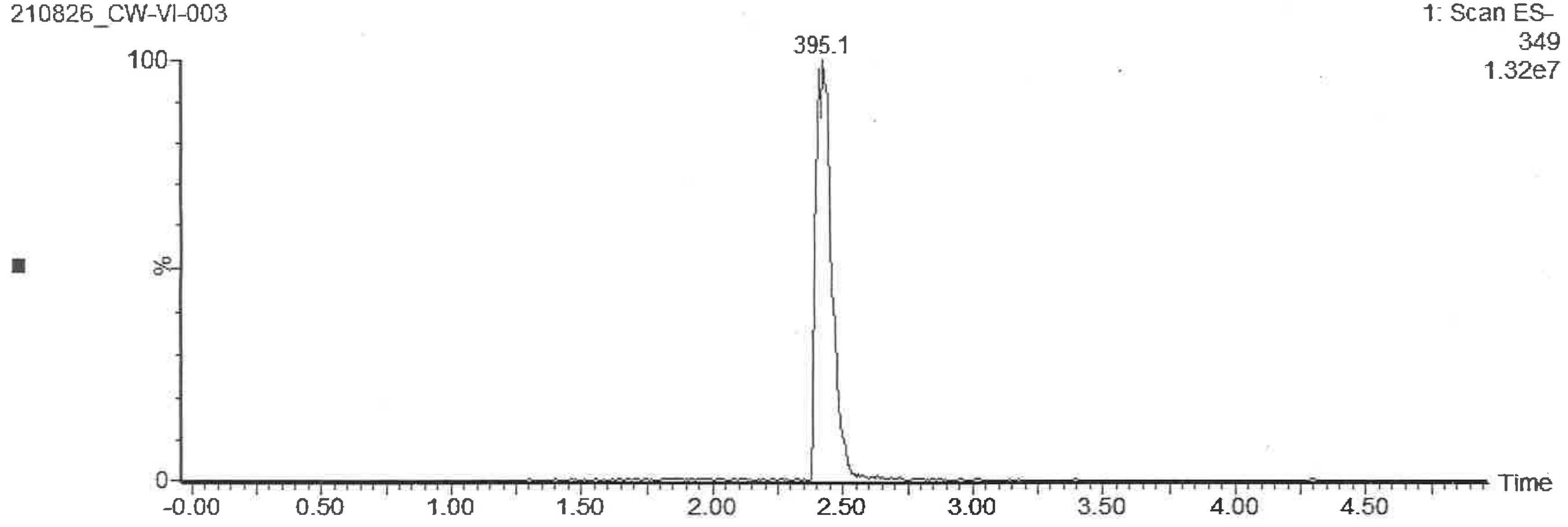
210826_CW-VI-003
1: Scan ES-
349
1.32e7
395.1
100
%
0
-0.00
0.50
1.00
1.50
2.00
2.50
3.00
3.50
4.00
4.50
Time

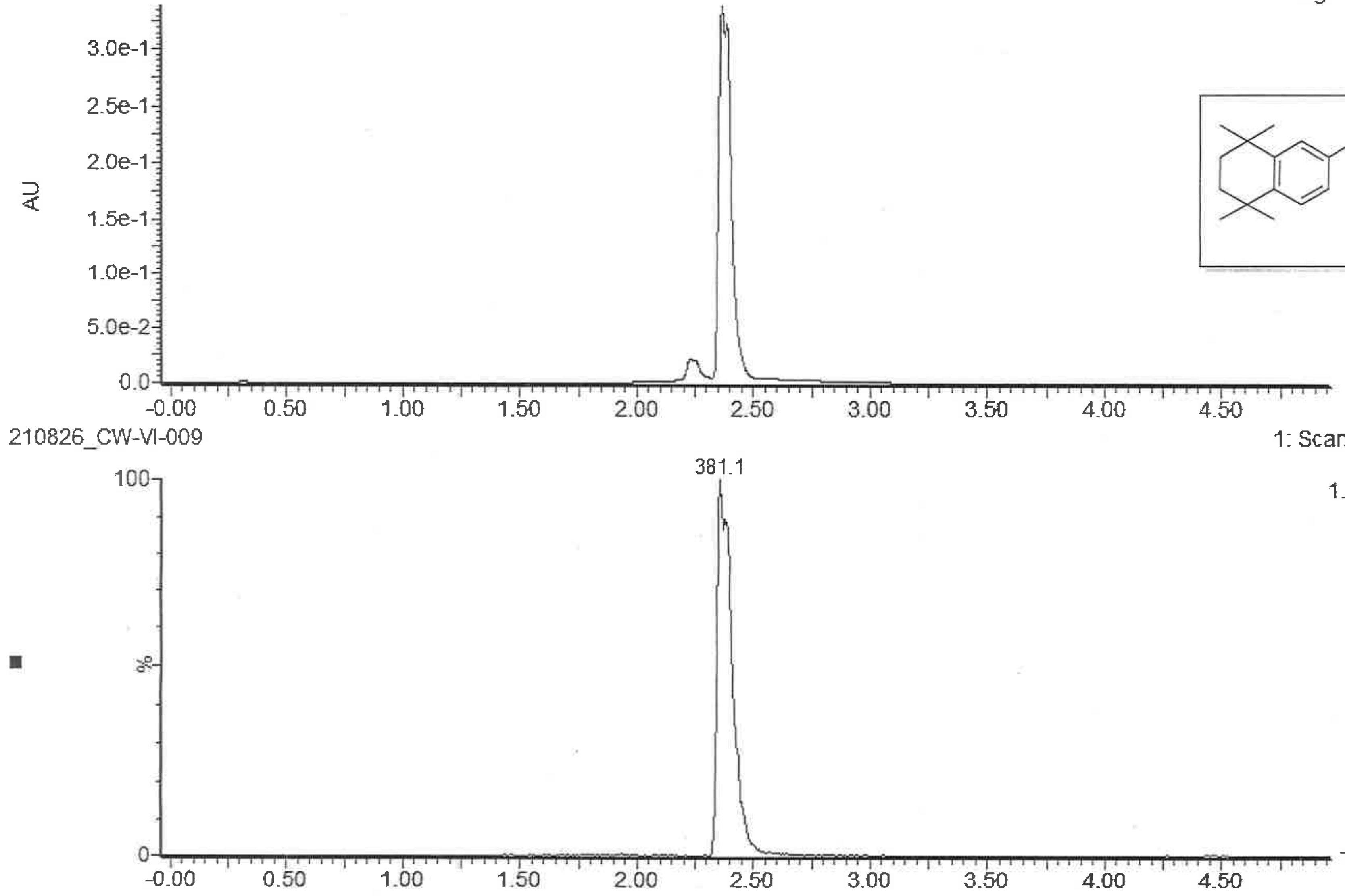
210826_CW-VI-009
(2) PDA Ch2 260nm@2.4nm
Range: 3e-1
AU
3.0e-1
2.5e-1
2.0e-1
1.5e-1
1.0e-1
5.0e-2
0.0
-0.00
0.50
1.00
1.50
2.00
2.50
3.00
3.50
4.00
4.50
210826_CW-VI-009
1: Scan ES-
335
1.73e7
381.1
100
%
0
Time

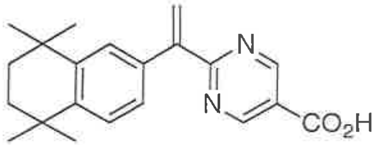
N
N
CO2H

34

210826_CW-VI-013

(2) PDA Ch2 260nm@2.4nm
Range: 4e-1

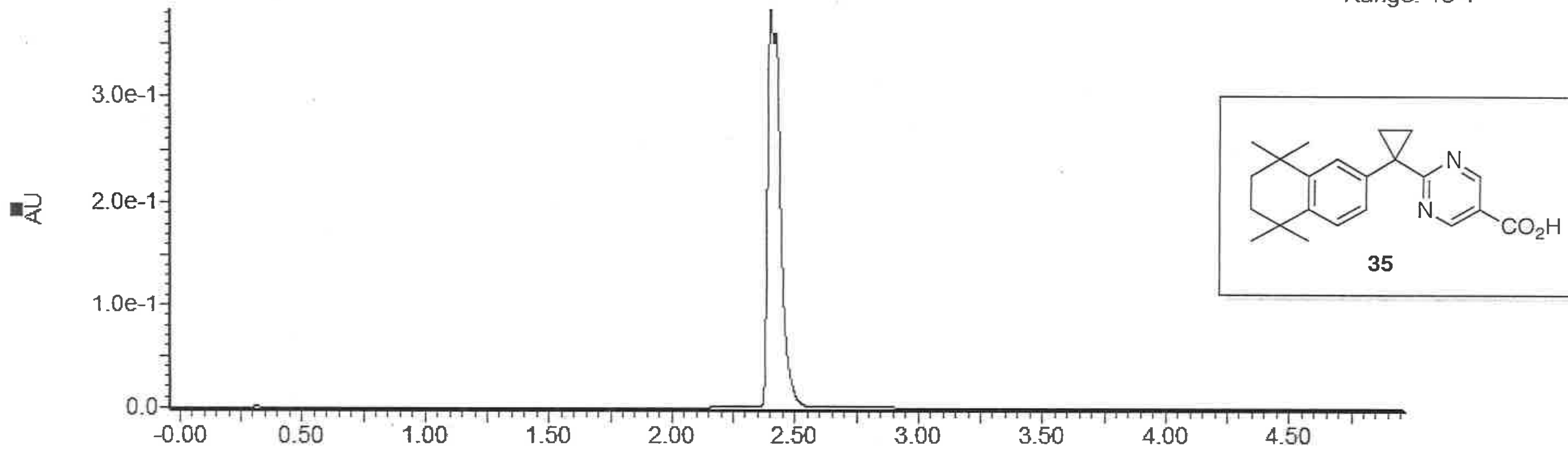

35

210826_CW-VI-013

1: Scan ES-
349
1.46e7

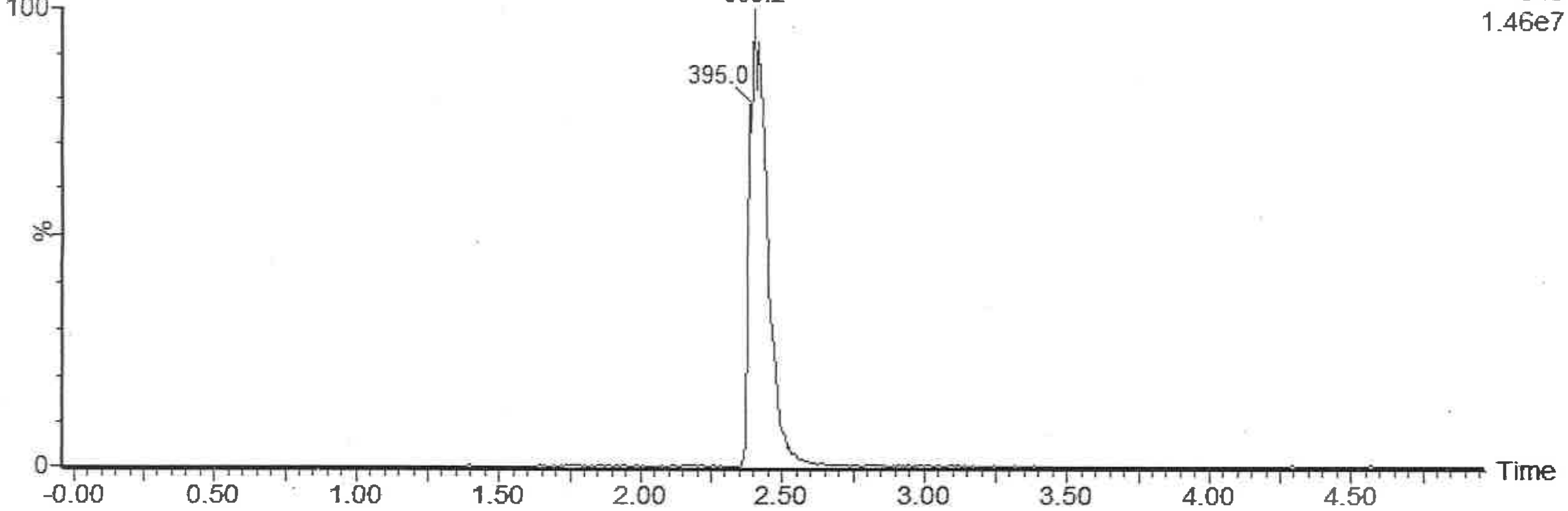

weak

210826_CW-VI-023_pos

(2) PDA Ch2 260nm@2.4nm
Range: 1

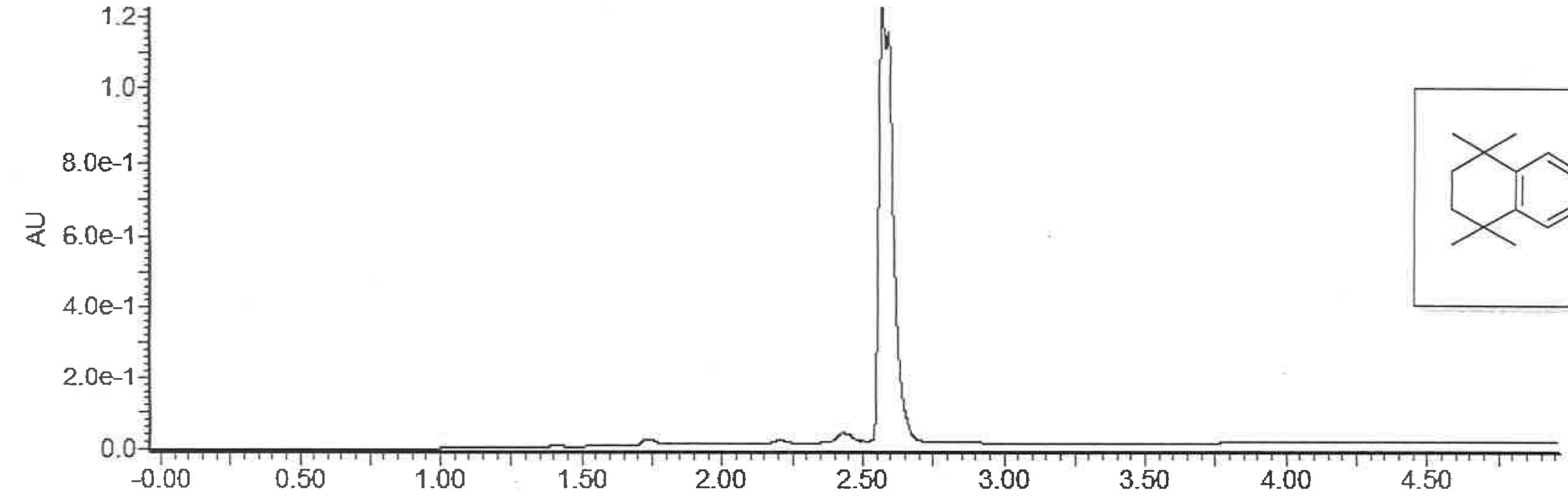

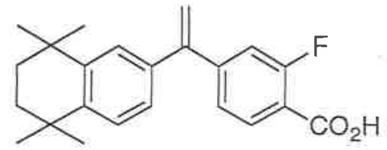

36

210826_CW-VI-023_pos

1: Scan ES+
353
2.84e7

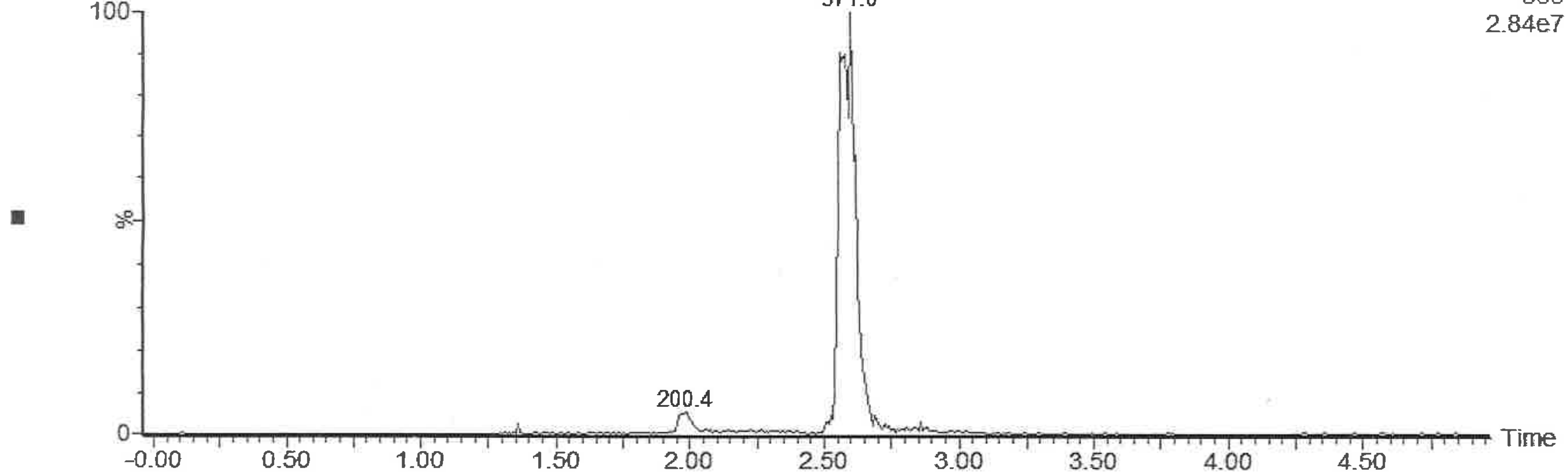

210826_CW-VI-073

(2) PDA Ch2 260nm@2.4nm
Range: 8e-1

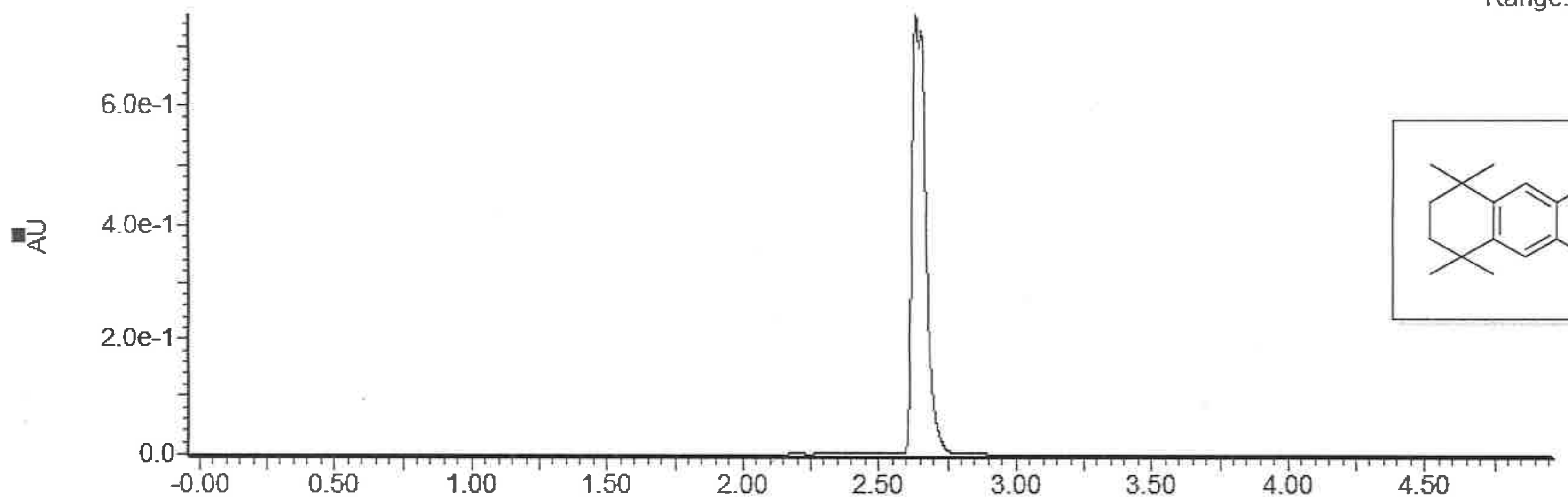

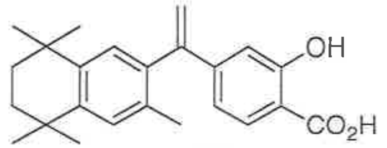

37a

210826_CW-VI-073

1: Scan ES-
363
9.63e7

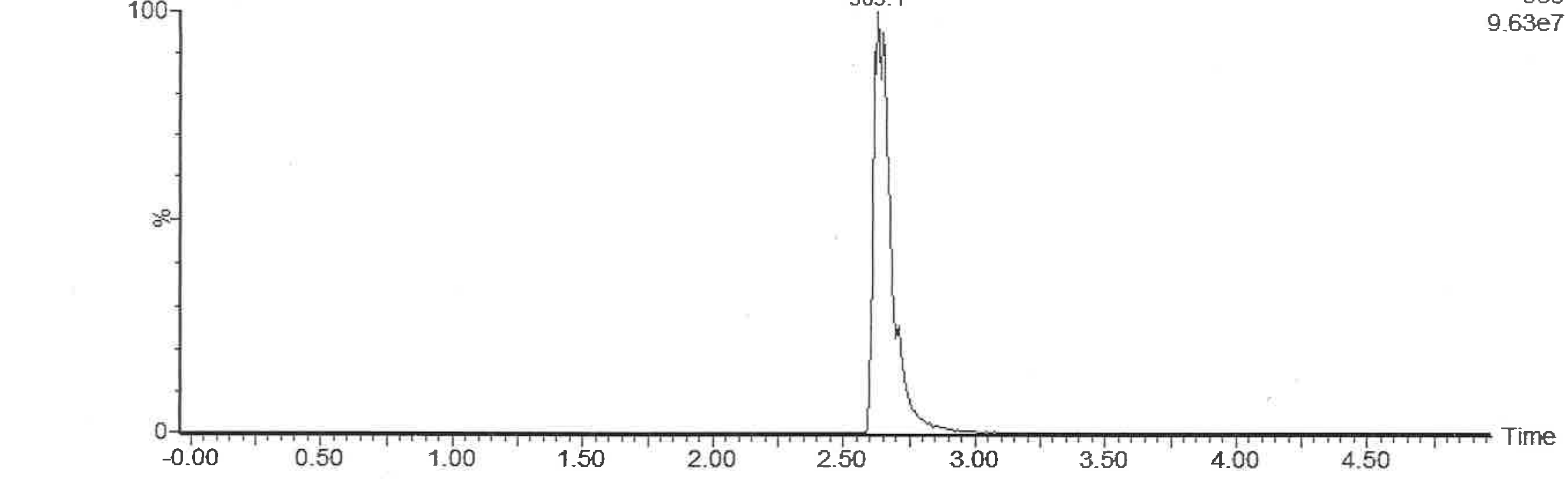

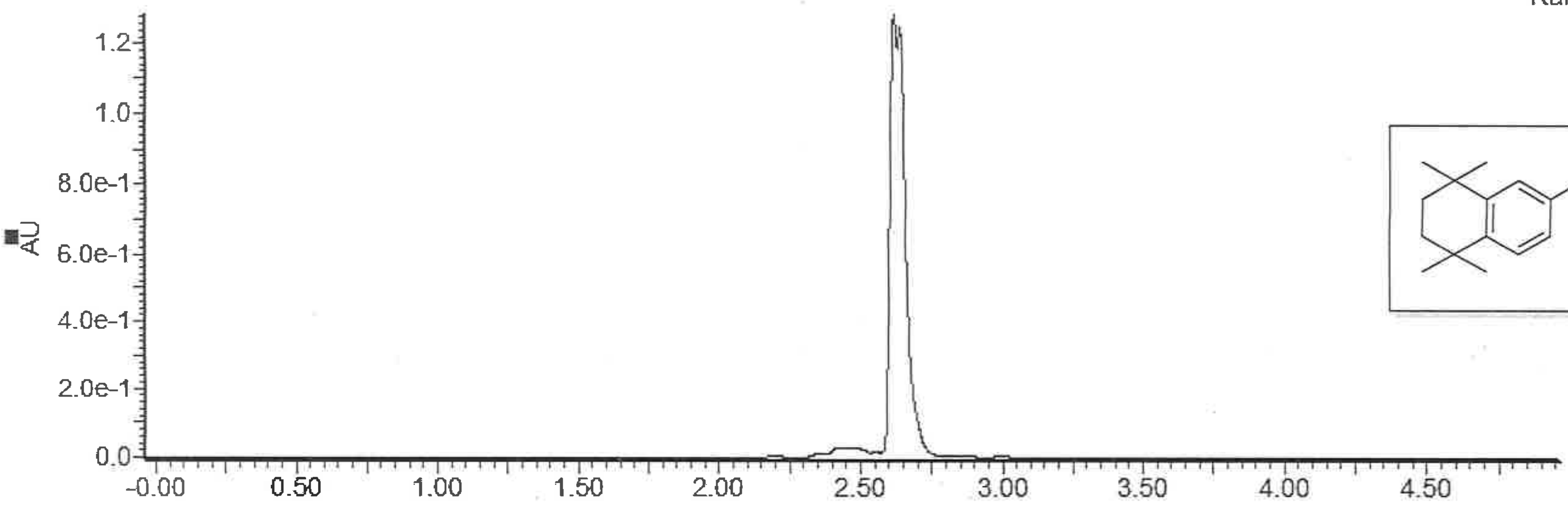
210826_CW-VI-103
(2) PDA Ch2 260nm@2.4nm
Range: 1
AU
1.2
1.0
8.0e-1
6.0e-1
4.0e-1
2.0e-1
0.0
-0.00
0.50
1.00
1.50
2.00
2.50
3.00
3.50
4.00
4.50

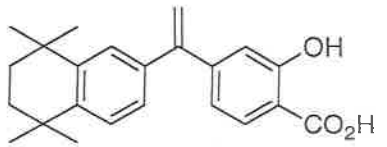
OH
CO_2H

37b

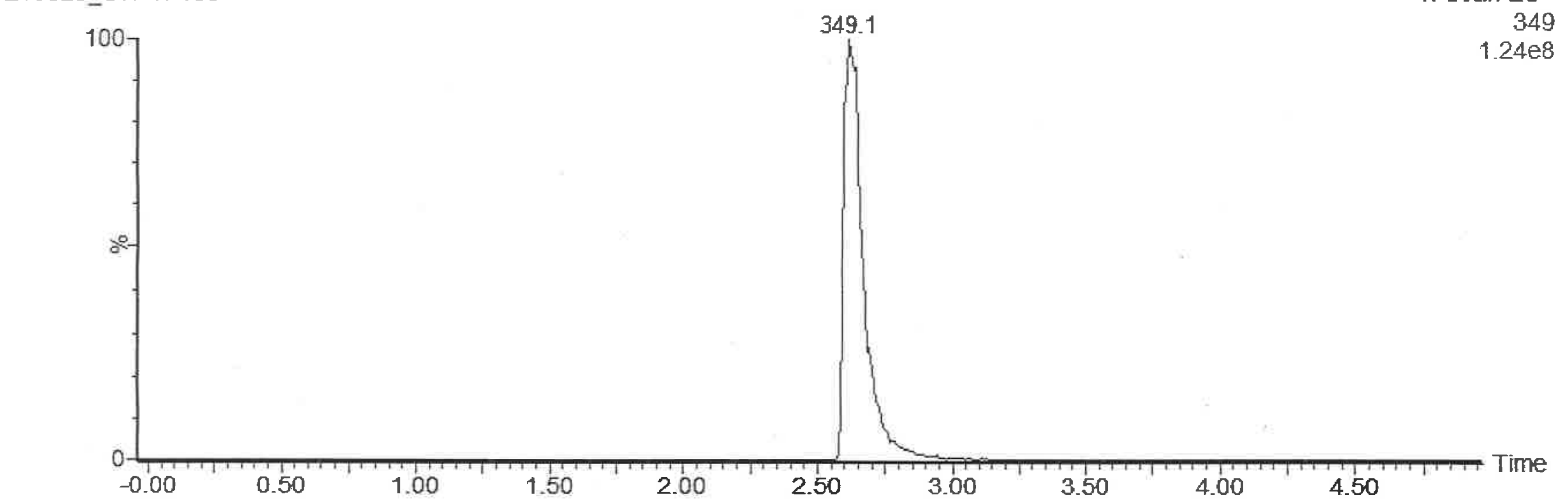
210826_CW-VI-103
1: Scan ES-
349
1.24e8
349.1
100
%
0
-0.00
0.50
1.00
1.50
2.00
2.50
3.00
3.50
4.00
4.50
Time
